# RNA-Seq data provide new insights into the molecular regulation of breast muscle glycogen reserves, a key factor in muscle function and meat quality in chickens^☆^

**DOI:** 10.1016/j.psj.2025.105136

**Published:** 2025-04-04

**Authors:** Philippe Bochereau, Sarah Maman Haddad, Elisabeth Le Bihan-Duval, Cécile Berri

**Affiliations:** a INRAE, Université de Tours, BOA, 37380 Nouzilly, France; b SIGENAE, Université de Toulouse, GenPhyse, INRAE, ENVT, F-31326, Castanet Tolosan, France

**Keywords:** Chicken, Muscle, Gut, RNA-Seq, Glycogen

## Abstract

Research is needed to better understand the molecular mechanisms that influence muscle glycogen reserves in chickens due to their critical influence on muscle function and meat quality. In this study, breast muscle RNA sequencing data (**RNA-Seq**) were used to compare the transcriptomic profile of two original chicken lines divergently selected for breast muscle ultimate pH, which is a proxy for glycogen reserves. Weighted gene co-expression network analysis (**WGCNA**) of muscle and jejunum RNA-Seq data was also performed to highlight biological processes specifically involved in the gut-muscle dialogue that may contribute to the divergence in glycogen reserves between the two lines. Breast muscle RNA-Seq analysis of 4-week-old birds from the 15th generation of selection, in which glycogen reserves in the pHu- line were twice as high as that in the pHu+ line, revealed 2676 differentially expressed genes (*P*_*adj*_ ≤ 0.05). Functional analysis of the genes overexpressed in the pHu- line highlighted enrichment in processes related to energy production from a wide range of substrates and pathways, as well as to processes involved in development of blood and lymph tissue. Diverse processes were enriched for genes overexpressed in the pHu+ line: muscle development and remodeling, lipid metabolism, immune response and inflammation, which suggested molecular changes much larger than those for carbohydrate metabolism. WGCNA revealed 64 modules of co-expressed genes. One, which contained 30 % genes expressed in the jejunum and 70 % genes expressed in the muscle, was correlated (*P* ≤ 0.05) with muscle glycogen reserves and several indicators of intestinal anatomy and health. Functional analysis of it showed an enrichment of processes related to transmission of nerve information and tissue oxygenation that seem to be involved in the gut-muscle dialogue that mediates establishment of breast muscle glycogen reserves. Finally, the study found that transcriptional regulations observed in muscle of the pHu+ line were similar to those in muscle afflicted with “wooden breast”, which highlighted a dysfunction of mitochondrial metabolism and suggested several potential gene markers for both conditions.

## Introduction

Since 2017, poultry has been the predominant meat produced in the world, and it is expected to represent nearly half of the total increase in meat production over the next decade (OECD/FAO, 2020). Several factors have influenced the success of poultry meat, such as its relatively low price, absence of religious barriers to it, and its healthy image and convenience. The poultry sector has transformed greatly in recent decades to adapt to changing consumption habits, with most chicken (*Gallus gallus*) meat being supplied as portions or processed products rather than whole carcasses. Technological quality has become essential to the competitiveness of the poultry industry, since it influences processing yields and several factors that influence meat purchases, such as color and texture.

In both chickens and pigs, differences in the onset of *rigor mortis* can significantly influence the technological quality of their meat. Specifically, a low ultimate pH (**pHu**) (measured 24 h after slaughter) results in acidic meat, also referred to as PSE-like (pale, soft, exudative) meat, with a pale color and lower water-holding capacity, while high pHu results in dark, firm, and dry (**DFD**) meat with a dark color and low storage quality (Allen et al., 1997; Barbut et al., 2008). For the pHu of meat from the breast (i.e., *Pectoralis major* muscle), an almost perfect genetic correlation (r_g_ = −0.97) with the glycolytic potential (**GP**) has been observed in chickens, which indicates that pHu is genetically determined by the glycogen reserves in the muscle at the time of death (Le Bihan-Duval et al., 2008). Selecting for increased growth and breast meat yield in standard chickens used for the portion and processed-product markets has been found to increase muscle-fiber hypertrophy but decrease glycogen reserves (Berri et al., 2007; Le Bihan-Duval et al., 2008). These histological and metabolic changes have resulted in breast myopathies such as “white striping” (**WS**) and “wooden breast” (**WB**), which are characterized by degeneration and regeneration of muscle fibers and increased deposition of fat (lipidosis) and connective tissue (fibrosis). Several omics studies have demonstrated a connection to carbohydrate metabolism by observing lower glycogen reserves in the breast muscle of chickens afflicted by WB (Abasht et al., 2016; Kuttappan et al., 2017). Other indirect evidence of reduced glycolytic capacity was lower glycogen reserves and higher pHu of the breast muscle of WB-afflicted chickens (Abasht et al., 2016).

Despite the critical role of muscle glycogen in maintaining optimal muscle function and meat quality, the factors that influence it in chickens remain less well known than those in mammals. To create chickens useful for physiological and genetic studies, divergent selection for breast muscle pHu, a proxy for muscle glycogen reserves, was initiated 16 years ago from a base population of fast-growing chickens. This selection experiment was extremely effective: after 6 generations, 61 % of breast muscle samples from the low-pHu (**pHu-**) line were PSE (pHu < 5.7), while 63 % of those from the high-pHu (**pHu+**) line were DFD (pHu > 6.1). A smaller, but still highly significant, difference was observed in the pHu of their thigh muscles, which indicated a general rather than specific effect of divergent selection on muscle metabolism (Alnahhas et al., 2014). Metabolomic and microarray analyses of 6-week-old chickens in the 6th generation of selection showed that the pHu- line had an overexpression of most genes involved in glycolysis/gluconeogenesis and an overabundance of carbohydrates in blood and muscle, while the pHu+ line had an overexpression of genes involved in muscle development and of metabolites related to oxidative stress, muscle proteolysis, and lipid β-oxidation (Beauclercq et al., 2016, 2017). The pHu+ line also had a higher frequency of WS, which is associated with more lipidosis (Alnahhas et al., 2016). Lipidomic analyses of the 14th generation of selection also revealed different circulating lipid profiles in chickens 17 days old (Beauclercq et al., 2022), when muscle glycogen reserves already differ between the two lines (Métayer-Coustard et al., 2021).

Genetic studies performed after several generations of selection highlighted polygenic and complex factors that influence meat pHu, although several candidate genes associated with human glycogen-storage diseases were identified (Bihan-Duval et al., 2018). Recent results for pHu-divergent lines have suggested that digestive functions could influence subsequent differences in muscle glycogen. Moreover, daily body weight and feed intake of chickens in the 14th generation of selection indicated that, although those in the pHu+ line consumed more feed from 23 days onwards, they had lower body weight and a higher feed conversion ratio (Berger et al., 2022). After 15 generations of selection, the upper part of the gastrointestinal tract (proventriculus plus gizzard) had developed more and the digestibility of calcium and nitrogen was higher in the pHu- line than those in the pHu+ line. These differences were accompanied by transcriptional regulations in the jejunum that involved local or more central processes, such as the immune response, tissue morphogenesis, lipid metabolism, and regulation of food intake, which could influence the intake and/or metabolic use of nutrients (Bochereau et al., 2024).

To better understand the complexity of factors that influence muscle glycogen in chickens, we used breast muscle RNA-sequencing (**RNA-Seq**) data to perform differential analysis of the two lines after 15 generations of selection. We also performed integrated gene-network analysis that included the jejunal transcriptome to highlight processes specifically involved in the gut-muscle dialogue that may have contributed to the divergence between the two lines.

## Materials and methods

All animal care and experimental procedures in this study complied with the French Animal Welfare Act and received approval from the Ethics Committee of the INRAE Centre Val de Loire under project reference no. APAFIS#30842-2021040107513853 v3. The Ethics Committee is registered with the French National Committee under no. D371751. This research was reported in accordance with the ARRIVE guidelines.

### Experimental design and sample collection

The chickens originated from two genetically distinct lines (pHu+ and pHu-) that had been selectively bred by INRAE for 15 generations based on the ultimate pH (pHu) of the *Pectoralis major* muscle. This selection process led to significant differences in muscle glycogen reserves and meat quality (Alnahhas et al., 2014). A total of 80 chicks (40 per line) were hatched and raised at the PEAT INRAE Poultry Experimental Facility (2018, https://doi.org/10.15454/1.5572326250887292E12). Chickens from both lines were kept together in a single 17.5 m² room, furnished with three circular feeders and a 3-meter-long nipple drinker line, until they reached 29 days of age. Rearing temperature was 32°C until 2 d, reduced by 1°C every other day to achieve 21°C after 22 d of age. Humidity was maintained between 50 and 70 %. The lighting program was maintained at a constant 40 lx for 24 h of light until day 7, then adjusted to a cycle of 18 h of light and 6 h of darkness from day 8 to day 29. During the experiment, birds were watered and fed *ad libitum*. On Day 28, all birds were weighed, and 15 chickens per line were selected based on their pedigree to ensure a broad representation of families within each line. These selected birds were then euthanized at day 29 using a lethal dose of Dolethal. *Pectoralis major* tissue was immediately collected, rapidly frozen in liquid nitrogen, and stored at -80°C for molecular analysis. The same birds were also used for detailed phenotypic analysis of the digestive tract and RNA-Seq analysis of the central part of the jejunum (Bochereau et al., 2024).

### Preparation of the Pectoralis major for RNA-Seq analysis

Total RNA was extracted from 30 mg of *Pectoralis major* tissue using the NucleoSpin RNA kit (Macherey-Nagel, Nucleospin RNA, ref: 740955.50), following the manufacturer's instructions. The tissue was first ground under liquid nitrogen. Due to its complexity, an additional step was included during lysis, in which 600 μL of lysis buffer RA1 and 450 μL of ethanol were added. RNA quality was evaluated using a NanoDrop ND-1000 spectrophotometer and further assessed on an Agilent RNA Nano Assay chip (Agilent RNA 6000 Nano Reagents, ref: 5067-1511, Agilent Technologies) after separation in agarose gels, using an Agilent Bioanalyzer 2100 (Agilent Technologies). All RNA samples had integrity numbers (RIN) above 8.6. The RNA was then sequenced at 2 × 150 bp on the NovaSeq 6000-A00318 platform (Get-PlaGe facility, Toulouse, France) and multiplexed according to the standard Illumina XP sequencing protocol.

### RNA sequencing and differential gene expression analyses

The Nextflow nf-core/rnaseq analysis pipeline (Nextflow v21.10.6, nf-core/rnaseq revision 3.5) was utilized with version 7b release 109 of the *Gallus gallus* reference genome (Gallus_gallus.bGal1.mat.broiler.GRCg7b.dna.toplevel.fa) and gene annotation file (Gallus_gallus.bGal1.mat.broiler.GRCg7b.109.gtf). Sequence alignment and transcript quantification were performed using STAR (Dobin et al., 2013) (v2.6.1d) and Salmon (Patro et al., 2015) (v1.5.3), respectively, applying each tool's default settings. RNA-Seq data were analyzed using generalized linear models implemented in the DESeq2 package (Varet et al., 2016) (v1.7.3) within the Galaxy Migale platform (MIGALE, 2018). For each gene, the line effect size was estimated as log2(FC), and fold change (FC) in expression was determined as 2 × log2(FC). Genes were considered differentially expressed (DE) between the two lines if their p-values, adjusted for false discovery rate using the Benjamini-Hochberg method, were less than or equal to 0.05.

### Gene co-expression network analysis

To construct gene co-expression networks that were significantly associated with phenotypic traits of interest, we used the Weighted Gene Correlation Network Analysis (WGCNA) (v. 1.72-5) package of R software (Core Team, 2024) (v. 4.3.3) and two matrices of gene expressions (one per tissue). The expression matrices included 13,416 genes expressed in the jejunum and 14,741 genes expressed in the *Pectoralis major* for the same 30 animals. A gene was considered expressed if it was counted at least once in 80 % of individuals in either line. Before the network analysis, the two matrices were merged into a single list of the 28,157 gene expressions in both tissues. From the gene-expression matrix, a Pearson correlation between each pair of genes was calculated and raised to the power of β = 12, based on the pickSoftThreshold function, to obtain a scale-free topology index (R²) of at least 0.70.

Genes were hierarchically clustered by calculating a dissimilarity measure (i.e., topological overlap matrix) and applying a module-height threshold to the branches of the hierarchical tree. This method yielded a dendrogram whose clusters were identified using the dynamicTreeCut algorithm with a sensitivity threshold set to 3. The minimum module size was set to 30 genes. WGCNA's moduleEigengenes function was used to represent each module by its eigengene, which was used to establish module-trait relations. Modules that had similar expression profiles (*r* ≥ 0.90) were merged due to the high probability that their genes were highly co-expressed.

Module-trait relations were estimated by calculating a Pearson correlation between each module's eigengene and the following muscle or digestive traits: *Pectoralis major* glycogen reserves estimated by glycolytic potential (GP) calculation, digestive tract morphology (ratio of the proventriculus plus gizzard, duodenum, jejunum, and ileum to body weight), jejunum structure (i.e., crypt depth, mucosal thickness, and villus height), digestive efficiency (i.e., ileal digestibility of calcium, phosphorus, and nitrogen), and health status of the jejunum (i.e., alkaline phosphatase and antioxidant status) (Bochereau et al., 2024).

### Functional annotation and enrichment analysis of genes

Gene ontology analysis was performed using the Visualization, Semantic Similarity, and Enrichment Analysis of Gene Ontology (**ViSEAGO**) package (Brionne et al., 2019) version 1.16.0. GO annotations for *Gallus gallus* (ID = “9031″) were obtained from EntrezGene through ViSEAGO's EntrezGene2GO function, followed by functional annotation. The ViSEAGO package utilizes the topGO tool to assess the enrichment of GO terms in gene sets of interest. We applied a node size of 10 to analyze the enrichment of DE genes and a node size of 5 for module enrichment after conducting WGCNA, focusing on the “biological process” GO category and using Fisher's exact test with the elimination (elim) algorithm. A P-value threshold of < 0.01 was used to determine statistical significance. The results of the enrichment analysis were then hierarchically clustered based on Wang's semantic similarity distance and the ward.D2 aggregation method. A heatmap was created to visualize the clustering, with GO terms ordered according to their functional similarity, and the significance of GO-term enrichment was represented as a color gradient (log10(P)).

## Results

### Transcriptomic profiles of the Pectoralis major in the two lines

We used RNA-Seq to obtain 34-86 million sequences per sample. To align these sequences with the GalGal7 reference genome for chickens in the Ensembl database, we used the STAR tool, which yielded excellent alignment rates (i.e., 76-82 %). Among the sequences aligned, we identified a mean of 50 million unique sequences in the samples. All of the aligned sequences had excellent FastQC quality (i.e., ≥ 33.6). In total, we identified 22,950 genes expressed in the *Pectoralis major*. Statistical analyses using the *DESeq2* package identified 2676 genes that were differentially expressed (**DE**) between the two pHu lines. Of the 2676 DE genes, 1520 genes were overexpressed in the pHu+ line, of which 12 were also DE in the jejunum of the same animals (Bochereau et al., 2024), while 1156 were overexpressed in the pHu- line, of which 18 were also DE in the jejunum of the same animals (Additional Table 1). Of these 20 common DE genes, only 2 were not regulated in the same way in both tissues. Of the 2676 DE genes, 1547 (58 %) were identified by a known name, while the remaining 1129 were classified as novel. The fold change (**FC**) (pHu-/pHu+) of DE genes (*P*_*adj*_ ≤ 0.05) ranged from 0.041 to 0.926 for genes down-regulated in the pHu- line, and from 1.076 to 72.77 for up-regulated genes. See Table 1 for the 15 overexpressed genes with the highest FC in each line.

**Table 1:** The 15 protein-coding genes most overexpressed in the *Pectoralis major* muscle of the pHu- and pHu+ lines of chickens.

ENSEMBL gene ID	Symbol	Description	FC (pHu-/pHu+)	Adjusted P-value
Up-regulated genes in the pHu- line				
ENSGALG00010001126	MINDY4B	MINDY Family Member 4B	42.404	5.76E-05
ENSGALG00010005198	TRPC3	Transient Receptor Potential Cation Channel Subfamily C Member 3	10.562	4.46E-10
ENSGALG00010003469	ZPAX	Zona pellucida protein	4.985	6.76E-03
ENSGALG00010008434	PTCHD4	Patched Domain Containing 4	4.862	1.74E-02
ENSGALG00010012371	ATP6V1G3	ATPase *H*+ Transporting V1 Subunit G3	4.785	1.11E-09
ENSGALG00010023773	OVOA	Ovomucin. alpha subunit	3.728	1.35E-08
ENSGALG00010018790	PRR35	Proline Rich 35	3.68	3.23E-02
ENSGALG00010005345	NOX3	NADPH Oxidase 3	3.57	1.00E-02
ENSGALG00010013423	ZNF804A	Zinc Finger Protein 804A	2.817	1.24E-02
ENSGALG00010001358	TCERG1L	Transcription Elongation Regulator 1 Like	2.723	1.80E-02
ENSGALG00010015904	MTMR7	Myotubularin Related Protein 7	2.721	2.27E-06
ENSGALG00010013296	AGXT2	Alanine–Glyoxylate Aminotransferase 2	2.594	1.01E-06
ENSGALG00010009106	THSD7B	Thrombospondin Type 1 Domain Containing 7B	2.545	5.28E-19
ENSGALG00010025555	CSRNP3	Cysteine And Serine Rich Nuclear Protein 3	2.531	3.04E-02
ENSGALG00010008824	GREM2	Gremlin 2. DAN Family BMP Antagonist	2.511	1.17E-08
Up-regulated genes in the pHu+ line				
ENSGALG00010016709	DNASE1	Deoxyribonuclease 1	0.062	3.22E-04
ENSGALG00010002294	DAZL	Deleted In Azoospermia Like	0.081	2.83E-02
ENSGALG00010016627	SH3RF2	SH3 Domain Containing Ring Finger 2	0.091	2.65E-10
ENSGALG00010013765	PRLL	Prolactin like	0.115	1.73E-03
ENSGALG00010004133	CRH	Corticotropin Releasing Hormone	0.128	2.91E-04
ENSGALG00010012923	MLANA	Melan-A	0.13	2.15E-03
ENSGALG00010008092	SPIRE2	Spire Type Actin Nucleation Factor 2	0.143	1.97E-03
ENSGALG00010004296	DSG1	Desmoglein 1	0.144	4.92E-02
ENSGALG00010004386	CFAP44	Cilia And Flagella Associated Protein 44	0.148	5.58E-06
ENSGALG00010014963	KMO	Kynurenine 3-Monooxygenase	0.151	3.54E-04
ENSGALG00010002960	BLEC2	C-type lectin-like receptor 2	0.16	1.57E-06
ENSGALG00010005122	IL8L1	Interleukin 8-like 1	0.163	1.22E-03
ENSGALG00010000757	SCEL	Sciellin	0.176	1.97E-09
ENSGALG00010010871	GCNT2	Glucosaminyl (N-Acetyl) Transferase 2 (I Blood Group)	0.19	3.44E-02
ENSGALG00010012750	CAPSL	Calcyphosine Like	0.201	4.14E-03

To better understand the biological processes associated with DE genes, we performed functional analysis based on gene ontology (**GO**) of the genes overexpressed in each line. Of the 2676 DE genes, 1066 were associated with 184 enriched GO terms (*P* *<* 0.001), including 130 GO terms associated mainly with genes up-regulated in the pHu+ line, 53 with genes up-regulated in the pHu- line, and 1 with genes up-regulated in both lines (Additional Table 2).

**Fig. 1:**
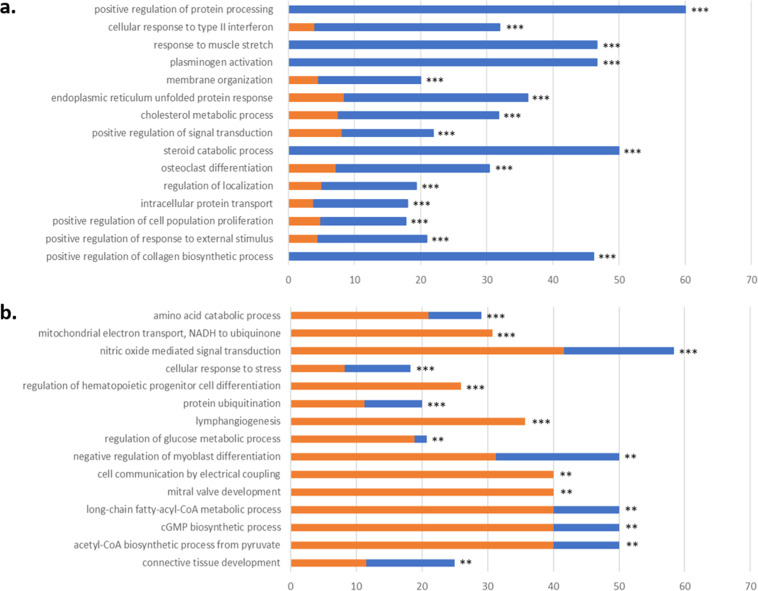
ViSEAGO functional enrichment analysis of biological processes of the genes up-regulated in the *Pectoralis major* muscle of the pHu+ and pHu- lines of chickens: the 15 most significant gene ontology (GO) terms for the (A) pHu+ line and (B) pHu- line. For each GO term, blue and orange bars indicate the percentage of genes of the GO term overexpressed in the pHu+ or pHu- line, respectively. Significance levels of enrichment: *** *P* ≤ 0.001 and ** *P* ≤ 0.01.

Among the 15 most significantly enriched GO terms associated with the pHu+ line (Fig. 1a), 5 GO terms contained only genes up-regulated in the pHu+ line: positive regulation of protein processing, response to muscle stretch, plasminogen activation, steroid catabolic process, and positive regulation of collagen biosynthetic process. For these 5 GO terms, the genes overexpressed in the pHu+ line represented 46-60 % of the genes associated with the GO term. The other 10 GO terms contained mainly genes up-regulated in the pHu+ line, some of which were also up-regulated in the pHu- line: cellular response to type II interferon, membrane organization, endoplasmic reticulum unfolded protein response, cholesterol metabolic process, positive regulation of signal transduction, osteoclast differentiation, regulation of localization, intracellular protein transport, positive regulation of cell population proliferation, and positive regulation of response to external stimulus. Similarly, of the 15 most significantly enriched GO terms associated with the pHu- line (Fig. 1b), 5 contained only genes up-regulated in the pHu- line: mitochondrial electron transport/NADH to ubiquinone, regulation of hematopoietic progenitor cell differentiation, lymphangiogenesis, cell communication by electrical coupling, and mitral valve development. For these 5 GO terms, the genes overexpressed in the pHu- line represented 26-40 % of the genes associated with the GO term. The other 10 GO terms contained mainly genes up-regulated in the pHu-, of which a smaller percentage were also up-regulated in the pHu+ line: amino acid catabolic process, nitric oxide mediated signal transduction, cellular response to stress, protein ubiquitination, regulation of glucose metabolic process, negative regulation of myoblast differentiation, long-chain fatty-acyl-CoA metabolic process, cGMP biosynthetic process, acetyl-CoA biosynthetic process from pyruvate, and connective tissue development.

### Integrated analysis of Pectoralis major muscle and jejunal transcriptomic data

To explore potential interactions between the *Pectoralis major* muscle and jejunum, we performed WGCNA of 13,416 and 14,741 genes expressed in the *Pectoralis major* and jejunum, respectively. We investigated correlations between module eigengenes and muscle glycogen reserves, as well as several digestive phenotypic traits measured in the two lines (Bochereau et al., 2024).

The WGCNA identified 64 modules of co-expressed genes that ranged in size from MEcyan (i.e., 4690 genes) to MEdarkgoldenrod4 (i.e., 30 genes). Of the 64 modules, 10 contained genes expressed only in the *Pectoralis major*, 7 contained genes expressed only in the jejunum, and 47 contained genes expressed in both tissues (mostly with large differences in the contribution of both transcriptomes).

**Fig. 2:**
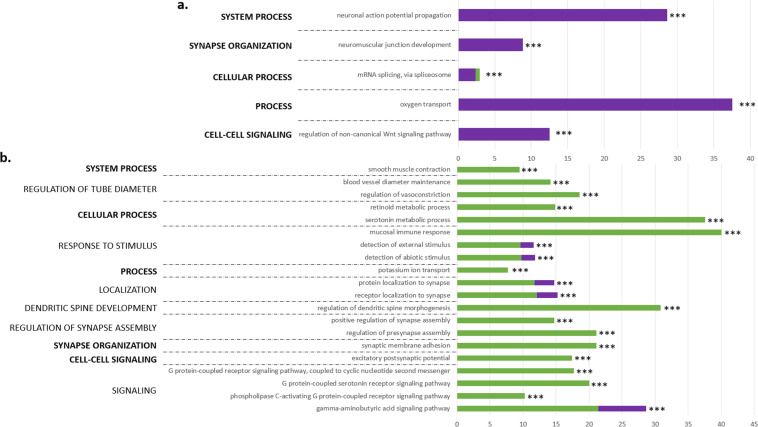
ViSEAGO functional enrichment analysis of biological processes of the MEgrey module that contained genes expressed in the jejunum and *Pectoralis major* muscle of chickens: (A) 5 most significant GO terms for the jejunal genes and (B) 20 most significant GO terms for the *Pectoralis major* genes. Bold text identifies the name of the Bayesian model averaging clustering common to both transcriptomes (jejunum and *Pectoralis major*). For each GO term, purple and green bars indicate the percentage of genes of the GO term expressed in the jejunum or muscle, respectively. Significance level of enrichment: *** *P* ≤ 0.001.

Correlations between module eigengenes and phenotypic traits indicated that 7 modules (MEdarkolivegreen1, MEmediumpurple3, MEdarkseagreen3, MElightgreen, MEchocolate4, MElightblue2, and MEgrey) correlated significantly (*P* ≤ 0.05) with *Pectoralis major* muscle GP. Of these 7 modules, only 2 (MEgrey and MElightblue2) contained significant number of genes expressed in both the *Pectoralis major* and jejunum. The MEgrey module was correlated with GP (*r* = 0.42, *P* *=* 0.02), percentage of proventriculus plus gizzard (*r* = 0.40, *P* *=* 0.03), jejunal alkaline phosphatase activity (*r* = 0.40, *P* *=* 0.03), and total antioxidant status (*r* = 0.37, *P* *=* 0.05). It contained 773 genes, of which 228 were expressed in the jejunum, 545 in the *Pectoralis major*, of which 21 in both tissues. Of the 228 genes expressed in the jejunum, 62 were included in a ViSEAGO enrichment analysis, which identified 5 GO terms (Fig. 2a). Of the 545 genes expressed in the *Pectoralis major*, 189 were included in enrichment analysis, which identified 46 GO terms (the 20 most significant in Fig. 2b). In comparison, the MElightblue2 module was correlated with GP (*r* = 0.36, *P* *=* 0.04) but not with the other phenotypic traits studied, and contained 29 genes expressed in the jejunum and 6 in the *Pectoralis major*. Due to this module's few genes, enrichment analysis was not performed.

## Discussion

### How divergent selection has affected a wide range of biological processes far beyond carbohydrate metabolism in muscle

To better understand how genes influence glycogen reserves in chicken breast muscle, which is a key factor that influences meat quality (Le Bihan-Duval et al., 2008) and animal robustness (Métayer-Coustard et al., 2021), we performed differential RNA-Seq analysis of the *Pectoralis major* muscle of two chicken lines (pHu+ and pHu-) that differed significantly in glycogen reserves after 15 generations of divergent selection (Bochereau et al., 2024). The analysis identified 2676 DE genes between the two lines, while a previous study of the same lines in the 6th generation of selection, when the glycogen reserves of the pHu+ line were only 20 % rather than 100 % as large, identified 1436 DE genes (Beauclercq et al., 2017). Although differences in technology (RNA-Seq vs. microarray), the reference genome (Ensembl vs. NCBI), genome version (Galgal7 vs. Galgal5), and age of the chickens (4 vs. 6 weeks old) prevented direct comparison of the DE genes, the functional enrichment analyses performed of the 6th or 15th generation highlighted losses or gains in biological processes that differentiated the lines over the course of selection. In generation 6, functional analysis of the muscle transcriptome of both lines revealed that many DE genes were involved in biological processes related to carbohydrate and energy metabolism, as well as muscle development and remodeling. Processes related to the intensive use of carbohydrate metabolism to produce energy were up-regulated in the pHu- line, while other catabolic processes such as protein degradation, muscle regeneration, and the response to oxidative stress were up-regulated in the pHu+ line (Beauclercq et al., 2017).

Nine generations later, metabolic processes remained associated with the divergence between the two lines. As observed in the 6th generation, several genes involved in regulating glucose metabolism were overexpressed in the breast muscle of the pHu- line in the 15th generation (Fig. 1, Additional Table 2). Interestingly, expression of other metabolic processes potentially involved in energy production in the pHu- line in the 15th generation involved a wider range of substrates, such as fatty acids (positive regulation of fatty acid metabolic process, long-chain fatty-acyl-CoA metabolic process, monocarboxylic acid metabolic process, dicarboxylic acid metabolic process, and diacylglycerol metabolic process) and amino acids (amino acid catabolic process, alpha-amino acid catabolic process, and sulfur amino acid biosynthetic process), and processes, such as mitochondrial activity (mitochondrial electron transport, NADH to ubiquinone, energy derivation by oxidation of organic compounds , acetyl-CoA biosynthetic process from pyruvate, ubiquinone biosynthetic process, and phosphate-containing compound metabolic process) and maintenance (mitochondrial genome maintenance). Among the most enriched GO terms for up-regulated genes in the pHu- line, several were related to development of blood and lymph tissue, such as the regulation of hematopoietic progenitor cell differentiation (regulation of hematopoietic progenitor cell differentiation), sprouting angiogenesis, valve development (mitral valve development, aortic valve development, pulmonary valve development, and heart valve morphogenesis), and lymphangiogenesis. Blood and lymph circulation in muscles plays a crucial role in maintaining their health and proper functioning. Blood circulation delivers oxygen and nutrients and removes metabolic waste products, and the response to nutrient levels (response to nutrient levels) was consistently enriched in the pHu- line (Additional Table 2). The lymphatic system drains fluids and detoxifies muscles, and is also involved in the immune response. These systems appear to be more efficient in the pHu- line, which allows muscles to function, be repaired, and regenerate better than in the pHu+ line, which has been found to have lower muscle capillary density (Alnahhas et al., 2015), greater muscle fiber regeneration (Beauclercq et al., 2017), and higher frequency and severity of WS (Alnahhas et al., 2016). The pHu+ line had many more enriched GO terms related to overexpressed genes than the pHu- line did (Additional Table 2), which indicated that glycogen depletion is associated with potential deregulation of many other biological processes besides carbohydrate metabolism. The 15 most enriched processes in the pHu+ line were related mainly to protein processing and signal transduction, plasminogen activation (which helps prevent excessive thrombosis and maintain blood circulation), response to muscle stretch, response to external stimulus, collagen biosynthesis, innate immune response, and lipid metabolic processes related to steroid and cholesterol. The differential analysis of the 6th generation highlighted up-regulation of biological processes involved in protein degradation and muscle remodeling in the pHu+ line, and results of the present study seemed to confirm that this still held true 9 generations later.

Interestingly, most of the 20 genes identified as good predictors of pHu in the 6th generation (Beauclercq et al., 2017) still differed in the 15th generation, and most of these latter genes were involved in muscle organization and physiology, but not directly in carbohydrate metabolism. Examples included thrombospondin type 1 domain-containing protein 7B (*THSD7B*), which is predicted to be involved in actin cytoskeleton reorganization; caveolin 3 (*CAV3*), which strongly influences muscle development and physiological processes, such as regulating signaling and mechanotransduction, as well as the pathophysiology of genetic myopathy (Berling et al., 2023); and ankyrin repeat domain 1 (*ANKRD1*), which is involved in regulating muscle development and regeneration in both humans and poultry. In contrast, lipid metabolism became one of the most important enriched biological processes for genes overexpressed in the pHu+ line in the 15th generation, which was consistent with recent results for 17-day-old chicks from both lines (Beauclercq et al., 2022) that showed a clear difference in circulating lipid profiles. It has been hypothesized that lipidosis in muscle (as observed in WS, which is more frequent and severe in the pHu+ line) may lead to lipotoxicity and oxidative stress, which in turn may lead to down-regulation of glycolysis and glycogenesis and redirection of glucose to other processes (Lake et al., 2019). Another interesting result of this long-term divergent selection was the enrichment of several processes related to the immune response and inflammation, as observed in the pHu+ line. These processes can help protect and repair muscle when the response is controlled and brief. However, chronic or excessive responses can degrade muscle health, particularly by breaking down muscles, and are likely to require energy in pHu+ muscles, which already do not have enough energy (Mukund and Subramaniam, 2020).

### Potential influence of the gut-muscle dialogue in controlling muscle glycogen

While several studies of the factors that influence meat quality in poultry have focused on understanding the associated molecular differences in muscles, few studies have explored interactions with other tissues, even though tissues in multicellular organisms do not function in isolation (Long et al., 2016). In the present study, we investigated molecular interactions between the intestines and muscles, since divergent selection for pHu has led to anatomical, histological, and metabolic differences in the jejunum, which is a major site of nutrient absorption (Bochereau et al., 2024). To identify genes that may be involved in the gut-muscle dialogue, we used WGCNA to identify co-expression networks in the two tissues that correlated with muscle glycogen reserves and with digestive phenotypic traits differential between the two lines. We identified several networks of genes co-expressed in the two tissues, but only two modules met the correlation conditions. One module (MEgrey) was especially interesting since it contained 30 % genes expressed in the jejunum and 70 % genes expressed in the breast muscle and was correlated with muscle glycogen reserves as well as intestinal anatomy and health, as assessed by measuring the ratio of the weight of the proventriculus plus gizzard to body weight, alkaline phosphatase activity, and antioxidant status of the jejunum. Functional enrichment analysis identified processes related to neuron functioning, blood circulation, and oxygen transport, of which synapse organization, post-synaptic GABA and serotonin receptor signaling pathways, and regulation of dendritic spine morphogenesis were most enriched in the muscle, while neuromuscular junction development, neuronal action potential propagation, and oxygen transport were most enriched in the gut (Fig. 2). Among the co-regulated genes expressed in the jejunum, *ANKRD6* and *SFRP5*, as negative regulators of the Wnt pathway, help maintain the balance between cell proliferation and differentiation, ensuring normal tissue growth and development. Others are involved in the development and function of the gut nervous system. *CACNB4*, which encodes an auxiliary subunit of voltage-gated calcium channels, is crucial for modulating synaptic transmission, neuronal excitability, and neurotransmitter release (Tadmouri et al., 2012; Li et al., 2020). *CNTNAP1*, involved in cell adhesion and myelin formation, is essential for nerve conduction and facilitates communication between neurons and glial cells. *RAPSN* encodes a protein necessary for acetylcholine receptor aggregation at the neuromuscular junction, enabling nerve impulse transmission and muscle contraction (Martínez-Martínez et al., 2009). Finally, the *HBA1, HBBA*, and *HBAD* genes, encoding the α, β, and δ subunits of hemoglobin, respectively, are involved in processes like hydrogen peroxide scavenging, cellular oxidant detoxification, and hydrogen peroxide metabolism, all crucial for repairing oxidative damage caused by reactive oxygen species (ROS). Additionally, they help reduce double-stranded DNA breaks, supporting DNA repair (Gafter-Gvili et al., 2013; Li et al., 2022). Altogether, these result suggest that the neural functions play a key role in coordinating metabolic functions and tissue development in response to physiological needs and in neuronal plasticity, which is essential to regulate energy metabolism effectively (Pénicaud et al., 2000). Glucose is a key signal to the nervous system that influences the perception of energy needs and coordinates the appropriate metabolic responses to maintain homeostasis (Fioramonti et al., 2007). Oxygenation and blood circulation are also essential for maintaining optimal neuronal function and energy production and are influenced by smooth-muscle contraction and maintenance of vascular diameter (Oberheim et al., 2012; Hayes et al., 2022). This integrated analysis thus suggests a potential relation between the efficiency of general processes (e.g., nerve-signal transmission, tissue oxygenation) and muscle energy metabolism, as well as indicators of the development and health of the digestive tract (e.g., alkaline phosphatase activity, antioxidant status). However, more research is needed to understand the factors that influence co-expressions in tissues (e.g., common genetic causes, inter-tissue regulation).

Direct comparison of the transcriptomic analyses of the *Pectoralis major* and jejunum of the same animals (Bochereau et al., 2024) identified 29 common DE genes between the two tissues, all of which were overexpressed in the same line in both tissues, except for *LIPA* and *TCERG1L*. Of the 10 DE genes with known functions, 5 were involved in the innate and adaptive immune responses, which highlights the importance of this function in differentiating the two lines. In chicken, there are two class II systems in chickens: a general *BLB2/DMB2* system in many tissues, and a more specialized *BLB1/DMB1* system expressed predominantly in the intestine and spleen (Parker and Kaufman, 2017). Very interestingly, *BLB2* is overexpressed in both the gut and muscle of the pHu+ line while *BLB1* is more expressed in the two tissues of the pHu- line. *BLB1* appears to play a more central role in the classical immune response by presenting antigens to helper T lymphocytes (CD4+), while *BLB2* may have a more specific and variable function depending on species and context, thereby influencing the diversity and effectiveness of immune responses. The impact of the balance in *BLB1* and *BLB2* expression on physiology, as well as digestive and muscle health, remains to be explored, raising intriguing questions. Among the immunity genes overexpressed in both muscle and gut of the pHu- line, *MHCYL* codes for a protein belonging to the MHC class I complex and play a role in modulating the immune response, especially in response to infections (Kim et al., 2024). Additionally, *DCLRE1C* is involved in V(D)J recombination in lymphocytes, which is a key process for immunological diversity and an enhanced adaptive immune response (Rohr et al., 2010), and *DLL4* in the notch signaling pathway, in which it regulates the development of innate lymphoid cells, helps form secondary lymphoid organs, and helps remodel tissue after injury or infection, thus playing a central role in the innate immune response (Pellegrinet et al., 2011; Nauman and Stanley, 2022). It is worthy to note that the three genes belonging to the MHC class I and II complexes, i.e., *BLB1, BLB2*, and *MHCYL* are all located in the Gga_rs15026791 genetic region associated with pHu reinforcing a potential role for them in determining glycogen reserve in muscle (Bochereau et al., 2024). These results confirm the hypothesis that immune differences, particularly in genes related to adaptation and defense mechanisms, may be a key factor in the differentiation of lines in terms of pHu and their physiological characteristics and meat quality.

Of the remaining 5 genes, 2 were involved in functions related to energy metabolism: *GATC*, which was overexpressed in the jejunum and muscle in the pHu+ line, is essential for mitochondrial protein synthesis (Nagao et al., 2009). This overexpression may be an attempt to adapt to mitochondrial dysfunction or stress. Indeed, when mitochondria experience problems (e.g., impaired energy function), the organism may compensate by increasing mitochondrial protein production to enhance energy production and restore cellular function. The gene *LIPA*, which was under expressed in the jejunum but overexpressed in the muscle of the pHu+ line, is involved in triglyceride degradation (Xu et al., 2023). In muscle, the overexpression of *LIPA* suggests an enhanced ability to use lipids as an energy source, which is essential for muscle contraction and growth. Muscle, particularly in fast-growing strains, is more dependent on lipid metabolism to meet the high energy demands associated with rapid tissue growth and physical activity. This dependency must be exacerbated by the lack of carbohydrate reserve that characterized the muscle of the pHu+ line (Beauclercq et al., 2017). Conversely, reduced *LIPA* expression in the jejunum of the pHu+ line may result in the accumulation of undegraded lipids, impairing intestinal absorption. This aligns with the lower nutrient intake digestive efficiency previously reported in this line (Berger et al., 2022; Bochereau et al., 2024).

The other three genes are involved in tissue development and morphogenesis. *TCERG1L*, which was under expressed in the jejunum but overexpressed in the muscle of the pHu+ line, plays a role in transcription (Yang et al., 2024). Its overexpression in muscle may be linked to cell proliferation and muscle protein synthesis, processes crucial for the rapid muscle growth and tissue renewal observed in the pHu+ line compared to the pHu- line (Alnahhas et al., 2014; Beauclercq et al., 2017). In contrast, the under expression of *TCERG1L* in the jejunum may reflect lower transcriptional regulatory demands, consistent with the reduced development of the digestive tract seen in this line (Bochereau et al., 2024). Finally, *NIF3L1* and *MEF2B*, both of which were overexpressed in the jejunum and muscle of the pHu+ line, are involved in neuronal differentiation (Akiyama et al., 2003) and myocyte proliferation (Chen et al., 2018), respectively. These results support the findings of the WGCNA analysis, which highlights the central role of neural activity in the gut-muscle communication that contributes to the trait divergence between the two lines.

### Similarities between transcriptional regulations involved in muscle glycogen depletion and wooden breast defect

A recent study re-examined the RNA-Seq data using the latest software and reference genome, as in the present study, to determine the initial causes of WB and identify novel genes that may be involved in its occurrence (Bordini et al., 2024). They identified several genes and functions, which helped us to discuss them in relation to results of the present study, since WB has been associated with lower glycogen reserves in breast muscle (Abasht et al., 2019), as in the pHu+ line (Alnahhas et al., 2014).

*WFIKKN1* was among the 10 up-regulated DE genes in WB-afflicted muscles in their study and was also overexpressed in the pHu+ line in the present study. *WFIKKN1* encodes a multidomain extracellular protein (GASP) and decreases the activity of semi-latent myostatin, a muscle-growth inhibitor (Szláma et al., 2016), thereby promoting muscle growth (Wang et al., 2015), which is higher in WB-afflicted muscles (compared to normal muscle), as in the pHu+ line (compared to the pHu- line).

**Fig. 3:**
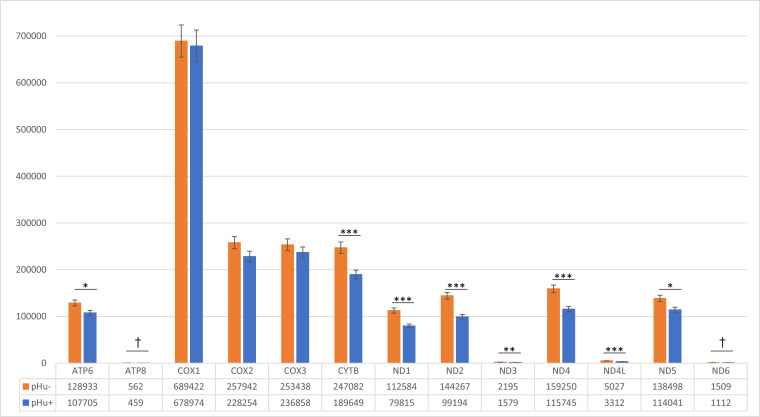
Expression of the 13 genes encoded by the mitochondrial genome in the *Pectoralis major* muscle of pHu- and pHu+ lines of chickens. Significance levels: *** *P* ≤ 0.001, ** *P* ≤ 0.01, * *P* ≤ 0.05, and † *P* ≤ 0.10.

Their study also indicated that WB is associated with mitochondrial dysfunction, with down-regulation of genes such as *PPARGC1A* and *PPARGC1B*, which are two transcriptional coactivators of the PGC1s family whose main function is to stimulate the number of mitochondria and oxidative metabolism. The pathways for AMP-activated protein kinase (AMPK) and calcium/calmodulin-dependent protein kinase (CAMK), which promote the autophagy of damaged mitochondria and mitochondrial biogenesis (Herzig and Shaw, 2018), are also enriched in WB-afflicted muscles. Like in their study, *PPARGC1A* and *PPARGC1B* were down-regulated in the pHu+ line, while *CAMK1D* was up-regulated, which likely limits the mitochondrial oxidative phosphorylation (OXPHOS) system, which produces most of the ATP in cells (Hubert and Athrey, 2022). Of the 13 mitochondrial genes that encode OXPHOS subunits, 8 (*ND1, ND2, ND3, ND4, ND4L, ND5, ATP6*, and *CYTB*) were underexpressed in the pHu+ line compared to pHu- line (Fig. 3).

Several additional genes involved in carbohydrate and glycogen metabolism (e.g., *PHKA2, GYG2, GFPT2*, and *PGM3* were overexpressed in the pHu+ line, while *PHKB, IDH3A, SUCLG1, LDHA, PGK2, UGP2*, and *CBS* was overexpressed in the pHu- line) were also regulated in a similar way in WB-afflicted muscles and in the pHu+ line. The same held true for genes associated with amino acid metabolism (*PRODH, DDAH1*, and *ENO2* were overexpressed in the pHu+ line, while *GLS2, SLC25A12, HIBADH* were overexpressed in the pHu- line) and fatty acid metabolism (*ALDOC* overexpressed in the pHu+ line) (Abasht et al., 2019). These similarities in the regulation of the expression of genes involved in energy production between WB-afflicted muscles and those in the pHu+ line support the hypothesis that energy reserves in muscle influence the occurrence of WB. Interestingly, the gene *ENSGALG00010018616*, when blasted with a sequence from the avian leukosis virus subgroup E (ALVE), which was by far the most down-regulated gene in the pHu+ line (72 times less expressed), was also the most down-regulated gene in WB-afflicted muscles (Bordini et al., 2024). This observation in chickens from two different genetic backgrounds suggests that this gene could be a useful marker to distinguish healthy muscles from those that lack glycogen reserves, including WB-afflicted muscle.

## Conclusion

This comprehensive analysis of the muscle transcriptome profile of two chicken lines, after 15 generations of selection based on breast meat ultimate pH (a marker of muscle glycogen reserves), confirmed the importance of genes involved in muscle physiology and organization that had been identified 9 generations earlier. It also revealed a broadening of the metabolic pathways involved in energy metabolism, as well as in muscle development and reconstruction, accompanied by a worsening of the deregulations observed, such as the marked dysfunction of mitochondrial metabolism in glycogen-depleted muscle (pHu+ line), similar to that observed in WB-afflicted muscle. Molecular interactions between the gut and muscle were also observed, which supported a relation between nerve-signal transmission, tissue oxygenation, immune response, and glycogen reserves in the breast muscle. This highlights the importance of using a holistic approach that considers dialogue between tissues to better understand the biological factors that influence muscle function and health and targets the most appropriate strategies to maintain them, whether genetic or related to rearing practices.

## Data availability

The RNA-Seq datasets generated and analyzed in this study are available from the United States National Center for Biotechnology Information: https://dataview.ncbi.nlm.nih.gov/object/PRJNA1205522.

## Declaration of competing interest

The authors declare that they have no known competing financial interests or personal relationships that could have appeared to influence the work reported in this paper.

## Appendix Supplementary materials

Image, application 1
