# Supplementary material for: RNA-Seq data provide new insights into the molecular regulation of breast muscle glycogen reserves, a key factor in muscle function and meat quality in chickens
Source: Poult Sci. 2025 Apr 4;104(6):105136. doi: 10.1016/j.psj.2025.105136 (PMC12018111; doi:10.1016/j.psj.2025.105136)
Supplement: Supplementary file 1 [file mmc1.docx]

**Additional Table 1.** The 2676 differentially expressed (DE) genes in the *Pectoralis major* muscle between the pHu- and pHu+ lines of chickens (N = 15 per line) ordered by their Ensembl gene ID. Base mean = mean expression of the gene. FC = fold change. Genes in italics are those DE in the *Pectoralis major* and jejunum of the same animals.

| **ENSEMBL gene ID** | **Symbol** | **Base mean** | **Mean expression in the pHu- line** | **Mean expression in the pHu+ line** | **FC**  **(pHu-/pHu+)** | **Adjusted P-value** |
| --- | --- | --- | --- | --- | --- | --- |
| ENSGALG00010000007 | ND1 | 96,200 | 112,584 | 79,815 | 1.411 | 4.90E-05 |
| ENSGALG00010000011 | ND2 | 121,730 | 144,267 | 99,194 | 1.454 | 3.43E-04 |
| ENSGALG00010000023 | ATP6 | 118,319 | 128,933 | 107,705 | 1.197 | 3.14E-02 |
| ENSGALG00010000026 | ND3 | 1,887 | 2,195 | 1,579 | 1.39 | 1.00E-02 |
| ENSGALG00010000028 | ND4L | 4,170 | 5,027 | 3,312 | 1.518 | 3.80E-03 |
| ENSGALG00010000029 | ND4 | 137,498 | 159,250 | 115,745 | 1.376 | 1.62E-04 |
| ENSGALG00010000033 | ND5 | 126,270 | 138,498 | 114,041 | 1.214 | 3.28E-02 |
| ENSGALG00010000034 | CYTB | 218,365 | 247,082 | 189,649 | 1.303 | 7.39E-04 |
| ENSGALG00010000039 | PDIA3 | 3,630 | 3,321 | 3,939 | 0.843 | 1.21E-02 |
| ENSGALG00010000044 | MAP1A | 8,113 | 6,846 | 9,379 | 0.73 | 1.32E-02 |
| ENSGALG00010000050 | CIB1 | 688 | 567 | 809 | 0.699 | 1.08E-05 |
| ENSGALG00010000070 |  | 4 | 6 | 2 | 3.135 | 4.27E-02 |
| ENSGALG00010000092 | INPPL1 | 984 | 865 | 1,104 | 0.784 | 7.50E-05 |
| ENSGALG00010000108 |  | 19 | 6 | 31 | 0.192 | 1.84E-02 |
| ENSGALG00010000133 | USP39 | 356 | 384 | 328 | 1.173 | 4.53E-02 |
| ENSGALG00010000145 | PRODH | 365 | 220 | 510 | 0.431 | 4.99E-06 |
| ENSGALG00010000173 | NIPSNAP1 | 86 | 76 | 97 | 0.779 | 6.89E-03 |
| ENSGALG00010000197 |  | 23 | 34 | 13 | 2.506 | 5.00E-02 |
| ENSGALG00010000298 | SLC35E4 | 47 | 35 | 59 | 0.597 | 2.59E-03 |
| ENSGALG00010000303 |  | 1,387 | 1,246 | 1,529 | 0.815 | 1.04E-02 |
| ENSGALG00010000337 |  | 41 | 14 | 68 | 0.205 | 1.98E-02 |
| ENSGALG00010000350 |  | 1,356 | 1,467 | 1,246 | 1.178 | 2.74E-02 |
| ENSGALG00010000372 |  | 7 | 10 | 5 | 2.041 | 8.01E-03 |
| *ENSGALG00010000396* |  | *21* | *28* | *14* | *1.994* | *1.04E-02* |
| ENSGALG00010000409 |  | 849 | 1,335 | 363 | 3.68 | 7.14E-13 |
| ENSGALG00010000421 |  | 30 | 38 | 22 | 1.713 | 1.00E-02 |
| ENSGALG00010000441 | SLC7A4 | 14 | 17 | 10 | 1.714 | 1.79E-02 |
| ENSGALG00010000443 | TBX3 | 309 | 341 | 276 | 1.235 | 2.88E-02 |
| ENSGALG00010000449 | PLBD2 | 799 | 662 | 936 | 0.707 | 1.10E-02 |
| ENSGALG00010000475 | RNFT2 | 170 | 195 | 144 | 1.349 | 1.61E-04 |
| ENSGALG00010000478 |  | 7 | 4 | 10 | 0.356 | 1.41E-02 |
| ENSGALG00010000498 |  | 7 | 5 | 9 | 0.583 | 3.40E-02 |
| ENSGALG00010000588 |  | 35,903 | 39,889 | 31,916 | 1.25 | 9.28E-03 |
| ENSGALG00010000589 |  | 223 | 340 | 105 | 3.249 | 4.88E-03 |
| ENSGALG00010000599 | RETREG1 | 4,849 | 4,106 | 5,591 | 0.734 | 4.81E-03 |
| *ENSGALG00010000604* |  | *66* | *86* | *46* | *1.833* | *1.79E-06* |
| ENSGALG00010000605 | MARCHF11 | 5 | 6 | 3 | 2.185 | 4.58E-02 |
| ENSGALG00010000639 | SCN1B | 2,026 | 2,282 | 1,770 | 1.29 | 5.70E-03 |
| ENSGALG00010000652 | SRD5A1 | 133 | 106 | 160 | 0.665 | 7.49E-03 |
| ENSGALG00010000684 | COBL | 2,484 | 2,117 | 2,851 | 0.742 | 2.25E-02 |
| ENSGALG00010000703 |  | 17 | 22 | 12 | 1.783 | 3.52E-02 |
| ENSGALG00010000757 | SCEL | 36 | 11 | 61 | 0.176 | 1.97E-09 |
| ENSGALG00010000783 | DAP | 1,375 | 1,277 | 1,474 | 0.865 | 3.82E-02 |
| ENSGALG00010000800 | LMO7 | 3,992 | 3,551 | 4,433 | 0.801 | 8.65E-04 |
| ENSGALG00010000803 | MTRR | 624 | 705 | 544 | 1.297 | 3.66E-03 |
| ENSGALG00010000810 | UCHL3 | 1,625 | 1,436 | 1,814 | 0.792 | 1.50E-02 |
| ENSGALG00010000820 | KLF5 | 626 | 458 | 793 | 0.577 | 1.26E-04 |
| ENSGALG00010000853 |  | 263 | 181 | 346 | 0.522 | 3.84E-04 |
| ENSGALG00010001053 |  | 5 | 7 | 3 | 2.034 | 3.78E-02 |
| ENSGALG00010001111 | SUCNR1 | 84 | 66 | 103 | 0.645 | 1.01E-02 |
| ENSGALG00010001112 | TSC22D2 | 967 | 1,023 | 911 | 1.123 | 4.96E-02 |
| ENSGALG00010001115 | PFN2 | 5,567 | 4,885 | 6,249 | 0.782 | 6.60E-06 |
| ENSGALG00010001121 | WWTR1 | 3,341 | 2,988 | 3,694 | 0.809 | 9.40E-04 |
| ENSGALG00010001126 | MINDY4B | 23 | 45 | 1 | 42.404 | 5.76E-05 |
| ENSGALG00010001141 | MTAP | 431 | 471 | 391 | 1.205 | 2.13E-02 |
| ENSGALG00010001143 | CDKN2A | 44 | 54 | 35 | 1.542 | 3.93E-02 |
| ENSGALG00010001152 | FEM1C | 4,658 | 5,198 | 4,118 | 1.262 | 4.09E-02 |
| ENSGALG00010001154 | PTGR3 | 595 | 644 | 546 | 1.181 | 1.64E-02 |
| ENSGALG00010001159 | RTTN | 121 | 135 | 108 | 1.243 | 2.70E-02 |
| ENSGALG00010001167 | SMC4 | 595 | 642 | 548 | 1.168 | 1.45E-03 |
| ENSGALG00010001170 | DCAF10 | 1,217 | 1,310 | 1,124 | 1.165 | 4.93E-02 |
| ENSGALG00010001174 |  | 109 | 123 | 94 | 1.294 | 4.56E-03 |
| ENSGALG00010001187 | GFM1 | 2,386 | 2,663 | 2,109 | 1.264 | 4.15E-02 |
| ENSGALG00010001191 | TOMM5 | 1,818 | 1,494 | 2,142 | 0.698 | 5.99E-03 |
| ENSGALG00010001200 | CTTNBP2 | 2,847 | 3,223 | 2,471 | 1.304 | 2.35E-03 |
| ENSGALG00010001204 | SNX2 | 2,315 | 2,710 | 1,920 | 1.412 | 3.63E-10 |
| ENSGALG00010001213 | PPIC | 416 | 366 | 467 | 0.784 | 1.02E-02 |
| ENSGALG00010001214 | TES | 454 | 512 | 396 | 1.29 | 9.91E-03 |
| ENSGALG00010001220 | PLXNB2 | 2,345 | 2,057 | 2,633 | 0.781 | 4.35E-02 |
| ENSGALG00010001230 | PTX3 | 86 | 43 | 128 | 0.336 | 4.66E-03 |
| ENSGALG00010001234 | AASS | 2,643 | 3,082 | 2,205 | 1.398 | 1.99E-04 |
| ENSGALG00010001242 |  | 18 | 5 | 31 | 0.164 | 2.16E-06 |
| ENSGALG00010001245 | SERPINI1 | 330 | 208 | 451 | 0.463 | 3.37E-05 |
| ENSGALG00010001254 | NETO1 | 19 | 25 | 13 | 1.974 | 2.35E-02 |
| ENSGALG00010001265 | OPN1LW | 27 | 14 | 40 | 0.351 | 1.25E-04 |
| ENSGALG00010001267 | BCHE | 1,054 | 698 | 1,409 | 0.495 | 1.02E-04 |
| ENSGALG00010001297 | MYL12A | 5,742 | 4,845 | 6,638 | 0.73 | 1.25E-02 |
| ENSGALG00010001306 | CEP120 | 568 | 602 | 534 | 1.129 | 3.09E-02 |
| ENSGALG00010001308 | MGMT | 127 | 108 | 147 | 0.734 | 5.81E-04 |
| ENSGALG00010001320 | CPED1 | 92 | 78 | 105 | 0.745 | 7.36E-03 |
| ENSGALG00010001328 |  | 154 | 187 | 122 | 1.532 | 1.11E-04 |
| ENSGALG00010001330 |  | 227 | 189 | 265 | 0.712 | 2.86E-02 |
| ENSGALG00010001343 |  | 5 | 4 | 8 | 0.454 | 3.70E-03 |
| *ENSGALG00010001358* | *TCERG1L* | *6* | *9* | *4* | *2.723* | *1.80E-02* |
| ENSGALG00010001363 | HIBADH | 2,522 | 3,122 | 1,921 | 1.626 | 9.85E-06 |
| ENSGALG00010001368 | WIPF3 | 2,300 | 2,590 | 2,012 | 1.288 | 8.97E-04 |
| ENSGALG00010001385 | MME | 661 | 789 | 532 | 1.483 | 6.90E-03 |
| ENSGALG00010001390 |  | 61 | 79 | 44 | 1.803 | 2.53E-04 |
| ENSGALG00010001407 | FOXP2 | 99 | 110 | 87 | 1.261 | 3.60E-02 |
| ENSGALG00010001411 | FGFR2 | 116 | 158 | 75 | 2.105 | 4.89E-02 |
| ENSGALG00010001423 | FAM210A | 2,611 | 2,913 | 2,309 | 1.262 | 1.35E-03 |
| ENSGALG00010001480 | JAKMIP3 | 3 | 1 | 5 | 0.23 | 5.79E-03 |
| ENSGALG00010001488 | GRPR | 12 | 9 | 15 | 0.595 | 4.56E-03 |
| ENSGALG00010001496 | PTP4A1 | 14,379 | 15,988 | 12,770 | 1.252 | 2.22E-03 |
| ENSGALG00010001505 | SSR3 | 2,109 | 1,947 | 2,271 | 0.857 | 2.16E-02 |
| ENSGALG00010001508 | CD109 | 79 | 46 | 113 | 0.408 | 7.32E-07 |
| ENSGALG00010001512 | PLCL2 | 1,875 | 2,070 | 1,681 | 1.232 | 4.46E-02 |
| ENSGALG00010001514 | MET | 232 | 194 | 269 | 0.721 | 4.92E-02 |
| ENSGALG00010001517 | CAV2 | 756 | 853 | 660 | 1.294 | 2.75E-04 |
| ENSGALG00010001525 | EEF1A1 | 35,632 | 30,742 | 40,522 | 0.759 | 1.48E-02 |
| ENSGALG00010001526 | PPP1R3A | 21,185 | 25,163 | 17,208 | 1.462 | 1.21E-06 |
| ENSGALG00010001531 |  | 1 | 3 | 0 | 16.624 | 3.54E-02 |
| ENSGALG00010001535 | RAP2B | 312 | 281 | 343 | 0.816 | 2.26E-03 |
| ENSGALG00010001545 | SELENOO | 270 | 305 | 236 | 1.298 | 2.66E-02 |
| ENSGALG00010001548 | AKAP17A | 821 | 896 | 746 | 1.203 | 3.59E-05 |
| ENSGALG00010001570 | SMPD1 | 272 | 223 | 322 | 0.693 | 1.08E-02 |
| ENSGALG00010001586 | ZBTB14 | 353 | 383 | 323 | 1.188 | 1.99E-03 |
| ENSGALG00010001595 | AKAIN1 | 34 | 46 | 22 | 2.104 | 8.64E-08 |
| ENSGALG00010001612 | HPX | 132 | 80 | 184 | 0.434 | 4.24E-02 |
| ENSGALG00010001621 | DLGAP2 | 62 | 41 | 82 | 0.493 | 4.86E-03 |
| ENSGALG00010001628 | MAPK11 | 63 | 49 | 77 | 0.637 | 2.90E-02 |
| ENSGALG00010001640 |  | 310 | 268 | 352 | 0.762 | 1.11E-02 |
| ENSGALG00010001675 |  | 31 | 19 | 43 | 0.432 | 9.85E-05 |
| ENSGALG00010001695 | LMOD2 | 299 | 182 | 415 | 0.439 | 8.71E-03 |
| ENSGALG00010001699 | ASB15 | 2,733 | 3,134 | 2,332 | 1.344 | 3.03E-02 |
| ENSGALG00010001706 | IQUB | 77 | 91 | 63 | 1.47 | 2.04E-02 |
| ENSGALG00010001713 | PNPLA4 | 158 | 178 | 138 | 1.295 | 2.88E-02 |
| ENSGALG00010001773 | NRCAM | 40 | 23 | 57 | 0.397 | 2.32E-04 |
| ENSGALG00010001776 | LMBRD1 | 1,515 | 1,320 | 1,711 | 0.772 | 7.42E-05 |
| ENSGALG00010001780 |  | 142 | 167 | 116 | 1.437 | 2.47E-02 |
| ENSGALG00010001788 | HOXA1 | 24 | 13 | 34 | 0.368 | 5.41E-06 |
| ENSGALG00010001803 | PPP4R1 | 668 | 604 | 731 | 0.826 | 1.43E-03 |
| ENSGALG00010001807 | ASMTL | 531 | 572 | 489 | 1.172 | 4.91E-02 |
| ENSGALG00010001831 | CSF2RA | 176 | 139 | 213 | 0.653 | 2.34E-02 |
| ENSGALG00010001845 | ANKRD12 | 701 | 744 | 657 | 1.132 | 3.24E-02 |
| ENSGALG00010001848 | HOXA11 | 126 | 145 | 107 | 1.362 | 8.50E-03 |
| ENSGALG00010001861 | CBX3 | 3,976 | 3,775 | 4,176 | 0.904 | 4.33E-02 |
| ENSGALG00010001873 |  | 35 | 44 | 25 | 1.778 | 4.07E-03 |
| ENSGALG00010001878 | XK | 11 | 14 | 7 | 1.762 | 1.98E-02 |
| ENSGALG00010001879 | IRAK4 | 211 | 197 | 225 | 0.872 | 1.68E-02 |
| ENSGALG00010001885 | SMPX | 3,056 | 1,624 | 4,487 | 0.362 | 2.53E-06 |
| ENSGALG00010001886 | NEU3 | 746 | 676 | 816 | 0.828 | 2.27E-02 |
| ENSGALG00010001888 | SNX10 | 568 | 528 | 607 | 0.869 | 3.27E-02 |
| ENSGALG00010001890 | KLHL34 | 8,529 | 7,288 | 9,769 | 0.746 | 1.69E-02 |
| ENSGALG00010001901 | PRPS2 | 724 | 826 | 622 | 1.329 | 4.82E-06 |
| ENSGALG00010001902 | WDR45 | 282 | 257 | 306 | 0.843 | 4.48E-02 |
| ENSGALG00010001923 | RAPGEF5 | 718 | 828 | 607 | 1.365 | 1.32E-05 |
| ENSGALG00010001928 | KCTD21 | 209 | 162 | 256 | 0.634 | 1.08E-05 |
| ENSGALG00010002005 | COLQ | 194 | 123 | 264 | 0.466 | 2.87E-04 |
| ENSGALG00010002024 | KLHL31 | 74,627 | 85,520 | 63,734 | 1.342 | 1.08E-04 |
| *ENSGALG00010002033* |  | *8* | *3* | *14* | *0.169* | *8.10E-05* |
| ENSGALG00010002062 | GSTA4 | 50 | 58 | 41 | 1.442 | 4.40E-02 |
| ENSGALG00010002070 |  | 2 | 0 | 4 | 0.118 | 1.27E-03 |
| ENSGALG00010002088 | OZFL | 98 | 117 | 79 | 1.49 | 2.23E-02 |
| ENSGALG00010002092 |  | 6 | 3 | 9 | 0.314 | 6.98E-03 |
| ENSGALG00010002100 | BTD | 195 | 175 | 215 | 0.814 | 2.45E-02 |
| ENSGALG00010002102 | PAK1 | 306 | 247 | 365 | 0.674 | 2.16E-04 |
| ENSGALG00010002135 | ELOVL5 | 972 | 1,076 | 869 | 1.235 | 1.97E-03 |
| ENSGALG00010002137 | PDK3 | 6,374 | 7,401 | 5,347 | 1.385 | 1.44E-06 |
| ENSGALG00010002148 | POLR2A | 1,302 | 1,461 | 1,143 | 1.279 | 1.56E-02 |
| ENSGALG00010002150 | EIF3A | 7,258 | 6,683 | 7,833 | 0.853 | 5.04E-03 |
| ENSGALG00010002154 |  | 25 | 21 | 30 | 0.678 | 1.82E-02 |
| ENSGALG00010002196 | FAM45A | 466 | 434 | 498 | 0.87 | 8.01E-03 |
| ENSGALG00010002237 |  | 12 | 16 | 8 | 1.892 | 2.48E-02 |
| ENSGALG00010002270 | IMMP2L | 81 | 67 | 95 | 0.711 | 6.14E-03 |
| ENSGALG00010002285 | CAV1 | 3,897 | 4,458 | 3,336 | 1.336 | 1.19E-09 |
| ENSGALG00010002293 | GYG2 | 30 | 26 | 35 | 0.73 | 2.87E-02 |
| ENSGALG00010002294 | DAZL | 8 | 1 | 15 | 0.081 | 2.83E-02 |
| ENSGALG00010002308 | GXYLT1 | 411 | 375 | 447 | 0.839 | 4.47E-02 |
| ENSGALG00010002309 | GADL1 | 24,281 | 28,425 | 20,137 | 1.412 | 2.88E-02 |
| ENSGALG00010002312 |  | 167 | 189 | 144 | 1.307 | 1.19E-03 |
| ENSGALG00010002332 |  | 23 | 16 | 29 | 0.548 | 2.85E-03 |
| *ENSGALG00010002333* |  | *44* | *67* | *21* | *3.106* | *2.92E-04* |
| ENSGALG00010002338 | CD99 | 8,800 | 6,806 | 10,794 | 0.63 | 6.08E-10 |
| ENSGALG00010002340 | RAB23 | 216 | 195 | 236 | 0.823 | 1.83E-02 |
| ENSGALG00010002341 | FAM49A | 1,222 | 989 | 1,456 | 0.679 | 4.32E-05 |
| ENSGALG00010002342 | BAG2 | 6,508 | 5,739 | 7,277 | 0.789 | 3.55E-02 |
| ENSGALG00010002355 | UFM1 | 1,200 | 1,016 | 1,385 | 0.734 | 5.04E-06 |
| ENSGALG00010002357 | RAB31 | 285 | 222 | 349 | 0.634 | 2.38E-05 |
| ENSGALG00010002358 | FREM2 | 645 | 540 | 749 | 0.721 | 3.98E-02 |
| ENSGALG00010002372 | SMC6 | 459 | 497 | 420 | 1.187 | 8.40E-04 |
| ENSGALG00010002375 | NPY | 13 | 8 | 17 | 0.5 | 1.40E-02 |
| ENSGALG00010002377 | ART7B | 626 | 709 | 543 | 1.305 | 1.29E-02 |
| ENSGALG00010002389 | COL21A1 | 407 | 336 | 477 | 0.703 | 1.63E-02 |
| ENSGALG00010002391 | REPS2 | 252 | 273 | 232 | 1.172 | 4.98E-02 |
| ENSGALG00010002392 | DFNA5 | 225 | 190 | 261 | 0.726 | 1.80E-02 |
| ENSGALG00010002402 | TXLNG | 990 | 1,148 | 832 | 1.383 | 3.98E-05 |
| ENSGALG00010002414 |  | 117 | 61 | 173 | 0.352 | 1.59E-02 |
| ENSGALG00010002415 | PCDH17 | 3 | 2 | 4 | 0.351 | 3.82E-02 |
| ENSGALG00010002427 | CYB5R4 | 370 | 346 | 394 | 0.88 | 3.77E-02 |
| ENSGALG00010002428 | IBSP | 187 | 105 | 269 | 0.39 | 3.15E-07 |
| ENSGALG00010002431 | BCKDHB | 1,509 | 1,698 | 1,320 | 1.288 | 2.09E-02 |
| ENSGALG00010002434 | GPR34 | 297 | 245 | 348 | 0.703 | 1.04E-02 |
| ENSGALG00010002441 | USO1 | 2,616 | 2,474 | 2,757 | 0.897 | 2.49E-03 |
| ENSGALG00010002459 | G3BP2 | 1,548 | 1,463 | 1,633 | 0.896 | 5.43E-03 |
| ENSGALG00010002467 | NEK3 | 24 | 20 | 27 | 0.732 | 2.79E-02 |
| ENSGALG00010002478 | PPME1 | 992 | 915 | 1,068 | 0.856 | 3.35E-03 |
| ENSGALG00010002481 | CKAP2 | 378 | 418 | 337 | 1.235 | 2.13E-02 |
| ENSGALG00010002547 | PBX4 | 85 | 92 | 78 | 1.18 | 4.30E-02 |
| ENSGALG00010002557 |  | 137 | 193 | 82 | 2.357 | 1.03E-02 |
| ENSGALG00010002563 |  | 3 | 1 | 4 | 0.34 | 2.70E-02 |
| ENSGALG00010002569 | GTPBP6 | 164 | 190 | 138 | 1.384 | 1.99E-02 |
| ENSGALG00010002573 | MVB12A | 425 | 359 | 490 | 0.733 | 1.91E-03 |
| ENSGALG00010002579 | MATN3 | 69 | 50 | 89 | 0.567 | 3.15E-03 |
| ENSGALG00010002590 | ATP6V1C2 | 92 | 66 | 118 | 0.56 | 2.57E-05 |
| ENSGALG00010002598 | NMT2 | 740 | 667 | 814 | 0.818 | 3.84E-04 |
| ENSGALG00010002601 | PLCXD1 | 210 | 174 | 246 | 0.708 | 3.83E-02 |
| ENSGALG00010002609 | PDIA6 | 3,932 | 3,543 | 4,320 | 0.82 | 4.23E-06 |
| ENSGALG00010002614 | GPAT3 | 1,001 | 864 | 1,139 | 0.758 | 3.84E-04 |
| ENSGALG00010002636 |  | 5 | 7 | 3 | 2.496 | 4.16E-02 |
| ENSGALG00010002681 | PLEKHB1 | 142 | 161 | 123 | 1.297 | 3.36E-03 |
| ENSGALG00010002685 | CA8 | 311 | 348 | 274 | 1.272 | 4.08E-02 |
| ENSGALG00010002688 |  | 66 | 100 | 31 | 3.155 | 3.43E-04 |
| ENSGALG00010002707 | RELT | 16 | 11 | 21 | 0.55 | 6.45E-03 |
| ENSGALG00010002718 |  | 6 | 3 | 9 | 0.402 | 3.21E-02 |
| ENSGALG00010002725 | TSPAN7 | 4,502 | 3,569 | 5,435 | 0.657 | 3.77E-06 |
| ENSGALG00010002728 | RP2 | 729 | 623 | 835 | 0.746 | 6.89E-03 |
| ENSGALG00010002741 | PDK4 | 282 | 126 | 437 | 0.289 | 2.65E-05 |
| ENSGALG00010002742 | PROSER1 | 484 | 544 | 425 | 1.277 | 1.63E-04 |
| ENSGALG00010002750 |  | 608 | 696 | 521 | 1.333 | 3.18E-03 |
| ENSGALG00010002760 | RAB2A | 4,805 | 4,478 | 5,131 | 0.873 | 3.35E-02 |
| ENSGALG00010002765 | MAP7D2 | 40 | 26 | 53 | 0.483 | 2.81E-02 |
| ENSGALG00010002774 | TBX18 | 1,602 | 2,050 | 1,154 | 1.777 | 8.04E-05 |
| ENSGALG00010002792 |  | 580 | 381 | 779 | 0.489 | 1.33E-12 |
| ENSGALG00010002809 | SH3KBP1 | 1,244 | 1,499 | 989 | 1.515 | 1.21E-05 |
| ENSGALG00010002814 | TERF1 | 157 | 174 | 140 | 1.235 | 3.14E-02 |
| ENSGALG00010002827 | SBSPON | 16 | 11 | 22 | 0.475 | 2.39E-02 |
| ENSGALG00010002866 |  | 1,302 | 1,455 | 1,149 | 1.266 | 3.25E-02 |
| ENSGALG00010002868 | PPP1R9A | 1,059 | 943 | 1,174 | 0.803 | 4.21E-02 |
| ENSGALG00010002871 |  | 5,305 | 6,016 | 4,594 | 1.31 | 6.08E-03 |
| ENSGALG00010002878 | P2RY2 | 3,162 | 3,656 | 2,669 | 1.37 | 3.34E-02 |
| ENSGALG00010002880 | RASGEF1B | 231 | 185 | 278 | 0.664 | 7.22E-03 |
| ENSGALG00010002881 |  | 825 | 955 | 694 | 1.378 | 2.19E-03 |
| ENSGALG00010002886 | HERC2 | 4,414 | 4,812 | 4,016 | 1.198 | 5.00E-02 |
| ENSGALG00010002912 | RPS3 | 10,902 | 9,770 | 12,035 | 0.812 | 8.63E-03 |
| ENSGALG00010002916 | SDCBP | 3,405 | 2,839 | 3,970 | 0.715 | 9.85E-04 |
| ENSGALG00010002927 | GABRA5 | 10 | 4 | 16 | 0.277 | 7.45E-04 |
| ENSGALG00010002946 | GDPD5 | 156 | 127 | 186 | 0.681 | 7.42E-04 |
| ENSGALG00010002960 | BLEC2 | 18 | 5 | 31 | 0.16 | 1.57E-06 |
| ENSGALG00010002965 | PRSS35 | 2,620 | 2,767 | 2,473 | 1.118 | 4.51E-02 |
| ENSGALG00010002972 | ME1 | 3,368 | 3,830 | 2,905 | 1.319 | 1.10E-03 |
| *ENSGALG00010002991* | *BLB1* | *539* | *767* | *310* | *2.472* | *2.14E-06* |
| ENSGALG00010003002 | PHKA2 | 226 | 145 | 306 | 0.475 | 7.01E-07 |
| ENSGALG00010003017 | PGM3 | 490 | 397 | 584 | 0.678 | 3.99E-04 |
| *ENSGALG00010003022* | *BLB2* | *850* | *405* | *1,295* | *0.313* | *1.38E-09* |
| ENSGALG00010003035 | DGAT2 | 711 | 812 | 610 | 1.331 | 3.71E-02 |
| ENSGALG00010003052 | ZFX | 1,493 | 1,602 | 1,384 | 1.158 | 1.42E-03 |
| ENSGALG00010003057 | DDX1 | 9,207 | 10,164 | 8,250 | 1.232 | 9.92E-03 |
| ENSGALG00010003074 | TTPA | 48 | 64 | 32 | 2.001 | 5.42E-03 |
| ENSGALG00010003101 | KLHL15 | 420 | 477 | 362 | 1.315 | 1.05E-02 |
| ENSGALG00010003115 | UBE3D | 81 | 93 | 68 | 1.382 | 3.26E-02 |
| ENSGALG00010003133 | SAT1 | 1,623 | 1,383 | 1,864 | 0.742 | 9.28E-03 |
| ENSGALG00010003159 | TAP2 | 394 | 308 | 480 | 0.642 | 3.85E-03 |
| ENSGALG00010003170 | C4 | 93 | 74 | 112 | 0.656 | 5.97E-03 |
| ENSGALG00010003176 | PRAG1 | 48 | 55 | 42 | 1.301 | 4.57E-02 |
| ENSGALG00010003192 | RGCC | 2,036 | 922 | 3,150 | 0.293 | 1.99E-06 |
| ENSGALG00010003193 | ACOT9 | 4,510 | 3,762 | 5,259 | 0.715 | 7.35E-08 |
| ENSGALG00010003218 | MAP3K7 | 7,032 | 6,554 | 7,510 | 0.873 | 8.36E-03 |
| ENSGALG00010003220 | SPP1 | 1,084 | 595 | 1,573 | 0.378 | 4.41E-02 |
| ENSGALG00010003222 | ITGA8 | 446 | 351 | 541 | 0.649 | 1.01E-02 |
| ENSGALG00010003239 | RRM1 | 990 | 1,050 | 929 | 1.13 | 8.44E-03 |
| ENSGALG00010003245 |  | 32 | 19 | 46 | 0.407 | 1.88E-06 |
| ENSGALG00010003250 | CD1C | 8 | 4 | 11 | 0.326 | 3.13E-04 |
| ENSGALG00010003255 | CALCR | 27 | 33 | 21 | 1.584 | 3.17E-03 |
| ENSGALG00010003261 | PEX2 | 651 | 708 | 595 | 1.188 | 1.56E-03 |
| ENSGALG00010003280 |  | 799 | 846 | 753 | 1.123 | 2.52E-02 |
| ENSGALG00010003290 |  | 39 | 46 | 32 | 1.431 | 3.66E-02 |
| ENSGALG00010003305 | CALR | 4,444 | 3,916 | 4,972 | 0.788 | 2.75E-03 |
| ENSGALG00010003347 | GPM6A | 46 | 30 | 63 | 0.473 | 2.51E-04 |
| ENSGALG00010003358 | DNAJC15 | 706 | 776 | 635 | 1.222 | 3.55E-02 |
| ENSGALG00010003393 |  | 3 | 5 | 1 | 3.396 | 2.19E-02 |
| ENSGALG00010003394 |  | 69 | 47 | 92 | 0.514 | 4.76E-05 |
| ENSGALG00010003397 | SPATA4 | 10 | 6 | 13 | 0.474 | 2.87E-02 |
| ENSGALG00010003405 | GABRR2 | 13 | 17 | 8 | 2.003 | 3.02E-04 |
| ENSGALG00010003412 | ASB5 | 1,603 | 1,367 | 1,840 | 0.743 | 4.75E-03 |
| ENSGALG00010003431 | PM20D2 | 3,683 | 2,456 | 4,911 | 0.5 | 5.52E-07 |
| ENSGALG00010003437 | PNRC1 | 3,369 | 3,051 | 3,687 | 0.828 | 2.55E-02 |
| ENSGALG00010003439 | GMIP | 125 | 97 | 152 | 0.637 | 4.24E-02 |
| ENSGALG00010003455 |  | 97 | 81 | 112 | 0.722 | 4.74E-02 |
| ENSGALG00010003469 | ZPAX | 3 | 4 | 1 | 4.985 | 6.76E-03 |
| ENSGALG00010003474 | RPS7 | 10,437 | 9,588 | 11,286 | 0.85 | 1.78E-02 |
| ENSGALG00010003485 | RNGTT | 628 | 666 | 590 | 1.13 | 2.31E-02 |
| ENSGALG00010003489 | SGCE | 661 | 543 | 780 | 0.696 | 2.76E-04 |
| ENSGALG00010003528 | GDAP1 | 43 | 34 | 53 | 0.639 | 3.22E-02 |
| ENSGALG00010003536 |  | 2 | 3 | 1 | 6.153 | 1.04E-02 |
| ENSGALG00010003543 | SOX11 | 382 | 330 | 433 | 0.76 | 1.07E-02 |
| ENSGALG00010003597 | RNF144A | 94 | 108 | 80 | 1.335 | 1.52E-02 |
| ENSGALG00010003652 | MYCN | 139 | 157 | 122 | 1.293 | 1.02E-02 |
| ENSGALG00010003665 | RDH14 | 1,174 | 1,331 | 1,018 | 1.308 | 4.57E-02 |
| ENSGALG00010003701 | SARAF | 2,063 | 1,800 | 2,325 | 0.774 | 2.50E-04 |
| ENSGALG00010003714 | ORC3 | 316 | 290 | 341 | 0.851 | 1.80E-02 |
| ENSGALG00010003716 | YLEC8 | 39 | 54 | 24 | 2.294 | 2.23E-02 |
| ENSGALG00010003733 | STAU2 | 3,477 | 3,238 | 3,716 | 0.871 | 7.02E-04 |
| ENSGALG00010003737 | SRP72 | 1,471 | 1,400 | 1,542 | 0.907 | 4.69E-02 |
| ENSGALG00010003746 | HOPX | 1,977 | 1,223 | 2,730 | 0.448 | 8.70E-05 |
| ENSGALG00010003753 |  | 1,093 | 1,293 | 894 | 1.448 | 2.87E-04 |
| ENSGALG00010003768 | ARSJ | 155 | 121 | 188 | 0.641 | 1.10E-02 |
| ENSGALG00010003772 |  | 133 | 188 | 77 | 2.446 | 1.11E-02 |
| ENSGALG00010003814 | TPP1 | 637 | 506 | 769 | 0.658 | 6.71E-03 |
| ENSGALG00010003816 | KLHL8 | 87 | 69 | 105 | 0.66 | 5.47E-05 |
| ENSGALG00010003817 | BF2 | 4,095 | 3,218 | 4,971 | 0.647 | 1.40E-03 |
| ENSGALG00010003821 | RUBCNL | 617 | 526 | 708 | 0.744 | 1.44E-02 |
| ENSGALG00010003823 | PTPN13 | 282 | 242 | 322 | 0.751 | 2.47E-02 |
| ENSGALG00010003826 |  | 711 | 770 | 652 | 1.181 | 9.42E-03 |
| ENSGALG00010003832 | ART7C | 15 | 9 | 22 | 0.407 | 4.76E-03 |
| ENSGALG00010003837 | FAM150B | 36 | 23 | 49 | 0.467 | 8.98E-05 |
| ENSGALG00010003839 |  | 20 | 8 | 31 | 0.266 | 5.68E-03 |
| ENSGALG00010003841 | UBE2W | 556 | 505 | 607 | 0.83 | 1.55E-03 |
| ENSGALG00010003848 | ESD | 1,512 | 1,405 | 1,620 | 0.867 | 9.74E-03 |
| ENSGALG00010003852 | TCEB1 | 2,594 | 2,372 | 2,817 | 0.842 | 2.03E-02 |
| ENSGALG00010003865 | AKAP8L | 1,103 | 1,206 | 1,001 | 1.205 | 1.48E-04 |
| ENSGALG00010003869 | LY96 | 178 | 148 | 208 | 0.708 | 1.23E-02 |
| ENSGALG00010003896 | MCUB | 133 | 115 | 151 | 0.76 | 2.01E-02 |
| ENSGALG00010003909 | DNAJB1 | 615 | 718 | 512 | 1.404 | 3.83E-03 |
| ENSGALG00010003925 | SEC31A | 3,548 | 3,233 | 3,862 | 0.837 | 1.19E-02 |
| ENSGALG00010003941 |  | 55 | 44 | 66 | 0.665 | 4.33E-02 |
| ENSGALG00010003958 | CYSLTR2 | 97 | 108 | 85 | 1.26 | 3.14E-02 |
| ENSGALG00010003960 | ASPH | 14,854 | 13,534 | 16,174 | 0.837 | 9.81E-03 |
| ENSGALG00010003977 | MYO6 | 103 | 116 | 90 | 1.277 | 4.87E-02 |
| ENSGALG00010003982 | GLCCI1 | 378 | 342 | 413 | 0.825 | 1.98E-02 |
| ENSGALG00010004039 | ALPK1 | 130 | 90 | 169 | 0.535 | 9.05E-06 |
| ENSGALG00010004070 | ARL11 | 78 | 62 | 94 | 0.662 | 9.90E-04 |
| ENSGALG00010004079 | AP1AR | 304 | 274 | 334 | 0.818 | 1.27E-03 |
| ENSGALG00010004104 | SCARB2 | 1,667 | 1,444 | 1,891 | 0.763 | 7.89E-07 |
| ENSGALG00010004113 | ECHDC1 | 70 | 80 | 60 | 1.321 | 1.56E-02 |
| ENSGALG00010004122 | TRIM55 | 4,462 | 4,108 | 4,816 | 0.853 | 1.37E-02 |
| ENSGALG00010004133 | CRH | 10 | 2 | 18 | 0.128 | 2.91E-04 |
| ENSGALG00010004143 | TCF24 | 86 | 109 | 62 | 1.753 | 2.28E-02 |
| ENSGALG00010004146 | NAAA | 329 | 292 | 366 | 0.798 | 1.98E-02 |
| ENSGALG00010004149 | SDAD1 | 412 | 441 | 384 | 1.143 | 1.24E-02 |
| ENSGALG00010004173 |  | 4 | 6 | 3 | 2.063 | 4.78E-02 |
| ENSGALG00010004219 | FGF2 | 292 | 268 | 316 | 0.845 | 4.56E-02 |
| ENSGALG00010004222 | SPAG6 | 29 | 23 | 34 | 0.662 | 4.29E-02 |
| ENSGALG00010004257 |  | 5 | 1 | 9 | 0.138 | 3.55E-03 |
| ENSGALG00010004262 |  | 188 | 138 | 239 | 0.578 | 2.80E-02 |
| ENSGALG00010004281 | VIM | 34,344 | 28,339 | 40,348 | 0.702 | 7.85E-04 |
| ENSGALG00010004283 | DCK | 211 | 189 | 233 | 0.809 | 3.43E-03 |
| ENSGALG00010004296 | DSG1 | 2 | 0 | 4 | 0.144 | 4.92E-02 |
| ENSGALG00010004300 | VNN2 | 345 | 280 | 411 | 0.68 | 2.84E-02 |
| ENSGALG00010004302 | CRPPA | 921 | 694 | 1,148 | 0.604 | 1.86E-04 |
| ENSGALG00010004306 | PNISR | 1,508 | 1,650 | 1,367 | 1.206 | 2.97E-03 |
| ENSGALG00010004307 | NPC2 | 773 | 659 | 887 | 0.743 | 3.23E-02 |
| ENSGALG00010004325 | LTBP2 | 2,329 | 2,051 | 2,608 | 0.786 | 9.51E-03 |
| ENSGALG00010004326 |  | 24 | 46 | 3 | 14.751 | 7.01E-03 |
| ENSGALG00010004328 |  | 6 | 10 | 2 | 5.219 | 3.18E-02 |
| ENSGALG00010004330 | STX7 | 2,152 | 1,992 | 2,311 | 0.862 | 2.42E-03 |
| ENSGALG00010004332 |  | 28 | 56 | 1 | 45.327 | 6.61E-03 |
| ENSGALG00010004342 | ZNF438 | 276 | 308 | 244 | 1.263 | 8.24E-04 |
| ENSGALG00010004346 | CEP128 | 35 | 41 | 30 | 1.344 | 2.70E-02 |
| ENSGALG00010004350 | MOXD1 | 1,053 | 860 | 1,247 | 0.69 | 1.11E-04 |
| ENSGALG00010004365 | TPD52 | 905 | 727 | 1,084 | 0.67 | 3.20E-04 |
| ENSGALG00010004372 | SYNJ2 | 355 | 286 | 423 | 0.676 | 2.72E-02 |
| ENSGALG00010004386 | CFAP44 | 23 | 6 | 39 | 0.148 | 5.58E-06 |
| ENSGALG00010004401 |  | 366 | 311 | 421 | 0.741 | 3.58E-02 |
| ENSGALG00010004406 |  | 85 | 94 | 76 | 1.226 | 2.81E-02 |
| ENSGALG00010004485 |  | 6 | 8 | 4 | 1.933 | 2.99E-02 |
| *ENSGALG00010004560* |  | *17* | *9* | *25* | *0.374* | *1.61E-04* |
| ENSGALG00010004692 |  | 705 | 550 | 860 | 0.639 | 1.21E-03 |
| ENSGALG00010004729 |  | 672 | 403 | 941 | 0.429 | 3.88E-04 |
| ENSGALG00010004739 |  | 8 | 4 | 13 | 0.302 | 2.06E-02 |
| ENSGALG00010004754 |  | 2,598 | 3,023 | 2,172 | 1.393 | 2.45E-06 |
| ENSGALG00010004765 |  | 393 | 300 | 487 | 0.614 | 4.06E-03 |
| ENSGALG00010004767 |  | 5 | 8 | 3 | 3.024 | 9.83E-03 |
| ENSGALG00010004803 | RMC1 | 1,097 | 1,242 | 951 | 1.307 | 1.25E-03 |
| ENSGALG00010004814 |  | 334 | 377 | 291 | 1.298 | 2.85E-02 |
| ENSGALG00010004831 |  | 4 | 1 | 7 | 0.211 | 3.10E-04 |
| ENSGALG00010004839 |  | 210 | 184 | 235 | 0.78 | 8.50E-03 |
| ENSGALG00010004852 |  | 927 | 734 | 1,121 | 0.654 | 6.96E-03 |
| ENSGALG00010004901 | CUL5 | 4,720 | 5,355 | 4,085 | 1.311 | 1.25E-02 |
| ENSGALG00010004907 | FRMD1 | 126 | 151 | 102 | 1.484 | 4.51E-03 |
| ENSGALG00010004917 |  | 324 | 284 | 365 | 0.777 | 2.96E-03 |
| ENSGALG00010004935 | SVIL | 12,239 | 10,876 | 13,603 | 0.8 | 4.86E-03 |
| ENSGALG00010004964 |  | 2 | 0 | 3 | 0.149 | 4.85E-02 |
| ENSGALG00010004965 | JCAD | 3,905 | 3,306 | 4,505 | 0.734 | 3.48E-02 |
| ENSGALG00010004969 | YTHDC1 | 1,281 | 1,357 | 1,206 | 1.124 | 1.19E-02 |
| ENSGALG00010004974 | MTPAP | 1,671 | 1,788 | 1,554 | 1.151 | 1.10E-03 |
| ENSGALG00010004976 |  | 122 | 98 | 146 | 0.676 | 4.82E-03 |
| ENSGALG00010004996 | GPR158 | 135 | 114 | 155 | 0.733 | 9.50E-03 |
| ENSGALG00010005033 | USP14 | 2,540 | 2,207 | 2,873 | 0.769 | 2.10E-04 |
| ENSGALG00010005037 | THNSL1 | 531 | 572 | 490 | 1.165 | 1.98E-02 |
| ENSGALG00010005049 |  | 7 | 9 | 4 | 2.153 | 1.22E-02 |
| ENSGALG00010005054 |  | 20 | 27 | 12 | 2.291 | 8.12E-03 |
| ENSGALG00010005060 | POMP | 3,314 | 2,972 | 3,657 | 0.813 | 3.85E-03 |
| ENSGALG00010005104 | ENPP3 | 255 | 291 | 219 | 1.325 | 1.20E-03 |
| ENSGALG00010005114 | PHLDB2 | 856 | 954 | 757 | 1.26 | 4.01E-04 |
| ENSGALG00010005122 | IL8L1 | 4 | 1 | 7 | 0.163 | 1.22E-03 |
| ENSGALG00010005141 | CCDC170 | 34 | 40 | 27 | 1.459 | 1.41E-02 |
| ENSGALG00010005146 | TGFB3 | 11,799 | 9,109 | 14,489 | 0.629 | 2.26E-03 |
| ENSGALG00010005150 | ALCAM | 839 | 753 | 926 | 0.813 | 1.74E-03 |
| ENSGALG00010005165 | METTL21C | 143 | 92 | 195 | 0.474 | 2.75E-03 |
| ENSGALG00010005167 | GUCY1A2 | 116 | 136 | 97 | 1.392 | 1.50E-02 |
| ENSGALG00010005172 | OSBPL1A | 2,028 | 1,890 | 2,165 | 0.873 | 4.72E-02 |
| ENSGALG00010005174 | TPP2 | 19,201 | 24,618 | 13,784 | 1.786 | 1.24E-07 |
| ENSGALG00010005190 |  | 102 | 76 | 127 | 0.597 | 9.01E-03 |
| ENSGALG00010005193 | EPB41L2 | 2,510 | 2,664 | 2,355 | 1.131 | 4.17E-02 |
| ENSGALG00010005198 | TRPC3 | 35 | 63 | 6 | 10.562 | 4.46E-10 |
| ENSGALG00010005211 | ESRRB | 70 | 84 | 56 | 1.492 | 1.41E-02 |
| ENSGALG00010005214 | BBS7 | 145 | 159 | 132 | 1.21 | 2.36E-02 |
| ENSGALG00010005215 | VASH1 | 2,281 | 2,588 | 1,975 | 1.31 | 3.83E-03 |
| ENSGALG00010005218 | GTPBP8 | 276 | 240 | 313 | 0.764 | 1.54E-02 |
| ENSGALG00010005226 | MYCT1 | 338 | 364 | 312 | 1.166 | 2.69E-02 |
| ENSGALG00010005244 | FBXO5 | 86 | 95 | 76 | 1.251 | 1.86E-02 |
| ENSGALG00010005246 |  | 85 | 63 | 108 | 0.575 | 8.95E-03 |
| ENSGALG00010005282 | FLT1 | 241 | 275 | 207 | 1.328 | 4.75E-03 |
| ENSGALG00010005286 |  | 30 | 17 | 43 | 0.395 | 2.17E-03 |
| ENSGALG00010005308 |  | 7 | 11 | 4 | 2.953 | 1.25E-04 |
| ENSGALG00010005311 | YME1L1 | 7,010 | 7,528 | 6,491 | 1.16 | 4.32E-02 |
| ENSGALG00010005313 | MPHOSPH8 | 359 | 391 | 326 | 1.204 | 6.33E-03 |
| ENSGALG00010005321 | HS6ST3 | 42 | 49 | 35 | 1.392 | 3.23E-02 |
| ENSGALG00010005333 | PLXNA4 | 70 | 53 | 87 | 0.613 | 1.97E-02 |
| ENSGALG00010005345 | NOX3 | 5 | 8 | 3 | 3.57 | 1.00E-02 |
| ENSGALG00010005349 | ACBD5 | 4,130 | 3,738 | 4,522 | 0.827 | 4.79E-03 |
| ENSGALG00010005350 |  | 70 | 55 | 85 | 0.65 | 1.34E-02 |
| ENSGALG00010005354 | M1AP | 18 | 23 | 14 | 1.646 | 2.32E-02 |
| ENSGALG00010005360 | CAMK1D | 551 | 393 | 709 | 0.555 | 2.05E-06 |
| ENSGALG00010005362 | ATP1B1 | 4,502 | 3,441 | 5,564 | 0.618 | 1.65E-03 |
| ENSGALG00010005368 | CCDC3 | 97 | 78 | 115 | 0.682 | 1.43E-02 |
| ENSGALG00010005369 | DPT | 1,248 | 1,103 | 1,393 | 0.792 | 1.31E-03 |
| ENSGALG00010005377 | EMCN | 1,639 | 1,840 | 1,439 | 1.28 | 5.99E-06 |
| ENSGALG00010005384 | MAPRE2 | 2,345 | 1,965 | 2,724 | 0.722 | 2.17E-03 |
| ENSGALG00010005389 | MCM10 | 244 | 327 | 161 | 2.024 | 1.41E-11 |
| ENSGALG00010005394 | PHYH | 4,198 | 4,568 | 3,828 | 1.194 | 3.05E-02 |
| ENSGALG00010005398 | SEPHS1 | 1,111 | 1,194 | 1,029 | 1.162 | 4.77E-03 |
| ENSGALG00010005404 | BEND7 | 856 | 947 | 765 | 1.239 | 3.10E-03 |
| ENSGALG00010005409 | PRKCQ | 8,356 | 6,766 | 9,945 | 0.68 | 3.88E-04 |
| ENSGALG00010005423 | ST3GAL6 | 3,397 | 3,848 | 2,946 | 1.307 | 1.66E-02 |
| ENSGALG00010005443 | BANK1 | 20 | 14 | 26 | 0.543 | 1.27E-02 |
| ENSGALG00010005445 | SFT2D2 | 272 | 327 | 216 | 1.51 | 4.40E-06 |
| ENSGALG00010005453 | UST | 583 | 541 | 625 | 0.865 | 2.39E-02 |
| ENSGALG00010005474 | COL8A1 | 596 | 523 | 669 | 0.781 | 6.97E-03 |
| ENSGALG00010005477 | TMEM161B | 537 | 602 | 471 | 1.278 | 1.90E-02 |
| ENSGALG00010005484 | GINM1 | 1,473 | 1,277 | 1,669 | 0.765 | 4.87E-03 |
| ENSGALG00010005510 | MYOZ2 | 1,082 | 725 | 1,439 | 0.504 | 5.63E-04 |
| ENSGALG00010005511 | AHCYL2 | 1,397 | 1,608 | 1,186 | 1.357 | 5.52E-06 |
| ENSGALG00010005515 | PCCA | 464 | 497 | 430 | 1.159 | 4.43E-02 |
| ENSGALG00010005518 | HPGDS | 187 | 229 | 144 | 1.584 | 1.66E-02 |
| ENSGALG00010005531 | SUSD1 | 530 | 421 | 638 | 0.659 | 2.58E-02 |
| ENSGALG00010005541 |  | 5 | 3 | 7 | 0.395 | 2.87E-02 |
| ENSGALG00010005554 | CRYL1 | 4,716 | 6,021 | 3,412 | 1.765 | 7.26E-05 |
| ENSGALG00010005555 | PRSS12 | 55 | 46 | 63 | 0.715 | 2.93E-02 |
| ENSGALG00010005564 | TM9SF2 | 3,792 | 3,586 | 3,998 | 0.897 | 1.06E-02 |
| ENSGALG00010005573 | CHST9 | 75 | 46 | 104 | 0.437 | 2.90E-05 |
| ENSGALG00010005618 | PHACTR2 | 1,649 | 1,802 | 1,496 | 1.204 | 2.45E-06 |
| ENSGALG00010005632 | ITGB1 | 11,886 | 10,972 | 12,800 | 0.857 | 1.48E-04 |
| ENSGALG00010005675 | GJA3 | 29 | 35 | 23 | 1.503 | 4.56E-03 |
| ENSGALG00010005681 | EPC1 | 776 | 828 | 723 | 1.146 | 1.13E-02 |
| ENSGALG00010005696 | NUBPL | 236 | 213 | 260 | 0.822 | 1.45E-03 |
| ENSGALG00010005704 | AKAP12 | 3,170 | 3,378 | 2,962 | 1.14 | 4.92E-02 |
| ENSGALG00010005724 | PROSER2 | 665 | 555 | 776 | 0.715 | 2.70E-02 |
| ENSGALG00010005759 |  | 6 | 5 | 8 | 0.519 | 1.42E-02 |
| ENSGALG00010005790 | MUSK | 193 | 159 | 227 | 0.7 | 1.03E-02 |
| ENSGALG00010005821 | DOCK9 | 1,396 | 1,511 | 1,281 | 1.179 | 3.98E-02 |
| ENSGALG00010005822 |  | 17 | 13 | 22 | 0.588 | 1.92E-02 |
| ENSGALG00010005830 | USP12 | 67 | 57 | 78 | 0.731 | 6.42E-03 |
| ENSGALG00010005837 |  | 4 | 3 | 6 | 0.43 | 4.77E-02 |
| ENSGALG00010005871 | EGLN3 | 1,959 | 2,144 | 1,775 | 1.208 | 1.27E-02 |
| ENSGALG00010005872 | STK24 | 680 | 626 | 734 | 0.853 | 4.99E-02 |
| ENSGALG00010005875 | CDK8 | 277 | 306 | 249 | 1.227 | 1.64E-05 |
| ENSGALG00010005888 | IMPG2 | 173 | 102 | 243 | 0.419 | 2.35E-04 |
| ENSGALG00010005891 | RNF6 | 504 | 536 | 472 | 1.132 | 2.72E-02 |
| ENSGALG00010005902 | EAPP | 267 | 244 | 290 | 0.841 | 2.70E-02 |
| ENSGALG00010005922 |  | 2 | 3 | 1 | 3.861 | 8.36E-03 |
| ENSGALG00010005965 |  | 28 | 54 | 1 | 37.003 | 1.84E-03 |
| ENSGALG00010005970 | SHISA2 | 299 | 238 | 361 | 0.66 | 1.50E-03 |
| ENSGALG00010005973 |  | 19 | 33 | 5 | 6.253 | 8.97E-04 |
| ENSGALG00010005979 | SUV39H2 | 907 | 980 | 835 | 1.175 | 6.45E-03 |
| ENSGALG00010005982 |  | 10 | 18 | 3 | 6.21 | 5.26E-03 |
| *ENSGALG00010006004* | *DCLRE1C* | *48* | *57* | *40* | *1.44* | *6.04E-03* |
| ENSGALG00010006010 | IPO5 | 5,954 | 5,319 | 6,590 | 0.807 | 4.03E-07 |
| ENSGALG00010006026 |  | 10 | 18 | 2 | 7.404 | 8.01E-03 |
| ENSGALG00010006028 | ARL5B | 1,260 | 1,384 | 1,137 | 1.218 | 8.75E-04 |
| ENSGALG00010006052 | RALGAPA1 | 721 | 791 | 651 | 1.215 | 3.77E-02 |
| ENSGALG00010006064 | DMTF1 | 512 | 556 | 468 | 1.19 | 1.99E-03 |
| ENSGALG00010006066 | ARHGAP18 | 1,530 | 1,796 | 1,264 | 1.421 | 3.16E-03 |
| ENSGALG00010006076 | PODXL | 1,855 | 2,110 | 1,601 | 1.318 | 7.11E-03 |
| ENSGALG00010006107 | PGF | 330 | 262 | 399 | 0.657 | 3.02E-02 |
| ENSGALG00010006123 | MLH3 | 1,434 | 1,551 | 1,317 | 1.179 | 3.02E-02 |
| ENSGALG00010006151 |  | 64 | 56 | 72 | 0.793 | 2.34E-02 |
| ENSGALG00010006163 |  | 20 | 12 | 28 | 0.433 | 3.50E-03 |
| ENSGALG00010006175 | IL2RA | 150 | 119 | 181 | 0.657 | 6.24E-03 |
| ENSGALG00010006202 | SACS | 4,141 | 4,928 | 3,354 | 1.469 | 1.54E-03 |
| ENSGALG00010006203 | MMR1L4 | 753 | 615 | 890 | 0.691 | 2.13E-02 |
| ENSGALG00010006241 | MRC1 | 191 | 210 | 172 | 1.225 | 3.55E-02 |
| ENSGALG00010006245 | PFKFB3 | 678 | 524 | 831 | 0.631 | 3.93E-02 |
| ENSGALG00010006254 | PROS1 | 2,250 | 2,065 | 2,435 | 0.847 | 4.37E-02 |
| ENSGALG00010006275 |  | 65 | 79 | 52 | 1.51 | 2.70E-02 |
| ENSGALG00010006279 | HACD1 | 19,981 | 18,572 | 21,390 | 0.868 | 1.43E-02 |
| ENSGALG00010006291 |  | 352 | 260 | 445 | 0.585 | 1.95E-03 |
| ENSGALG00010006328 | SYNDIG1L | 135 | 68 | 201 | 0.336 | 2.76E-07 |
| ENSGALG00010006343 | SEC23A | 5,837 | 5,599 | 6,075 | 0.922 | 1.67E-02 |
| ENSGALG00010006348 |  | 317 | 371 | 261 | 1.423 | 3.32E-02 |
| ENSGALG00010006463 | SCO2 | 217 | 176 | 259 | 0.679 | 4.39E-03 |
| ENSGALG00010006478 |  | 786 | 713 | 859 | 0.83 | 1.95E-02 |
| ENSGALG00010006496 | UBE2H | 6,643 | 6,255 | 7,030 | 0.89 | 2.58E-02 |
| ENSGALG00010006499 | CCR6 | 11 | 8 | 14 | 0.568 | 3.28E-02 |
| ENSGALG00010006532 | KLHDC10 | 1,954 | 2,141 | 1,768 | 1.212 | 2.97E-04 |
| ENSGALG00010006533 |  | 20 | 26 | 14 | 1.848 | 2.27E-02 |
| ENSGALG00010006535 |  | 4 | 2 | 6 | 0.394 | 1.79E-02 |
| ENSGALG00010006538 | TEK | 598 | 668 | 528 | 1.266 | 9.78E-06 |
| ENSGALG00010006544 | MOB3B | 28 | 36 | 19 | 1.855 | 1.11E-04 |
| ENSGALG00010006550 |  | 20 | 26 | 15 | 1.792 | 1.76E-03 |
| ENSGALG00010006561 |  | 16 | 10 | 23 | 0.411 | 1.25E-03 |
| ENSGALG00010006569 |  | 24 | 20 | 28 | 0.69 | 2.58E-02 |
| ENSGALG00010006596 |  | 3 | 4 | 1 | 3.598 | 7.95E-03 |
| ENSGALG00010006605 |  | 19 | 25 | 13 | 1.881 | 4.46E-05 |
| ENSGALG00010006608 | KL | 128 | 149 | 107 | 1.404 | 4.30E-02 |
| ENSGALG00010006615 | GRAMD1C | 331 | 306 | 356 | 0.862 | 3.83E-02 |
| ENSGALG00010006653 | CFL2 | 24,906 | 22,955 | 26,858 | 0.855 | 5.48E-03 |
| ENSGALG00010006682 | ARHGAP10 | 544 | 465 | 623 | 0.746 | 2.89E-03 |
| ENSGALG00010006715 | IRF8 | 570 | 498 | 643 | 0.774 | 1.40E-02 |
| ENSGALG00010006716 |  | 1,490 | 1,606 | 1,374 | 1.17 | 3.88E-02 |
| ENSGALG00010006738 |  | 14 | 18 | 10 | 1.778 | 4.91E-03 |
| ENSGALG00010006740 | BRCA2 | 218 | 270 | 167 | 1.62 | 1.53E-07 |
| ENSGALG00010006767 |  | 938 | 818 | 1,058 | 0.773 | 4.79E-02 |
| ENSGALG00010006814 |  | 21 | 36 | 7 | 5.653 | 3.49E-08 |
| ENSGALG00010006843 | STON2 | 830 | 679 | 980 | 0.693 | 8.84E-04 |
| ENSGALG00010006888 |  | 94 | 81 | 107 | 0.758 | 8.50E-03 |
| ENSGALG00010006903 | B3GLCT | 324 | 346 | 302 | 1.143 | 4.54E-02 |
| ENSGALG00010006926 | ID4 | 16 | 12 | 21 | 0.599 | 4.33E-03 |
| ENSGALG00010006947 |  | 88 | 97 | 79 | 1.233 | 4.54E-02 |
| ENSGALG00010006960 |  | 30 | 19 | 41 | 0.461 | 1.23E-02 |
| ENSGALG00010006963 | SLC46A2 | 57 | 45 | 68 | 0.662 | 4.05E-02 |
| ENSGALG00010006973 |  | 4 | 6 | 2 | 2.747 | 1.70E-02 |
| ENSGALG00010007011 | HMGB1 | 3,958 | 4,109 | 3,806 | 1.08 | 3.92E-02 |
| ENSGALG00010007017 | SRD5A2 | 345 | 267 | 421 | 0.635 | 2.69E-03 |
| ENSGALG00010007092 | DYNC1LI2 | 3,521 | 3,292 | 3,751 | 0.878 | 1.58E-02 |
| ENSGALG00010007100 |  | 44 | 54 | 35 | 1.531 | 8.03E-03 |
| ENSGALG00010007130 | IRF2 | 900 | 845 | 956 | 0.883 | 4.43E-02 |
| ENSGALG00010007143 | CKMT2 | 44,720 | 52,668 | 36,771 | 1.432 | 1.11E-02 |
| ENSGALG00010007162 |  | 1,207 | 861 | 1,553 | 0.554 | 1.44E-05 |
| ENSGALG00010007165 |  | 27 | 18 | 35 | 0.495 | 6.74E-03 |
| ENSGALG00010007173 |  | 72 | 101 | 43 | 2.349 | 4.03E-05 |
| ENSGALG00010007184 | VGLL3 | 479 | 439 | 519 | 0.845 | 2.84E-02 |
| ENSGALG00010007200 |  | 292 | 339 | 245 | 1.379 | 1.02E-02 |
| ENSGALG00010007214 | MTHFSD | 491 | 560 | 423 | 1.33 | 2.16E-04 |
| ENSGALG00010007268 |  | 58 | 70 | 46 | 1.524 | 2.92E-03 |
| ENSGALG00010007270 | CDH5 | 2,544 | 2,943 | 2,145 | 1.372 | 5.79E-06 |
| *ENSGALG00010007287* |  | *185* | *357* | *13* | *28.143* | *8.58E-07* |
| ENSGALG00010007298 |  | 284 | 224 | 345 | 0.649 | 1.29E-04 |
| ENSGALG00010007300 | CCDC125 | 31 | 22 | 39 | 0.573 | 1.48E-02 |
| ENSGALG00010007318 | RMDN2 | 360 | 438 | 283 | 1.552 | 3.84E-04 |
| ENSGALG00010007360 | ROBO1 | 123 | 110 | 137 | 0.798 | 4.94E-02 |
| ENSGALG00010007369 |  | 14 | 9 | 19 | 0.452 | 9.00E-03 |
| ENSGALG00010007379 |  | 10 | 4 | 15 | 0.273 | 4.25E-03 |
| ENSGALG00010007429 | C21orf91 | 625 | 479 | 771 | 0.621 | 1.27E-05 |
| ENSGALG00010007437 | TUSC1 | 35 | 27 | 42 | 0.651 | 2.51E-02 |
| ENSGALG00010007441 | STRN | 549 | 607 | 491 | 1.238 | 1.69E-02 |
| ENSGALG00010007467 | CHODL | 143 | 99 | 186 | 0.534 | 3.82E-02 |
| ENSGALG00010007477 |  | 44 | 50 | 37 | 1.366 | 4.63E-02 |
| ENSGALG00010007485 | CTSC | 2,540 | 2,026 | 3,054 | 0.663 | 2.28E-02 |
| ENSGALG00010007528 |  | 5 | 2 | 9 | 0.186 | 4.92E-03 |
| ENSGALG00010007539 | RPS3A | 29,731 | 26,955 | 32,508 | 0.829 | 4.46E-02 |
| ENSGALG00010007542 | MRPL36 | 1,048 | 1,195 | 901 | 1.328 | 1.04E-02 |
| ENSGALG00010007544 | MLYCD | 341 | 380 | 303 | 1.256 | 2.72E-02 |
| ENSGALG00010007551 | FEZ2 | 1,773 | 1,557 | 1,989 | 0.783 | 1.93E-04 |
| ENSGALG00010007565 |  | 59 | 94 | 23 | 4.018 | 2.06E-09 |
| ENSGALG00010007566 | FHIP1A | 544 | 458 | 629 | 0.728 | 6.76E-03 |
| ENSGALG00010007577 | WFDC1 | 1,175 | 964 | 1,386 | 0.695 | 1.23E-02 |
| ENSGALG00010007601 | TMEM154 | 33 | 27 | 39 | 0.707 | 4.78E-02 |
| ENSGALG00010007624 | TPPP | 322 | 365 | 280 | 1.309 | 8.78E-03 |
| ENSGALG00010007638 |  | 103 | 84 | 122 | 0.691 | 3.82E-02 |
| ENSGALG00010007651 | CTNNAL1 | 247 | 203 | 292 | 0.694 | 1.40E-03 |
| ENSGALG00010007673 | FHDC1 | 80 | 46 | 114 | 0.398 | 1.02E-04 |
| ENSGALG00010007713 | SCLT1 | 284 | 232 | 336 | 0.69 | 7.44E-07 |
| ENSGALG00010007716 |  | 2 | 4 | 1 | 3.612 | 4.73E-03 |
| ENSGALG00010007719 | LTBP1 | 1,413 | 1,206 | 1,619 | 0.745 | 4.07E-05 |
| ENSGALG00010007721 |  | 954 | 1,299 | 608 | 2.136 | 1.85E-02 |
| ENSGALG00010007734 |  | 11 | 14 | 8 | 1.891 | 4.71E-03 |
| ENSGALG00010007768 |  | 191 | 157 | 225 | 0.694 | 2.39E-04 |
| ENSGALG00010007786 | SAMSN1 | 130 | 93 | 167 | 0.556 | 5.13E-04 |
| ENSGALG00010007788 | PALM2AKAP2 | 87 | 115 | 58 | 1.966 | 5.15E-05 |
| ENSGALG00010007804 |  | 1 | 3 | 0 | 7.457 | 9.03E-03 |
| ENSGALG00010007835 | GUCY1A1 | 646 | 737 | 555 | 1.33 | 2.93E-04 |
| ENSGALG00010007846 |  | 104 | 85 | 123 | 0.691 | 4.98E-03 |
| ENSGALG00010007852 | SLC20A2 | 1,278 | 989 | 1,567 | 0.631 | 3.15E-08 |
| ENSGALG00010007861 | INTU | 86 | 96 | 75 | 1.27 | 4.96E-03 |
| ENSGALG00010007867 | ZNF516 | 158 | 141 | 175 | 0.801 | 4.25E-02 |
| ENSGALG00010007871 | HSPA4L | 1,663 | 1,539 | 1,787 | 0.861 | 1.15E-02 |
| ENSGALG00010007873 |  | 1,391 | 2,622 | 160 | 16.388 | 1.68E-33 |
| ENSGALG00010007874 |  | 5 | 6 | 3 | 1.989 | 4.69E-02 |
| ENSGALG00010007889 | DCAF6 | 21,278 | 19,667 | 22,890 | 0.859 | 1.55E-02 |
| ENSGALG00010007893 | MFSD8 | 518 | 447 | 588 | 0.76 | 6.91E-05 |
| ENSGALG00010007896 | TIMMDC1 | 2,383 | 2,053 | 2,713 | 0.757 | 8.26E-03 |
| ENSGALG00010007899 | APRT | 140 | 127 | 153 | 0.827 | 2.41E-02 |
| ENSGALG00010007900 | FHOD3 | 2,807 | 2,061 | 3,553 | 0.58 | 1.95E-03 |
| ENSGALG00010007912 | TRAPPC2L | 159 | 144 | 175 | 0.82 | 4.66E-02 |
| ENSGALG00010007926 | EIF3E | 7,748 | 7,154 | 8,342 | 0.858 | 1.96E-02 |
| ENSGALG00010007927 | DLL1 | 99 | 108 | 90 | 1.209 | 4.19E-02 |
| ENSGALG00010007929 | LRRC14B | 430 | 534 | 326 | 1.64 | 4.76E-05 |
| ENSGALG00010007942 | PGRMC2 | 733 | 683 | 783 | 0.872 | 9.16E-03 |
| ENSGALG00010007950 | ARC | 351 | 159 | 543 | 0.293 | 1.87E-03 |
| ENSGALG00010007958 |  | 74 | 56 | 93 | 0.595 | 1.97E-02 |
| ENSGALG00010007978 | JADE1 | 721 | 819 | 623 | 1.313 | 2.72E-02 |
| ENSGALG00010007987 |  | 147 | 116 | 179 | 0.65 | 6.89E-03 |
| ENSGALG00010007991 | PCF11 | 1,533 | 1,666 | 1,399 | 1.19 | 8.89E-03 |
| ENSGALG00010007996 | NUDCD1 | 852 | 919 | 785 | 1.172 | 2.66E-04 |
| ENSGALG00010007997 | TMEM192 | 189 | 176 | 203 | 0.863 | 3.26E-02 |
| ENSGALG00010008001 | ALDH5A1 | 2,384 | 2,647 | 2,121 | 1.248 | 6.24E-03 |
| ENSGALG00010008016 | GSR | 500 | 426 | 573 | 0.743 | 3.39E-05 |
| ENSGALG00010008030 | GJA5 | 221 | 254 | 188 | 1.347 | 1.51E-02 |
| ENSGALG00010008031 | MAP3K21 | 54 | 41 | 67 | 0.618 | 1.68E-02 |
| ENSGALG00010008045 | DCDC2 | 118 | 69 | 168 | 0.407 | 1.44E-02 |
| ENSGALG00010008078 | GUCY1B1 | 1,702 | 1,913 | 1,492 | 1.283 | 4.29E-03 |
| ENSGALG00010008092 | SPIRE2 | 69 | 17 | 121 | 0.143 | 1.97E-03 |
| ENSGALG00010008101 | CENPQ | 105 | 132 | 77 | 1.711 | 1.17E-06 |
| ENSGALG00010008105 | MMUT | 1,155 | 1,439 | 870 | 1.654 | 3.27E-06 |
| ENSGALG00010008145 |  | 138 | 117 | 158 | 0.739 | 7.10E-04 |
| ENSGALG00010008150 | TERT | 6 | 3 | 8 | 0.467 | 2.85E-02 |
| ENSGALG00010008152 |  | 1,023 | 871 | 1,176 | 0.74 | 3.03E-02 |
| ENSGALG00010008161 | TMEFF1 | 43 | 25 | 60 | 0.413 | 1.18E-02 |
| ENSGALG00010008162 | HSBP1L1 | 29 | 20 | 37 | 0.552 | 1.22E-03 |
| ENSGALG00010008164 | TG | 51 | 62 | 41 | 1.503 | 3.05E-02 |
| ENSGALG00010008175 |  | 35 | 26 | 44 | 0.579 | 3.78E-02 |
| ENSGALG00010008179 | SEMA6A | 536 | 607 | 464 | 1.306 | 7.42E-05 |
| ENSGALG00010008202 | KCNG2 | 289 | 247 | 330 | 0.749 | 3.52E-02 |
| ENSGALG00010008209 | PPARGC1B | 106 | 124 | 88 | 1.411 | 2.62E-03 |
| ENSGALG00010008219 | NIP7 | 416 | 371 | 462 | 0.805 | 2.35E-02 |
| ENSGALG00010008227 | DUSP1 | 671 | 560 | 782 | 0.716 | 1.59E-02 |
| ENSGALG00010008240 | RCAN2 | 1,673 | 1,162 | 2,185 | 0.532 | 2.43E-06 |
| ENSGALG00010008245 | FBXO32 | 1,364 | 1,077 | 1,650 | 0.653 | 4.55E-04 |
| ENSGALG00010008247 | GASK1B | 447 | 512 | 383 | 1.338 | 2.69E-03 |
| ENSGALG00010008263 | C1orf101 | 4 | 3 | 6 | 0.482 | 4.69E-02 |
| ENSGALG00010008265 |  | 8 | 5 | 11 | 0.458 | 2.95E-02 |
| ENSGALG00010008266 | ECSCR | 500 | 547 | 454 | 1.205 | 9.64E-03 |
| ENSGALG00010008273 | TMEM144 | 459 | 412 | 507 | 0.811 | 1.93E-02 |
| ENSGALG00010008275 | CELSR1 | 290 | 221 | 360 | 0.615 | 2.62E-03 |
| ENSGALG00010008278 | CYP39A1 | 360 | 315 | 404 | 0.779 | 1.03E-02 |
| ENSGALG00010008289 | MTMR2 | 695 | 614 | 776 | 0.79 | 5.12E-03 |
| ENSGALG00010008290 | FAM13A | 777 | 569 | 985 | 0.577 | 5.70E-06 |
| ENSGALG00010008293 | PLA2G7 | 1,024 | 906 | 1,141 | 0.793 | 1.70E-02 |
| ENSGALG00010008298 |  | 1,029 | 1,150 | 908 | 1.27 | 1.63E-04 |
| ENSGALG00010008312 | ZBTB18 | 7,339 | 8,144 | 6,534 | 1.247 | 6.33E-03 |
| ENSGALG00010008316 | ADGRF5 | 372 | 437 | 307 | 1.423 | 5.16E-03 |
| ENSGALG00010008318 | ADPRH | 3,471 | 2,993 | 3,950 | 0.758 | 1.71E-02 |
| ENSGALG00010008319 | ERP44 | 1,663 | 1,575 | 1,750 | 0.901 | 2.23E-02 |
| ENSGALG00010008325 | AADAT | 268 | 297 | 239 | 1.241 | 2.33E-02 |
| ENSGALG00010008327 | TGFB1 | 158 | 123 | 193 | 0.637 | 2.67E-02 |
| ENSGALG00010008334 | STX17 | 1,422 | 1,665 | 1,179 | 1.413 | 8.13E-03 |
| ENSGALG00010008340 | PDIA4 | 2,623 | 2,384 | 2,862 | 0.833 | 2.17E-03 |
| ENSGALG00010008351 | IST1 | 2,234 | 2,069 | 2,400 | 0.862 | 1.19E-04 |
| ENSGALG00010008395 | ENDOD1 | 442 | 367 | 516 | 0.712 | 2.22E-04 |
| ENSGALG00010008396 | LRRTM2 | 5 | 2 | 7 | 0.329 | 2.26E-03 |
| ENSGALG00010008407 | GNAI1 | 593 | 649 | 537 | 1.207 | 5.29E-03 |
| ENSGALG00010008410 | NAPRT | 324 | 290 | 359 | 0.806 | 8.90E-03 |
| ENSGALG00010008418 | TMEM245 | 1,838 | 2,056 | 1,620 | 1.269 | 1.11E-02 |
| ENSGALG00010008424 | PPIP5K2 | 455 | 486 | 424 | 1.145 | 3.58E-02 |
| ENSGALG00010008429 | IGFBP3 | 3,043 | 3,467 | 2,619 | 1.325 | 1.44E-03 |
| ENSGALG00010008434 | PTCHD4 | 2 | 3 | 1 | 4.862 | 1.74E-02 |
| ENSGALG00010008456 | NHLRC1 | 409 | 459 | 358 | 1.285 | 1.33E-02 |
| ENSGALG00010008497 | PAM | 547 | 510 | 584 | 0.873 | 4.96E-02 |
| ENSGALG00010008504 | RNF139 | 3,021 | 3,340 | 2,701 | 1.237 | 2.47E-02 |
| ENSGALG00010008516 | MRE11 | 471 | 424 | 518 | 0.819 | 4.85E-02 |
| ENSGALG00010008522 | NUDT12 | 400 | 432 | 368 | 1.175 | 3.93E-02 |
| ENSGALG00010008529 | GPR83 | 15 | 20 | 10 | 2.035 | 1.74E-04 |
| ENSGALG00010008564 |  | 52 | 44 | 59 | 0.749 | 4.96E-02 |
| ENSGALG00010008580 | PANX1 | 76 | 62 | 91 | 0.684 | 4.79E-02 |
| ENSGALG00010008588 | PYCR3 | 91 | 76 | 106 | 0.718 | 1.98E-02 |
| ENSGALG00010008628 |  | 7 | 4 | 10 | 0.375 | 1.74E-03 |
| ENSGALG00010008666 | SLC16A9 | 887 | 1,162 | 612 | 1.901 | 3.27E-06 |
| ENSGALG00010008674 | RXFP1 | 93 | 105 | 80 | 1.316 | 3.72E-02 |
| ENSGALG00010008686 |  | 17 | 23 | 11 | 2.128 | 1.95E-02 |
| ENSGALG00010008750 | PFDN1 | 984 | 825 | 1,144 | 0.722 | 1.28E-02 |
| ENSGALG00010008752 | GOLM1 | 2,516 | 2,163 | 2,867 | 0.754 | 3.77E-04 |
| ENSGALG00010008764 | ETFDH | 2,251 | 2,612 | 1,889 | 1.382 | 6.24E-09 |
| ENSGALG00010008767 |  | 10 | 13 | 6 | 2.074 | 4.83E-02 |
| ENSGALG00010008824 | GREM2 | 175 | 251 | 100 | 2.511 | 1.17E-08 |
| ENSGALG00010008839 | ZNF512 | 1,935 | 2,054 | 1,817 | 1.131 | 1.48E-02 |
| ENSGALG00010008846 | SLC35D2 | 39 | 48 | 31 | 1.509 | 2.11E-02 |
| ENSGALG00010008864 | ADAT1 | 601 | 661 | 542 | 1.22 | 3.40E-02 |
| ENSGALG00010008872 |  | 4 | 5 | 2 | 3.027 | 2.27E-03 |
| ENSGALG00010008904 |  | 9 | 16 | 2 | 9.615 | 9.47E-06 |
| ENSGALG00010008926 |  | 27 | 19 | 35 | 0.547 | 1.44E-02 |
| ENSGALG00010008938 | ZBED4 | 2,084 | 2,292 | 1,876 | 1.222 | 7.32E-03 |
| ENSGALG00010008946 | COL22A1 | 195 | 112 | 278 | 0.402 | 7.99E-05 |
| ENSGALG00010008956 | LVRN | 519 | 246 | 793 | 0.31 | 4.53E-05 |
| ENSGALG00010008960 | LRRC2 | 2,754 | 2,111 | 3,396 | 0.622 | 1.47E-04 |
| ENSGALG00010008971 | ANXA11 | 2,862 | 2,658 | 3,067 | 0.867 | 1.78E-02 |
| ENSGALG00010008996 | CHRM3 | 37 | 44 | 29 | 1.523 | 3.38E-03 |
| ENSGALG00010009002 |  | 920 | 820 | 1,020 | 0.804 | 3.31E-02 |
| ENSGALG00010009026 | RAPGEF2 | 1,034 | 836 | 1,232 | 0.678 | 9.57E-03 |
| ENSGALG00010009057 | NXNL2 | 150 | 188 | 113 | 1.664 | 7.73E-05 |
| *ENSGALG00010009067* |  | *340* | *648* | *31* | *21.144* | *2.08E-05* |
| ENSGALG00010009106 | THSD7B | 343 | 492 | 194 | 2.545 | 5.28E-19 |
| ENSGALG00010009115 | COG5 | 791 | 722 | 859 | 0.841 | 2.35E-02 |
| ENSGALG00010009119 | CISD1 | 10,526 | 11,403 | 9,648 | 1.182 | 3.36E-02 |
| ENSGALG00010009147 | HNMT | 538 | 712 | 365 | 1.949 | 9.05E-06 |
| ENSGALG00010009153 | SECISBP2 | 1,153 | 1,211 | 1,095 | 1.107 | 2.83E-02 |
| ENSGALG00010009159 | PTK2 | 4,411 | 4,756 | 4,066 | 1.17 | 4.02E-02 |
| ENSGALG00010009166 | CLGN | 155 | 115 | 194 | 0.594 | 5.10E-04 |
| ENSGALG00010009199 | SEMA4D | 72 | 59 | 86 | 0.683 | 5.19E-03 |
| ENSGALG00010009210 | PCDH15 | 8 | 4 | 12 | 0.35 | 1.98E-03 |
| ENSGALG00010009219 | TNFRSF11B | 847 | 729 | 965 | 0.755 | 3.87E-03 |
| ENSGALG00010009220 | GADD45G | 515 | 406 | 624 | 0.651 | 2.61E-03 |
| ENSGALG00010009232 | ECH1 | 254 | 198 | 310 | 0.638 | 4.19E-02 |
| ENSGALG00010009235 | KLHDC4 | 2,436 | 2,673 | 2,199 | 1.216 | 2.22E-02 |
| ENSGALG00010009243 | BCAP29 | 2,393 | 1,755 | 3,030 | 0.579 | 2.51E-09 |
| ENSGALG00010009258 | DNAJC12 | 469 | 386 | 551 | 0.701 | 5.00E-02 |
| ENSGALG00010009279 | ELMOD2 | 1,171 | 1,291 | 1,050 | 1.229 | 1.99E-02 |
| ENSGALG00010009286 |  | 45 | 35 | 54 | 0.662 | 4.86E-02 |
| ENSGALG00010009297 | CTNNA3 | 1,190 | 1,062 | 1,318 | 0.806 | 1.39E-02 |
| ENSGALG00010009299 | MAP4K1 | 162 | 116 | 209 | 0.555 | 2.23E-02 |
| ENSGALG00010009313 | UNC13B | 21 | 17 | 24 | 0.685 | 4.70E-02 |
| ENSGALG00010009326 | DLD | 6,779 | 7,857 | 5,700 | 1.379 | 1.44E-04 |
| ENSGALG00010009330 | EIF3K | 4,086 | 3,502 | 4,670 | 0.75 | 1.41E-03 |
| ENSGALG00010009334 | SS18L2 | 160 | 137 | 183 | 0.752 | 2.78E-02 |
| ENSGALG00010009337 | ZNF330 | 782 | 854 | 710 | 1.204 | 2.02E-02 |
| ENSGALG00010009340 | SYK | 101 | 78 | 123 | 0.634 | 2.42E-02 |
| ENSGALG00010009360 | TNFAIP8 | 340 | 290 | 391 | 0.742 | 1.76E-03 |
| ENSGALG00010009363 | TAF2 | 1,671 | 1,766 | 1,576 | 1.12 | 3.72E-02 |
| ENSGALG00010009365 | CA5B | 293 | 255 | 330 | 0.771 | 3.93E-02 |
| ENSGALG00010009371 | PHTF2 | 5,408 | 5,036 | 5,779 | 0.872 | 4.31E-02 |
| ENSGALG00010009387 | DEPTOR | 3,929 | 4,474 | 3,383 | 1.323 | 7.85E-04 |
| ENSGALG00010009425 |  | 7 | 10 | 4 | 2.284 | 7.11E-03 |
| ENSGALG00010009446 |  | 14,828 | 12,632 | 17,024 | 0.742 | 5.65E-04 |
| ENSGALG00010009453 | GSAP | 358 | 393 | 322 | 1.221 | 2.56E-02 |
| ENSGALG00010009463 | MRPL13 | 1,211 | 1,094 | 1,327 | 0.824 | 3.25E-02 |
| ENSGALG00010009477 | ZNF365 | 621 | 737 | 505 | 1.463 | 5.22E-04 |
| ENSGALG00010009484 | RTKN2 | 159 | 189 | 129 | 1.458 | 2.24E-05 |
| ENSGALG00010009490 | SNTB1 | 1,014 | 1,172 | 856 | 1.368 | 1.25E-03 |
| ENSGALG00010009498 | ORC4 | 311 | 292 | 330 | 0.886 | 1.79E-02 |
| ENSGALG00010009503 | IL11RA | 605 | 515 | 694 | 0.742 | 5.56E-03 |
| ENSGALG00010009540 | RAB10 | 14,244 | 15,385 | 13,104 | 1.174 | 1.85E-02 |
| ENSGALG00010009542 | WDR1 | 6,212 | 5,677 | 6,746 | 0.842 | 3.74E-02 |
| ENSGALG00010009606 | CHSY3 | 91 | 55 | 128 | 0.426 | 9.36E-11 |
| ENSGALG00010009608 | AFAP1 | 922 | 839 | 1,005 | 0.835 | 4.49E-02 |
| ENSGALG00010009612 | DERL1 | 1,841 | 1,696 | 1,986 | 0.854 | 4.33E-04 |
| ENSGALG00010009620 | TMOD1 | 4,946 | 3,427 | 6,465 | 0.53 | 1.75E-07 |
| ENSGALG00010009623 | XPO5 | 2,076 | 2,194 | 1,958 | 1.121 | 4.02E-02 |
| ENSGALG00010009637 | CYBA | 184 | 151 | 217 | 0.694 | 6.12E-03 |
| ENSGALG00010009640 | NPR2 | 787 | 552 | 1,022 | 0.54 | 1.35E-06 |
| ENSGALG00010009653 | ABLIM2 | 3,178 | 2,762 | 3,594 | 0.768 | 3.83E-03 |
| ENSGALG00010009657 | MSMP | 5 | 2 | 8 | 0.213 | 9.04E-04 |
| ENSGALG00010009678 | JARID2 | 293 | 317 | 270 | 1.172 | 4.60E-02 |
| ENSGALG00010009686 | MAT2B | 4,176 | 4,508 | 3,845 | 1.173 | 8.01E-03 |
| *ENSGALG00010009697* |  | *17* | *22* | *12* | *1.819* | *7.69E-03* |
| ENSGALG00010009700 | SH3TC1 | 80 | 91 | 68 | 1.334 | 3.80E-02 |
| ENSGALG00010009702 |  | 242 | 272 | 213 | 1.283 | 3.46E-02 |
| ENSGALG00010009710 |  | 318 | 349 | 287 | 1.224 | 2.86E-02 |
| ENSGALG00010009717 | EDN1 | 94 | 104 | 83 | 1.261 | 4.09E-02 |
| ENSGALG00010009718 |  | 92 | 105 | 79 | 1.349 | 2.50E-02 |
| ENSGALG00010009722 | GBA2 | 1,138 | 1,076 | 1,201 | 0.896 | 2.84E-02 |
| ENSGALG00010009728 | CCDC149 | 240 | 211 | 269 | 0.784 | 8.48E-03 |
| ENSGALG00010009736 | WISP1 | 84 | 69 | 100 | 0.686 | 3.39E-03 |
| ENSGALG00010009749 | NDRG1 | 1,591 | 1,155 | 2,027 | 0.57 | 3.44E-07 |
| ENSGALG00010009765 | STX10 | 584 | 544 | 623 | 0.872 | 1.01E-03 |
| ENSGALG00010009772 |  | 5 | 9 | 2 | 4.914 | 1.61E-03 |
| ENSGALG00010009777 | ST3GAL1 | 2,011 | 1,697 | 2,325 | 0.73 | 2.90E-03 |
| ENSGALG00010009785 |  | 2 | 4 | 1 | 4.104 | 4.46E-02 |
| ENSGALG00010009786 | C1HXORF36 | 140 | 154 | 126 | 1.225 | 2.34E-02 |
| ENSGALG00010009789 |  | 6 | 10 | 2 | 5.68 | 1.69E-03 |
| ENSGALG00010009793 | TNFAIP6 | 63 | 35 | 91 | 0.381 | 3.02E-04 |
| ENSGALG00010009806 | WDCP | 318 | 289 | 348 | 0.829 | 1.75E-03 |
| *ENSGALG00010009822* |  | *150* | *222* | *79* | *2.847* | *9.17E-13* |
| ENSGALG00010009833 | SOD3 | 444 | 370 | 519 | 0.711 | 1.24E-02 |
| *ENSGALG00010009840* |  | *156* | *205* | *106* | *1.944* | *1.57E-06* |
| ENSGALG00010009847 | TMEM14C | 344 | 306 | 383 | 0.799 | 2.64E-02 |
| ENSGALG00010009857 | ORC5 | 282 | 259 | 304 | 0.854 | 5.63E-03 |
| ENSGALG00010009866 | NAGA | 278 | 239 | 317 | 0.75 | 2.04E-02 |
| *ENSGALG00010009883* |  | *146* | *214* | *79* | *2.747* | *9.20E-16* |
| *ENSGALG00010009925* |  | *4* | *6* | *2* | *2.631* | *2.60E-02* |
| ENSGALG00010009932 |  | 130 | 175 | 85 | 2.04 | 1.96E-05 |
| ENSGALG00010009941 | CACNA1E | 6 | 3 | 9 | 0.367 | 8.50E-03 |
| ENSGALG00010009948 | WBP2NL | 4,422 | 4,243 | 4,601 | 0.922 | 2.12E-02 |
| ENSGALG00010009979 |  | 45 | 28 | 62 | 0.459 | 2.22E-03 |
| ENSGALG00010009990 |  | 3 | 4 | 1 | 4.145 | 3.26E-02 |
| ENSGALG00010009991 | LHFPL3 | 13 | 6 | 20 | 0.312 | 2.60E-03 |
| ENSGALG00010010006 | PPARGC1A | 250 | 313 | 188 | 1.672 | 2.45E-04 |
| ENSGALG00010010007 | CTIF | 1,300 | 1,379 | 1,220 | 1.133 | 3.79E-02 |
| ENSGALG00010010017 | CCDC59 | 599 | 660 | 538 | 1.226 | 1.75E-02 |
| ENSGALG00010010019 |  | 2,002 | 1,709 | 2,296 | 0.744 | 1.10E-03 |
| ENSGALG00010010062 | RGS7BP | 185 | 147 | 222 | 0.664 | 1.99E-02 |
| ENSGALG00010010082 |  | 4 | 6 | 2 | 2.326 | 1.48E-02 |
| ENSGALG00010010094 | SLC2A9 | 53 | 44 | 61 | 0.717 | 4.62E-02 |
| ENSGALG00010010106 | C7orf73 | 534 | 476 | 593 | 0.804 | 4.95E-02 |
| ENSGALG00010010108 |  | 19 | 12 | 25 | 0.465 | 2.23E-03 |
| ENSGALG00010010186 | TNPO1 | 3,058 | 3,273 | 2,843 | 1.152 | 1.93E-02 |
| ENSGALG00010010232 |  | 58 | 41 | 75 | 0.541 | 3.19E-02 |
| ENSGALG00010010243 | ESCO2 | 184 | 205 | 163 | 1.266 | 1.09E-03 |
| ENSGALG00010010257 |  | 108 | 119 | 97 | 1.231 | 3.07E-02 |
| ENSGALG00010010260 |  | 55 | 28 | 82 | 0.342 | 6.58E-03 |
| ENSGALG00010010262 | HSPA9 | 9,308 | 10,639 | 7,978 | 1.334 | 1.67E-04 |
| ENSGALG00010010267 | CCSER2 | 661 | 805 | 517 | 1.558 | 1.46E-04 |
| ENSGALG00010010271 | HUNK | 97 | 112 | 83 | 1.336 | 9.47E-04 |
| ENSGALG00010010280 | HSP90B1 | 8,500 | 7,499 | 9,501 | 0.789 | 2.08E-05 |
| ENSGALG00010010285 |  | 9 | 6 | 12 | 0.523 | 9.29E-03 |
| ENSGALG00010010287 | STAM2 | 2,031 | 1,928 | 2,134 | 0.904 | 2.71E-02 |
| ENSGALG00010010288 | TXNL1 | 2,507 | 2,256 | 2,757 | 0.818 | 4.69E-02 |
| ENSGALG00010010294 |  | 17 | 13 | 22 | 0.602 | 3.20E-02 |
| ENSGALG00010010303 | ETF1 | 7,830 | 8,888 | 6,771 | 1.313 | 2.70E-08 |
| ENSGALG00010010304 |  | 3 | 5 | 1 | 5.086 | 1.34E-02 |
| ENSGALG00010010310 | NT5DC3 | 1,292 | 1,404 | 1,180 | 1.19 | 3.56E-02 |
| ENSGALG00010010311 | CTSV | 2,073 | 1,751 | 2,395 | 0.731 | 4.94E-04 |
| ENSGALG00010010315 |  | 41 | 62 | 19 | 3.238 | 1.06E-05 |
| ENSGALG00010010320 | SYPL1 | 23 | 28 | 18 | 1.516 | 2.88E-02 |
| ENSGALG00010010337 | SCARA3 | 139 | 116 | 161 | 0.718 | 3.88E-02 |
| ENSGALG00010010351 | NAMPTP1 | 16,688 | 15,125 | 18,251 | 0.829 | 3.43E-02 |
| ENSGALG00010010366 | CLU | 5,342 | 3,643 | 7,042 | 0.517 | 1.92E-03 |
| ENSGALG00010010374 | CMTM4 | 243 | 271 | 215 | 1.26 | 3.85E-02 |
| ENSGALG00010010379 | SLC35F6 | 304 | 269 | 339 | 0.794 | 2.47E-02 |
| ENSGALG00010010384 |  | 797 | 868 | 727 | 1.193 | 4.56E-02 |
| ENSGALG00010010385 |  | 9 | 14 | 4 | 4.014 | 4.12E-04 |
| ENSGALG00010010410 | CACNA2D1 | 22,208 | 19,889 | 24,527 | 0.811 | 2.49E-03 |
| ENSGALG00010010411 | CD180 | 28 | 17 | 39 | 0.442 | 8.11E-03 |
| ENSGALG00010010461 | SLC7A5 | 11,792 | 13,538 | 10,046 | 1.348 | 4.92E-03 |
| ENSGALG00010010463 | ACAA2 | 1,804 | 1,622 | 1,987 | 0.816 | 1.66E-02 |
| ENSGALG00010010469 |  | 6 | 8 | 4 | 2.13 | 4.96E-02 |
| ENSGALG00010010471 | KCNJ3 | 42 | 33 | 52 | 0.63 | 1.30E-02 |
| ENSGALG00010010472 | MMRN2 | 1,035 | 1,169 | 901 | 1.296 | 2.08E-05 |
| ENSGALG00010010475 | CCDC71L | 357 | 271 | 444 | 0.611 | 4.33E-06 |
| ENSGALG00010010505 | CD38 | 333 | 282 | 384 | 0.734 | 1.43E-03 |
| ENSGALG00010010516 |  | 5 | 7 | 2 | 3.351 | 7.53E-04 |
| ENSGALG00010010546 | MICALL1 | 2,628 | 2,152 | 3,105 | 0.693 | 7.31E-05 |
| ENSGALG00010010572 | AQP3 | 2,556 | 2,850 | 2,261 | 1.261 | 7.01E-03 |
| ENSGALG00010010578 | GLUD2 | 1,810 | 1,925 | 1,695 | 1.136 | 4.19E-02 |
| ENSGALG00010010588 | SLC25A3 | 18,827 | 20,512 | 17,142 | 1.197 | 2.16E-02 |
| ENSGALG00010010593 | EIF3L | 6,066 | 5,705 | 6,426 | 0.888 | 1.86E-02 |
| ENSGALG00010010599 | F2RL1 | 191 | 158 | 223 | 0.706 | 4.90E-02 |
| ENSGALG00010010623 | SMAD2 | 1,361 | 1,426 | 1,296 | 1.1 | 3.38E-02 |
| ENSGALG00010010624 |  | 5 | 3 | 6 | 0.454 | 9.24E-03 |
| ENSGALG00010010648 | CENPM | 15 | 19 | 12 | 1.587 | 1.07E-02 |
| ENSGALG00010010653 |  | 8 | 13 | 3 | 4.513 | 3.22E-05 |
| ENSGALG00010010655 | C1QTNF7 | 63 | 54 | 73 | 0.731 | 6.60E-03 |
| ENSGALG00010010662 | MGST3 | 524 | 444 | 604 | 0.735 | 7.36E-03 |
| ENSGALG00010010671 | SIGMAR1 | 354 | 308 | 399 | 0.772 | 2.53E-04 |
| ENSGALG00010010678 | KCNIP1 | 5 | 6 | 3 | 2.218 | 2.22E-02 |
| ENSGALG00010010689 | POFUT2 | 1,219 | 1,143 | 1,295 | 0.882 | 1.25E-03 |
| ENSGALG00010010690 | MTPN | 5,828 | 4,709 | 6,947 | 0.678 | 4.90E-05 |
| ENSGALG00010010699 | DNAJC27 | 224 | 206 | 242 | 0.851 | 6.33E-03 |
| ENSGALG00010010710 | UCK2 | 856 | 731 | 981 | 0.745 | 9.85E-05 |
| ENSGALG00010010717 |  | 9 | 7 | 10 | 0.613 | 4.93E-02 |
| ENSGALG00010010730 |  | 2 | 1 | 3 | 0.3 | 4.93E-02 |
| ENSGALG00010010743 | KLHL20 | 867 | 923 | 811 | 1.137 | 3.01E-02 |
| ENSGALG00010010779 | DOCK2 | 172 | 138 | 205 | 0.673 | 2.36E-02 |
| ENSGALG00010010796 |  | 5,220 | 5,794 | 4,646 | 1.247 | 6.87E-08 |
| ENSGALG00010010799 | DARS2 | 478 | 530 | 426 | 1.245 | 4.14E-03 |
| ENSGALG00010010802 |  | 8 | 4 | 12 | 0.358 | 5.65E-04 |
| ENSGALG00010010825 | MAK | 106 | 142 | 70 | 2.039 | 2.21E-06 |
| ENSGALG00010010844 | CDC14B | 650 | 692 | 609 | 1.135 | 1.92E-02 |
| ENSGALG00010010848 | RGR | 14 | 21 | 8 | 2.448 | 7.43E-04 |
| ENSGALG00010010865 |  | 4,401 | 4,762 | 4,039 | 1.179 | 4.78E-02 |
| ENSGALG00010010871 | GCNT2 | 3 | 1 | 5 | 0.19 | 3.44E-02 |
| ENSGALG00010010882 | CEP350 | 4,813 | 5,354 | 4,272 | 1.254 | 1.19E-02 |
| ENSGALG00010010896 | LY86 | 1,895 | 1,546 | 2,244 | 0.689 | 1.63E-03 |
| ENSGALG00010010905 | ENC1 | 1,234 | 1,710 | 757 | 2.259 | 5.03E-08 |
| ENSGALG00010010909 | SLIT3 | 265 | 201 | 328 | 0.611 | 1.01E-02 |
| ENSGALG00010010912 | BAALC | 61 | 27 | 94 | 0.289 | 2.06E-06 |
| ENSGALG00010010915 | APP | 7,665 | 6,360 | 8,969 | 0.709 | 1.27E-05 |
| ENSGALG00010010928 | HECW2 | 384 | 447 | 320 | 1.392 | 2.37E-03 |
| ENSGALG00010010931 | CYYR1 | 64 | 77 | 50 | 1.526 | 3.49E-03 |
| ENSGALG00010010949 | FZD3 | 64 | 44 | 84 | 0.523 | 3.99E-06 |
| ENSGALG00010010956 | AXDND1 | 4 | 3 | 6 | 0.49 | 3.56E-02 |
| ENSGALG00010010966 |  | 23 | 27 | 18 | 1.484 | 2.67E-02 |
| ENSGALG00010010970 | TPM2 | 21,616 | 10,715 | 32,518 | 0.33 | 3.61E-08 |
| ENSGALG00010010991 | C2CD2 | 488 | 533 | 443 | 1.202 | 2.72E-02 |
| ENSGALG00010011003 | FECH | 1,199 | 1,300 | 1,097 | 1.186 | 3.59E-03 |
| ENSGALG00010011004 | UMODL1 | 20 | 14 | 26 | 0.518 | 3.60E-03 |
| ENSGALG00010011024 |  | 5 | 6 | 3 | 2.091 | 2.35E-02 |
| ENSGALG00010011035 |  | 4 | 6 | 2 | 3.964 | 6.87E-04 |
| ENSGALG00010011059 |  | 278 | 322 | 233 | 1.381 | 3.17E-03 |
| ENSGALG00010011091 | YARS2 | 418 | 449 | 388 | 1.158 | 1.27E-02 |
| ENSGALG00010011095 | TNN | 16 | 10 | 21 | 0.474 | 3.22E-02 |
| ENSGALG00010011096 | LDB2 | 417 | 457 | 377 | 1.217 | 1.70E-04 |
| ENSGALG00010011116 | TAPT1 | 1,597 | 1,372 | 1,822 | 0.753 | 6.52E-04 |
| ENSGALG00010011124 | CACYBP | 2,779 | 2,501 | 3,057 | 0.818 | 2.25E-03 |
| ENSGALG00010011128 | GCNT4 | 116 | 97 | 135 | 0.718 | 4.16E-02 |
| ENSGALG00010011130 | DNM1L | 3,816 | 4,083 | 3,549 | 1.151 | 9.63E-05 |
| ENSGALG00010011146 | BMS1 | 924 | 1,010 | 838 | 1.205 | 4.99E-02 |
| ENSGALG00010011158 |  | 22 | 30 | 14 | 2.138 | 4.66E-02 |
| ENSGALG00010011166 | SOX7 | 223 | 246 | 199 | 1.236 | 1.43E-03 |
| ENSGALG00010011175 | GPR52 | 14 | 11 | 18 | 0.601 | 2.88E-03 |
| ENSGALG00010011192 | RPL17 | 11,129 | 9,971 | 12,287 | 0.811 | 2.32E-02 |
| ENSGALG00010011202 |  | 14 | 23 | 6 | 3.704 | 4.46E-05 |
| ENSGALG00010011231 | PLBD1 | 1,616 | 1,298 | 1,934 | 0.671 | 1.55E-03 |
| ENSGALG00010011250 |  | 963 | 1,031 | 895 | 1.151 | 6.89E-03 |
| ENSGALG00010011278 |  | 526 | 218 | 834 | 0.262 | 6.30E-09 |
| ENSGALG00010011295 | FAM167A | 15 | 19 | 11 | 1.71 | 3.93E-02 |
| ENSGALG00010011305 |  | 18 | 22 | 14 | 1.603 | 2.13E-02 |
| ENSGALG00010011318 | PRPF4B | 1,670 | 1,765 | 1,575 | 1.121 | 2.73E-02 |
| ENSGALG00010011340 | PKNOX1 | 487 | 563 | 412 | 1.366 | 6.17E-03 |
| ENSGALG00010011348 | SLC22A23 | 197 | 165 | 229 | 0.717 | 3.47E-02 |
| ENSGALG00010011353 |  | 27 | 36 | 17 | 2.015 | 4.92E-03 |
| ENSGALG00010011371 |  | 79 | 52 | 106 | 0.494 | 1.96E-05 |
| ENSGALG00010011375 | C12orf50 | 476 | 524 | 429 | 1.226 | 5.86E-03 |
| ENSGALG00010011381 | C1H12orf29 | 258 | 285 | 230 | 1.24 | 3.20E-02 |
| ENSGALG00010011383 |  | 93 | 114 | 72 | 1.596 | 4.33E-04 |
| ENSGALG00010011386 | CTSB | 12,428 | 10,537 | 14,320 | 0.736 | 8.63E-05 |
| ENSGALG00010011388 | GPR50 | 60 | 68 | 52 | 1.301 | 1.29E-02 |
| ENSGALG00010011400 | BPHL | 380 | 431 | 330 | 1.31 | 1.39E-03 |
| ENSGALG00010011426 | NQO2 | 475 | 410 | 541 | 0.757 | 4.93E-02 |
| ENSGALG00010011429 | UBAP2 | 2,852 | 2,523 | 3,181 | 0.793 | 8.71E-04 |
| ENSGALG00010011433 | RNASE6 | 290 | 222 | 359 | 0.616 | 2.18E-02 |
| ENSGALG00010011434 | NPL | 368 | 321 | 415 | 0.772 | 1.27E-02 |
| ENSGALG00010011441 | CD99L2 | 8,237 | 7,554 | 8,921 | 0.847 | 1.31E-02 |
| ENSGALG00010011449 | DUSP22 | 1,389 | 1,225 | 1,554 | 0.788 | 3.95E-02 |
| ENSGALG00010011450 | PTDSS1 | 891 | 812 | 969 | 0.837 | 4.01E-04 |
| ENSGALG00010011452 | KITLG | 364 | 424 | 304 | 1.393 | 1.12E-04 |
| ENSGALG00010011458 | TPGS2 | 457 | 413 | 502 | 0.825 | 4.94E-03 |
| ENSGALG00010011465 | PPP2R2C | 55 | 64 | 46 | 1.407 | 3.07E-02 |
| ENSGALG00010011470 | TAS2R7 | 5 | 3 | 8 | 0.412 | 1.16E-02 |
| ENSGALG00010011471 | NMNAT2 | 8 | 6 | 11 | 0.502 | 8.63E-03 |
| ENSGALG00010011509 | EXOC2 | 1,204 | 1,294 | 1,114 | 1.162 | 9.13E-03 |
| ENSGALG00010011549 | KIAA0232 | 3,683 | 4,098 | 3,268 | 1.254 | 2.63E-02 |
| ENSGALG00010011551 | GMDS | 422 | 349 | 495 | 0.704 | 4.15E-05 |
| ENSGALG00010011559 | MTX3 | 575 | 624 | 526 | 1.188 | 1.33E-03 |
| ENSGALG00010011573 | CMAS | 683 | 625 | 741 | 0.844 | 4.80E-02 |
| ENSGALG00010011575 |  | 17 | 12 | 22 | 0.561 | 4.00E-02 |
| ENSGALG00010011619 | PCDH1 | 259 | 297 | 221 | 1.339 | 1.61E-04 |
| ENSGALG00010011634 | AVD | 577 | 275 | 879 | 0.313 | 1.14E-04 |
| ENSGALG00010011635 | PCDH12 | 162 | 179 | 145 | 1.232 | 3.98E-02 |
| ENSGALG00010011636 | GRIK1 | 117 | 154 | 80 | 1.919 | 1.74E-06 |
| ENSGALG00010011651 | SERPINB6 | 918 | 808 | 1,028 | 0.786 | 1.41E-03 |
| ENSGALG00010011682 | USP15 | 5,513 | 5,945 | 5,080 | 1.171 | 2.05E-02 |
| ENSGALG00010011735 |  | 59 | 29 | 89 | 0.33 | 1.62E-04 |
| ENSGALG00010011752 | RNF2 | 220 | 183 | 257 | 0.713 | 3.58E-03 |
| ENSGALG00010011759 | CREB3 | 976 | 883 | 1,069 | 0.826 | 5.05E-05 |
| ENSGALG00010011772 | GRHL2 | 13 | 8 | 17 | 0.469 | 1.35E-02 |
| ENSGALG00010011792 | HDHD5 | 2,328 | 2,553 | 2,104 | 1.214 | 6.38E-03 |
| ENSGALG00010011793 | TLN1 | 4,337 | 3,547 | 5,126 | 0.692 | 5.29E-04 |
| ENSGALG00010011814 |  | 286 | 457 | 115 | 3.96 | 1.57E-10 |
| ENSGALG00010011839 | CBS | 211 | 259 | 164 | 1.577 | 3.20E-02 |
| ENSGALG00010011859 | NTN4 | 198 | 158 | 237 | 0.669 | 8.14E-03 |
| ENSGALG00010011874 | KCNS2 | 5 | 2 | 9 | 0.23 | 7.26E-03 |
| ENSGALG00010011875 | PSD2 | 29 | 33 | 25 | 1.298 | 4.76E-02 |
| ENSGALG00010011885 | NCALD | 566 | 459 | 672 | 0.682 | 4.61E-02 |
| ENSGALG00010011887 | PGM5 | 5,582 | 4,922 | 6,243 | 0.788 | 7.93E-03 |
| ENSGALG00010011896 | SIK1 | 5,360 | 6,081 | 4,639 | 1.311 | 3.84E-05 |
| ENSGALG00010011900 |  | 3 | 4 | 1 | 4.484 | 4.11E-02 |
| ENSGALG00010011903 | LTA4H | 2,035 | 1,657 | 2,413 | 0.687 | 5.90E-03 |
| ENSGALG00010011911 | NUDT2 | 252 | 232 | 273 | 0.845 | 4.98E-02 |
| ENSGALG00010011926 | DYRK2 | 5,474 | 6,012 | 4,937 | 1.218 | 9.77E-04 |
| ENSGALG00010011941 |  | 57 | 65 | 50 | 1.304 | 1.97E-02 |
| ENSGALG00010011962 | CPSF6 | 902 | 943 | 861 | 1.093 | 2.18E-02 |
| ENSGALG00010011965 | ATIC | 2,137 | 1,696 | 2,578 | 0.658 | 4.88E-05 |
| ENSGALG00010011968 |  | 419 | 483 | 354 | 1.368 | 1.53E-02 |
| ENSGALG00010011976 | TNFRSF11A | 778 | 688 | 868 | 0.792 | 2.52E-02 |
| ENSGALG00010011997 | DENND5B | 178 | 126 | 231 | 0.543 | 6.00E-05 |
| ENSGALG00010012001 | EEIG2 | 1,901 | 1,263 | 2,540 | 0.497 | 2.54E-04 |
| ENSGALG00010012011 | HOOK3 | 1,833 | 2,002 | 1,665 | 1.203 | 1.15E-02 |
| ENSGALG00010012017 | N4BP3 | 110 | 96 | 124 | 0.783 | 1.94E-02 |
| ENSGALG00010012024 | UBR5 | 2,966 | 3,170 | 2,762 | 1.147 | 3.54E-02 |
| ENSGALG00010012026 | SYT1 | 9 | 6 | 12 | 0.495 | 3.20E-02 |
| ENSGALG00010012034 | ARHGEF9 | 510 | 577 | 444 | 1.298 | 7.85E-04 |
| ENSGALG00010012043 | NUP50 | 1,811 | 1,901 | 1,721 | 1.106 | 1.37E-02 |
| ENSGALG00010012046 | LOXHD1 | 87 | 110 | 63 | 1.748 | 3.82E-05 |
| ENSGALG00010012047 | LMAN2 | 849 | 755 | 944 | 0.799 | 2.36E-03 |
| ENSGALG00010012053 |  | 55 | 23 | 87 | 0.263 | 8.58E-08 |
| ENSGALG00010012062 | HMGA2 | 100 | 77 | 122 | 0.636 | 2.04E-04 |
| ENSGALG00010012064 | RGS14 | 326 | 213 | 438 | 0.486 | 1.43E-06 |
| ENSGALG00010012074 | KIAA0930 | 806 | 657 | 955 | 0.688 | 1.97E-03 |
| ENSGALG00010012075 | PIGN | 201 | 185 | 218 | 0.849 | 2.08E-02 |
| ENSGALG00010012078 | TMBIM4 | 777 | 725 | 829 | 0.875 | 1.08E-03 |
| ENSGALG00010012083 | MGP | 12,727 | 11,085 | 14,368 | 0.772 | 4.83E-02 |
| ENSGALG00010012096 |  | 143 | 160 | 127 | 1.258 | 4.73E-03 |
| ENSGALG00010012097 | GART | 1,257 | 1,591 | 923 | 1.726 | 5.52E-07 |
| ENSGALG00010012105 | DONSON | 43 | 37 | 50 | 0.736 | 4.93E-02 |
| ENSGALG00010012107 | CLCC1 | 1,036 | 834 | 1,238 | 0.674 | 3.02E-03 |
| ENSGALG00010012113 | WDR47 | 463 | 411 | 515 | 0.796 | 3.84E-03 |
| ENSGALG00010012121 | SREK1 | 1,555 | 1,650 | 1,460 | 1.131 | 3.93E-02 |
| ENSGALG00010012123 | RASL11AL | 146 | 118 | 173 | 0.681 | 3.26E-02 |
| ENSGALG00010012125 | RNF152 | 363 | 448 | 279 | 1.606 | 7.39E-05 |
| ENSGALG00010012129 | CRYZL1 | 340 | 296 | 384 | 0.771 | 2.39E-03 |
| ENSGALG00010012140 | GLOD5 | 2 | 1 | 4 | 0.297 | 1.38E-02 |
| ENSGALG00010012141 |  | 643 | 691 | 594 | 1.164 | 4.90E-02 |
| ENSGALG00010012147 | ARFGAP3 | 951 | 886 | 1,016 | 0.873 | 3.64E-03 |
| ENSGALG00010012149 | SFXN1 | 411 | 344 | 479 | 0.716 | 6.95E-03 |
| ENSGALG00010012163 | PDP1 | 3,415 | 4,015 | 2,814 | 1.427 | 1.62E-02 |
| ENSGALG00010012191 | BEST3 | 685 | 622 | 748 | 0.832 | 1.56E-02 |
| ENSGALG00010012212 | RAD54B | 61 | 69 | 52 | 1.327 | 2.27E-02 |
| ENSGALG00010012260 | MBD2 | 66 | 57 | 75 | 0.759 | 4.89E-02 |
| ENSGALG00010012297 | ABLIM3 | 264 | 162 | 366 | 0.44 | 7.35E-08 |
| ENSGALG00010012303 | CA9 | 651 | 868 | 435 | 1.999 | 1.04E-07 |
| *ENSGALG00010012307* |  | *21* | *13* | *29* | *0.457* | *1.23E-04* |
| ENSGALG00010012315 | ATP6V1E1 | 2,931 | 2,685 | 3,176 | 0.845 | 1.54E-02 |
| ENSGALG00010012325 | MLPH | 6 | 2 | 11 | 0.205 | 3.21E-04 |
| ENSGALG00010012326 | SGTB | 534 | 489 | 579 | 0.843 | 2.05E-03 |
| ENSGALG00010012341 | GOPC | 1,162 | 1,091 | 1,234 | 0.885 | 2.69E-03 |
| ENSGALG00010012371 | ATP6V1G3 | 61 | 101 | 21 | 4.785 | 1.11E-09 |
| ENSGALG00010012386 |  | 2 | 3 | 0 | 8.415 | 1.91E-03 |
| ENSGALG00010012406 | PROB1 | 2,724 | 3,046 | 2,403 | 1.268 | 3.28E-02 |
| ENSGALG00010012410 | CACNA1I | 6 | 8 | 3 | 2.323 | 4.79E-02 |
| ENSGALG00010012420 |  | 36 | 42 | 31 | 1.325 | 3.04E-02 |
| ENSGALG00010012423 |  | 4 | 3 | 5 | 0.453 | 3.57E-02 |
| ENSGALG00010012431 | LAMP2 | 4,590 | 4,276 | 4,904 | 0.872 | 2.11E-02 |
| ENSGALG00010012436 |  | 4,249 | 3,607 | 4,891 | 0.737 | 2.30E-03 |
| ENSGALG00010012466 | PDSS2 | 2,620 | 2,870 | 2,369 | 1.212 | 4.56E-03 |
| ENSGALG00010012483 | SLC7A7 | 237 | 276 | 198 | 1.393 | 5.54E-03 |
| ENSGALG00010012508 | MDM1 | 376 | 400 | 351 | 1.136 | 3.93E-02 |
| ENSGALG00010012511 | OXCT1 | 13,163 | 14,390 | 11,935 | 1.206 | 4.80E-02 |
| ENSGALG00010012522 |  | 4 | 2 | 7 | 0.344 | 2.27E-02 |
| ENSGALG00010012538 | RAP1B | 2,965 | 2,657 | 3,272 | 0.812 | 2.14E-06 |
| ENSGALG00010012586 | CDC73 | 2,542 | 2,782 | 2,302 | 1.209 | 7.45E-03 |
| ENSGALG00010012587 | MBLAC2 | 312 | 367 | 256 | 1.435 | 7.39E-04 |
| ENSGALG00010012601 | TUBA8A | 3,886 | 4,378 | 3,393 | 1.29 | 9.95E-03 |
| ENSGALG00010012618 | B3GALT2 | 26 | 18 | 34 | 0.543 | 3.38E-03 |
| ENSGALG00010012624 | PDGFB | 192 | 209 | 175 | 1.196 | 1.88E-02 |
| ENSGALG00010012629 | NUS1 | 874 | 783 | 965 | 0.812 | 3.45E-02 |
| ENSGALG00010012640 | PRRC1 | 1,443 | 1,519 | 1,368 | 1.111 | 4.66E-03 |
| ENSGALG00010012654 | TENT5B | 1,660 | 1,896 | 1,423 | 1.333 | 4.07E-03 |
| ENSGALG00010012662 | SLC35E3 | 762 | 683 | 841 | 0.811 | 2.16E-04 |
| ENSGALG00010012667 | SCAMP1 | 934 | 877 | 991 | 0.884 | 4.15E-02 |
| ENSGALG00010012676 |  | 3 | 1 | 6 | 0.095 | 7.49E-03 |
| ENSGALG00010012696 | DNAL4 | 180 | 164 | 196 | 0.833 | 1.79E-02 |
| ENSGALG00010012699 | RNF128 | 407 | 341 | 473 | 0.722 | 3.07E-03 |
| ENSGALG00010012705 | CPM | 1,078 | 897 | 1,260 | 0.712 | 7.99E-04 |
| ENSGALG00010012711 | ELL2 | 229 | 244 | 214 | 1.143 | 2.77E-02 |
| ENSGALG00010012714 | RGS2 | 592 | 493 | 692 | 0.711 | 1.22E-02 |
| ENSGALG00010012726 | IL7R | 80 | 64 | 95 | 0.676 | 3.17E-03 |
| ENSGALG00010012730 | CAP2 | 20,024 | 18,648 | 21,401 | 0.871 | 3.40E-02 |
| ENSGALG00010012744 | ARL4C | 210 | 152 | 268 | 0.568 | 3.53E-02 |
| ENSGALG00010012750 | CAPSL | 5 | 2 | 9 | 0.201 | 4.14E-03 |
| ENSGALG00010012751 | AP3B1 | 1,719 | 1,609 | 1,829 | 0.879 | 8.51E-03 |
| ENSGALG00010012770 |  | 5 | 7 | 3 | 2.398 | 4.80E-02 |
| ENSGALG00010012778 | TBC1D15 | 1,088 | 997 | 1,178 | 0.846 | 8.19E-05 |
| ENSGALG00010012791 | CFAP418 | 189 | 208 | 169 | 1.229 | 5.00E-03 |
| ENSGALG00010012819 | RANGAP1 | 1,554 | 1,478 | 1,630 | 0.907 | 4.11E-02 |
| ENSGALG00010012832 | CHADL | 28 | 37 | 19 | 1.925 | 9.07E-03 |
| ENSGALG00010012839 | TGFBI | 1,622 | 1,396 | 1,847 | 0.756 | 5.28E-03 |
| ENSGALG00010012848 | SERINC1 | 11,921 | 10,669 | 13,172 | 0.81 | 3.71E-03 |
| ENSGALG00010012858 |  | 56 | 68 | 45 | 1.507 | 9.05E-06 |
| ENSGALG00010012867 | A4GALT | 10,044 | 8,286 | 11,803 | 0.702 | 2.85E-08 |
| ENSGALG00010012875 | HSF2 | 1,430 | 1,117 | 1,743 | 0.641 | 2.90E-03 |
| ENSGALG00010012879 | RCAN1 | 1,910 | 1,669 | 2,151 | 0.776 | 3.82E-02 |
| ENSGALG00010012880 | CLK4 | 959 | 1,038 | 880 | 1.181 | 3.10E-02 |
| ENSGALG00010012894 | CLIC6 | 17 | 10 | 24 | 0.404 | 5.96E-04 |
| ENSGALG00010012896 | STK38L | 921 | 981 | 861 | 1.14 | 2.39E-02 |
| ENSGALG00010012899 |  | 408 | 358 | 458 | 0.782 | 1.66E-02 |
| ENSGALG00010012900 | RBX1 | 1,275 | 1,082 | 1,468 | 0.737 | 3.47E-02 |
| ENSGALG00010012903 | RGMB | 612 | 667 | 556 | 1.201 | 2.54E-03 |
| ENSGALG00010012908 | STRAP | 2,444 | 2,299 | 2,588 | 0.888 | 2.80E-02 |
| ENSGALG00010012913 | EGFLAM | 91 | 109 | 74 | 1.466 | 1.09E-04 |
| ENSGALG00010012917 | RUNX1 | 378 | 286 | 470 | 0.608 | 4.06E-02 |
| ENSGALG00010012923 | MLANA | 6 | 1 | 10 | 0.13 | 2.15E-03 |
| ENSGALG00010012924 | GLIPR1 | 13 | 8 | 17 | 0.486 | 6.17E-03 |
| ENSGALG00010012945 | CLINT1 | 2,702 | 2,838 | 2,567 | 1.105 | 3.27E-03 |
| ENSGALG00010012966 | GLIPR1L | 143 | 110 | 175 | 0.628 | 3.26E-02 |
| ENSGALG00010012976 | SLC9A2 | 214 | 174 | 255 | 0.684 | 5.28E-03 |
| ENSGALG00010012988 | RPL18A | 6,750 | 5,767 | 7,732 | 0.746 | 1.53E-02 |
| ENSGALG00010012994 |  | 2 | 1 | 4 | 0.168 | 2.91E-02 |
| ENSGALG00010012995 | SERINC2 | 464 | 371 | 556 | 0.668 | 2.81E-02 |
| ENSGALG00010013012 | TMEM182 | 15,401 | 13,268 | 17,535 | 0.757 | 1.02E-05 |
| ENSGALG00010013013 | DCSTAMP | 13 | 8 | 17 | 0.478 | 4.56E-02 |
| ENSGALG00010013032 | BAIAP2L2 | 57 | 39 | 75 | 0.52 | 1.15E-03 |
| ENSGALG00010013042 | SLC25A48 | 14 | 19 | 9 | 2.049 | 1.18E-02 |
| ENSGALG00010013046 | SLC12A5 | 25 | 15 | 34 | 0.445 | 1.83E-02 |
| ENSGALG00010013050 |  | 3 | 5 | 1 | 8.038 | 1.62E-04 |
| ENSGALG00010013065 | FKTN | 1,049 | 782 | 1,316 | 0.594 | 1.30E-05 |
| ENSGALG00010013082 | CHAF1B | 358 | 388 | 329 | 1.175 | 2.10E-02 |
| ENSGALG00010013084 | SGSM3 | 335 | 357 | 314 | 1.136 | 3.83E-02 |
| ENSGALG00010013085 | LYSMD3 | 367 | 389 | 345 | 1.136 | 4.10E-03 |
| ENSGALG00010013086 | CXCL14 | 245 | 345 | 145 | 2.374 | 1.54E-05 |
| ENSGALG00010013123 | PPFIBP1 | 4,969 | 4,373 | 5,565 | 0.786 | 1.62E-03 |
| ENSGALG00010013146 | TNRC6B | 927 | 1,047 | 808 | 1.296 | 2.42E-02 |
| ENSGALG00010013149 | MCTP1 | 39 | 33 | 46 | 0.715 | 3.77E-02 |
| ENSGALG00010013165 | NUDC | 3,380 | 3,197 | 3,564 | 0.898 | 3.69E-03 |
| ENSGALG00010013172 | TNFSF13B | 41 | 33 | 50 | 0.655 | 4.25E-02 |
| ENSGALG00010013177 | OXR1 | 1,360 | 1,459 | 1,261 | 1.158 | 2.91E-02 |
| ENSGALG00010013179 | TBC1D30 | 76 | 88 | 65 | 1.336 | 2.37E-02 |
| ENSGALG00010013185 |  | 2 | 1 | 3 | 0.19 | 3.86E-02 |
| ENSGALG00010013186 | DDX46 | 2,053 | 2,230 | 1,875 | 1.19 | 7.14E-03 |
| ENSGALG00010013192 | B4GALT5 | 997 | 875 | 1,119 | 0.781 | 2.46E-03 |
| ENSGALG00010013209 |  | 581 | 663 | 500 | 1.326 | 2.86E-02 |
| ENSGALG00010013224 | ATP6V0A4 | 8 | 10 | 5 | 1.907 | 2.43E-02 |
| ENSGALG00010013232 | FHL2 | 216 | 300 | 132 | 2.273 | 2.02E-03 |
| ENSGALG00010013237 | SVOPL | 4 | 5 | 2 | 2.108 | 3.28E-02 |
| ENSGALG00010013250 | E2F5 | 266 | 285 | 247 | 1.149 | 2.42E-02 |
| ENSGALG00010013251 | MTA1 | 861 | 904 | 818 | 1.107 | 4.81E-02 |
| ENSGALG00010013261 | JADE2 | 473 | 536 | 409 | 1.312 | 8.01E-03 |
| ENSGALG00010013264 | DNAJC21 | 1,364 | 1,478 | 1,250 | 1.183 | 1.79E-02 |
| ENSGALG00010013268 | PTPRR | 63 | 76 | 50 | 1.521 | 1.25E-03 |
| ENSGALG00010013284 | MANEAL | 37 | 26 | 49 | 0.515 | 4.33E-06 |
| ENSGALG00010013289 | TM9SF4 | 893 | 819 | 966 | 0.848 | 8.33E-05 |
| ENSGALG00010013296 | AGXT2 | 51 | 74 | 28 | 2.594 | 1.01E-06 |
| ENSGALG00010013301 | PARVB | 6,140 | 5,170 | 7,109 | 0.727 | 1.92E-03 |
| ENSGALG00010013308 | NMU | 7 | 4 | 11 | 0.357 | 9.92E-03 |
| ENSGALG00010013316 | KDM7A | 1,914 | 2,150 | 1,678 | 1.282 | 2.50E-03 |
| ENSGALG00010013320 | CALCRL | 603 | 662 | 544 | 1.216 | 2.42E-02 |
| ENSGALG00010013325 | CA2 | 11,609 | 15,031 | 8,188 | 1.836 | 1.58E-04 |
| ENSGALG00010013328 | SLC37A3 | 560 | 603 | 518 | 1.166 | 7.32E-03 |
| ENSGALG00010013330 | ERG | 255 | 275 | 236 | 1.168 | 4.69E-02 |
| ENSGALG00010013337 | RAB19 | 689 | 875 | 503 | 1.742 | 8.25E-08 |
| ENSGALG00010013339 | ATP6V0D2 | 60 | 35 | 85 | 0.407 | 3.23E-02 |
| ENSGALG00010013351 | EDAR | 19 | 11 | 26 | 0.409 | 3.53E-03 |
| ENSGALG00010013354 | WWP1 | 9,295 | 10,462 | 8,129 | 1.287 | 1.75E-03 |
| ENSGALG00010013356 | AMER1 | 803 | 877 | 729 | 1.203 | 4.45E-02 |
| ENSGALG00010013360 | PRR5 | 543 | 456 | 630 | 0.723 | 5.86E-03 |
| ENSGALG00010013364 | OTOGL | 35 | 25 | 45 | 0.551 | 3.80E-02 |
| ENSGALG00010013376 | ARHGAP8 | 33 | 38 | 29 | 1.33 | 1.43E-02 |
| ENSGALG00010013389 | NAP1L1 | 4,442 | 3,925 | 4,960 | 0.791 | 5.67E-04 |
| ENSGALG00010013399 | FGF1 | 62 | 40 | 84 | 0.478 | 9.00E-10 |
| ENSGALG00010013405 | RPS14 | 8,698 | 7,630 | 9,766 | 0.781 | 2.76E-02 |
| ENSGALG00010013414 | LAPTM5 | 1,239 | 1,009 | 1,469 | 0.687 | 4.14E-02 |
| ENSGALG00010013423 | ZNF804A | 3 | 5 | 2 | 2.817 | 1.24E-02 |
| ENSGALG00010013428 | BPGM | 77,398 | 86,649 | 68,146 | 1.272 | 2.31E-02 |
| ENSGALG00010013431 | TMEM175 | 756 | 667 | 844 | 0.791 | 3.41E-08 |
| ENSGALG00010013440 | EEA1 | 1,341 | 1,226 | 1,457 | 0.841 | 3.86E-02 |
| ENSGALG00010013445 |  | 5 | 7 | 3 | 2.297 | 9.75E-03 |
| ENSGALG00010013458 | LAMA4 | 3,524 | 3,778 | 3,270 | 1.155 | 4.15E-02 |
| ENSGALG00010013468 | DEPDC1B | 39 | 45 | 33 | 1.336 | 2.74E-02 |
| ENSGALG00010013470 | RPL31 | 5,530 | 4,922 | 6,139 | 0.802 | 3.53E-02 |
| ENSGALG00010013477 | RFC1 | 1,009 | 1,077 | 941 | 1.145 | 1.28E-02 |
| ENSGALG00010013484 | TCOF1 | 1,134 | 1,183 | 1,085 | 1.091 | 3.29E-02 |
| ENSGALG00010013486 | PLPP6 | 354 | 404 | 303 | 1.332 | 2.39E-02 |
| ENSGALG00010013493 |  | 7,850 | 7,025 | 8,675 | 0.81 | 2.30E-02 |
| ENSGALG00010013497 | FAM172A | 621 | 572 | 671 | 0.851 | 3.51E-03 |
| ENSGALG00010013498 | KDR | 509 | 572 | 447 | 1.277 | 3.44E-03 |
| ENSGALG00010013501 | AKR1B10 | 278 | 224 | 332 | 0.674 | 4.99E-02 |
| ENSGALG00010013511 | FAM78B | 839 | 943 | 735 | 1.282 | 8.44E-03 |
| ENSGALG00010013527 | ADARB1 | 276 | 304 | 248 | 1.228 | 1.21E-02 |
| ENSGALG00010013544 |  | 43 | 22 | 65 | 0.337 | 4.33E-03 |
| ENSGALG00010013550 | KLHL5 | 547 | 494 | 599 | 0.825 | 3.62E-03 |
| ENSGALG00010013563 | FKBP1A | 3,034 | 2,680 | 3,388 | 0.791 | 3.65E-02 |
| *ENSGALG00010013569* |  | *92* | *21* | *162* | *0.13* | *1.31E-09* |
| ENSGALG00010013580 | RANBP3L | 21 | 26 | 16 | 1.549 | 1.65E-02 |
| ENSGALG00010013582 | MAP4K4 | 1,181 | 1,000 | 1,362 | 0.734 | 1.23E-02 |
| ENSGALG00010013598 | PAQR7 | 271 | 219 | 324 | 0.678 | 4.91E-02 |
| ENSGALG00010013663 | OPA1 | 2,161 | 2,319 | 2,003 | 1.158 | 7.11E-03 |
| ENSGALG00010013669 | NPR3 | 205 | 254 | 155 | 1.645 | 2.33E-02 |
| ENSGALG00010013675 | CHRNA6 | 8 | 11 | 5 | 2.222 | 2.83E-02 |
| ENSGALG00010013751 | FUT10 | 155 | 140 | 170 | 0.826 | 4.79E-02 |
| ENSGALG00010013761 |  | 17 | 27 | 8 | 3.314 | 1.59E-02 |
| ENSGALG00010013765 | PRLL | 20 | 4 | 35 | 0.115 | 1.73E-03 |
| ENSGALG00010013767 | PGK2 | 243,662 | 273,148 | 214,177 | 1.275 | 3.43E-02 |
| ENSGALG00010013772 | KIAA0319L | 6,811 | 7,207 | 6,415 | 1.124 | 4.95E-02 |
| ENSGALG00010013777 | CPLANE1 | 467 | 513 | 422 | 1.217 | 9.37E-04 |
| *ENSGALG00010013778* |  | *46* | *5* | *86* | *0.064* | *5.24E-06* |
| ENSGALG00010013787 | ZCCHC10 | 493 | 455 | 530 | 0.857 | 1.01E-02 |
| ENSGALG00010013805 | HSPA4 | 9,829 | 9,132 | 10,525 | 0.868 | 2.94E-02 |
| ENSGALG00010013815 | SRXN1 | 82 | 61 | 103 | 0.591 | 5.30E-03 |
| ENSGALG00010013872 | GLS2 | 7,466 | 8,774 | 6,157 | 1.425 | 1.77E-02 |
| ENSGALG00010013890 | SFPQ | 2,061 | 2,152 | 1,969 | 1.093 | 1.03E-02 |
| ENSGALG00010013896 |  | 4 | 7 | 1 | 5.961 | 7.33E-04 |
| ENSGALG00010013914 | NRG1 | 84 | 71 | 97 | 0.733 | 6.45E-03 |
| ENSGALG00010013917 | TRPC5 | 2 | 1 | 3 | 0.203 | 8.37E-03 |
| ENSGALG00010013929 |  | 13 | 9 | 17 | 0.514 | 3.48E-02 |
| ENSGALG00010013930 | SLC22A16 | 2,271 | 2,585 | 1,956 | 1.321 | 4.54E-02 |
| ENSGALG00010013934 | STAT1 | 1,906 | 1,527 | 2,285 | 0.668 | 1.48E-03 |
| ENSGALG00010013940 | UPF3A | 15 | 9 | 21 | 0.398 | 3.38E-03 |
| ENSGALG00010013942 | CACNA2D4 | 10 | 8 | 13 | 0.585 | 1.55E-02 |
| ENSGALG00010013958 | TUSC3 | 241 | 181 | 302 | 0.598 | 4.14E-04 |
| ENSGALG00010013981 | RLF | 837 | 925 | 748 | 1.237 | 1.70E-03 |
| ENSGALG00010013989 |  | 41 | 34 | 48 | 0.708 | 4.46E-02 |
| ENSGALG00010014000 | STAT4 | 23 | 16 | 30 | 0.545 | 6.61E-03 |
| ENSGALG00010014014 | PARP8 | 161 | 125 | 197 | 0.633 | 5.45E-03 |
| ENSGALG00010014018 | DPYSL3 | 614 | 552 | 677 | 0.814 | 1.64E-02 |
| ENSGALG00010014019 | IRAG2 | 57 | 48 | 67 | 0.708 | 2.36E-02 |
| ENSGALG00010014030 | WASF1 | 25 | 28 | 22 | 1.319 | 3.23E-02 |
| ENSGALG00010014032 | PSD3 | 412 | 494 | 331 | 1.495 | 1.60E-03 |
| ENSGALG00010014033 | SLC38A4 | 566 | 437 | 695 | 0.629 | 4.29E-02 |
| ENSGALG00010014038 |  | 100 | 117 | 84 | 1.389 | 4.06E-03 |
| ENSGALG00010014041 | SKIV2L2 | 2,010 | 1,914 | 2,106 | 0.909 | 1.79E-02 |
| ENSGALG00010014045 | GOLT1B | 926 | 786 | 1,065 | 0.738 | 4.94E-06 |
| ENSGALG00010014056 | FMO3 | 455 | 512 | 398 | 1.292 | 8.34E-03 |
| ENSGALG00010014080 | MYO1B | 1,743 | 1,949 | 1,537 | 1.268 | 6.82E-05 |
| ENSGALG00010014082 | HNRNPR | 3,024 | 3,134 | 2,914 | 1.076 | 5.86E-03 |
| ENSGALG00010014087 |  | 183 | 281 | 87 | 3.241 | 4.49E-02 |
| ENSGALG00010014102 | TRIM2 | 2,317 | 2,763 | 1,871 | 1.477 | 3.33E-04 |
| ENSGALG00010014117 |  | 33 | 40 | 26 | 1.499 | 2.08E-02 |
| ENSGALG00010014120 | SOX5 | 48 | 53 | 42 | 1.253 | 4.77E-02 |
| ENSGALG00010014134 | RPAP3 | 1,029 | 1,099 | 959 | 1.147 | 3.67E-02 |
| ENSGALG00010014156 | CD164 | 6,575 | 6,124 | 7,027 | 0.872 | 2.04E-02 |
| ENSGALG00010014164 |  | 57 | 32 | 82 | 0.394 | 1.11E-03 |
| ENSGALG00010014178 | LRTM2 | 49 | 33 | 64 | 0.515 | 8.66E-03 |
| ENSGALG00010014192 | KDM1A | 1,205 | 1,135 | 1,274 | 0.89 | 4.78E-02 |
| ENSGALG00010014197 | SH3GL2 | 23 | 17 | 28 | 0.604 | 2.19E-02 |
| ENSGALG00010014201 |  | 18 | 22 | 15 | 1.489 | 1.47E-02 |
| ENSGALG00010014203 | TMEM38B | 5,089 | 4,060 | 6,119 | 0.663 | 5.64E-06 |
| ENSGALG00010014225 | ZMPSTE24 | 1,048 | 993 | 1,102 | 0.902 | 3.20E-02 |
| *ENSGALG00010014241* |  | *125* | *10* | *240* | *0.041* | *1.32E-05* |
| ENSGALG00010014244 | SESN1 | 2,368 | 2,736 | 2,001 | 1.368 | 1.65E-03 |
| ENSGALG00010014274 | FIRRM | 320 | 376 | 265 | 1.426 | 4.99E-07 |
| ENSGALG00010014275 |  | 6 | 2 | 11 | 0.147 | 9.13E-03 |
| ENSGALG00010014294 | TMPO | 5,137 | 5,672 | 4,601 | 1.233 | 2.40E-02 |
| ENSGALG00010014302 | FOXO3 | 363 | 416 | 310 | 1.339 | 2.07E-02 |
| ENSGALG00010014319 |  | 411 | 344 | 478 | 0.72 | 6.04E-03 |
| ENSGALG00010014324 | COL4A2 | 15,775 | 16,928 | 14,623 | 1.158 | 2.32E-02 |
| ENSGALG00010014325 |  | 301 | 349 | 252 | 1.389 | 1.09E-03 |
| ENSGALG00010014334 | HMGCS1 | 3,558 | 3,794 | 3,321 | 1.142 | 4.28E-02 |
| ENSGALG00010014355 | RAB20 | 276 | 249 | 303 | 0.821 | 3.02E-02 |
| ENSGALG00010014361 | SSPN | 11,106 | 9,354 | 12,858 | 0.728 | 1.08E-04 |
| ENSGALG00010014380 | SNX3 | 12,824 | 10,885 | 14,763 | 0.737 | 2.53E-04 |
| ENSGALG00010014465 | SIMC1 | 1,548 | 1,751 | 1,345 | 1.303 | 1.02E-02 |
| ENSGALG00010014470 | RPS6KA1 | 978 | 608 | 1,348 | 0.451 | 1.31E-09 |
| ENSGALG00010014476 | C5orf34 | 75 | 65 | 85 | 0.76 | 1.66E-02 |
| ENSGALG00010014480 |  | 448 | 539 | 356 | 1.519 | 2.57E-02 |
| ENSGALG00010014484 |  | 361 | 397 | 325 | 1.223 | 1.04E-02 |
| ENSGALG00010014488 |  | 372 | 184 | 560 | 0.329 | 7.56E-04 |
| ENSGALG00010014493 |  | 5 | 6 | 3 | 2.125 | 2.36E-02 |
| ENSGALG00010014494 | KLHL9 | 808 | 896 | 721 | 1.243 | 1.57E-06 |
| ENSGALG00010014505 | CYTH4 | 154 | 132 | 176 | 0.746 | 3.42E-02 |
| ENSGALG00010014537 | GKAP1 | 2,355 | 1,911 | 2,799 | 0.683 | 6.17E-06 |
| ENSGALG00010014579 | NNT | 8,016 | 8,632 | 7,400 | 1.167 | 4.96E-02 |
| ENSGALG00010014585 |  | 1,180 | 1,054 | 1,305 | 0.808 | 2.01E-02 |
| ENSGALG00010014592 | CZH9orf64 | 798 | 737 | 859 | 0.858 | 1.78E-02 |
| ENSGALG00010014614 | GHR | 3,251 | 3,617 | 2,885 | 1.254 | 2.28E-02 |
| ENSGALG00010014632 | NWD2 | 17 | 21 | 12 | 1.81 | 1.39E-02 |
| ENSGALG00010014657 |  | 59 | 44 | 75 | 0.587 | 1.95E-02 |
| ENSGALG00010014680 |  | 62 | 76 | 48 | 1.559 | 1.86E-02 |
| ENSGALG00010014710 | NPEPL1 | 3,684 | 3,946 | 3,421 | 1.154 | 4.59E-02 |
| ENSGALG00010014715 |  | 4 | 2 | 5 | 0.432 | 4.63E-02 |
| ENSGALG00010014717 | ELOVL7 | 138 | 155 | 122 | 1.271 | 6.67E-03 |
| ENSGALG00010014730 |  | 2 | 3 | 1 | 3.601 | 2.87E-02 |
| ENSGALG00010014737 | RMI1 | 776 | 870 | 683 | 1.278 | 1.73E-04 |
| ENSGALG00010014746 |  | 51 | 35 | 66 | 0.53 | 7.73E-04 |
| ENSGALG00010014748 | FGFR1OP2 | 478 | 515 | 442 | 1.163 | 3.70E-03 |
| ENSGALG00010014756 |  | 79 | 105 | 54 | 1.94 | 1.33E-08 |
| ENSGALG00010014769 | CCDC69 | 209 | 140 | 278 | 0.504 | 1.51E-06 |
| ENSGALG00010014771 |  | 56 | 70 | 42 | 1.693 | 3.92E-03 |
| ENSGALG00010014777 | APCDD1L | 103 | 77 | 128 | 0.596 | 1.89E-04 |
| ENSGALG00010014783 |  | 666 | 621 | 711 | 0.875 | 2.39E-02 |
| ENSGALG00010014797 | ZDHHC9 | 1,859 | 1,646 | 2,072 | 0.795 | 4.26E-04 |
| ENSGALG00010014816 | TST | 667 | 536 | 797 | 0.672 | 3.55E-03 |
| ENSGALG00010014821 | C3AR1L | 393 | 290 | 496 | 0.584 | 3.03E-02 |
| ENSGALG00010014824 | ANXA6 | 18,241 | 13,770 | 22,712 | 0.606 | 1.35E-08 |
| ENSGALG00010014829 |  | 66 | 49 | 83 | 0.589 | 1.33E-02 |
| ENSGALG00010014840 | NCF4 | 116 | 96 | 135 | 0.706 | 4.35E-02 |
| ENSGALG00010014867 | TP63 | 513 | 375 | 651 | 0.576 | 1.44E-03 |
| ENSGALG00010014878 | TMCO1 | 775 | 706 | 845 | 0.836 | 5.55E-03 |
| ENSGALG00010014898 |  | 5 | 3 | 7 | 0.491 | 3.47E-02 |
| ENSGALG00010014903 | MCF2L | 559 | 635 | 484 | 1.312 | 3.95E-03 |
| ENSGALG00010014912 | PDCD1 | 52 | 39 | 65 | 0.6 | 4.30E-02 |
| ENSGALG00010014925 |  | 29 | 17 | 41 | 0.419 | 2.14E-02 |
| ENSGALG00010014929 | FHL1 | 9,116 | 4,824 | 13,408 | 0.36 | 7.44E-07 |
| ENSGALG00010014942 | PMEPA1 | 272 | 240 | 303 | 0.792 | 3.05E-02 |
| ENSGALG00010014953 |  | 17 | 13 | 22 | 0.562 | 2.80E-02 |
| ENSGALG00010014963 | KMO | 115 | 30 | 200 | 0.151 | 3.54E-04 |
| ENSGALG00010014984 | LPP | 329 | 403 | 255 | 1.579 | 1.37E-02 |
| ENSGALG00010014991 |  | 45 | 35 | 55 | 0.645 | 2.27E-03 |
| ENSGALG00010015002 | ADAM19 | 778 | 610 | 947 | 0.645 | 1.63E-02 |
| ENSGALG00010015010 |  | 376 | 298 | 454 | 0.654 | 1.04E-05 |
| ENSGALG00010015016 | ARGLU1 | 3,238 | 3,404 | 3,071 | 1.109 | 1.44E-02 |
| ENSGALG00010015017 | FAM184A | 74 | 64 | 84 | 0.757 | 1.55E-02 |
| ENSGALG00010015030 |  | 663 | 947 | 379 | 2.5 | 1.67E-11 |
| ENSGALG00010015039 | FAM114A1 | 759 | 687 | 830 | 0.827 | 1.03E-02 |
| ENSGALG00010015043 | COQ10B | 7,083 | 7,704 | 6,461 | 1.193 | 3.67E-02 |
| ENSGALG00010015047 | NPY2R | 119 | 88 | 149 | 0.594 | 4.99E-05 |
| ENSGALG00010015061 | MMP9 | 49 | 25 | 73 | 0.348 | 1.51E-02 |
| ENSGALG00010015068 | ESM1 | 61 | 71 | 50 | 1.44 | 4.27E-02 |
| ENSGALG00010015076 | ITK | 10 | 7 | 14 | 0.489 | 1.16E-02 |
| ENSGALG00010015095 | RBFOX2 | 5,603 | 6,492 | 4,715 | 1.377 | 1.23E-07 |
| ENSGALG00010015109 | PPT1 | 932 | 767 | 1,096 | 0.699 | 7.35E-08 |
| ENSGALG00010015119 | TLR1A | 39 | 31 | 48 | 0.639 | 2.33E-02 |
| ENSGALG00010015124 | UXS1 | 1,181 | 1,067 | 1,295 | 0.824 | 2.64E-02 |
| ENSGALG00010015128 | OCRL | 1,342 | 1,471 | 1,213 | 1.214 | 1.40E-03 |
| ENSGALG00010015134 | PLN | 453 | 269 | 637 | 0.422 | 1.67E-02 |
| ENSGALG00010015152 | BOLL | 204 | 231 | 177 | 1.305 | 1.02E-02 |
| ENSGALG00010015155 | HMOX1 | 762 | 630 | 894 | 0.704 | 3.01E-02 |
| ENSGALG00010015161 | GFPT2 | 315 | 270 | 360 | 0.75 | 4.99E-02 |
| ENSGALG00010015166 | YTHDF2 | 974 | 1,042 | 907 | 1.15 | 1.71E-02 |
| ENSGALG00010015172 | PPP4R3A | 1,299 | 1,367 | 1,230 | 1.112 | 2.34E-02 |
| ENSGALG00010015181 | VPS13A | 5,871 | 6,532 | 5,210 | 1.254 | 4.80E-02 |
| ENSGALG00010015191 | TOM1 | 27,039 | 21,225 | 32,852 | 0.646 | 4.30E-06 |
| ENSGALG00010015192 |  | 41 | 56 | 25 | 2.202 | 5.86E-04 |
| ENSGALG00010015199 |  | 50 | 43 | 57 | 0.743 | 2.37E-02 |
| ENSGALG00010015212 | GNAQ | 860 | 918 | 802 | 1.144 | 9.42E-03 |
| ENSGALG00010015218 | PLCL1 | 57 | 67 | 46 | 1.462 | 1.16E-02 |
| ENSGALG00010015327 | LARGE1 | 1,264 | 1,100 | 1,428 | 0.771 | 1.21E-02 |
| ENSGALG00010015370 |  | 7,768 | 6,749 | 8,787 | 0.768 | 1.08E-02 |
| ENSGALG00010015410 | HTR2B | 52 | 37 | 66 | 0.564 | 2.92E-02 |
| ENSGALG00010015422 | DGKK | 4 | 5 | 2 | 2.06 | 4.54E-02 |
| ENSGALG00010015443 | PAK3 | 1,950 | 2,138 | 1,763 | 1.213 | 3.20E-02 |
| ENSGALG00010015469 | TMEM33 | 1,116 | 1,164 | 1,067 | 1.09 | 1.23E-02 |
| ENSGALG00010015472 | LGMN | 3,796 | 3,092 | 4,500 | 0.687 | 1.59E-04 |
| ENSGALG00010015518 |  | 11 | 14 | 8 | 1.839 | 1.94E-02 |
| ENSGALG00010015520 | UBE3A | 4,400 | 4,842 | 3,957 | 1.224 | 4.92E-03 |
| ENSGALG00010015567 |  | 57 | 70 | 45 | 1.557 | 1.93E-04 |
| ENSGALG00010015577 |  | 41 | 47 | 35 | 1.336 | 4.81E-02 |
| ENSGALG00010015596 | UCHL1 | 1,682 | 793 | 2,572 | 0.308 | 3.27E-06 |
| ENSGALG00010015606 | TMEM131 | 2,717 | 2,975 | 2,459 | 1.21 | 1.37E-02 |
| ENSGALG00010015610 |  | 5,939 | 4,667 | 7,210 | 0.647 | 1.25E-03 |
| ENSGALG00010015613 | PWP1 | 1,040 | 1,100 | 981 | 1.122 | 3.99E-02 |
| ENSGALG00010015616 | NADK2 | 704 | 756 | 653 | 1.158 | 1.51E-02 |
| ENSGALG00010015624 | ITGA1 | 816 | 900 | 731 | 1.23 | 5.79E-03 |
| ENSGALG00010015628 | UBQLN1 | 2,476 | 2,310 | 2,643 | 0.874 | 1.96E-02 |
| ENSGALG00010015643 |  | 700 | 767 | 633 | 1.212 | 5.43E-03 |
| ENSGALG00010015647 | PDZD2 | 287 | 225 | 350 | 0.643 | 2.45E-03 |
| ENSGALG00010015655 |  | 92 | 106 | 78 | 1.347 | 4.08E-02 |
| ENSGALG00010015673 |  | 27 | 18 | 36 | 0.489 | 3.96E-06 |
| ENSGALG00010015676 | TGM2 | 2,730 | 2,028 | 3,432 | 0.591 | 3.37E-05 |
| ENSGALG00010015695 | ASAH1 | 2,342 | 1,926 | 2,758 | 0.698 | 5.67E-08 |
| *ENSGALG00010015728* |  | *12* | *19* | *4* | *4.44* | *5.16E-03* |
| ENSGALG00010015733 | COL4A6 | 899 | 770 | 1,029 | 0.747 | 2.04E-02 |
| ENSGALG00010015757 |  | 705 | 539 | 870 | 0.619 | 1.80E-03 |
| ENSGALG00010015785 | FGL1A | 169 | 145 | 192 | 0.754 | 1.69E-02 |
| ENSGALG00010015800 | BVES | 4,362 | 3,377 | 5,348 | 0.631 | 9.13E-08 |
| ENSGALG00010015804 | CKB | 1,203 | 1,041 | 1,364 | 0.763 | 4.29E-02 |
| ENSGALG00010015817 |  | 393 | 305 | 480 | 0.636 | 4.01E-04 |
| ENSGALG00010015827 |  | 138 | 122 | 155 | 0.787 | 3.59E-02 |
| ENSGALG00010015829 | SMAD5 | 4,132 | 4,511 | 3,754 | 1.202 | 1.41E-04 |
| ENSGALG00010015831 | LYG2 | 17 | 20 | 14 | 1.519 | 5.00E-02 |
| ENSGALG00010015832 | RAB39B | 933 | 869 | 997 | 0.87 | 2.25E-03 |
| ENSGALG00010015839 | U2SURP | 1,395 | 1,504 | 1,285 | 1.171 | 1.07E-03 |
| ENSGALG00010015840 | MTUS1 | 1,261 | 1,425 | 1,098 | 1.299 | 8.26E-03 |
| ENSGALG00010015843 | RXFP2 | 5 | 3 | 8 | 0.35 | 4.68E-03 |
| ENSGALG00010015846 |  | 7 | 9 | 5 | 1.926 | 2.06E-02 |
| ENSGALG00010015862 | PDGFRL | 905 | 764 | 1,046 | 0.729 | 1.76E-03 |
| ENSGALG00010015863 |  | 8 | 5 | 11 | 0.442 | 1.46E-02 |
| ENSGALG00010015869 | SLC7A2 | 1,177 | 1,368 | 986 | 1.389 | 1.69E-02 |
| ENSGALG00010015887 | SLC9A9 | 407 | 342 | 473 | 0.722 | 1.76E-03 |
| ENSGALG00010015904 | MTMR7 | 422 | 616 | 227 | 2.721 | 2.27E-06 |
| ENSGALG00010015909 | TNNC2 | 172,225 | 145,788 | 198,661 | 0.734 | 5.05E-03 |
| ENSGALG00010015914 | SRRM1 | 2,026 | 2,148 | 1,904 | 1.128 | 2.25E-02 |
| ENSGALG00010015916 | COA8 | 1,451 | 1,713 | 1,190 | 1.441 | 3.70E-03 |
| ENSGALG00010015935 | EPHX1 | 461 | 406 | 516 | 0.788 | 2.35E-02 |
| ENSGALG00010015939 | SUN2 | 1,237 | 1,095 | 1,379 | 0.794 | 1.15E-03 |
| ENSGALG00010015954 | CTSA | 2,549 | 2,131 | 2,966 | 0.718 | 1.83E-02 |
| ENSGALG00010015959 | TRNAU1AP | 818 | 913 | 724 | 1.264 | 1.23E-02 |
| ENSGALG00010015972 | FSTL4 | 214 | 286 | 143 | 2.001 | 4.20E-03 |
| ENSGALG00010015974 | VPS37A | 1,635 | 1,837 | 1,432 | 1.285 | 7.26E-05 |
| ENSGALG00010015984 | PDCL3 | 3,381 | 2,909 | 3,852 | 0.755 | 7.04E-04 |
| ENSGALG00010016004 | XRCC3 | 213 | 239 | 188 | 1.278 | 3.91E-03 |
| ENSGALG00010016006 | PLTP | 395 | 251 | 538 | 0.467 | 3.66E-02 |
| ENSGALG00010016016 | ZDHHC2 | 703 | 505 | 901 | 0.561 | 3.07E-04 |
| ENSGALG00010016021 |  | 12 | 16 | 7 | 2.168 | 6.20E-03 |
| ENSGALG00010016030 | TGM4 | 79 | 36 | 122 | 0.298 | 2.30E-02 |
| ENSGALG00010016040 | RPSAP58 | 22,702 | 19,761 | 25,643 | 0.771 | 9.01E-04 |
| ENSGALG00010016051 |  | 199 | 227 | 171 | 1.324 | 1.26E-02 |
| ENSGALG00010016073 | SAXO1 | 27 | 18 | 36 | 0.509 | 3.44E-02 |
| ENSGALG00010016117 |  | 29 | 34 | 23 | 1.471 | 6.13E-03 |
| ENSGALG00010016167 |  | 3 | 1 | 5 | 0.311 | 3.44E-02 |
| ENSGALG00010016168 | PLIN2 | 4,029 | 3,386 | 4,671 | 0.725 | 9.75E-03 |
| ENSGALG00010016225 | P4HA2 | 1,129 | 852 | 1,405 | 0.607 | 1.70E-19 |
| ENSGALG00010016257 | L3MBTL4 | 153 | 140 | 166 | 0.842 | 3.53E-02 |
| ENSGALG00010016260 | INTS6L | 1,159 | 1,229 | 1,089 | 1.131 | 4.99E-02 |
| ENSGALG00010016308 | RPS6 | 22,301 | 19,710 | 24,892 | 0.792 | 1.91E-03 |
| ENSGALG00010016315 | RPS4Y1 | 13,553 | 11,973 | 15,133 | 0.791 | 1.96E-02 |
| ENSGALG00010016328 | USH2A | 330 | 434 | 225 | 1.927 | 1.41E-03 |
| ENSGALG00010016341 | SLX4 | 619 | 673 | 565 | 1.192 | 9.00E-03 |
| ENSGALG00010016345 | CTSZ | 1,818 | 1,674 | 1,961 | 0.853 | 1.71E-02 |
| ENSGALG00010016362 | NUP62 | 400 | 374 | 426 | 0.874 | 1.75E-02 |
| ENSGALG00010016372 | DNAAF6 | 257 | 188 | 325 | 0.578 | 2.22E-04 |
| ENSGALG00010016373 | SRL | 50,537 | 46,804 | 54,271 | 0.862 | 2.70E-02 |
| ENSGALG00010016382 | UGDH | 1,218 | 1,098 | 1,337 | 0.82 | 1.99E-02 |
| ENSGALG00010016390 |  | 133 | 165 | 102 | 1.613 | 1.40E-03 |
| ENSGALG00010016395 | HHIPL1 | 129 | 107 | 150 | 0.713 | 1.05E-02 |
| ENSGALG00010016400 |  | 18,472 | 20,376 | 16,569 | 1.23 | 4.03E-03 |
| ENSGALG00010016407 | RPL9 | 12,704 | 11,597 | 13,812 | 0.84 | 9.40E-03 |
| ENSGALG00010016410 | TRAK1 | 3,203 | 3,390 | 3,016 | 1.124 | 4.75E-02 |
| ENSGALG00010016412 | TMSB15B | 587 | 476 | 698 | 0.682 | 1.52E-02 |
| ENSGALG00010016413 |  | 8 | 10 | 6 | 1.746 | 1.33E-02 |
| ENSGALG00010016416 | CYP46A1 | 618 | 542 | 695 | 0.78 | 1.33E-03 |
| ENSGALG00010016422 | SHKBP1 | 3,721 | 3,407 | 4,036 | 0.844 | 3.48E-02 |
| ENSGALG00010016451 | USP46 | 1,490 | 1,674 | 1,306 | 1.283 | 2.73E-03 |
| ENSGALG00010016460 | LBR | 1,499 | 1,570 | 1,428 | 1.098 | 1.64E-02 |
| ENSGALG00010016464 | PANK2 | 216 | 200 | 233 | 0.857 | 1.21E-02 |
| ENSGALG00010016466 | TMEM164 | 115 | 133 | 97 | 1.376 | 1.61E-02 |
| ENSGALG00010016469 | MAVS | 1,351 | 1,177 | 1,525 | 0.771 | 5.02E-04 |
| ENSGALG00010016470 | TNIP1 | 2,893 | 2,435 | 3,350 | 0.727 | 7.72E-08 |
| ENSGALG00010016479 |  | 30 | 24 | 36 | 0.662 | 4.44E-02 |
| ENSGALG00010016480 | GPX3 | 7,386 | 6,283 | 8,490 | 0.74 | 2.00E-03 |
| ENSGALG00010016504 | SNRK | 534 | 599 | 469 | 1.278 | 4.70E-04 |
| ENSGALG00010016508 | CREBBP | 1,265 | 1,390 | 1,140 | 1.22 | 7.23E-03 |
| ENSGALG00010016543 | TCEA3 | 2,543 | 2,167 | 2,920 | 0.742 | 6.26E-03 |
| ENSGALG00010016548 | EVL | 956 | 732 | 1,181 | 0.619 | 1.40E-08 |
| ENSGALG00010016551 |  | 126 | 151 | 100 | 1.514 | 2.27E-02 |
| ENSGALG00010016560 | DCUN1D4 | 1,869 | 2,118 | 1,621 | 1.307 | 2.70E-02 |
| ENSGALG00010016566 | CNTLN | 30 | 36 | 23 | 1.557 | 1.28E-02 |
| ENSGALG00010016574 |  | 57 | 67 | 46 | 1.431 | 2.45E-02 |
| ENSGALG00010016578 | GRIK3 | 3 | 2 | 5 | 0.382 | 4.77E-02 |
| ENSGALG00010016584 | EHBP1 | 3,688 | 3,315 | 4,060 | 0.816 | 1.55E-02 |
| ENSGALG00010016588 | ATP11B | 2,415 | 2,581 | 2,250 | 1.147 | 1.96E-02 |
| ENSGALG00010016591 | HOMER2 | 253 | 169 | 337 | 0.502 | 1.38E-09 |
| ENSGALG00010016595 |  | 15 | 6 | 23 | 0.243 | 2.99E-10 |
| ENSGALG00010016596 |  | 41 | 26 | 55 | 0.477 | 5.04E-05 |
| ENSGALG00010016602 | HSPB7 | 1,707 | 832 | 2,581 | 0.322 | 3.78E-10 |
| ENSGALG00010016619 | SLC25A47 | 55 | 41 | 69 | 0.597 | 5.13E-03 |
| ENSGALG00010016627 | SH3RF2 | 12 | 2 | 22 | 0.091 | 2.65E-10 |
| ENSGALG00010016630 |  | 54 | 47 | 61 | 0.776 | 1.54E-02 |
| ENSGALG00010016631 | FAM53A | 2,499 | 2,204 | 2,794 | 0.789 | 1.59E-02 |
| ENSGALG00010016647 | EGR1 | 2,470 | 1,866 | 3,075 | 0.607 | 1.86E-02 |
| ENSGALG00010016654 | SMIM3 | 88 | 78 | 98 | 0.79 | 2.42E-02 |
| ENSGALG00010016673 | LRPAP1 | 1,029 | 925 | 1,132 | 0.817 | 3.39E-03 |
| ENSGALG00010016699 | SYNPO | 25,879 | 22,315 | 29,443 | 0.758 | 6.51E-03 |
| ENSGALG00010016706 | RTN4 | 34,926 | 32,235 | 37,617 | 0.857 | 4.32E-02 |
| ENSGALG00010016709 | DNASE1 | 3 | 0 | 6 | 0.062 | 3.22E-04 |
| ENSGALG00010016732 | ARHGAP26 | 408 | 347 | 468 | 0.742 | 1.32E-06 |
| ENSGALG00010016745 | BEGAIN | 105 | 145 | 64 | 2.262 | 6.01E-06 |
| ENSGALG00010016754 |  | 104 | 87 | 122 | 0.709 | 1.64E-02 |
| ENSGALG00010016760 | DLK1 | 108 | 136 | 79 | 1.75 | 2.17E-03 |
| ENSGALG00010016761 | GPR160 | 797 | 985 | 609 | 1.618 | 2.59E-03 |
| ENSGALG00010016771 |  | 3 | 1 | 5 | 0.182 | 4.94E-03 |
| ENSGALG00010016777 | TNMD | 237 | 188 | 285 | 0.657 | 1.29E-02 |
| ENSGALG00010016783 | CLBA1 | 32 | 23 | 41 | 0.558 | 1.28E-02 |
| ENSGALG00010016787 | PPP2R5C | 1,673 | 1,598 | 1,749 | 0.914 | 4.22E-02 |
| ENSGALG00010016793 | SLC9A1 | 1,438 | 1,251 | 1,626 | 0.77 | 4.32E-03 |
| ENSGALG00010016797 | SEC62 | 1,261 | 1,205 | 1,317 | 0.916 | 3.48E-02 |
| ENSGALG00010016800 | RPL14 | 4,565 | 4,127 | 5,003 | 0.825 | 4.56E-02 |
| ENSGALG00010016804 | CSF1R | 2,851 | 2,352 | 3,351 | 0.702 | 2.36E-02 |
| ENSGALG00010016820 | EIF2AK3 | 309 | 279 | 338 | 0.822 | 1.21E-02 |
| ENSGALG00010016832 | DYNC1H1 | 14,314 | 12,678 | 15,950 | 0.795 | 6.34E-03 |
| ENSGALG00010016850 | GPR83L | 5 | 3 | 6 | 0.436 | 3.40E-02 |
| ENSGALG00010016853 |  | 17 | 13 | 21 | 0.622 | 2.93E-02 |
| ENSGALG00010016862 | TXK | 32 | 26 | 38 | 0.675 | 2.22E-02 |
| ENSGALG00010016877 | UBE2D3 | 5,278 | 4,982 | 5,574 | 0.894 | 3.62E-02 |
| ENSGALG00010016878 | SUCLG1 | 9,941 | 10,915 | 8,966 | 1.218 | 1.79E-02 |
| ENSGALG00010016879 | RNF130 | 667 | 522 | 812 | 0.643 | 9.12E-09 |
| ENSGALG00010016881 | C1QB | 524 | 415 | 633 | 0.655 | 2.28E-02 |
| ENSGALG00010016885 | CDCA4 | 806 | 752 | 860 | 0.873 | 4.77E-02 |
| ENSGALG00010016889 | FUNDC2 | 23,198 | 21,449 | 24,947 | 0.86 | 1.71E-03 |
| ENSGALG00010016890 | UBR7 | 2,864 | 3,031 | 2,697 | 1.125 | 5.94E-03 |
| ENSGALG00010016901 | MECOM | 272 | 319 | 225 | 1.414 | 1.96E-06 |
| ENSGALG00010016904 | EAF1 | 1,752 | 1,942 | 1,562 | 1.243 | 2.17E-05 |
| ENSGALG00010016905 | C1QC | 348 | 279 | 417 | 0.669 | 4.76E-03 |
| ENSGALG00010016907 | SQSTM1 | 3,576 | 3,092 | 4,061 | 0.761 | 5.96E-04 |
| ENSGALG00010016915 | PLOD2 | 1,996 | 1,333 | 2,659 | 0.501 | 2.74E-10 |
| ENSGALG00010016917 | METTL6 | 1,287 | 1,699 | 874 | 1.946 | 5.98E-09 |
| ENSGALG00010016920 | C1QA | 637 | 504 | 771 | 0.653 | 4.92E-03 |
| ENSGALG00010016922 |  | 217 | 194 | 239 | 0.81 | 2.95E-02 |
| ENSGALG00010016924 | LETM1 | 3,341 | 3,689 | 2,993 | 1.233 | 2.43E-03 |
| ENSGALG00010016927 | MGAT4B | 1,398 | 1,200 | 1,595 | 0.753 | 9.71E-03 |
| ENSGALG00010016947 | CANX | 4,350 | 3,605 | 5,094 | 0.708 | 2.58E-06 |
| ENSGALG00010016948 | CCR8 | 12 | 8 | 15 | 0.57 | 2.11E-02 |
| ENSGALG00010016967 | ERLEC1 | 1,006 | 925 | 1,086 | 0.852 | 4.52E-03 |
| ENSGALG00010016976 |  | 33 | 42 | 23 | 1.836 | 6.14E-04 |
| ENSGALG00010016987 | ASB2 | 3,761 | 2,918 | 4,603 | 0.634 | 1.40E-02 |
| ENSGALG00010016988 | ECI1 | 2,148 | 1,854 | 2,441 | 0.759 | 3.79E-03 |
| ENSGALG00010016989 | RHOG2 | 1,419 | 1,671 | 1,168 | 1.43 | 1.54E-08 |
| ENSGALG00010016994 | ATL2 | 4,054 | 4,344 | 3,764 | 1.155 | 4.53E-02 |
| ENSGALG00010017008 | RTN1 | 135 | 150 | 121 | 1.249 | 2.90E-02 |
| ENSGALG00010017012 | SYNGR3 | 249 | 175 | 323 | 0.541 | 1.21E-03 |
| ENSGALG00010017030 |  | 13 | 9 | 17 | 0.527 | 1.06E-02 |
| ENSGALG00010017033 | LDAF1 | 1,036 | 956 | 1,116 | 0.857 | 1.83E-02 |
| ENSGALG00010017039 | MAP2K5 | 452 | 424 | 481 | 0.885 | 8.40E-03 |
| ENSGALG00010017042 | HNRNPLL | 1,164 | 1,102 | 1,225 | 0.9 | 2.31E-02 |
| ENSGALG00010017044 |  | 2 | 1 | 4 | 0.16 | 1.43E-02 |
| ENSGALG00010017045 | GLB1 | 741 | 687 | 796 | 0.862 | 3.61E-03 |
| ENSGALG00010017050 | L3HYPDH | 79 | 66 | 92 | 0.713 | 1.63E-02 |
| ENSGALG00010017054 | GALM | 231 | 196 | 266 | 0.735 | 1.94E-02 |
| ENSGALG00010017056 | AGTR1 | 267 | 299 | 236 | 1.265 | 4.90E-02 |
| ENSGALG00010017077 | CTNNBIP1 | 1,107 | 1,018 | 1,195 | 0.852 | 1.69E-02 |
| ENSGALG00010017089 | CRTAP | 722 | 543 | 901 | 0.603 | 7.42E-05 |
| ENSGALG00010017096 | DCTN5 | 678 | 645 | 712 | 0.907 | 1.54E-02 |
| ENSGALG00010017098 | DAAM1 | 418 | 447 | 389 | 1.151 | 4.87E-02 |
| ENSGALG00010017100 | SUSD5 | 7 | 5 | 10 | 0.513 | 4.37E-02 |
| ENSGALG00010017106 | CRYM | 100 | 85 | 116 | 0.733 | 3.40E-02 |
| ENSGALG00010017115 | LGALSL | 5,947 | 7,000 | 4,894 | 1.431 | 1.20E-03 |
| ENSGALG00010017116 |  | 39 | 21 | 57 | 0.375 | 4.37E-04 |
| ENSGALG00010017127 | SOX8 | 262 | 295 | 229 | 1.293 | 4.01E-04 |
| ENSGALG00010017132 | TMEM108 | 78 | 44 | 113 | 0.39 | 3.01E-05 |
| ENSGALG00010017141 | AFTPH | 633 | 679 | 587 | 1.154 | 4.63E-03 |
| ENSGALG00010017143 | FAM149A | 81 | 62 | 99 | 0.626 | 2.90E-02 |
| ENSGALG00010017153 | SLC35F4 | 7 | 3 | 11 | 0.255 | 3.20E-02 |
| ENSGALG00010017163 | CDV3 | 5,469 | 4,620 | 6,317 | 0.731 | 5.09E-04 |
| ENSGALG00010017215 | FRMD7 | 57 | 71 | 44 | 1.605 | 2.87E-02 |
| ENSGALG00010017246 | GGA2 | 187 | 167 | 208 | 0.798 | 1.61E-03 |
| ENSGALG00010017248 | TECPR2 | 1,743 | 1,865 | 1,621 | 1.151 | 4.14E-02 |
| ENSGALG00010017252 | EXOC5 | 1,696 | 1,561 | 1,832 | 0.852 | 2.62E-04 |
| ENSGALG00010017296 | SCNN1G | 156 | 180 | 132 | 1.374 | 1.04E-03 |
| ENSGALG00010017307 | TMEM186 | 1,132 | 1,525 | 739 | 2.065 | 1.94E-07 |
| ENSGALG00010017314 | CALML4 | 803 | 514 | 1,092 | 0.47 | 1.76E-06 |
| ENSGALG00010017318 | ADSS1 | 34,530 | 40,693 | 28,368 | 1.434 | 7.39E-04 |
| ENSGALG00010017337 | ABAT | 1,239 | 1,361 | 1,117 | 1.218 | 3.23E-02 |
| ENSGALG00010017344 | RERE | 1,840 | 2,106 | 1,573 | 1.339 | 7.15E-03 |
| ENSGALG00010017346 | GM2A | 196 | 157 | 235 | 0.666 | 4.03E-05 |
| ENSGALG00010017352 | GPCPD1 | 3,021 | 3,365 | 2,678 | 1.257 | 1.81E-02 |
| ENSGALG00010017359 | EHD3 | 7,524 | 8,024 | 7,025 | 1.143 | 2.26E-03 |
| ENSGALG00010017362 | ENO1 | 26,326 | 23,258 | 29,394 | 0.791 | 5.25E-05 |
| ENSGALG00010017368 | ERLIN2 | 6,195 | 6,538 | 5,851 | 1.118 | 1.95E-02 |
| ENSGALG00010017383 |  | 421 | 498 | 344 | 1.453 | 7.75E-04 |
| ENSGALG00010017400 | GPC4 | 2,748 | 2,966 | 2,530 | 1.173 | 5.94E-03 |
| ENSGALG00010017403 | ITGA11 | 199 | 233 | 164 | 1.424 | 5.99E-03 |
| ENSGALG00010017418 | CHGB | 782 | 503 | 1,061 | 0.473 | 1.18E-05 |
| ENSGALG00010017424 | APOD | 107 | 70 | 144 | 0.483 | 4.79E-04 |
| ENSGALG00010017435 | ACTR10 | 1,436 | 1,295 | 1,576 | 0.822 | 2.17E-03 |
| ENSGALG00010017470 | HSPA2 | 17,911 | 15,640 | 20,181 | 0.775 | 4.33E-04 |
| ENSGALG00010017475 | STUM | 1,145 | 826 | 1,465 | 0.564 | 8.87E-05 |
| ENSGALG00010017477 | MCF2 | 3,248 | 3,882 | 2,615 | 1.485 | 6.26E-05 |
| ENSGALG00010017485 | LBH | 2,858 | 2,611 | 3,106 | 0.841 | 4.01E-04 |
| ENSGALG00010017487 | C1QTNF8 | 85 | 110 | 60 | 1.838 | 5.13E-04 |
| ENSGALG00010017493 | YPEL5 | 1,288 | 1,160 | 1,415 | 0.819 | 7.42E-04 |
| ENSGALG00010017496 | PARP1 | 2,502 | 2,216 | 2,789 | 0.794 | 1.61E-04 |
| ENSGALG00010017513 | TNIP2 | 75 | 60 | 89 | 0.674 | 1.43E-03 |
| ENSGALG00010017528 | RAI1 | 309 | 274 | 345 | 0.793 | 2.32E-02 |
| ENSGALG00010017547 | RNF4 | 1,684 | 1,794 | 1,574 | 1.142 | 2.45E-03 |
| ENSGALG00010017568 | CDCP1 | 27 | 19 | 36 | 0.53 | 3.49E-02 |
| ENSGALG00010017571 | ADAM9 | 1,725 | 1,561 | 1,889 | 0.826 | 1.63E-03 |
| ENSGALG00010017574 | DYNC1LI1 | 1,943 | 1,548 | 2,338 | 0.662 | 3.79E-11 |
| ENSGALG00010017576 |  | 72 | 61 | 83 | 0.724 | 1.44E-02 |
| ENSGALG00010017581 | EFCAB11 | 5 | 2 | 7 | 0.376 | 1.63E-02 |
| ENSGALG00010017587 | MB21D2 | 215 | 175 | 255 | 0.687 | 1.10E-03 |
| ENSGALG00010017591 | ESR2 | 42 | 52 | 32 | 1.664 | 2.82E-03 |
| ENSGALG00010017604 | GATM | 6,315 | 7,526 | 5,104 | 1.475 | 2.34E-02 |
| ENSGALG00010017631 | NAT8L | 61 | 49 | 74 | 0.658 | 3.52E-02 |
| ENSGALG00010017638 | GFRA2 | 162 | 214 | 111 | 1.928 | 1.38E-09 |
| ENSGALG00010017641 | GPRC5B | 4,201 | 5,049 | 3,353 | 1.506 | 1.03E-04 |
| ENSGALG00010017643 | CALM1 | 57,421 | 52,257 | 62,585 | 0.835 | 1.69E-02 |
| ENSGALG00010017644 | RBBP6 | 1,677 | 1,786 | 1,568 | 1.14 | 1.98E-02 |
| ENSGALG00010017647 | ITGA9 | 743 | 873 | 612 | 1.428 | 2.59E-03 |
| ENSGALG00010017649 |  | 132 | 148 | 116 | 1.27 | 9.39E-03 |
| ENSGALG00010017652 | C1orf115 | 202 | 225 | 178 | 1.262 | 6.89E-03 |
| ENSGALG00010017653 |  | 253 | 299 | 208 | 1.442 | 1.24E-04 |
| ENSGALG00010017654 | KBTBD13 | 162 | 134 | 190 | 0.709 | 1.68E-02 |
| ENSGALG00010017655 |  | 266 | 284 | 248 | 1.143 | 4.50E-02 |
| ENSGALG00010017660 | CLIP4 | 13,119 | 14,744 | 11,494 | 1.283 | 2.77E-02 |
| ENSGALG00010017661 | NHSL3 | 56 | 47 | 65 | 0.724 | 2.94E-02 |
| ENSGALG00010017669 |  | 52 | 64 | 40 | 1.601 | 2.15E-05 |
| ENSGALG00010017691 | LZTS1 | 62 | 40 | 85 | 0.472 | 1.45E-05 |
| ENSGALG00010017693 | MTFMT | 314 | 347 | 280 | 1.242 | 3.12E-02 |
| ENSGALG00010017695 | FNDC5 | 343 | 228 | 458 | 0.497 | 5.65E-04 |
| ENSGALG00010017713 | ALKBH5 | 17,413 | 20,341 | 14,485 | 1.404 | 1.08E-03 |
| ENSGALG00010017715 | CILP | 5,119 | 3,145 | 7,093 | 0.443 | 5.96E-05 |
| ENSGALG00010017721 | VWA1 | 1,961 | 1,437 | 2,485 | 0.578 | 1.15E-05 |
| ENSGALG00010017724 | FLII | 6,148 | 5,703 | 6,594 | 0.865 | 2.25E-02 |
| ENSGALG00010017737 |  | 38 | 46 | 31 | 1.478 | 3.38E-03 |
| ENSGALG00010017746 | MSH6 | 1,368 | 1,544 | 1,192 | 1.296 | 9.18E-03 |
| ENSGALG00010017761 | PARP16 | 375 | 266 | 485 | 0.547 | 8.42E-09 |
| ENSGALG00010017772 |  | 269 | 137 | 401 | 0.343 | 2.00E-14 |
| ENSGALG00010017776 | GPR68 | 362 | 212 | 512 | 0.415 | 7.34E-05 |
| ENSGALG00010017789 | IMMT | 10,316 | 11,588 | 9,043 | 1.282 | 4.70E-04 |
| ENSGALG00010017801 | SMNDC1 | 1,156 | 1,218 | 1,094 | 1.114 | 9.75E-03 |
| ENSGALG00010017804 | DUSP5 | 197 | 119 | 275 | 0.433 | 6.25E-04 |
| ENSGALG00010017808 | SFRP5 | 67 | 40 | 93 | 0.43 | 1.11E-04 |
| ENSGALG00010017816 | PRKCH | 218 | 295 | 140 | 2.104 | 5.79E-05 |
| ENSGALG00010017822 | VWA3A | 33 | 40 | 25 | 1.628 | 2.05E-02 |
| ENSGALG00010017828 |  | 533 | 358 | 709 | 0.504 | 5.67E-08 |
| ENSGALG00010017830 | DENND4A | 2,294 | 2,735 | 1,853 | 1.476 | 5.97E-03 |
| ENSGALG00010017850 | TRMT5 | 280 | 305 | 255 | 1.194 | 9.79E-03 |
| ENSGALG00010017852 | LETM2 | 236 | 271 | 202 | 1.343 | 1.04E-02 |
| ENSGALG00010017858 | GPR65 | 205 | 149 | 261 | 0.572 | 2.80E-02 |
| ENSGALG00010017868 | CHRND | 1,412 | 690 | 2,133 | 0.324 | 4.79E-18 |
| ENSGALG00010017878 | CHRNG | 862 | 414 | 1,310 | 0.316 | 9.19E-12 |
| ENSGALG00010017886 | DDRGK1 | 2,085 | 1,705 | 2,465 | 0.692 | 5.29E-05 |
| ENSGALG00010017898 | NHERF2 | 880 | 938 | 821 | 1.141 | 1.93E-02 |
| ENSGALG00010017905 | EIF4E2 | 1,092 | 995 | 1,189 | 0.837 | 3.45E-04 |
| ENSGALG00010017927 | PCSK2 | 132 | 149 | 116 | 1.285 | 3.20E-02 |
| ENSGALG00010017963 | LPAR4 | 270 | 293 | 247 | 1.186 | 2.86E-02 |
| ENSGALG00010017974 | MXRA8 | 1,299 | 1,104 | 1,494 | 0.738 | 4.21E-02 |
| ENSGALG00010017991 | RAB26 | 264 | 223 | 304 | 0.734 | 4.13E-03 |
| ENSGALG00010017996 | ZC3H14 | 1,030 | 1,096 | 964 | 1.138 | 1.63E-02 |
| ENSGALG00010018002 | ZC3H8 | 1,108 | 1,201 | 1,014 | 1.186 | 8.10E-03 |
| ENSGALG00010018007 | DNAAF9 | 1,688 | 2,123 | 1,254 | 1.693 | 4.44E-04 |
| *ENSGALG00010018011* | *LIPA* | *1,721* | *1,389* | *2,054* | *0.676* | *1.15E-05* |
| ENSGALG00010018032 | ERO1A | 221 | 206 | 236 | 0.869 | 3.29E-02 |
| ENSGALG00010018036 | RPL4 | 26,852 | 24,546 | 29,157 | 0.842 | 9.07E-04 |
| ENSGALG00010018037 | GZF1 | 257 | 277 | 237 | 1.167 | 4.19E-02 |
| ENSGALG00010018051 | GNPNAT1 | 481 | 512 | 451 | 1.138 | 3.97E-02 |
| ENSGALG00010018059 |  | 16 | 10 | 22 | 0.435 | 1.64E-03 |
| ENSGALG00010018063 | DECR2 | 794 | 724 | 864 | 0.839 | 1.59E-02 |
| ENSGALG00010018067 |  | 210 | 238 | 183 | 1.302 | 2.26E-03 |
| ENSGALG00010018068 | ADAM33 | 450 | 380 | 520 | 0.729 | 6.89E-03 |
| ENSGALG00010018070 |  | 5 | 6 | 3 | 1.963 | 4.89E-02 |
| ENSGALG00010018085 | PGP | 7,481 | 8,499 | 6,462 | 1.315 | 2.70E-02 |
| ENSGALG00010018099 | SMAD3 | 1,305 | 1,082 | 1,527 | 0.708 | 7.48E-04 |
| ENSGALG00010018121 | FERMT2 | 2,647 | 2,480 | 2,815 | 0.881 | 3.76E-02 |
| ENSGALG00010018163 | CCT7 | 3,258 | 3,021 | 3,495 | 0.864 | 3.26E-02 |
| ENSGALG00010018165 | DACH2 | 123 | 137 | 109 | 1.266 | 3.76E-02 |
| ENSGALG00010018172 | ACTL6A | 617 | 648 | 586 | 1.108 | 3.28E-02 |
| ENSGALG00010018198 | BTBD3 | 496 | 553 | 439 | 1.262 | 5.65E-04 |
| ENSGALG00010018209 | GMPPA | 1,085 | 968 | 1,203 | 0.805 | 2.65E-03 |
| ENSGALG00010018226 | JAG1 | 580 | 634 | 527 | 1.201 | 2.21E-02 |
| ENSGALG00010018251 | USP48 | 1,291 | 1,241 | 1,341 | 0.926 | 3.49E-02 |
| ENSGALG00010018261 | PRNP | 3,165 | 2,705 | 3,625 | 0.746 | 4.73E-03 |
| ENSGALG00010018264 | CFAP61 | 13 | 17 | 8 | 2.116 | 5.62E-04 |
| ENSGALG00010018276 | ARHGAP17 | 2,505 | 2,313 | 2,697 | 0.857 | 4.81E-02 |
| ENSGALG00010018287 | MKKS | 1,769 | 1,925 | 1,614 | 1.193 | 4.05E-02 |
| ENSGALG00010018299 | SHOC2 | 513 | 544 | 482 | 1.13 | 3.55E-02 |
| ENSGALG00010018304 |  | 4 | 6 | 2 | 2.646 | 5.94E-03 |
| ENSGALG00010018306 | DES | 44,696 | 31,514 | 57,877 | 0.545 | 2.61E-12 |
| ENSGALG00010018313 | MYEF2 | 846 | 903 | 788 | 1.143 | 1.44E-03 |
| ENSGALG00010018329 |  | 101 | 75 | 128 | 0.584 | 6.69E-03 |
| ENSGALG00010018337 | TMEM151B | 58 | 36 | 80 | 0.449 | 2.44E-02 |
| ENSGALG00010018338 | PDCD6IP | 7,975 | 7,149 | 8,801 | 0.812 | 1.02E-03 |
| ENSGALG00010018344 | SFXN5 | 131 | 148 | 114 | 1.303 | 4.46E-04 |
| ENSGALG00010018345 | WDR24 | 582 | 636 | 529 | 1.205 | 1.37E-02 |
| ENSGALG00010018346 |  | 69 | 30 | 108 | 0.28 | 3.95E-03 |
| ENSGALG00010018351 | RIN2 | 1,436 | 1,361 | 1,511 | 0.9 | 4.93E-02 |
| ENSGALG00010018365 | HPSE2 | 66 | 93 | 38 | 2.478 | 4.04E-05 |
| ENSGALG00010018371 |  | 1,566 | 1,373 | 1,758 | 0.78 | 1.03E-02 |
| ENSGALG00010018374 | HPS1 | 83 | 58 | 108 | 0.54 | 1.86E-06 |
| ENSGALG00010018395 | MED12 | 958 | 1,063 | 852 | 1.248 | 3.40E-02 |
| ENSGALG00010018399 | ABHD12 | 835 | 731 | 940 | 0.777 | 3.98E-02 |
| ENSGALG00010018400 |  | 7 | 5 | 9 | 0.545 | 2.62E-02 |
| ENSGALG00010018404 | IL2RG | 213 | 165 | 262 | 0.629 | 3.23E-02 |
| ENSGALG00010018414 | EPAS1 | 4,723 | 5,222 | 4,225 | 1.236 | 3.38E-03 |
| ENSGALG00010018419 | USP7 | 4,443 | 4,721 | 4,164 | 1.134 | 2.18E-02 |
| ENSGALG00010018423 | IGFALS | 16 | 12 | 20 | 0.604 | 3.60E-02 |
| ENSGALG00010018434 | HSPBAP1 | 240 | 268 | 213 | 1.262 | 2.74E-02 |
| ENSGALG00010018443 | PLCB1 | 2,593 | 2,898 | 2,288 | 1.267 | 5.86E-03 |
| ENSGALG00010018446 | FBN1 | 6,260 | 6,925 | 5,595 | 1.238 | 3.20E-02 |
| ENSGALG00010018462 | KTN1 | 3,564 | 3,242 | 3,887 | 0.834 | 1.10E-03 |
| ENSGALG00010018464 |  | 5 | 3 | 7 | 0.34 | 1.76E-02 |
| ENSGALG00010018469 | KLHDC7A | 214 | 250 | 178 | 1.404 | 7.56E-03 |
| ENSGALG00010018473 |  | 76 | 93 | 59 | 1.571 | 2.77E-02 |
| ENSGALG00010018480 | EME2 | 120 | 156 | 83 | 1.886 | 1.03E-04 |
| ENSGALG00010018490 | NME3 | 844 | 1,027 | 660 | 1.556 | 2.35E-09 |
| ENSGALG00010018511 | SOCS5 | 906 | 966 | 846 | 1.142 | 5.55E-03 |
| ENSGALG00010018512 | FERMT1 | 127 | 110 | 144 | 0.757 | 7.22E-05 |
| ENSGALG00010018514 | EAF2 | 469 | 580 | 358 | 1.617 | 3.09E-06 |
| ENSGALG00010018529 | UBR2 | 3,196 | 3,520 | 2,873 | 1.225 | 2.25E-02 |
| ENSGALG00010018534 | NCEH1 | 4,076 | 4,978 | 3,173 | 1.569 | 9.70E-05 |
| ENSGALG00010018544 | HACD2 | 302 | 326 | 277 | 1.171 | 3.27E-02 |
| ENSGALG00010018548 | RGMA | 469 | 504 | 434 | 1.164 | 3.07E-02 |
| ENSGALG00010018561 | ACTR1A | 5,108 | 4,716 | 5,499 | 0.857 | 8.99E-03 |
| ENSGALG00010018564 | DLGAP5 | 275 | 306 | 244 | 1.249 | 1.57E-02 |
| ENSGALG00010018566 |  | 45 | 30 | 60 | 0.505 | 1.25E-02 |
| ENSGALG00010018577 | NARFL | 731 | 784 | 679 | 1.155 | 4.09E-02 |
| ENSGALG00010018583 | PPL | 1,009 | 830 | 1,187 | 0.698 | 4.14E-02 |
| ENSGALG00010018594 | BICRAL | 368 | 406 | 330 | 1.232 | 1.82E-02 |
| *ENSGALG00010018616* |  | *8,211* | *16,199* | *222* | *72.77* | *2.37E-34* |
| ENSGALG00010018649 | HAGH | 4,248 | 3,619 | 4,877 | 0.742 | 7.10E-04 |
| ENSGALG00010018670 | MRAS | 2,330 | 1,704 | 2,956 | 0.576 | 7.95E-03 |
| ENSGALG00010018694 | MGME1 | 230 | 260 | 201 | 1.296 | 1.35E-04 |
| ENSGALG00010018696 |  | 271 | 336 | 205 | 1.638 | 1.00E-03 |
| ENSGALG00010018704 | PGPEP1L | 620 | 860 | 381 | 2.263 | 9.85E-04 |
| ENSGALG00010018756 | EML4 | 927 | 1,016 | 838 | 1.212 | 2.39E-02 |
| ENSGALG00010018758 | TNFRSF9 | 212 | 185 | 240 | 0.769 | 3.39E-02 |
| ENSGALG00010018774 | MSRB1 | 873 | 774 | 971 | 0.797 | 1.18E-02 |
| ENSGALG00010018790 | PRR35 | 2 | 3 | 1 | 3.68 | 3.23E-02 |
| ENSGALG00010018806 | GCH1 | 121 | 138 | 103 | 1.347 | 1.94E-02 |
| ENSGALG00010018811 | VAMP3 | 1,736 | 1,520 | 1,952 | 0.779 | 1.91E-03 |
| ENSGALG00010018822 | TRAF5 | 57 | 47 | 66 | 0.704 | 1.18E-04 |
| ENSGALG00010018831 | WBP1L | 651 | 553 | 748 | 0.738 | 2.75E-04 |
| ENSGALG00010018832 | EEPD1 | 570 | 466 | 673 | 0.693 | 9.70E-05 |
| ENSGALG00010018837 | RCOR3 | 839 | 903 | 775 | 1.165 | 3.03E-02 |
| ENSGALG00010018838 | WFIKKN1 | 4 | 2 | 6 | 0.281 | 3.72E-02 |
| ENSGALG00010018839 | RPL3L | 20,868 | 17,722 | 24,014 | 0.738 | 3.89E-05 |
| ENSGALG00010018848 | MEF2A | 8,030 | 8,634 | 7,425 | 1.163 | 3.48E-03 |
| ENSGALG00010018867 | RPS2 | 19,152 | 16,574 | 21,731 | 0.763 | 3.78E-04 |
| ENSGALG00010018873 |  | 65 | 74 | 56 | 1.339 | 3.40E-02 |
| ENSGALG00010018886 |  | 1,380 | 1,697 | 1,062 | 1.598 | 9.96E-04 |
| ENSGALG00010018897 | CCDC85A | 197 | 227 | 167 | 1.364 | 1.25E-05 |
| ENSGALG00010018902 |  | 138 | 102 | 173 | 0.59 | 1.77E-02 |
| ENSGALG00010018904 | UGP2 | 12,315 | 14,431 | 10,199 | 1.415 | 2.76E-07 |
| ENSGALG00010018913 |  | 85 | 67 | 103 | 0.649 | 5.63E-04 |
| ENSGALG00010018917 | DNAJC11 | 1,245 | 1,323 | 1,167 | 1.135 | 1.00E-02 |
| ENSGALG00010018919 | RALB | 4,956 | 5,395 | 4,518 | 1.194 | 5.37E-03 |
| ENSGALG00010018928 |  | 9 | 4 | 14 | 0.257 | 3.39E-03 |
| ENSGALG00010018933 | PHF13 | 567 | 513 | 622 | 0.824 | 1.74E-02 |
| ENSGALG00010018936 | SYT14 | 374 | 438 | 311 | 1.41 | 3.28E-02 |
| ENSGALG00010018939 |  | 13 | 19 | 7 | 2.764 | 2.29E-02 |
| ENSGALG00010018954 |  | 9 | 13 | 4 | 3.447 | 4.90E-05 |
| ENSGALG00010018972 |  | 4 | 7 | 2 | 3.58 | 7.04E-04 |
| ENSGALG00010018991 | RYBP | 141 | 128 | 155 | 0.821 | 1.84E-02 |
| ENSGALG00010019001 | ALDH1A3 | 1,422 | 1,167 | 1,678 | 0.696 | 2.05E-05 |
| ENSGALG00010019017 | IRAK2 | 3,936 | 2,985 | 4,887 | 0.611 | 2.51E-14 |
| ENSGALG00010019020 | KCNK2 | 123 | 84 | 162 | 0.514 | 2.35E-02 |
| ENSGALG00010019023 | AP2M1 | 7,232 | 6,477 | 7,987 | 0.811 | 1.27E-06 |
| ENSGALG00010019029 | CENPF | 237 | 265 | 208 | 1.272 | 1.44E-02 |
| ENSGALG00010019036 | REL | 387 | 343 | 432 | 0.791 | 2.26E-02 |
| ENSGALG00010019050 | TNS1 | 1,880 | 1,580 | 2,181 | 0.724 | 9.11E-04 |
| ENSGALG00010019051 | IGSF6 | 76 | 60 | 91 | 0.656 | 2.50E-02 |
| *ENSGALG00010019065* | *CAND2* | *19,881* | *21,704* | *18,058* | *1.202* | *1.73E-02* |
| ENSGALG00010019081 | PCSK6 | 99 | 114 | 84 | 1.373 | 6.36E-03 |
| ENSGALG00010019094 | SNRNP25 | 87 | 67 | 106 | 0.637 | 5.10E-03 |
| ENSGALG00010019108 | KDELR2 | 1,907 | 1,782 | 2,032 | 0.877 | 1.51E-03 |
| ENSGALG00010019113 | KLHDC1 | 635 | 568 | 701 | 0.81 | 4.72E-03 |
| ENSGALG00010019127 | TMBIM1 | 1,816 | 1,548 | 2,084 | 0.743 | 2.48E-08 |
| ENSGALG00010019186 |  | 81 | 103 | 58 | 1.787 | 2.06E-03 |
| ENSGALG00010019213 | RHBDF1 | 1,304 | 916 | 1,692 | 0.541 | 2.51E-20 |
| ENSGALG00010019221 | VASH2 | 2,797 | 1,999 | 3,594 | 0.556 | 1.66E-06 |
| ENSGALG00010019232 | SOS2 | 4,083 | 4,367 | 3,800 | 1.149 | 2.13E-02 |
| ENSGALG00010019233 | ARPC2 | 1,701 | 1,555 | 1,848 | 0.841 | 1.22E-02 |
| ENSGALG00010019265 | RUFY4 | 4 | 2 | 6 | 0.438 | 6.89E-03 |
| ENSGALG00010019297 | SLC25A26 | 273 | 243 | 303 | 0.803 | 2.25E-02 |
| ENSGALG00010019298 | PGAP6 | 1,261 | 1,127 | 1,395 | 0.808 | 3.52E-02 |
| ENSGALG00010019322 | EGR3 | 34 | 26 | 43 | 0.601 | 1.81E-02 |
| ENSGALG00010019344 | DNAJC24 | 234 | 260 | 208 | 1.248 | 4.08E-03 |
| ENSGALG00010019391 | GANC | 5,431 | 6,179 | 4,684 | 1.319 | 1.25E-02 |
| ENSGALG00010019397 |  | 6 | 9 | 4 | 2.401 | 8.73E-03 |
| ENSGALG00010019399 | CD248 | 269 | 310 | 228 | 1.357 | 6.08E-03 |
| ENSGALG00010019441 | LMCD1 | 1,857 | 1,143 | 2,570 | 0.445 | 6.23E-11 |
| ENSGALG00010019451 | CAV3 | 6,442 | 4,794 | 8,091 | 0.593 | 1.46E-08 |
| ENSGALG00010019534 | PPP2R5A | 3,587 | 3,298 | 3,874 | 0.852 | 3.06E-02 |
| ENSGALG00010019572 | NFKBIE | 69 | 52 | 85 | 0.605 | 9.52E-04 |
| ENSGALG00010019598 | KCNAB2 | 182 | 148 | 216 | 0.688 | 1.09E-03 |
| ENSGALG00010019610 | CORO7 | 265 | 230 | 300 | 0.766 | 1.83E-02 |
| ENSGALG00010019621 |  | 5,022 | 4,188 | 5,857 | 0.715 | 5.02E-04 |
| ENSGALG00010019624 | SRGAP3 | 239 | 215 | 263 | 0.817 | 1.68E-02 |
| ENSGALG00010019641 | CAPN11 | 10,623 | 8,954 | 12,290 | 0.728 | 1.14E-10 |
| ENSGALG00010019649 | TMEM63B | 1,173 | 1,052 | 1,294 | 0.813 | 4.66E-04 |
| ENSGALG00010019650 | KBTBD8 | 1,859 | 1,994 | 1,723 | 1.157 | 8.32E-03 |
| ENSGALG00010019667 | ITPRID1 | 15 | 21 | 9 | 2.349 | 1.19E-02 |
| ENSGALG00010019674 |  | 2 | 4 | 1 | 3.065 | 3.27E-02 |
| ENSGALG00010019716 | VEGFA | 629 | 769 | 489 | 1.572 | 1.01E-07 |
| ENSGALG00010019731 | PANK4 | 1,708 | 1,910 | 1,506 | 1.27 | 3.88E-04 |
| ENSGALG00010019778 | ABHD17C | 393 | 430 | 356 | 1.205 | 2.15E-03 |
| ENSGALG00010019804 | ELFN1 | 65 | 32 | 98 | 0.332 | 3.20E-06 |
| ENSGALG00010019811 | UBXN4 | 4,986 | 4,531 | 5,441 | 0.833 | 2.74E-02 |
| ENSGALG00010019816 | GPR132 | 24 | 19 | 29 | 0.652 | 4.77E-02 |
| ENSGALG00010019820 | BBOX1 | 1,169 | 1,498 | 840 | 1.787 | 1.44E-05 |
| ENSGALG00010019830 | JAG2 | 1,198 | 1,311 | 1,085 | 1.208 | 1.43E-03 |
| ENSGALG00010019836 | MINAR1 | 18 | 24 | 13 | 1.928 | 1.28E-03 |
| ENSGALG00010019839 |  | 2 | 0 | 3 | 0.087 | 3.22E-02 |
| ENSGALG00010019841 |  | 4 | 2 | 6 | 0.305 | 2.07E-02 |
| ENSGALG00010019845 |  | 2 | 1 | 3 | 0.279 | 1.85E-02 |
| ENSGALG00010019846 |  | 816 | 947 | 686 | 1.382 | 6.02E-06 |
| ENSGALG00010019848 | MAD1L1 | 315 | 292 | 339 | 0.862 | 1.98E-02 |
| ENSGALG00010019850 |  | 3 | 0 | 6 | 0.087 | 7.26E-03 |
| ENSGALG00010019891 | PIGH | 276 | 243 | 308 | 0.79 | 2.32E-02 |
| ENSGALG00010019919 | LIN7C | 2,207 | 2,440 | 1,975 | 1.236 | 1.09E-03 |
| ENSGALG00010019945 |  | 3 | 5 | 2 | 3.061 | 4.46E-02 |
| ENSGALG00010019968 | RHCG | 4 | 2 | 7 | 0.232 | 3.55E-02 |
| ENSGALG00010019972 | PPP1CB | 12,670 | 11,168 | 14,173 | 0.788 | 6.26E-04 |
| ENSGALG00010019982 | PLCE1 | 930 | 1,067 | 794 | 1.343 | 1.85E-02 |
| ENSGALG00010019992 | CHAC2 | 129 | 110 | 148 | 0.745 | 1.08E-03 |
| ENSGALG00010020002 |  | 47 | 36 | 57 | 0.628 | 3.64E-02 |
| ENSGALG00010020005 | EOGT | 788 | 897 | 678 | 1.323 | 9.44E-05 |
| ENSGALG00010020009 |  | 464 | 506 | 422 | 1.202 | 4.15E-02 |
| ENSGALG00010020013 | ADAM8 | 56 | 29 | 83 | 0.348 | 1.88E-02 |
| ENSGALG00010020056 | UACA | 1,533 | 1,301 | 1,765 | 0.737 | 1.61E-03 |
| ENSGALG00010020059 | PRKCZ | 370 | 415 | 324 | 1.285 | 4.68E-03 |
| ENSGALG00010020066 |  | 29 | 22 | 35 | 0.622 | 1.16E-02 |
| ENSGALG00010020070 | ARL6IP5 | 7,048 | 5,880 | 8,216 | 0.716 | 7.62E-06 |
| ENSGALG00010020072 |  | 84 | 97 | 71 | 1.37 | 1.18E-02 |
| ENSGALG00010020074 |  | 2,090 | 1,552 | 2,628 | 0.591 | 2.78E-02 |
| ENSGALG00010020098 | GABRD | 248 | 325 | 172 | 1.891 | 1.66E-02 |
| ENSGALG00010020100 | MOCS1 | 5,111 | 5,736 | 4,486 | 1.279 | 6.08E-03 |
| ENSGALG00010020103 | PSAP | 26,726 | 23,494 | 29,958 | 0.784 | 9.03E-03 |
| ENSGALG00010020104 | BRAT1 | 270 | 300 | 240 | 1.251 | 1.56E-04 |
| ENSGALG00010020136 | KCNK17 | 28 | 22 | 34 | 0.626 | 4.02E-02 |
| ENSGALG00010020193 | VSTM4 | 121 | 137 | 106 | 1.291 | 2.94E-02 |
| ENSGALG00010020199 | DET1 | 601 | 568 | 634 | 0.894 | 2.44E-02 |
| ENSGALG00010020250 |  | 115 | 137 | 93 | 1.479 | 2.00E-02 |
| ENSGALG00010020282 | MAGI1 | 127 | 140 | 114 | 1.222 | 4.38E-02 |
| ENSGALG00010020283 | FBXO42 | 471 | 506 | 436 | 1.162 | 1.89E-02 |
| ENSGALG00010020285 | ADAMTS14 | 52 | 39 | 64 | 0.603 | 4.23E-02 |
| ENSGALG00010020290 | NELL1 | 10 | 4 | 16 | 0.216 | 1.21E-03 |
| ENSGALG00010020307 | SHQ1 | 139 | 116 | 161 | 0.722 | 2.49E-03 |
| ENSGALG00010020350 | PDZRN3 | 3,592 | 3,870 | 3,315 | 1.168 | 2.80E-02 |
| ENSGALG00010020351 | CROCC | 43 | 50 | 35 | 1.422 | 2.88E-02 |
| ENSGALG00010020377 | TMEM230 | 267 | 239 | 295 | 0.81 | 1.07E-03 |
| ENSGALG00010020443 | PREPL | 298 | 331 | 264 | 1.254 | 2.37E-02 |
| ENSGALG00010020460 | MAPK8 | 752 | 822 | 682 | 1.205 | 9.24E-03 |
| ENSGALG00010020478 |  | 10 | 16 | 5 | 3.382 | 7.99E-06 |
| ENSGALG00010020485 | LMAN2L | 1,565 | 1,311 | 1,819 | 0.721 | 5.67E-04 |
| ENSGALG00010020507 | ANKRD1 | 46,563 | 36,139 | 56,987 | 0.634 | 3.74E-02 |
| ENSGALG00010020524 |  | 49 | 37 | 60 | 0.622 | 9.16E-03 |
| ENSGALG00010020533 | UNKL | 423 | 394 | 452 | 0.868 | 2.35E-02 |
| ENSGALG00010020550 | HECTD2 | 1,800 | 1,452 | 2,147 | 0.676 | 1.85E-03 |
| ENSGALG00010020564 |  | 69 | 93 | 44 | 2.115 | 2.90E-03 |
| ENSGALG00010020568 |  | 57 | 80 | 34 | 2.33 | 4.48E-07 |
| ENSGALG00010020584 | PARP9 | 462 | 355 | 570 | 0.623 | 9.51E-03 |
| ENSGALG00010020612 | RPL27A | 7,084 | 6,084 | 8,084 | 0.753 | 1.61E-02 |
| ENSGALG00010020616 | PI4K2A | 582 | 528 | 636 | 0.83 | 2.45E-03 |
| ENSGALG00010020623 | NEK2 | 49 | 56 | 43 | 1.298 | 4.37E-02 |
| ENSGALG00010020625 | SLC30A1 | 725 | 671 | 779 | 0.861 | 2.99E-02 |
| ENSGALG00010020626 | MORN4 | 254 | 207 | 301 | 0.687 | 2.80E-04 |
| ENSGALG00010020636 | NBL1 | 806 | 728 | 884 | 0.823 | 1.50E-02 |
| ENSGALG00010020657 | DALRD3 | 214 | 194 | 235 | 0.826 | 2.31E-02 |
| ENSGALG00010020662 | BFAR | 434 | 392 | 475 | 0.828 | 7.42E-04 |
| ENSGALG00010020673 | MMS19 | 540 | 500 | 580 | 0.86 | 4.79E-02 |
| ENSGALG00010020677 | CYR61 | 1,080 | 617 | 1,543 | 0.4 | 1.94E-07 |
| ENSGALG00010020681 | BCL10 | 531 | 482 | 580 | 0.829 | 4.35E-03 |
| ENSGALG00010020685 | RPL6 | 16,062 | 14,798 | 17,325 | 0.854 | 3.26E-02 |
| ENSGALG00010020694 |  | 25 | 20 | 30 | 0.659 | 3.60E-02 |
| ENSGALG00010020712 | ZC3H7A | 628 | 664 | 591 | 1.122 | 3.44E-02 |
| ENSGALG00010020732 | ARIH2 | 3,151 | 3,532 | 2,771 | 1.275 | 8.06E-03 |
| ENSGALG00010020748 |  | 27 | 35 | 18 | 1.937 | 9.03E-03 |
| ENSGALG00010020750 | AIFM2 | 221 | 178 | 263 | 0.677 | 3.66E-03 |
| ENSGALG00010020782 | PPIF | 489 | 581 | 396 | 1.469 | 1.12E-04 |
| ENSGALG00010020786 | DDX19 | 3,155 | 3,326 | 2,984 | 1.115 | 3.99E-02 |
| ENSGALG00010020787 | TMEM132C | 34 | 43 | 26 | 1.626 | 4.71E-03 |
| ENSGALG00010020794 | STEAP3 | 257 | 219 | 295 | 0.741 | 4.12E-02 |
| ENSGALG00010020809 |  | 1,894 | 1,676 | 2,112 | 0.794 | 1.59E-02 |
| ENSGALG00010020810 | IFITM5 | 725 | 591 | 860 | 0.687 | 9.41E-04 |
| ENSGALG00010020827 |  | 1,546 | 1,431 | 1,661 | 0.861 | 2.12E-02 |
| ENSGALG00010020845 | RAB35 | 1,031 | 985 | 1,076 | 0.916 | 1.44E-02 |
| ENSGALG00010020875 |  | 577 | 687 | 467 | 1.474 | 1.66E-06 |
| ENSGALG00010020900 | PDHB | 3,262 | 3,493 | 3,032 | 1.153 | 3.04E-02 |
| ENSGALG00010020903 | AGMAT | 54 | 71 | 36 | 1.99 | 1.11E-03 |
| ENSGALG00010020912 | PDE8A | 786 | 873 | 699 | 1.25 | 1.90E-02 |
| ENSGALG00010020921 | LDB1 | 176 | 162 | 190 | 0.849 | 4.95E-02 |
| ENSGALG00010020952 | GNG5 | 460 | 408 | 513 | 0.795 | 4.09E-02 |
| ENSGALG00010020956 |  | 11 | 14 | 7 | 1.901 | 5.64E-03 |
| ENSGALG00010020960 | CLEC19A | 9 | 6 | 11 | 0.548 | 4.81E-02 |
| ENSGALG00010020975 | HCCS | 4,153 | 4,574 | 3,732 | 1.226 | 1.43E-02 |
| ENSGALG00010020978 | BCL2L10 | 4,706 | 3,816 | 5,597 | 0.682 | 5.49E-05 |
| ENSGALG00010020981 | CIT | 441 | 291 | 590 | 0.493 | 1.74E-03 |
| ENSGALG00010020992 | KCNH7 | 36 | 46 | 25 | 1.86 | 1.84E-02 |
| ENSGALG00010021011 | SCARB1 | 202 | 180 | 224 | 0.804 | 3.00E-02 |
| ENSGALG00010021012 | KCNIP2 | 24 | 13 | 34 | 0.383 | 3.03E-02 |
| ENSGALG00010021014 | FBXL15 | 733 | 830 | 636 | 1.308 | 6.87E-05 |
| ENSGALG00010021015 | RBM25 | 1,553 | 1,665 | 1,442 | 1.156 | 8.36E-04 |
| ENSGALG00010021016 | FHIT | 26 | 21 | 32 | 0.636 | 2.25E-02 |
| ENSGALG00010021018 | F3 | 141 | 121 | 161 | 0.75 | 1.02E-02 |
| ENSGALG00010021020 | ZDHHC13 | 227 | 201 | 252 | 0.795 | 6.98E-03 |
| ENSGALG00010021023 | MGEA5 | 3,463 | 3,712 | 3,213 | 1.155 | 1.90E-02 |
| ENSGALG00010021024 |  | 335 | 300 | 370 | 0.81 | 1.82E-02 |
| ENSGALG00010021025 | CUEDC2 | 859 | 983 | 735 | 1.341 | 2.54E-04 |
| ENSGALG00010021027 | PLA2G4A | 432 | 388 | 477 | 0.812 | 4.58E-02 |
| ENSGALG00010021028 |  | 2 | 3 | 1 | 4.991 | 7.95E-03 |
| ENSGALG00010021030 | CSRP3 | 195 | 90 | 300 | 0.302 | 2.01E-02 |
| ENSGALG00010021041 | GLTP | 63 | 45 | 81 | 0.554 | 7.83E-03 |
| ENSGALG00010021042 | LRRC38 | 501 | 444 | 558 | 0.794 | 4.06E-03 |
| ENSGALG00010021052 | C12H3orf14 | 104 | 116 | 92 | 1.262 | 4.64E-03 |
| ENSGALG00010021069 | PGAM5 | 2,420 | 2,273 | 2,568 | 0.885 | 1.96E-02 |
| ENSGALG00010021070 | RNF141 | 2,300 | 2,525 | 2,075 | 1.218 | 9.28E-03 |
| ENSGALG00010021074 | TTC17 | 1,158 | 1,041 | 1,275 | 0.816 | 4.74E-04 |
| ENSGALG00010021075 | DHRS3 | 280 | 240 | 320 | 0.748 | 5.36E-03 |
| ENSGALG00010021080 | TTLL7 | 1,058 | 1,221 | 896 | 1.363 | 3.60E-02 |
| ENSGALG00010021085 | AMPD3 | 8,171 | 6,187 | 10,156 | 0.609 | 3.34E-07 |
| ENSGALG00010021087 | THBS1 | 1,507 | 1,142 | 1,872 | 0.61 | 1.07E-02 |
| ENSGALG00010021095 | NT5C2 | 1,722 | 1,542 | 1,902 | 0.811 | 4.15E-03 |
| ENSGALG00010021097 | KATNBL1 | 793 | 747 | 839 | 0.891 | 2.81E-02 |
| ENSGALG00010021099 | HMCN1 | 251 | 292 | 210 | 1.386 | 6.86E-03 |
| ENSGALG00010021100 | EMC7 | 1,960 | 1,775 | 2,145 | 0.828 | 3.91E-03 |
| ENSGALG00010021110 | PKLR | 384,539 | 442,646 | 326,431 | 1.356 | 1.21E-03 |
| ENSGALG00010021116 | FAM21C | 1,316 | 1,258 | 1,375 | 0.915 | 4.33E-02 |
| ENSGALG00010021125 | LY75 | 6,244 | 5,354 | 7,134 | 0.75 | 4.92E-03 |
| ENSGALG00010021126 | ECE1 | 668 | 712 | 624 | 1.139 | 3.36E-02 |
| ENSGALG00010021129 | TXNRD3 | 1,072 | 1,158 | 987 | 1.176 | 4.58E-03 |
| ENSGALG00010021134 | CHFR | 701 | 619 | 784 | 0.79 | 4.10E-03 |
| ENSGALG00010021147 | ARID3B | 324 | 349 | 300 | 1.167 | 1.68E-02 |
| ENSGALG00010021154 | CHST13 | 93 | 72 | 114 | 0.626 | 2.56E-03 |
| ENSGALG00010021157 |  | 75 | 55 | 94 | 0.584 | 9.89E-03 |
| ENSGALG00010021158 | ACTG1 | 1,512 | 1,360 | 1,664 | 0.817 | 9.57E-04 |
| ENSGALG00010021159 | MMD2 | 1,299 | 1,122 | 1,475 | 0.761 | 5.28E-03 |
| ENSGALG00010021165 | PTRF | 11,792 | 10,797 | 12,787 | 0.844 | 9.07E-03 |
| ENSGALG00010021167 | KIAA1671 | 1,321 | 916 | 1,726 | 0.53 | 2.32E-04 |
| ENSGALG00010021168 | IVNS1ABP | 3,933 | 3,737 | 4,129 | 0.905 | 4.40E-02 |
| ENSGALG00010021170 | UBL7 | 334 | 304 | 363 | 0.833 | 1.28E-02 |
| ENSGALG00010021173 | KLF15 | 794 | 887 | 701 | 1.267 | 2.80E-02 |
| ENSGALG00010021181 | DAGLA | 1,018 | 1,175 | 861 | 1.365 | 1.29E-02 |
| ENSGALG00010021183 | CFAP100 | 30 | 39 | 20 | 2.001 | 1.76E-04 |
| ENSGALG00010021190 |  | 807 | 857 | 757 | 1.136 | 2.85E-02 |
| ENSGALG00010021192 | NTL | 92 | 112 | 73 | 1.545 | 7.61E-04 |
| ENSGALG00010021194 | CYP11A1 | 9 | 6 | 12 | 0.563 | 4.39E-02 |
| ENSGALG00010021218 | DPF3 | 168 | 187 | 148 | 1.276 | 9.79E-03 |
| ENSGALG00010021229 | GLO1 | 25,595 | 28,832 | 22,357 | 1.29 | 4.59E-03 |
| ENSGALG00010021241 | TMEM43 | 1,085 | 904 | 1,266 | 0.713 | 2.63E-05 |
| ENSGALG00010021242 | BCAR3 | 722 | 536 | 908 | 0.59 | 4.97E-05 |
| ENSGALG00010021244 | PPP3CB | 2,913 | 3,107 | 2,720 | 1.142 | 1.94E-02 |
| ENSGALG00010021248 | ANXA7 | 798 | 538 | 1,058 | 0.508 | 1.17E-07 |
| ENSGALG00010021253 | TMEM169 | 223 | 246 | 201 | 1.219 | 2.83E-02 |
| ENSGALG00010021261 | JUP | 1,779 | 1,576 | 1,983 | 0.794 | 5.38E-03 |
| ENSGALG00010021266 | COQ5 | 733 | 807 | 660 | 1.224 | 2.25E-02 |
| *ENSGALG00010021296* | *GATC* | *298* | *238* | *358* | *0.665* | *1.76E-03* |
| ENSGALG00010021298 | RPGRIP1L | 69 | 76 | 62 | 1.249 | 3.38E-02 |
| ENSGALG00010021325 | ARPC1A | 2,738 | 2,186 | 3,290 | 0.665 | 3.31E-04 |
| ENSGALG00010021330 | COMMD4 | 277 | 229 | 325 | 0.703 | 4.63E-02 |
| ENSGALG00010021353 | IPO13 | 790 | 654 | 926 | 0.706 | 1.25E-06 |
| ENSGALG00010021358 | MMP15 | 4,973 | 4,381 | 5,566 | 0.787 | 2.03E-02 |
| ENSGALG00010021360 | PTPRN | 36 | 22 | 50 | 0.439 | 1.24E-04 |
| ENSGALG00010021361 | WRAP73 | 190 | 203 | 177 | 1.149 | 1.37E-02 |
| ENSGALG00010021362 | ARPC1B | 744 | 578 | 909 | 0.636 | 2.49E-03 |
| ENSGALG00010021378 | KIF11 | 318 | 358 | 278 | 1.286 | 4.73E-03 |
| ENSGALG00010021383 | MSI1 | 1,084 | 1,231 | 936 | 1.316 | 1.84E-03 |
| ENSGALG00010021387 | SLC2A14 | 1,301 | 1,103 | 1,499 | 0.735 | 6.77E-07 |
| ENSGALG00010021388 | DPH2 | 65 | 54 | 76 | 0.719 | 2.98E-02 |
| ENSGALG00010021390 | GSTO2 | 208 | 167 | 250 | 0.665 | 8.99E-03 |
| ENSGALG00010021391 |  | 576 | 339 | 814 | 0.417 | 1.57E-07 |
| ENSGALG00010021400 | B4GALT2 | 3,737 | 3,360 | 4,115 | 0.817 | 1.27E-02 |
| ENSGALG00010021408 | TTC9 | 159 | 130 | 188 | 0.691 | 4.62E-02 |
| ENSGALG00010021411 | MCU | 6,639 | 5,609 | 7,669 | 0.731 | 5.22E-09 |
| ENSGALG00010021420 | PLXNB3 | 720 | 877 | 564 | 1.556 | 8.09E-06 |
| ENSGALG00010021421 | GLB1L | 92 | 78 | 105 | 0.742 | 2.86E-02 |
| ENSGALG00010021423 |  | 5 | 3 | 7 | 0.474 | 4.72E-02 |
| ENSGALG00010021426 | SLC38A7 | 955 | 764 | 1,147 | 0.666 | 2.27E-06 |
| ENSGALG00010021427 | RPLP0 | 22,876 | 20,434 | 25,319 | 0.807 | 3.97E-02 |
| ENSGALG00010021429 | MED8 | 495 | 445 | 544 | 0.819 | 2.72E-02 |
| ENSGALG00010021441 |  | 65 | 55 | 75 | 0.731 | 4.86E-02 |
| ENSGALG00010021447 | OCSTAMP | 52 | 28 | 76 | 0.364 | 1.21E-03 |
| ENSGALG00010021450 | OLFML1 | 257 | 209 | 305 | 0.684 | 1.99E-03 |
| ENSGALG00010021462 | SETD5 | 908 | 971 | 845 | 1.148 | 1.27E-02 |
| ENSGALG00010021463 |  | 5 | 3 | 8 | 0.328 | 2.98E-02 |
| ENSGALG00010021481 | MICU1 | 945 | 803 | 1,087 | 0.738 | 3.17E-03 |
| ENSGALG00010021493 | UNC93B1 | 95 | 79 | 112 | 0.703 | 3.94E-02 |
| ENSGALG00010021501 | SLC23A3 | 9 | 12 | 7 | 1.819 | 3.14E-02 |
| ENSGALG00010021524 |  | 64 | 51 | 76 | 0.674 | 1.13E-03 |
| ENSGALG00010021526 | KCNJ12 | 1,276 | 1,752 | 799 | 2.193 | 3.00E-07 |
| ENSGALG00010021535 | SUSD6 | 1,438 | 1,575 | 1,300 | 1.212 | 3.80E-02 |
| ENSGALG00010021538 | TNFRSF18 | 62 | 46 | 79 | 0.576 | 4.69E-02 |
| ENSGALG00010021541 | CYP27A1 | 149 | 119 | 180 | 0.663 | 1.56E-02 |
| ENSGALG00010021550 | MAP2K3 | 1,887 | 1,683 | 2,091 | 0.805 | 8.97E-04 |
| ENSGALG00010021554 | SNX11 | 1,108 | 996 | 1,220 | 0.816 | 2.61E-02 |
| ENSGALG00010021566 |  | 278 | 299 | 257 | 1.16 | 3.06E-02 |
| ENSGALG00010021572 | MYL9 | 1,867 | 1,407 | 2,327 | 0.605 | 5.02E-07 |
| ENSGALG00010021573 | NATD1 | 1,131 | 1,018 | 1,244 | 0.819 | 2.37E-02 |
| ENSGALG00010021574 | TTLL4 | 779 | 636 | 921 | 0.691 | 2.98E-02 |
| ENSGALG00010021583 | NFE2L1 | 9,771 | 8,437 | 11,105 | 0.76 | 2.75E-05 |
| ENSGALG00010021593 | DCAF7 | 555 | 591 | 519 | 1.139 | 3.39E-03 |
| ENSGALG00010021599 | ARPC4 | 807 | 715 | 899 | 0.796 | 4.69E-02 |
| ENSGALG00010021611 | GREM1 | 128 | 100 | 156 | 0.642 | 3.80E-04 |
| ENSGALG00010021617 | SORBS1 | 5,039 | 3,860 | 6,217 | 0.621 | 1.98E-06 |
| ENSGALG00010021622 | PTHLH | 91 | 65 | 117 | 0.559 | 8.97E-04 |
| ENSGALG00010021623 | FAR1 | 5,252 | 5,679 | 4,825 | 1.177 | 4.53E-02 |
| ENSGALG00010021626 | ZNF142 | 348 | 378 | 317 | 1.194 | 4.33E-02 |
| ENSGALG00010021629 | DHRS7B | 2,422 | 2,625 | 2,220 | 1.183 | 1.59E-02 |
| ENSGALG00010021638 | SRC | 156 | 132 | 181 | 0.726 | 4.20E-02 |
| ENSGALG00010021647 | ARHGAP11B | 279 | 344 | 214 | 1.605 | 5.22E-09 |
| ENSGALG00010021649 | PXN | 5,987 | 5,148 | 6,826 | 0.754 | 1.14E-05 |
| ENSGALG00010021650 | CD276 | 395 | 335 | 454 | 0.738 | 7.10E-04 |
| ENSGALG00010021658 | STRADA | 1,654 | 1,735 | 1,573 | 1.104 | 4.66E-02 |
| ENSGALG00010021663 | CNOT9 | 2,500 | 2,312 | 2,688 | 0.86 | 2.64E-02 |
| ENSGALG00010021677 |  | 1,160 | 1,317 | 1,003 | 1.314 | 4.73E-06 |
| ENSGALG00010021680 | PIGT | 530 | 494 | 565 | 0.876 | 7.01E-03 |
| ENSGALG00010021681 | BTBD10 | 1,333 | 1,174 | 1,491 | 0.787 | 1.41E-02 |
| ENSGALG00010021695 | FNBP4 | 588 | 631 | 544 | 1.16 | 2.83E-02 |
| ENSGALG00010021698 | GYPC | 1,195 | 1,316 | 1,074 | 1.225 | 1.33E-02 |
| ENSGALG00010021717 | SDC4 | 431 | 358 | 504 | 0.71 | 1.45E-02 |
| ENSGALG00010021721 | TP53I11 | 617 | 710 | 525 | 1.353 | 6.02E-06 |
| ENSGALG00010021726 | ARNTL | 316 | 344 | 288 | 1.189 | 3.94E-02 |
| ENSGALG00010021731 | SRM | 680 | 724 | 637 | 1.14 | 6.61E-03 |
| ENSGALG00010021734 | PARD3B | 726 | 817 | 636 | 1.286 | 2.49E-02 |
| ENSGALG00010021741 | RPL19 | 13,938 | 12,488 | 15,387 | 0.812 | 2.92E-02 |
| ENSGALG00010021754 | SLC17A9 | 10 | 7 | 14 | 0.461 | 2.33E-02 |
| ENSGALG00010021760 | CSTA | 226 | 150 | 302 | 0.497 | 9.84E-03 |
| ENSGALG00010021779 | UNC13C | 103 | 114 | 92 | 1.237 | 3.12E-02 |
| ENSGALG00010021788 | RPL5 | 34,496 | 31,742 | 37,250 | 0.852 | 3.28E-02 |
| ENSGALG00010021792 | KLHDC8B | 47 | 32 | 62 | 0.515 | 6.86E-03 |
| ENSGALG00010021829 | ERCC6 | 668 | 739 | 596 | 1.24 | 6.16E-05 |
| ENSGALG00010021837 |  | 30 | 41 | 19 | 2.192 | 4.25E-03 |
| ENSGALG00010021848 | LARGE2 | 1,416 | 1,137 | 1,696 | 0.67 | 1.37E-04 |
| ENSGALG00010021867 | CXCL12 | 2,605 | 2,954 | 2,256 | 1.309 | 3.84E-04 |
| ENSGALG00010021869 | GJC3 | 357 | 399 | 316 | 1.259 | 4.57E-03 |
| ENSGALG00010021903 | MORC1 | 873 | 816 | 930 | 0.877 | 4.37E-02 |
| ENSGALG00010021928 |  | 79 | 128 | 31 | 4.156 | 4.37E-04 |
| ENSGALG00010021931 | ITGA6 | 5,471 | 4,589 | 6,354 | 0.722 | 5.97E-04 |
| ENSGALG00010021935 | CETP | 2,345 | 1,795 | 2,896 | 0.62 | 3.16E-03 |
| ENSGALG00010021953 | PITPNM1 | 153 | 123 | 183 | 0.676 | 2.69E-02 |
| ENSGALG00010021989 | NCAPD2 | 327 | 348 | 306 | 1.135 | 2.18E-02 |
| ENSGALG00010022024 | MICAL2 | 1,946 | 2,143 | 1,748 | 1.226 | 1.69E-02 |
| ENSGALG00010022028 | FGD5 | 701 | 763 | 640 | 1.193 | 4.95E-02 |
| ENSGALG00010022030 | ZNF341 | 115 | 124 | 106 | 1.176 | 2.89E-02 |
| ENSGALG00010022041 | MAPK8IP1 | 501 | 411 | 591 | 0.695 | 3.21E-05 |
| ENSGALG00010022044 | PXMP4 | 1,184 | 1,117 | 1,251 | 0.894 | 4.37E-02 |
| ENSGALG00010022051 | BAIAP2L1 | 153 | 119 | 187 | 0.631 | 7.62E-05 |
| ENSGALG00010022066 | DAD1 | 793 | 711 | 874 | 0.813 | 1.44E-02 |
| ENSGALG00010022108 | USP47 | 4,635 | 4,351 | 4,919 | 0.885 | 3.27E-02 |
| ENSGALG00010022115 | E2F4 | 659 | 700 | 619 | 1.13 | 2.72E-02 |
| ENSGALG00010022116 | CYP20A1 | 280 | 260 | 301 | 0.861 | 3.40E-02 |
| ENSGALG00010022124 |  | 569 | 483 | 655 | 0.738 | 5.44E-03 |
| ENSGALG00010022172 | GIPC2 | 533 | 407 | 659 | 0.619 | 2.23E-02 |
| ENSGALG00010022179 |  | 1 | 2 | 1 | 3.872 | 2.32E-02 |
| ENSGALG00010022203 | DNAAF5 | 311 | 405 | 218 | 1.865 | 4.22E-15 |
| ENSGALG00010022208 | CCL17 | 10 | 6 | 13 | 0.438 | 2.30E-02 |
| ENSGALG00010022210 | EZH1 | 649 | 616 | 682 | 0.901 | 3.21E-02 |
| ENSGALG00010022213 | IDH1 | 2,330 | 2,159 | 2,500 | 0.864 | 2.56E-03 |
| ENSGALG00010022219 | EIF4E3 | 813 | 683 | 942 | 0.725 | 2.10E-03 |
| ENSGALG00010022222 |  | 108 | 121 | 94 | 1.279 | 4.24E-02 |
| ENSGALG00010022223 | DOK4 | 85 | 53 | 117 | 0.453 | 4.40E-06 |
| ENSGALG00010022229 | RAPSN | 764 | 841 | 686 | 1.227 | 3.44E-02 |
| ENSGALG00010022230 | NFS1 | 1,468 | 1,560 | 1,376 | 1.133 | 2.69E-02 |
| ENSGALG00010022231 | ZNF503 | 34 | 28 | 40 | 0.681 | 1.94E-02 |
| ENSGALG00010022236 | SHF | 3,851 | 3,421 | 4,280 | 0.799 | 2.13E-03 |
| ENSGALG00010022274 | CCDC91 | 275 | 309 | 242 | 1.274 | 4.44E-04 |
| ENSGALG00010022280 | PDZD8 | 620 | 673 | 566 | 1.187 | 2.19E-03 |
| ENSGALG00010022282 | METTL7A | 343 | 286 | 400 | 0.715 | 2.23E-02 |
| ENSGALG00010022293 | DNAJC10 | 1,223 | 1,291 | 1,156 | 1.118 | 4.18E-02 |
| ENSGALG00010022303 | DUSP13A | 2,221 | 1,770 | 2,673 | 0.662 | 8.97E-07 |
| ENSGALG00010022304 | ADAP1 | 101 | 83 | 119 | 0.693 | 8.86E-03 |
| *ENSGALG00010022311* | *DLL4* | *299* | *345* | *254* | *1.358* | *1.28E-04* |
| ENSGALG00010022327 |  | 58 | 67 | 49 | 1.403 | 2.71E-02 |
| ENSGALG00010022345 | USP33 | 578 | 611 | 545 | 1.12 | 3.18E-02 |
| ENSGALG00010022347 | SEC11A | 309 | 278 | 340 | 0.818 | 9.13E-03 |
| ENSGALG00010022353 | SLC39A13 | 3,622 | 3,333 | 3,912 | 0.852 | 4.55E-06 |
| ENSGALG00010022356 | TCFL5 | 20 | 24 | 16 | 1.492 | 2.31E-02 |
| ENSGALG00010022373 | AK5 | 12 | 16 | 8 | 2.076 | 6.77E-03 |
| ENSGALG00010022392 | EMILIN3 | 262 | 177 | 347 | 0.512 | 8.06E-03 |
| ENSGALG00010022401 | LCAT | 209 | 145 | 272 | 0.535 | 3.06E-02 |
| ENSGALG00010022421 | CMKLR1 | 475 | 377 | 573 | 0.657 | 2.01E-02 |
| ENSGALG00010022429 | ADK | 1,291 | 1,410 | 1,171 | 1.203 | 6.71E-03 |
| ENSGALG00010022433 | LNPK | 2,413 | 2,196 | 2,631 | 0.834 | 1.87E-02 |
| ENSGALG00010022435 | PIGK | 1,621 | 1,321 | 1,922 | 0.687 | 4.51E-04 |
| ENSGALG00010022436 | AFAP1L2 | 742 | 641 | 843 | 0.76 | 2.44E-02 |
| ENSGALG00010022438 | RPN2 | 2,220 | 2,074 | 2,366 | 0.876 | 1.47E-02 |
| ENSGALG00010022439 | IFI35 | 482 | 416 | 547 | 0.762 | 2.63E-03 |
| ENSGALG00010022449 |  | 603 | 519 | 686 | 0.757 | 2.14E-02 |
| ENSGALG00010022459 | FGF6 | 11 | 7 | 15 | 0.451 | 1.32E-02 |
| ENSGALG00010022462 | ITGA5 | 264 | 179 | 350 | 0.512 | 5.84E-05 |
| ENSGALG00010022468 | MYBPC3 | 3,504 | 2,276 | 4,732 | 0.481 | 3.18E-04 |
| ENSGALG00010022474 | VCL | 10,512 | 9,381 | 11,643 | 0.806 | 3.10E-03 |
| ENSGALG00010022478 | ATP6V1A | 1,494 | 1,377 | 1,610 | 0.855 | 4.34E-02 |
| ENSGALG00010022479 |  | 76 | 52 | 101 | 0.518 | 3.08E-02 |
| ENSGALG00010022489 | NUSAP1 | 210 | 236 | 184 | 1.279 | 6.67E-03 |
| ENSGALG00010022498 | NAA50 | 15,987 | 17,109 | 14,864 | 1.151 | 1.67E-02 |
| ENSGALG00010022516 | TRUB1 | 205 | 184 | 225 | 0.821 | 3.57E-02 |
| ENSGALG00010022524 | SOX18 | 129 | 144 | 114 | 1.265 | 1.65E-02 |
| ENSGALG00010022538 | WIPF1 | 808 | 906 | 709 | 1.277 | 1.86E-02 |
| ENSGALG00010022552 | GPR155 | 3,482 | 3,767 | 3,196 | 1.179 | 4.90E-02 |
| ENSGALG00010022553 | PRPF6 | 2,447 | 2,562 | 2,332 | 1.099 | 2.50E-02 |
| ENSGALG00010022563 |  | 3 | 1 | 4 | 0.273 | 2.31E-02 |
| ENSGALG00010022608 | P3H4 | 152 | 135 | 169 | 0.8 | 4.99E-02 |
| ENSGALG00010022622 | MPPED2 | 310 | 276 | 345 | 0.804 | 1.63E-02 |
| ENSGALG00010022625 |  | 316 | 282 | 349 | 0.806 | 4.25E-02 |
| ENSGALG00010022627 | CTCF | 2,246 | 2,369 | 2,123 | 1.117 | 2.88E-02 |
| ENSGALG00010022650 | NR1H3 | 3,469 | 3,773 | 3,164 | 1.193 | 1.58E-04 |
| ENSGALG00010022659 | TIE1 | 919 | 1,017 | 822 | 1.237 | 1.57E-03 |
| ENSGALG00010022671 | DBR1 | 1,524 | 1,627 | 1,422 | 1.146 | 1.71E-02 |
| ENSGALG00010022677 |  | 1,613 | 1,748 | 1,477 | 1.184 | 1.80E-02 |
| ENSGALG00010022695 | PIK3IP1 | 1,050 | 958 | 1,142 | 0.839 | 2.15E-02 |
| ENSGALG00010022708 | SPRED1 | 916 | 812 | 1,019 | 0.796 | 1.63E-05 |
| ENSGALG00010022715 |  | 415 | 452 | 378 | 1.195 | 2.35E-02 |
| ENSGALG00010022717 | ELOVL1 | 1,040 | 774 | 1,306 | 0.593 | 7.41E-07 |
| ENSGALG00010022719 | MSL2 | 630 | 674 | 586 | 1.152 | 6.89E-03 |
| ENSGALG00010022724 | LUZP2 | 38 | 25 | 52 | 0.476 | 4.24E-02 |
| ENSGALG00010022727 | ACP2 | 659 | 717 | 602 | 1.193 | 3.54E-03 |
| ENSGALG00010022751 | SLC25A12 | 11,568 | 13,299 | 9,838 | 1.352 | 1.10E-02 |
| ENSGALG00010022755 | MINPP1 | 1,303 | 884 | 1,721 | 0.513 | 4.94E-06 |
| ENSGALG00010022768 |  | 38 | 48 | 28 | 1.711 | 4.92E-02 |
| ENSGALG00010022769 | FXYD6 | 3,393 | 2,827 | 3,959 | 0.714 | 2.66E-04 |
| ENSGALG00010022776 | CDH4 | 386 | 422 | 351 | 1.204 | 2.35E-02 |
| ENSGALG00010022785 |  | 56 | 65 | 47 | 1.383 | 1.84E-02 |
| ENSGALG00010022792 | DDB2 | 269 | 240 | 298 | 0.804 | 2.86E-02 |
| ENSGALG00010022810 |  | 49 | 61 | 38 | 1.605 | 1.65E-02 |
| ENSGALG00010022815 | LRRC8D | 547 | 476 | 619 | 0.769 | 3.86E-03 |
| ENSGALG00010022819 | PACSIN3 | 10,314 | 7,805 | 12,823 | 0.609 | 4.70E-07 |
| ENSGALG00010022827 | CMTR2 | 501 | 569 | 434 | 1.31 | 3.28E-04 |
| ENSGALG00010022831 | LRRC8C | 183 | 205 | 161 | 1.268 | 6.29E-03 |
| ENSGALG00010022844 |  | 7 | 4 | 10 | 0.359 | 2.89E-02 |
| ENSGALG00010022863 | FAM81A | 45 | 29 | 61 | 0.469 | 1.19E-03 |
| ENSGALG00010022873 | NGEF | 160 | 112 | 209 | 0.533 | 1.10E-03 |
| ENSGALG00010022877 | KYAT3 | 617 | 559 | 675 | 0.829 | 2.94E-02 |
| ENSGALG00010022878 |  | 182 | 134 | 230 | 0.58 | 3.19E-04 |
| ENSGALG00010022885 | M6PR | 3,947 | 3,358 | 4,535 | 0.74 | 1.96E-12 |
| ENSGALG00010022923 | CD63 | 5,283 | 4,243 | 6,323 | 0.671 | 1.06E-05 |
| ENSGALG00010022925 |  | 38 | 45 | 31 | 1.425 | 9.79E-03 |
| ENSGALG00010022942 | NEU2 | 35 | 25 | 46 | 0.552 | 4.39E-03 |
| ENSGALG00010022956 | PBLD | 219 | 263 | 176 | 1.492 | 1.09E-03 |
| ENSGALG00010022957 | SLTM | 1,456 | 1,600 | 1,311 | 1.221 | 1.28E-05 |
| ENSGALG00010022965 |  | 488 | 219 | 757 | 0.289 | 1.85E-04 |
| ENSGALG00010022966 |  | 34 | 23 | 45 | 0.502 | 4.11E-04 |
| ENSGALG00010022992 | SLC7A6 | 790 | 713 | 867 | 0.822 | 1.19E-02 |
| ENSGALG00010023007 | LMO4 | 1,825 | 1,701 | 1,948 | 0.874 | 2.62E-02 |
| ENSGALG00010023017 | BUB1B | 171 | 191 | 151 | 1.261 | 1.21E-02 |
| ENSGALG00010023043 | CNP | 867 | 653 | 1,082 | 0.603 | 2.31E-07 |
| ENSGALG00010023055 |  | 47 | 24 | 71 | 0.338 | 1.19E-03 |
| ENSGALG00010023058 | PPARA | 248 | 284 | 212 | 1.342 | 5.01E-03 |
| ENSGALG00010023061 |  | 15 | 19 | 10 | 1.975 | 6.13E-03 |
| ENSGALG00010023062 | STX2 | 3,667 | 3,468 | 3,866 | 0.897 | 4.93E-02 |
| ENSGALG00010023070 | SIDT2 | 1,567 | 1,462 | 1,671 | 0.875 | 3.28E-02 |
| ENSGALG00010023073 | POMGNT1 | 1,202 | 1,087 | 1,317 | 0.826 | 5.63E-04 |
| ENSGALG00010023079 | SLC25A16 | 887 | 956 | 819 | 1.169 | 3.74E-02 |
| ENSGALG00010023100 |  | 22 | 31 | 13 | 2.347 | 1.01E-04 |
| ENSGALG00010023113 | MYZAP | 177 | 203 | 151 | 1.334 | 2.66E-03 |
| ENSGALG00010023125 | COL20A1 | 629 | 746 | 512 | 1.458 | 2.09E-04 |
| ENSGALG00010023131 | SOX6 | 375 | 441 | 309 | 1.423 | 4.66E-03 |
| ENSGALG00010023136 | MDK | 70 | 42 | 98 | 0.428 | 3.73E-03 |
| ENSGALG00010023142 | TLK2 | 2,910 | 3,242 | 2,579 | 1.258 | 9.81E-03 |
| ENSGALG00010023157 | STOX1 | 12 | 14 | 9 | 1.601 | 4.56E-02 |
| ENSGALG00010023162 | CHRNA4 | 66 | 75 | 57 | 1.322 | 4.60E-02 |
| ENSGALG00010023172 | DHX58 | 81 | 70 | 91 | 0.769 | 1.44E-02 |
| ENSGALG00010023181 | PLEKHA7 | 324 | 377 | 270 | 1.398 | 1.63E-04 |
| ENSGALG00010023190 | SF3B3 | 2,494 | 2,600 | 2,387 | 1.089 | 1.86E-02 |
| ENSGALG00010023198 | PTK6 | 8 | 10 | 5 | 2.062 | 4.28E-02 |
| ENSGALG00010023202 | PID1 | 187 | 154 | 221 | 0.696 | 1.60E-02 |
| ENSGALG00010023212 | RIMBP2 | 1,570 | 1,788 | 1,352 | 1.322 | 4.02E-02 |
| ENSGALG00010023219 | TEX9 | 258 | 298 | 217 | 1.377 | 7.42E-04 |
| ENSGALG00010023225 | PDPR | 394 | 442 | 346 | 1.278 | 1.41E-02 |
| ENSGALG00010023238 | NUCB2 | 3,618 | 3,276 | 3,960 | 0.827 | 1.10E-03 |
| ENSGALG00010023251 |  | 64 | 51 | 78 | 0.655 | 4.25E-02 |
| ENSGALG00010023257 | PAK6 | 17 | 21 | 13 | 1.601 | 2.69E-02 |
| ENSGALG00010023259 | TSPAN15 | 268 | 243 | 293 | 0.826 | 2.98E-02 |
| ENSGALG00010023263 | SRP14 | 896 | 834 | 957 | 0.872 | 5.87E-03 |
| ENSGALG00010023266 | RFWD3 | 135 | 150 | 119 | 1.259 | 3.95E-03 |
| ENSGALG00010023281 | B2M | 22,864 | 19,799 | 25,928 | 0.764 | 4.29E-02 |
| ENSGALG00010023304 |  | 308 | 234 | 383 | 0.612 | 1.21E-02 |
| ENSGALG00010023322 | PRKAB1 | 960 | 757 | 1,163 | 0.651 | 2.77E-06 |
| ENSGALG00010023331 | CD4 | 45 | 34 | 57 | 0.587 | 2.54E-03 |
| ENSGALG00010023340 | P3H3 | 127 | 137 | 117 | 1.165 | 4.92E-02 |
| ENSGALG00010023343 | TMEM233 | 574 | 427 | 720 | 0.594 | 1.56E-04 |
| ENSGALG00010023362 | GADD45A | 1,297 | 1,120 | 1,474 | 0.76 | 1.61E-02 |
| ENSGALG00010023363 | CPEB1 | 126 | 98 | 155 | 0.627 | 5.65E-05 |
| ENSGALG00010023380 | USP5 | 2,275 | 2,077 | 2,473 | 0.84 | 9.51E-04 |
| ENSGALG00010023389 | GNG12 | 556 | 525 | 587 | 0.893 | 2.41E-02 |
| ENSGALG00010023392 | TPI1 | 208,724 | 242,044 | 175,405 | 1.38 | 4.14E-03 |
| ENSGALG00010023394 | HOXB5 | 57 | 65 | 49 | 1.351 | 2.26E-03 |
| ENSGALG00010023422 | WLS | 1,115 | 910 | 1,320 | 0.689 | 7.86E-04 |
| ENSGALG00010023434 | ATN1 | 281 | 240 | 322 | 0.744 | 1.38E-02 |
| ENSGALG00010023437 | KCNJ5 | 51 | 37 | 65 | 0.575 | 2.56E-02 |
| ENSGALG00010023441 | CCDC9B | 319 | 249 | 390 | 0.636 | 2.35E-02 |
| ENSGALG00010023447 | C12orf57 | 129 | 95 | 163 | 0.583 | 7.82E-03 |
| ENSGALG00010023473 | BOK | 165 | 180 | 149 | 1.204 | 2.81E-02 |
| ENSGALG00010023489 | ETV5 | 626 | 497 | 755 | 0.659 | 6.46E-05 |
| ENSGALG00010023520 |  | 41 | 52 | 30 | 1.74 | 3.59E-04 |
| ENSGALG00010023523 |  | 2 | 4 | 1 | 4.204 | 8.86E-03 |
| ENSGALG00010023560 |  | 7 | 5 | 9 | 0.541 | 2.31E-02 |
| ENSGALG00010023569 | LPCAT3 | 1,035 | 964 | 1,106 | 0.873 | 4.29E-02 |
| ENSGALG00010023592 | BAHD1 | 940 | 859 | 1,021 | 0.842 | 2.44E-02 |
| ENSGALG00010023597 | MOGAT1 | 475 | 377 | 574 | 0.656 | 5.58E-04 |
| ENSGALG00010023613 | COPB1 | 1,831 | 1,685 | 1,977 | 0.852 | 4.12E-04 |
| ENSGALG00010023620 | IRS1 | 1,228 | 1,338 | 1,117 | 1.198 | 4.79E-02 |
| ENSGALG00010023621 | CD79B | 9 | 5 | 13 | 0.388 | 1.08E-02 |
| ENSGALG00010023636 | PDE3B | 1,547 | 1,724 | 1,371 | 1.258 | 1.97E-04 |
| ENSGALG00010023639 | KLHL23 | 385 | 279 | 492 | 0.567 | 8.15E-04 |
| ENSGALG00010023663 | PHOSPHO2 | 405 | 464 | 346 | 1.34 | 2.07E-03 |
| ENSGALG00010023693 | SRSF11 | 2,075 | 2,177 | 1,972 | 1.104 | 5.73E-03 |
| ENSGALG00010023711 |  | 3 | 1 | 5 | 0.292 | 1.75E-02 |
| ENSGALG00010023712 | ARHGAP40 | 897 | 636 | 1,158 | 0.549 | 1.66E-05 |
| ENSGALG00010023743 | CD3D | 163 | 125 | 201 | 0.619 | 8.26E-03 |
| ENSGALG00010023748 |  | 108 | 85 | 130 | 0.651 | 2.34E-03 |
| ENSGALG00010023760 | ZW10 | 597 | 642 | 551 | 1.169 | 2.27E-03 |
| *ENSGALG00010023766* |  | *353* | *103* | *604* | *0.17* | *3.77E-08* |
| ENSGALG00010023773 | OVOA | 17 | 27 | 7 | 3.728 | 1.35E-08 |
| ENSGALG00010023791 | VTI1B | 426 | 482 | 371 | 1.298 | 2.70E-02 |
| ENSGALG00010023798 | ARCN1 | 2,227 | 2,131 | 2,322 | 0.917 | 1.87E-03 |
| ENSGALG00010023818 | ALOX5 | 114 | 91 | 137 | 0.665 | 1.48E-03 |
| ENSGALG00010023820 | CYP1C1 | 21 | 11 | 30 | 0.36 | 1.24E-02 |
| ENSGALG00010023829 | USP1 | 772 | 819 | 724 | 1.132 | 1.96E-02 |
| ENSGALG00010023831 | SATB2 | 61 | 87 | 35 | 2.492 | 2.26E-04 |
| ENSGALG00010023838 |  | 1,759 | 2,116 | 1,401 | 1.511 | 7.16E-03 |
| ENSGALG00010023861 | SLC2A11L5 | 118 | 135 | 101 | 1.335 | 5.94E-03 |
| ENSGALG00010023862 | TRMT13 | 159 | 140 | 177 | 0.793 | 3.16E-02 |
| ENSGALG00010023863 | EYA2 | 74 | 61 | 88 | 0.691 | 2.71E-02 |
| ENSGALG00010023920 |  | 49 | 66 | 32 | 2.043 | 2.05E-06 |
| ENSGALG00010023926 | ZYX | 1,352 | 1,112 | 1,592 | 0.698 | 4.89E-03 |
| ENSGALG00010023927 | KPNB1 | 4,898 | 4,619 | 5,178 | 0.892 | 1.39E-02 |
| ENSGALG00010023931 | CSRNP2 | 30 | 35 | 25 | 1.455 | 3.83E-02 |
| ENSGALG00010023945 | LETMD1 | 181 | 202 | 160 | 1.261 | 4.44E-02 |
| ENSGALG00010023946 | ZNRF3 | 137 | 157 | 116 | 1.356 | 1.08E-02 |
| ENSGALG00010023948 | SULF2 | 509 | 555 | 463 | 1.195 | 1.08E-02 |
| ENSGALG00010023954 | PLEKHF1 | 470 | 450 | 490 | 0.919 | 4.48E-02 |
| ENSGALG00010023984 | FMNL1 | 139 | 114 | 164 | 0.692 | 2.47E-02 |
| ENSGALG00010023991 | TSPAN3 | 2,413 | 2,304 | 2,522 | 0.913 | 8.06E-03 |
| ENSGALG00010024001 |  | 402 | 527 | 277 | 1.904 | 9.19E-06 |
| ENSGALG00010024030 | STAU1 | 4,892 | 4,455 | 5,328 | 0.836 | 4.61E-04 |
| ENSGALG00010024031 | LIMS2 | 7,065 | 6,248 | 7,882 | 0.793 | 3.20E-04 |
| ENSGALG00010024049 | RCN2 | 1,243 | 1,104 | 1,381 | 0.799 | 1.72E-02 |
| ENSGALG00010024052 |  | 388 | 333 | 443 | 0.753 | 1.25E-04 |
| ENSGALG00010024058 | SLC44A5 | 1,056 | 1,246 | 866 | 1.44 | 2.47E-06 |
| ENSGALG00010024071 |  | 28 | 41 | 16 | 2.648 | 3.60E-06 |
| ENSGALG00010024083 | PALMD | 290 | 320 | 259 | 1.234 | 3.20E-02 |
| ENSGALG00010024086 | GABARAPL1 | 2,899 | 2,561 | 3,237 | 0.791 | 1.17E-04 |
| ENSGALG00010024091 | ETFA | 2,062 | 2,245 | 1,879 | 1.195 | 4.45E-02 |
| ENSGALG00010024132 |  | 107 | 91 | 123 | 0.74 | 1.47E-02 |
| ENSGALG00010024141 | YTHDF1 | 1,195 | 1,252 | 1,138 | 1.1 | 3.82E-02 |
| ENSGALG00010024142 | DNAJC6 | 151 | 110 | 193 | 0.568 | 1.30E-06 |
| ENSGALG00010024149 | TCIRG1 | 243 | 189 | 297 | 0.635 | 4.32E-03 |
| ENSGALG00010024152 | ZNF423 | 1,071 | 1,145 | 997 | 1.15 | 1.67E-02 |
| ENSGALG00010024154 | VPS29L | 11 | 6 | 16 | 0.357 | 5.85E-04 |
| ENSGALG00010024179 | ATG4C | 461 | 505 | 417 | 1.214 | 2.26E-04 |
| ENSGALG00010024184 |  | 137 | 116 | 157 | 0.737 | 3.46E-03 |
| ENSGALG00010024185 | CHRNA5 | 325 | 368 | 281 | 1.311 | 2.47E-02 |
| ENSGALG00010024201 | IREB2 | 3,264 | 3,509 | 3,020 | 1.162 | 3.38E-04 |
| ENSGALG00010024205 | ANKRD13D | 199 | 150 | 248 | 0.606 | 7.05E-04 |
| ENSGALG00010024206 | KRT80 | 14 | 7 | 20 | 0.332 | 1.25E-03 |
| ENSGALG00010024220 | SIAH1 | 3,692 | 4,407 | 2,977 | 1.481 | 5.18E-04 |
| ENSGALG00010024222 | KCNK15 | 22 | 17 | 27 | 0.615 | 2.89E-03 |
| ENSGALG00010024254 |  | 17 | 11 | 22 | 0.512 | 2.93E-04 |
| ENSGALG00010024258 | DNAJA4 | 1,539 | 1,290 | 1,788 | 0.722 | 1.52E-02 |
| ENSGALG00010024263 | PHKB | 65,383 | 73,650 | 57,116 | 1.289 | 1.62E-02 |
| ENSGALG00010024274 | ACSBG1 | 33 | 21 | 46 | 0.456 | 2.76E-04 |
| ENSGALG00010024276 |  | 183 | 200 | 166 | 1.199 | 2.72E-02 |
| ENSGALG00010024277 | ITGB3BP | 89 | 102 | 77 | 1.306 | 1.00E-02 |
| ENSGALG00010024280 | DUSP3 | 5,830 | 5,267 | 6,392 | 0.824 | 3.19E-02 |
| ENSGALG00010024285 | CCND2 | 1,100 | 1,012 | 1,187 | 0.851 | 1.57E-02 |
| ENSGALG00010024288 | CHID1 | 86 | 75 | 97 | 0.779 | 3.40E-02 |
| ENSGALG00010024289 |  | 258 | 161 | 355 | 0.452 | 2.12E-07 |
| ENSGALG00010024300 | MEOX1 | 47 | 33 | 61 | 0.536 | 1.16E-02 |
| ENSGALG00010024309 | ABCB11 | 134 | 84 | 184 | 0.459 | 7.86E-04 |
| ENSGALG00010024313 | SERINC3 | 1,636 | 1,498 | 1,773 | 0.844 | 5.55E-03 |
| ENSGALG00010024314 | ITFG1 | 2,385 | 2,728 | 2,042 | 1.336 | 5.73E-04 |
| ENSGALG00010024322 | IDH3A | 10,444 | 12,134 | 8,755 | 1.386 | 2.07E-03 |
| ENSGALG00010024325 | PCBP2 | 4,213 | 4,482 | 3,945 | 1.136 | 6.04E-03 |
| ENSGALG00010024326 | KLHL24 | 2,752 | 2,379 | 3,125 | 0.761 | 2.34E-02 |
| ENSGALG00010024330 | SDF2L1 | 586 | 535 | 637 | 0.839 | 5.48E-03 |
| ENSGALG00010024333 | ETV4 | 113 | 85 | 142 | 0.602 | 1.33E-02 |
| ENSGALG00010024339 | TOP3B | 658 | 692 | 624 | 1.109 | 4.60E-02 |
| ENSGALG00010024383 | DUSP28 | 111 | 96 | 125 | 0.769 | 3.76E-02 |
| ENSGALG00010024388 |  | 2 | 3 | 1 | 2.581 | 4.41E-02 |
| ENSGALG00010024413 | AMFR | 4,959 | 4,586 | 5,331 | 0.86 | 4.30E-02 |
| ENSGALG00010024419 | PGAP3 | 215 | 183 | 247 | 0.743 | 1.21E-02 |
| ENSGALG00010024454 | HPS5 | 1,112 | 1,167 | 1,057 | 1.104 | 4.20E-02 |
| ENSGALG00010024472 | LDHA | 816,216 | 911,466 | 720,965 | 1.264 | 3.13E-02 |
| ENSGALG00010024473 | RAVER2 | 726 | 675 | 776 | 0.87 | 3.07E-02 |
| ENSGALG00010024480 | CDC45 | 175 | 193 | 156 | 1.229 | 8.63E-03 |
| ENSGALG00010024494 | DDAH1 | 53 | 30 | 76 | 0.391 | 8.64E-06 |
| ENSGALG00010024512 | CCNDBP1 | 805 | 916 | 694 | 1.323 | 4.25E-03 |
| ENSGALG00010024515 | C3orf70 | 161 | 146 | 177 | 0.822 | 3.22E-02 |
| ENSGALG00010024517 | LACTB | 762 | 814 | 711 | 1.149 | 4.93E-02 |
| ENSGALG00010024527 | ATP6V0B | 558 | 491 | 625 | 0.787 | 1.07E-02 |
| ENSGALG00010024530 |  | 29 | 21 | 36 | 0.585 | 2.62E-02 |
| ENSGALG00010024534 | VWF | 4,757 | 5,483 | 4,029 | 1.361 | 2.63E-05 |
| ENSGALG00010024546 | KY | 6,448 | 8,182 | 4,715 | 1.735 | 1.24E-04 |
| ENSGALG00010024549 | GNB1L | 316 | 343 | 289 | 1.186 | 9.51E-03 |
| ENSGALG00010024552 | CD81 | 7,854 | 6,667 | 9,041 | 0.737 | 1.37E-04 |
| ENSGALG00010024554 | ST6GALNAC3 | 55 | 63 | 47 | 1.309 | 4.78E-02 |
| ENSGALG00010024555 | EPHB1 | 877 | 951 | 803 | 1.184 | 2.81E-02 |
| ENSGALG00010024566 | MTSS2 | 324 | 279 | 370 | 0.755 | 1.79E-02 |
| ENSGALG00010024568 | DZIP1L | 26 | 20 | 32 | 0.631 | 3.55E-02 |
| ENSGALG00010024569 | ASCL2 | 9 | 7 | 10 | 0.641 | 4.61E-02 |
| ENSGALG00010024587 |  | 276 | 229 | 323 | 0.71 | 9.90E-03 |
| ENSGALG00010024597 | DNAJB11 | 757 | 716 | 799 | 0.897 | 4.96E-02 |
| ENSGALG00010024605 | LSP1P1 | 2,224 | 1,993 | 2,454 | 0.812 | 4.79E-02 |
| ENSGALG00010024615 | SYT8 | 3 | 2 | 5 | 0.364 | 2.25E-02 |
| ENSGALG00010024619 |  | 392 | 278 | 507 | 0.548 | 4.82E-04 |
| ENSGALG00010024624 | CTSD | 10,468 | 9,317 | 11,619 | 0.802 | 4.46E-04 |
| ENSGALG00010024636 | PANX3 | 34 | 39 | 30 | 1.282 | 4.92E-02 |
| ENSGALG00010024654 | CCDC92 | 239 | 276 | 201 | 1.374 | 2.55E-03 |
| ENSGALG00010024655 | IGF2BP2 | 251 | 202 | 300 | 0.674 | 6.76E-04 |
| ENSGALG00010024671 | KCTD20 | 1,951 | 2,104 | 1,797 | 1.171 | 4.85E-02 |
| ENSGALG00010024683 | SHISA4 | 1,000 | 824 | 1,175 | 0.701 | 1.42E-02 |
| ENSGALG00010024685 |  | 105 | 122 | 87 | 1.394 | 4.66E-03 |
| ENSGALG00010024691 | TCTN2 | 175 | 161 | 188 | 0.856 | 3.75E-02 |
| ENSGALG00010024693 | ANXA2 | 6,641 | 4,576 | 8,707 | 0.525 | 3.05E-04 |
| ENSGALG00010024694 | GTF2H3 | 514 | 477 | 552 | 0.863 | 1.44E-02 |
| ENSGALG00010024695 | TRABD2B | 81 | 65 | 96 | 0.672 | 1.11E-03 |
| ENSGALG00010024699 | FBXL8 | 237 | 215 | 258 | 0.831 | 6.61E-03 |
| ENSGALG00010024700 | TOLLIP | 2,574 | 2,884 | 2,265 | 1.274 | 3.61E-08 |
| *ENSGALG00010024707* |  | *1,041* | *1,599* | *482* | *3.314* | *2.63E-12* |
| ENSGALG00010024710 | HSDL1 | 179 | 161 | 197 | 0.815 | 9.58E-03 |
| ENSGALG00010024717 | TF | 769 | 617 | 921 | 0.669 | 4.50E-02 |
| ENSGALG00010024754 | EIF2S2 | 7,446 | 8,642 | 6,251 | 1.383 | 8.97E-04 |
| ENSGALG00010024755 | PIGX | 145 | 121 | 170 | 0.711 | 1.94E-02 |
| ENSGALG00010024770 | CDC27 | 3,931 | 4,282 | 3,579 | 1.197 | 2.29E-03 |
| ENSGALG00010024773 | APOA1 | 11,930 | 8,903 | 14,957 | 0.595 | 8.12E-04 |
| ENSGALG00010024793 | FKBP4 | 4,213 | 3,876 | 4,550 | 0.852 | 2.74E-02 |
| ENSGALG00010024799 |  | 4,223 | 4,570 | 3,875 | 1.18 | 1.91E-02 |
| ENSGALG00010024808 | CELSR2 | 8 | 5 | 11 | 0.441 | 3.40E-02 |
| ENSGALG00010024810 | ADIPOR1 | 1,766 | 1,649 | 1,884 | 0.875 | 3.55E-02 |
| *ENSGALG00010024811* | *NIF3L1* | *335* | *304* | *365* | *0.835* | *1.56E-02* |
| ENSGALG00010024816 | NFIA | 873 | 949 | 797 | 1.191 | 2.44E-03 |
| ENSGALG00010024833 | MAPT | 2,005 | 1,783 | 2,227 | 0.801 | 2.45E-03 |
| ENSGALG00010024834 | INSIG1 | 2,430 | 2,843 | 2,017 | 1.41 | 1.51E-02 |
| ENSGALG00010024848 | ESYT1 | 628 | 549 | 708 | 0.774 | 3.74E-03 |
| ENSGALG00010024862 | METTL16 | 1,118 | 1,270 | 965 | 1.316 | 6.17E-06 |
| ENSGALG00010024863 | LMOD1 | 114 | 90 | 138 | 0.654 | 2.61E-02 |
| ENSGALG00010024873 | HYOU1 | 2,150 | 1,974 | 2,326 | 0.848 | 7.57E-06 |
| ENSGALG00010024879 | D2HGDH | 686 | 790 | 583 | 1.358 | 4.46E-04 |
| ENSGALG00010024881 | CASP10 | 185 | 167 | 204 | 0.813 | 3.03E-02 |
| ENSGALG00010024903 | CASP8 | 157 | 135 | 179 | 0.752 | 8.04E-03 |
| ENSGALG00010024910 | UBTF | 1,463 | 1,606 | 1,319 | 1.218 | 7.26E-07 |
| ENSGALG00010024911 | CAT | 4,669 | 4,352 | 4,985 | 0.873 | 3.56E-02 |
| ENSGALG00010024914 |  | 65 | 85 | 46 | 1.87 | 1.64E-05 |
| ENSGALG00010024915 | STXBP5L | 104 | 134 | 73 | 1.829 | 2.18E-02 |
| ENSGALG00010024918 | ATG4B | 1,206 | 1,139 | 1,272 | 0.894 | 4.43E-02 |
| ENSGALG00010024921 | STRADB | 995 | 1,091 | 899 | 1.213 | 1.85E-02 |
| ENSGALG00010024925 | GTF2E1 | 534 | 569 | 499 | 1.143 | 4.25E-02 |
| ENSGALG00010024931 | UBE3C | 2,310 | 2,557 | 2,062 | 1.24 | 4.05E-03 |
| ENSGALG00010024935 | KLHL30 | 1,422 | 1,021 | 1,824 | 0.56 | 1.33E-08 |
| ENSGALG00010024942 | DNAJB6 | 2,644 | 2,436 | 2,853 | 0.854 | 1.27E-06 |
| ENSGALG00010024943 | ABCB9 | 102 | 90 | 114 | 0.787 | 9.79E-03 |
| ENSGALG00010024950 | NCAM1 | 3,135 | 2,314 | 3,957 | 0.585 | 4.80E-06 |
| ENSGALG00010024956 | VPS37B | 3,875 | 3,029 | 4,721 | 0.642 | 9.14E-10 |
| ENSGALG00010024986 | PTS | 51 | 41 | 61 | 0.668 | 1.14E-02 |
| ENSGALG00010025003 |  | 65,978 | 57,118 | 74,838 | 0.763 | 4.15E-02 |
| ENSGALG00010025007 | ZYG11A | 1,668 | 1,499 | 1,836 | 0.817 | 3.43E-03 |
| ENSGALG00010025042 | BCO2 | 9 | 3 | 15 | 0.228 | 1.07E-04 |
| ENSGALG00010025044 | TMEM9 | 117 | 67 | 167 | 0.402 | 6.92E-15 |
| ENSGALG00010025067 | SLC1A2 | 107 | 83 | 130 | 0.638 | 2.18E-02 |
| ENSGALG00010025072 | VAMP2 | 19 | 11 | 29 | 0.366 | 1.03E-03 |
| ENSGALG00010025097 | LMBR1 | 1,419 | 1,334 | 1,503 | 0.888 | 2.80E-02 |
| ENSGALG00010025118 |  | 3 | 5 | 1 | 4.415 | 2.96E-03 |
| ENSGALG00010025136 |  | 15 | 17 | 12 | 1.395 | 4.92E-02 |
| ENSGALG00010025139 | COPB2 | 3,866 | 3,621 | 4,110 | 0.881 | 1.48E-02 |
| ENSGALG00010025149 | IFT81 | 168 | 154 | 182 | 0.845 | 3.20E-02 |
| ENSGALG00010025156 | DLAT | 3,828 | 4,276 | 3,380 | 1.266 | 9.18E-04 |
| ENSGALG00010025168 | POPDC2 | 1,558 | 1,340 | 1,776 | 0.754 | 4.02E-03 |
| ENSGALG00010025188 | PPFIA4 | 4 | 2 | 6 | 0.383 | 3.71E-03 |
| ENSGALG00010025207 | BMPR2 | 2,264 | 2,708 | 1,819 | 1.489 | 1.99E-03 |
| ENSGALG00010025210 | HSPB2 | 2,389 | 1,851 | 2,928 | 0.632 | 8.50E-05 |
| ENSGALG00010025216 | DPP6 | 19 | 25 | 14 | 1.78 | 2.11E-02 |
| ENSGALG00010025238 | IGSF9B | 40 | 28 | 53 | 0.523 | 2.88E-02 |
| ENSGALG00010025257 | LPCAT2 | 525 | 489 | 560 | 0.871 | 6.78E-04 |
| ENSGALG00010025292 | ATP1A1 | 15,584 | 11,787 | 19,380 | 0.608 | 1.99E-05 |
| ENSGALG00010025298 |  | 100 | 68 | 132 | 0.52 | 6.36E-04 |
| ENSGALG00010025303 | TMCC2 | 3,112 | 2,679 | 3,546 | 0.755 | 6.38E-03 |
| ENSGALG00010025323 | KLHDC8A | 70 | 47 | 93 | 0.5 | 4.69E-06 |
| ENSGALG00010025334 | RHOF | 114 | 138 | 91 | 1.529 | 4.20E-02 |
| ENSGALG00010025359 | CDCP2 | 16 | 21 | 11 | 1.887 | 4.36E-02 |
| ENSGALG00010025360 | THYN1 | 3,120 | 2,628 | 3,613 | 0.727 | 2.11E-04 |
| ENSGALG00010025362 | G6PC2 | 40 | 32 | 48 | 0.672 | 1.48E-02 |
| ENSGALG00010025365 | SIKE1 | 3,803 | 4,371 | 3,236 | 1.351 | 3.83E-03 |
| ENSGALG00010025370 | HPD | 97 | 56 | 139 | 0.398 | 1.35E-09 |
| ENSGALG00010025389 | SNED1 | 223 | 249 | 196 | 1.268 | 2.46E-02 |
| ENSGALG00010025404 | CERS6 | 131 | 108 | 154 | 0.7 | 3.10E-02 |
| ENSGALG00010025413 | NECAB3 | 554 | 491 | 617 | 0.794 | 1.08E-02 |
| ENSGALG00010025419 | CCND1 | 796 | 854 | 737 | 1.16 | 1.02E-03 |
| ENSGALG00010025420 | STK39 | 276 | 216 | 336 | 0.642 | 2.61E-03 |
| ENSGALG00010025429 | SCN3B | 13 | 18 | 8 | 2.203 | 1.23E-02 |
| ENSGALG00010025442 | ABCF2 | 19,922 | 22,423 | 17,420 | 1.287 | 5.09E-03 |
| ENSGALG00010025477 |  | 8,496 | 9,157 | 7,834 | 1.169 | 1.55E-03 |
| ENSGALG00010025481 | TMEM39A | 502 | 462 | 542 | 0.852 | 7.24E-03 |
| ENSGALG00010025483 | FGF4 | 138 | 181 | 95 | 1.919 | 4.23E-06 |
| ENSGALG00010025485 | DENND2C | 1,302 | 1,492 | 1,111 | 1.342 | 2.45E-03 |
| ENSGALG00010025488 | SLC4A8 | 36 | 45 | 26 | 1.692 | 6.02E-04 |
| ENSGALG00010025508 |  | 374 | 295 | 452 | 0.653 | 1.12E-05 |
| ENSGALG00010025538 | ANO1 | 2,371 | 2,071 | 2,671 | 0.775 | 1.28E-03 |
| ENSGALG00010025555 |  | 3 | 5 | 2 | 2.531 | 3.04E-02 |
| ENSGALG00010025559 | SLC6A17 | 17 | 7 | 27 | 0.267 | 1.25E-04 |
| ENSGALG00010025569 | PPTC7 | 1,944 | 2,294 | 1,595 | 1.439 | 1.22E-06 |
| ENSGALG00010025579 | CSRNP3 | 6 | 9 | 3 | 2.64 | 1.78E-02 |
| ENSGALG00010025590 | HRAS | 1,890 | 1,483 | 2,298 | 0.645 | 5.00E-08 |
| ENSGALG00010025594 | UPK1B | 15 | 6 | 23 | 0.269 | 2.56E-03 |
| ENSGALG00010025600 |  | 200 | 179 | 221 | 0.81 | 1.48E-02 |
| ENSGALG00010025610 | RNH1 | 4,667 | 3,562 | 5,773 | 0.617 | 2.70E-08 |
| ENSGALG00010025613 | CDKN1A | 3,833 | 2,568 | 5,097 | 0.504 | 1.56E-06 |
| ENSGALG00010025623 |  | 39 | 29 | 49 | 0.589 | 1.43E-02 |
| ENSGALG00010025635 | CHKA | 880 | 669 | 1,091 | 0.613 | 3.95E-03 |
| ENSGALG00010025658 | PDE1B | 44 | 52 | 36 | 1.423 | 4.68E-03 |
| ENSGALG00010025659 | COBLL1 | 505 | 579 | 431 | 1.342 | 3.91E-03 |
| ENSGALG00010025685 | NCKAP1L | 256 | 204 | 308 | 0.659 | 3.98E-02 |
| ENSGALG00010025703 | UBASH3B | 1,151 | 984 | 1,318 | 0.747 | 4.86E-02 |
| ENSGALG00010025705 | ACSL3 | 565 | 604 | 526 | 1.151 | 2.26E-03 |
| ENSGALG00010025713 |  | 11 | 15 | 8 | 1.718 | 2.88E-02 |
| ENSGALG00010025717 |  | 21 | 28 | 14 | 2.001 | 1.21E-02 |
| ENSGALG00010025748 | ETNK2 | 35 | 30 | 40 | 0.725 | 4.79E-02 |
| ENSGALG00010025786 |  | 7 | 8 | 5 | 1.838 | 3.13E-02 |
| ENSGALG00010025791 | FIGN | 160 | 176 | 143 | 1.238 | 4.13E-02 |
| ENSGALG00010025833 |  | 715 | 782 | 649 | 1.206 | 3.83E-02 |
| ENSGALG00010025839 |  | 88 | 101 | 75 | 1.356 | 4.63E-03 |
| ENSGALG00010025847 | DEF6 | 197 | 170 | 224 | 0.758 | 2.39E-02 |
| ENSGALG00010025890 | SCG2 | 150 | 109 | 191 | 0.569 | 1.66E-03 |
| ENSGALG00010025892 | ZC3H11A | 1,472 | 1,541 | 1,404 | 1.097 | 3.43E-02 |
| ENSGALG00010025897 | MAPKAPK5 | 1,772 | 1,984 | 1,561 | 1.272 | 1.85E-03 |
| ENSGALG00010025922 |  | 3 | 1 | 5 | 0.283 | 4.56E-02 |
| ENSGALG00010025931 | TMEM136 | 39 | 50 | 29 | 1.757 | 7.12E-03 |
| ENSGALG00010025937 |  | 3 | 2 | 5 | 0.404 | 4.22E-02 |
| ENSGALG00010025940 | HECTD3 | 1,124 | 1,007 | 1,241 | 0.811 | 2.44E-02 |
| ENSGALG00010025961 |  | 2 | 3 | 0 | 6.573 | 5.00E-02 |
| ENSGALG00010025963 | OAF | 636 | 525 | 748 | 0.702 | 2.22E-02 |
| ENSGALG00010025982 | SERPINE2 | 1,843 | 1,568 | 2,117 | 0.741 | 1.13E-02 |
| ENSGALG00010025984 | NDUFS1 | 9,509 | 10,854 | 8,163 | 1.33 | 2.08E-05 |
| ENSGALG00010025997 | CCDC43 | 732 | 775 | 688 | 1.127 | 2.49E-02 |
| ENSGALG00010026002 | FTH1 | 31,495 | 26,500 | 36,491 | 0.726 | 8.90E-03 |
| ENSGALG00010026005 | DERL3 | 259 | 240 | 277 | 0.863 | 3.46E-02 |
| ENSGALG00010026008 | NRP2 | 2,773 | 2,468 | 3,078 | 0.802 | 3.09E-02 |
| ENSGALG00010026009 |  | 119 | 164 | 74 | 2.192 | 5.12E-10 |
| ENSGALG00010026011 | GJC2 | 396 | 431 | 362 | 1.189 | 2.87E-02 |
| ENSGALG00010026023 | THY1 | 1,085 | 873 | 1,297 | 0.673 | 1.58E-02 |
| ENSGALG00010026036 | TUBA5 | 2,645 | 3,148 | 2,143 | 1.469 | 6.81E-03 |
| ENSGALG00010026046 |  | 834 | 684 | 985 | 0.694 | 5.01E-03 |
| ENSGALG00010026078 | UBE2E3 | 1,294 | 1,405 | 1,184 | 1.188 | 2.96E-02 |
| ENSGALG00010026081 | KPNA1 | 3,088 | 3,352 | 2,824 | 1.187 | 2.37E-02 |
| ENSGALG00010026094 | C6orf106 | 9,244 | 10,180 | 8,308 | 1.226 | 1.83E-02 |
| ENSGALG00010026098 | STAT3 | 5,045 | 4,649 | 5,441 | 0.854 | 2.62E-02 |
| ENSGALG00010026111 |  | 369 | 303 | 435 | 0.694 | 2.67E-02 |
| ENSGALG00010026112 | ZFP91 | 3,524 | 3,837 | 3,210 | 1.196 | 6.04E-03 |
| ENSGALG00010026115 | MMACHC | 170 | 192 | 148 | 1.297 | 1.63E-02 |
| ENSGALG00010026124 | PACSIN1 | 18 | 23 | 13 | 1.753 | 8.99E-03 |
| ENSGALG00010026128 | PRDX1 | 4,659 | 4,205 | 5,112 | 0.823 | 2.85E-02 |
| ENSGALG00010026133 |  | 43 | 31 | 55 | 0.555 | 2.14E-04 |
| ENSGALG00010026154 | ZNF385B | 350 | 263 | 437 | 0.602 | 5.73E-03 |
| ENSGALG00010026159 |  | 80 | 57 | 103 | 0.551 | 6.60E-07 |
| ENSGALG00010026184 | NUDT3 | 2,454 | 2,798 | 2,110 | 1.327 | 7.26E-04 |
| ENSGALG00010026213 | AKR1A1 | 1,228 | 1,160 | 1,296 | 0.894 | 4.61E-02 |
| ENSGALG00010026214 |  | 21 | 27 | 16 | 1.689 | 2.42E-02 |
| ENSGALG00010026221 | CRKL | 3,221 | 3,467 | 2,975 | 1.166 | 3.10E-02 |
| ENSGALG00010026229 | CRHR2 | 1,004 | 1,220 | 788 | 1.549 | 3.81E-03 |
| ENSGALG00010026237 | KLHL22 | 2,167 | 2,517 | 1,817 | 1.386 | 9.23E-04 |
| ENSGALG00010026242 | CCDC141 | 3,248 | 2,709 | 3,788 | 0.715 | 4.66E-03 |
| ENSGALG00010026252 | ENO2 | 35 | 24 | 46 | 0.526 | 2.14E-02 |
| ENSGALG00010026293 |  | 3,238 | 3,856 | 2,621 | 1.472 | 3.37E-05 |
| ENSGALG00010026308 |  | 222 | 275 | 169 | 1.631 | 4.66E-03 |
| ENSGALG00010026329 |  | 60 | 46 | 74 | 0.622 | 1.20E-02 |
| ENSGALG00010026338 |  | 38 | 51 | 25 | 2.108 | 2.18E-06 |
| ENSGALG00010026354 |  | 48 | 25 | 72 | 0.339 | 9.23E-04 |
| ENSGALG00010026355 | PPP1R12B | 13,848 | 12,235 | 15,462 | 0.791 | 2.07E-04 |
| ENSGALG00010026367 | RBP | 6 | 3 | 8 | 0.426 | 1.26E-02 |
| ENSGALG00010026381 |  | 67 | 84 | 50 | 1.656 | 2.34E-03 |
| ENSGALG00010026393 | SLC43A3 | 338 | 254 | 422 | 0.601 | 9.20E-08 |
| ENSGALG00010026403 | GUCD1 | 2,267 | 2,430 | 2,104 | 1.155 | 4.67E-02 |
| ENSGALG00010026448 | ADORA2A | 69 | 80 | 58 | 1.369 | 1.41E-02 |
| ENSGALG00010026472 |  | 10 | 13 | 7 | 1.887 | 2.56E-02 |
| ENSGALG00010026563 |  | 4 | 3 | 5 | 0.484 | 3.41E-02 |
| ENSGALG00010026580 |  | 8 | 5 | 11 | 0.465 | 8.66E-03 |
| ENSGALG00010026588 | CCND3 | 1,208 | 1,326 | 1,091 | 1.216 | 1.00E-02 |
| ENSGALG00010026605 |  | 39 | 45 | 34 | 1.314 | 1.18E-02 |
| ENSGALG00010026608 |  | 23 | 16 | 30 | 0.549 | 1.66E-02 |
| ENSGALG00010026609 |  | 677 | 542 | 813 | 0.667 | 2.54E-04 |
| ENSGALG00010026636 |  | 22 | 27 | 16 | 1.641 | 1.53E-02 |
| ENSGALG00010026643 | YWHAH | 2,804 | 2,532 | 3,076 | 0.823 | 2.31E-02 |
| ENSGALG00010026707 | GORASP1 | 1,936 | 1,801 | 2,072 | 0.87 | 1.01E-02 |
| ENSGALG00010026717 |  | 380 | 466 | 295 | 1.575 | 1.62E-02 |
| ENSGALG00010026725 |  | 344 | 376 | 312 | 1.202 | 1.47E-02 |
| ENSGALG00010026740 | DAB1 | 25 | 30 | 19 | 1.558 | 4.10E-02 |
| ENSGALG00010026753 |  | 65 | 81 | 49 | 1.657 | 2.56E-03 |
| ENSGALG00010026773 |  | 30 | 37 | 23 | 1.629 | 1.80E-02 |
| ENSGALG00010026800 |  | 61 | 82 | 39 | 2.091 | 2.10E-06 |
| ENSGALG00010026805 |  | 224 | 191 | 257 | 0.743 | 4.97E-06 |
| ENSGALG00010026814 |  | 119 | 131 | 107 | 1.214 | 1.23E-02 |
| ENSGALG00010026826 |  | 12 | 15 | 8 | 1.937 | 3.29E-02 |
| ENSGALG00010026827 | FAM117A | 944 | 843 | 1,045 | 0.807 | 1.59E-02 |
| ENSGALG00010026846 | MICAL1 | 356 | 256 | 455 | 0.562 | 6.37E-03 |
| ENSGALG00010026866 |  | 37 | 45 | 29 | 1.539 | 2.45E-02 |
| ENSGALG00010026893 | FKBP5 | 240 | 181 | 298 | 0.606 | 5.65E-04 |
| ENSGALG00010026897 |  | 17 | 21 | 14 | 1.51 | 4.56E-02 |
| ENSGALG00010026909 |  | 209 | 183 | 236 | 0.773 | 3.21E-02 |
| ENSGALG00010026954 | MFSD4A | 59 | 68 | 50 | 1.338 | 2.59E-02 |
| ENSGALG00010026958 | CLTCL1 | 6,333 | 5,675 | 6,991 | 0.812 | 6.72E-05 |
| ENSGALG00010026964 | SRPK1 | 2,489 | 2,590 | 2,389 | 1.084 | 4.87E-02 |
| ENSGALG00010026971 |  | 3 | 4 | 2 | 2.34 | 4.80E-02 |
| ENSGALG00010027002 |  | 22 | 30 | 14 | 2.2 | 8.14E-03 |
| ENSGALG00010027004 |  | 7 | 10 | 3 | 3.46 | 6.67E-03 |
| ENSGALG00010027020 |  | 6 | 9 | 4 | 2.387 | 1.03E-02 |
| ENSGALG00010027025 | ORAI1 | 641 | 685 | 597 | 1.149 | 3.18E-02 |
| ENSGALG00010027027 | ARHGEF3 | 326 | 307 | 345 | 0.886 | 4.90E-02 |
| ENSGALG00010027039 |  | 4 | 6 | 2 | 3.208 | 8.38E-03 |
| ENSGALG00010027040 | CLIP1 | 2,773 | 2,491 | 3,054 | 0.816 | 1.27E-02 |
| ENSGALG00010027041 | MAPK14 | 5,422 | 4,933 | 5,910 | 0.835 | 3.55E-06 |
| ENSGALG00010027042 |  | 18 | 22 | 14 | 1.542 | 1.03E-02 |
| ENSGALG00010027043 | IL17RD | 271 | 229 | 313 | 0.731 | 2.56E-03 |
| ENSGALG00010027045 | MAPK13 | 240 | 180 | 300 | 0.599 | 1.10E-03 |
| ENSGALG00010027050 | GSTT1 | 2,260 | 1,630 | 2,891 | 0.564 | 5.14E-07 |
| ENSGALG00010027063 | RAB7L1 | 920 | 822 | 1,017 | 0.808 | 2.49E-03 |
| ENSGALG00010027074 | BRPF3 | 1,468 | 1,573 | 1,364 | 1.155 | 1.96E-02 |
| ENSGALG00010027077 | ARF4 | 3,462 | 3,256 | 3,669 | 0.887 | 2.67E-02 |
| ENSGALG00010027081 | PDE4B | 706 | 828 | 585 | 1.417 | 1.66E-02 |
| ENSGALG00010027085 | PIM1 | 100 | 79 | 120 | 0.655 | 3.71E-02 |
| ENSGALG00010027086 | CACNA1S | 20,412 | 18,236 | 22,588 | 0.807 | 5.09E-03 |
| ENSGALG00010027087 | RAB7B | 41 | 29 | 52 | 0.553 | 2.92E-02 |
| ENSGALG00010027108 |  | 101 | 86 | 116 | 0.731 | 3.93E-02 |
| ENSGALG00010027117 | RASSF5 | 125 | 95 | 155 | 0.613 | 3.70E-03 |
| ENSGALG00010027128 | EIF2D | 1,155 | 1,251 | 1,059 | 1.183 | 7.01E-03 |
| ENSGALG00010027133 | CELF5 | 6 | 3 | 8 | 0.422 | 3.19E-03 |
| ENSGALG00010027139 | HSD11B1L | 87 | 79 | 95 | 0.831 | 3.65E-02 |
| ENSGALG00010027150 | YOD1 | 1,109 | 1,342 | 875 | 1.534 | 6.17E-07 |
| ENSGALG00010027152 | C4BPS | 340 | 264 | 417 | 0.632 | 1.26E-03 |
| ENSGALG00010027155 |  | 198 | 153 | 242 | 0.629 | 2.45E-02 |
| ENSGALG00010027163 | SAFB | 614 | 654 | 574 | 1.136 | 2.82E-03 |
| ENSGALG00010027176 |  | 1,291 | 1,375 | 1,206 | 1.141 | 1.58E-02 |
| ENSGALG00010027178 | C4BPM | 1,369 | 1,300 | 1,438 | 0.905 | 9.24E-03 |
| ENSGALG00010027183 | CD34 | 589 | 680 | 497 | 1.368 | 9.44E-06 |
| ENSGALG00010027186 | PLXNA2 | 340 | 396 | 283 | 1.403 | 1.32E-03 |
| ENSGALG00010027190 | IPPK | 448 | 484 | 413 | 1.174 | 1.44E-02 |
| ENSGALG00010027195 | CENPP | 25 | 29 | 20 | 1.396 | 4.05E-02 |
| ENSGALG00010027208 | UBXN6 | 484 | 413 | 555 | 0.744 | 1.59E-04 |
| ENSGALG00010027210 |  | 42 | 34 | 51 | 0.659 | 1.39E-02 |
| ENSGALG00010027212 | IRF6 | 79 | 43 | 116 | 0.368 | 1.06E-11 |
| ENSGALG00010027222 | GUCA1B | 26 | 14 | 38 | 0.371 | 5.67E-03 |
| ENSGALG00010027231 | ATP2B2 | 58 | 71 | 45 | 1.559 | 4.01E-04 |
| ENSGALG00010027249 | C2CD4C | 5 | 2 | 8 | 0.22 | 1.54E-03 |
| ENSGALG00010027258 |  | 119 | 58 | 180 | 0.321 | 7.40E-14 |
| ENSGALG00010027274 | COPG1 | 2,358 | 2,239 | 2,478 | 0.903 | 2.82E-04 |
| ENSGALG00010027276 |  | 6,170 | 5,251 | 7,089 | 0.741 | 2.73E-03 |
| ENSGALG00010027277 | KCND3 | 70 | 37 | 103 | 0.358 | 7.52E-04 |
| ENSGALG00010027281 |  | 36 | 31 | 41 | 0.74 | 4.86E-02 |
| ENSGALG00010027286 | RPN1 | 4,080 | 3,685 | 4,474 | 0.824 | 2.21E-07 |
| ENSGALG00010027328 | RBM15 | 659 | 777 | 541 | 1.438 | 4.15E-05 |
| ENSGALG00010027337 | AQP1 | 672 | 517 | 828 | 0.624 | 1.54E-02 |
| ENSGALG00010027358 | RNF126 | 699 | 664 | 734 | 0.906 | 1.48E-02 |
| ENSGALG00010027364 | FSTL3 | 53 | 41 | 64 | 0.65 | 4.29E-02 |
| ENSGALG00010027367 | SEC22C | 409 | 431 | 387 | 1.115 | 3.56E-02 |
| ENSGALG00010027375 | CFTII | 27 | 20 | 34 | 0.581 | 3.40E-02 |
| ENSGALG00010027381 | KLHL40 | 13,934 | 11,803 | 16,065 | 0.735 | 2.43E-02 |
| ENSGALG00010027388 | URM1 | 1,396 | 1,216 | 1,575 | 0.772 | 3.66E-03 |
| ENSGALG00010027408 |  | 7 | 9 | 5 | 1.83 | 3.82E-02 |
| ENSGALG00010027417 | WNT2B | 51 | 34 | 68 | 0.504 | 2.27E-03 |
| ENSGALG00010027438 | ST7L | 583 | 552 | 615 | 0.897 | 8.37E-03 |
| ENSGALG00010027440 | TWF2 | 3,724 | 3,516 | 3,932 | 0.894 | 4.56E-02 |
| ENSGALG00010027443 | MED16 | 562 | 530 | 595 | 0.889 | 2.69E-02 |
| ENSGALG00010027445 | PPM1M | 425 | 359 | 492 | 0.729 | 3.13E-07 |
| ENSGALG00010027468 | BSG | 6,644 | 5,846 | 7,443 | 0.785 | 1.01E-05 |
| ENSGALG00010027490 | ATP2B4 | 7,428 | 5,696 | 9,161 | 0.622 | 3.82E-06 |
| ENSGALG00010027510 | RHOC | 381 | 236 | 526 | 0.448 | 8.44E-11 |
| ENSGALG00010027513 | SEMA3G | 237 | 290 | 185 | 1.575 | 5.30E-04 |
| ENSGALG00010027516 | PPM1J | 450 | 399 | 502 | 0.794 | 4.48E-02 |
| ENSGALG00010027522 | PSMD5 | 1,023 | 949 | 1,097 | 0.866 | 7.36E-03 |
| ENSGALG00010027525 | CUTA | 1,009 | 708 | 1,311 | 0.54 | 8.85E-10 |
| ENSGALG00010027530 | PHF19 | 146 | 117 | 174 | 0.673 | 5.66E-05 |
| ENSGALG00010027539 | DDR1 | 1,250 | 1,152 | 1,347 | 0.855 | 1.99E-02 |
| ENSGALG00010027543 | TUBB4B | 7,227 | 6,270 | 8,183 | 0.766 | 2.01E-02 |
| ENSGALG00010027545 | PPARD | 472 | 437 | 506 | 0.861 | 1.39E-02 |
| ENSGALG00010027548 | PCBP4 | 77 | 65 | 89 | 0.733 | 3.60E-02 |
| ENSGALG00010027557 | C6orf132 | 44 | 31 | 57 | 0.545 | 2.54E-04 |
| ENSGALG00010027559 |  | 156 | 107 | 205 | 0.521 | 1.79E-02 |
| ENSGALG00010027567 | PARP3 | 229 | 166 | 291 | 0.57 | 2.51E-08 |
| ENSGALG00010027571 | RBM10 | 2,956 | 3,090 | 2,822 | 1.094 | 4.71E-02 |
| ENSGALG00010027575 | FCER1G | 51 | 39 | 63 | 0.614 | 3.40E-02 |
| ENSGALG00010027586 | HJV | 1,311 | 1,461 | 1,162 | 1.258 | 1.15E-02 |
| ENSGALG00010027590 | SEMA3F | 172 | 200 | 143 | 1.401 | 1.04E-02 |
| ENSGALG00010027612 | BAK1 | 835 | 670 | 999 | 0.671 | 5.78E-05 |
| ENSGALG00010027617 |  | 1,759 | 1,953 | 1,565 | 1.248 | 1.61E-02 |
| ENSGALG00010027623 | APOBEC2 | 9,577 | 7,778 | 11,376 | 0.684 | 3.69E-04 |
| ENSGALG00010027628 | C9orf116 | 19 | 15 | 22 | 0.651 | 2.13E-02 |
| ENSGALG00010027654 | AIF1L | 2,494 | 2,239 | 2,748 | 0.815 | 2.37E-02 |
| ENSGALG00010027663 | COPA | 3,574 | 3,297 | 3,851 | 0.856 | 4.81E-03 |
| ENSGALG00010027665 | TRAF1 | 170 | 146 | 194 | 0.754 | 6.77E-03 |
| ENSGALG00010027702 | FOXP4 | 127 | 101 | 153 | 0.656 | 2.57E-02 |
| ENSGALG00010027706 | GSN | 25,865 | 20,861 | 30,869 | 0.676 | 1.57E-10 |
| ENSGALG00010027711 | WNK2 | 6,496 | 7,184 | 5,807 | 1.237 | 3.12E-02 |
| ENSGALG00010027712 | STOM | 2,677 | 2,164 | 3,190 | 0.679 | 9.26E-05 |
| ENSGALG00010027730 |  | 103 | 117 | 89 | 1.305 | 2.33E-02 |
| ENSGALG00010027745 | BICD2 | 10,324 | 12,190 | 8,458 | 1.441 | 4.59E-07 |
| ENSGALG00010027752 | SCAI | 324 | 348 | 299 | 1.164 | 3.11E-02 |
| ENSGALG00010027778 |  | 42 | 25 | 60 | 0.412 | 7.44E-08 |
| ENSGALG00010027803 | SAPCD2 | 199 | 222 | 176 | 1.269 | 4.34E-02 |
| ENSGALG00010027821 | NMRK2 | 6,699 | 7,610 | 5,788 | 1.315 | 1.65E-02 |
| ENSGALG00010027869 | NSMF | 303 | 219 | 387 | 0.565 | 1.73E-05 |
| ENSGALG00010027874 | FRS3 | 372 | 405 | 338 | 1.199 | 3.25E-02 |
| ENSGALG00010027877 | OAZ1 | 20,663 | 18,446 | 22,880 | 0.806 | 2.87E-02 |
| ENSGALG00010027888 | PFKFB2 | 360 | 392 | 329 | 1.192 | 4.76E-03 |
| ENSGALG00010027891 | CCDC66 | 292 | 321 | 263 | 1.221 | 1.63E-03 |
| ENSGALG00010027896 | TOR4A | 162 | 119 | 205 | 0.579 | 5.27E-05 |
| ENSGALG00010027907 | MKNK2 | 2,391 | 2,656 | 2,126 | 1.25 | 2.75E-03 |
| ENSGALG00010027908 | PBXIP1 | 1,419 | 1,200 | 1,638 | 0.733 | 3.96E-02 |
| ENSGALG00010027920 | SCAMP4 | 1,378 | 1,302 | 1,453 | 0.896 | 2.06E-02 |
| ENSGALG00010027922 | SHC1 | 761 | 717 | 806 | 0.89 | 4.94E-02 |
| ENSGALG00010027928 | FLAD1 | 285 | 323 | 247 | 1.31 | 1.86E-02 |
| ENSGALG00010027942 | KHDC4 | 718 | 764 | 672 | 1.137 | 5.98E-03 |
| ENSGALG00010027954 | CFAP92 | 26 | 36 | 17 | 2.078 | 2.36E-03 |
| ENSGALG00010027958 |  | 469 | 549 | 390 | 1.41 | 2.41E-03 |
| ENSGALG00010027969 | HSPA5 | 9,849 | 9,059 | 10,638 | 0.852 | 5.03E-03 |
| ENSGALG00010027976 | LRSAM1 | 752 | 660 | 845 | 0.78 | 2.28E-02 |
| ENSGALG00010027982 | ENTPD2 | 979 | 1,117 | 841 | 1.33 | 5.69E-05 |
| ENSGALG00010027985 | PSMB4 | 2,624 | 2,115 | 3,133 | 0.675 | 1.43E-02 |
| ENSGALG00010027988 | HDAC11 | 517 | 561 | 473 | 1.186 | 8.17E-03 |
| ENSGALG00010027991 |  | 269 | 319 | 219 | 1.462 | 6.73E-03 |
| ENSGALG00010027993 | CLIC3 | 57 | 36 | 77 | 0.471 | 1.47E-05 |
| ENSGALG00010027994 | TUFT1 | 37 | 28 | 45 | 0.61 | 1.59E-02 |
| ENSGALG00010028027 |  | 58 | 93 | 22 | 4.306 | 3.45E-02 |
| ENSGALG00010028031 | NISCH | 2,899 | 3,269 | 2,529 | 1.293 | 1.21E-03 |
| ENSGALG00010028034 | MUM1L1 | 571 | 540 | 603 | 0.895 | 4.40E-02 |
| ENSGALG00010028042 | DPP7 | 679 | 784 | 575 | 1.365 | 9.64E-03 |
| ENSGALG00010028044 | GAMT | 4,579 | 3,522 | 5,636 | 0.625 | 3.47E-07 |
| ENSGALG00010028048 | S100A10 | 1,041 | 729 | 1,354 | 0.538 | 5.21E-05 |
| ENSGALG00010028056 | SLC25A25 | 3,586 | 4,240 | 2,932 | 1.447 | 1.25E-03 |
| ENSGALG00010028065 | REEP6 | 42 | 28 | 56 | 0.508 | 1.21E-03 |
| ENSGALG00010028080 | MUSTN1 | 15,251 | 8,891 | 21,611 | 0.411 | 1.39E-03 |
| ENSGALG00010028107 | NOTCH1 | 685 | 746 | 624 | 1.195 | 1.96E-02 |
| ENSGALG00010028114 | PFKFB4 | 663 | 575 | 751 | 0.764 | 5.78E-05 |
| ENSGALG00010028124 | SUGP2 | 1,098 | 1,211 | 986 | 1.228 | 6.60E-03 |
| ENSGALG00010028132 | CACNA2D2 | 212 | 235 | 189 | 1.244 | 1.51E-02 |
| ENSGALG00010028142 | PMPCA | 2,509 | 2,674 | 2,344 | 1.141 | 2.46E-05 |
| ENSGALG00010028146 | MAPKAPK3 | 828 | 644 | 1,013 | 0.636 | 7.50E-05 |
| ENSGALG00010028147 | SLC25A42 | 1,205 | 1,374 | 1,036 | 1.326 | 2.70E-03 |
| ENSGALG00010028149 | SNAPC4 | 262 | 292 | 233 | 1.254 | 1.06E-03 |
| ENSGALG00010028150 | ARMC6 | 1,147 | 1,282 | 1,011 | 1.269 | 3.20E-02 |
| ENSGALG00010028151 | DOCK3 | 15 | 11 | 19 | 0.57 | 2.81E-02 |
| ENSGALG00010028152 |  | 11 | 14 | 7 | 1.896 | 2.16E-02 |
| ENSGALG00010028160 |  | 77 | 99 | 55 | 1.778 | 9.14E-04 |
| ENSGALG00010028170 | COPE | 1,196 | 1,119 | 1,273 | 0.879 | 3.34E-02 |
| ENSGALG00010028171 | RNF123 | 3,043 | 3,316 | 2,771 | 1.197 | 5.16E-03 |
| ENSGALG00010028177 | COMP | 6,273 | 4,904 | 7,642 | 0.642 | 5.65E-04 |
| ENSGALG00010028191 | KLHL26 | 333 | 365 | 301 | 1.215 | 2.40E-03 |
| ENSGALG00010028198 | UCK1 | 840 | 656 | 1,024 | 0.64 | 5.81E-04 |
| ENSGALG00010028247 | MVP | 477 | 416 | 539 | 0.771 | 4.71E-03 |
| ENSGALG00010028254 |  | 3 | 1 | 4 | 0.382 | 4.14E-02 |
| ENSGALG00010028283 |  | 1,495 | 1,361 | 1,629 | 0.836 | 2.40E-02 |
| ENSGALG00010028322 | STRBP | 999 | 1,125 | 872 | 1.29 | 8.74E-04 |
| ENSGALG00010028348 | NES | 2,471 | 1,554 | 3,388 | 0.459 | 1.11E-03 |
| ENSGALG00010028364 | MHM2 | 6,381 | 3,116 | 9,645 | 0.323 | 6.26E-04 |
| ENSGALG00010028375 | LMNB2 | 2,637 | 2,917 | 2,357 | 1.237 | 4.23E-06 |
| ENSGALG00010028378 |  | 7 | 4 | 10 | 0.401 | 1.49E-02 |
| ENSGALG00010028394 | ZBTB26 | 788 | 823 | 752 | 1.096 | 4.39E-02 |
| ENSGALG00010028405 | S100A6 | 1,285 | 516 | 2,054 | 0.251 | 1.41E-11 |
| ENSGALG00010028412 |  | 295 | 132 | 458 | 0.288 | 5.22E-09 |
| ENSGALG00010028422 | RXRA | 1,891 | 2,177 | 1,604 | 1.357 | 9.00E-03 |
| ENSGALG00010028430 | NR2C2AP | 201 | 172 | 230 | 0.751 | 3.48E-03 |
| ENSGALG00010028435 | ATP8B3 | 18 | 22 | 13 | 1.682 | 4.33E-02 |
| ENSGALG00010028443 |  | 62 | 40 | 84 | 0.477 | 3.07E-03 |
| *ENSGALG00010028444* | *MEF2B* | *139* | *84* | *195* | *0.434* | *6.87E-05* |
| ENSGALG00010028450 | PTGDS | 319 | 246 | 393 | 0.627 | 2.13E-02 |
| ENSGALG00010028456 | FBXW5 | 3,093 | 3,335 | 2,852 | 1.17 | 1.67E-02 |
| ENSGALG00010028459 | MCL1 | 1,830 | 1,579 | 2,080 | 0.76 | 3.38E-03 |
| ENSGALG00010028460 | CRLF1 | 67 | 45 | 88 | 0.51 | 1.28E-05 |
| ENSGALG00010028467 |  | 43 | 32 | 54 | 0.595 | 7.12E-04 |
| ENSGALG00010028477 | NRARP | 48 | 55 | 41 | 1.341 | 6.09E-03 |
| ENSGALG00010028503 | NDOR1 | 105 | 95 | 116 | 0.815 | 1.77E-02 |
| ENSGALG00010028517 | RNF208 | 19 | 13 | 25 | 0.518 | 5.03E-03 |
| ENSGALG00010028538 | FEM1A | 19,421 | 21,475 | 17,367 | 1.237 | 1.44E-04 |
| ENSGALG00010028541 |  | 9 | 12 | 6 | 1.909 | 5.06E-03 |
| ENSGALG00010028559 | WDR34 | 73 | 64 | 81 | 0.792 | 3.82E-02 |
| ENSGALG00010028565 | PKN3 | 229 | 251 | 207 | 1.213 | 2.11E-02 |
| ENSGALG00010028573 | KCNN1 | 135 | 115 | 155 | 0.742 | 4.99E-02 |
| ENSGALG00010028583 | USHBP1 | 9 | 12 | 7 | 1.896 | 4.76E-02 |
| ENSGALG00010028585 | MINDY1 | 1,590 | 1,357 | 1,824 | 0.744 | 1.06E-03 |
| ENSGALG00010028626 | CRAT | 9,354 | 10,821 | 7,888 | 1.372 | 3.61E-07 |
| ENSGALG00010028642 | APH1A | 300 | 251 | 349 | 0.721 | 7.94E-06 |
| ENSGALG00010028649 |  | 1,018 | 799 | 1,236 | 0.647 | 9.02E-04 |
| ENSGALG00010028653 |  | 73 | 84 | 62 | 1.346 | 1.69E-02 |
| ENSGALG00010028669 | ZBTB34 | 1,168 | 1,309 | 1,027 | 1.277 | 1.56E-05 |
| ENSGALG00010028671 | CADM3 | 1,590 | 1,254 | 1,926 | 0.651 | 1.21E-03 |
| ENSGALG00010028685 |  | 11 | 15 | 7 | 2.144 | 7.50E-03 |
| ENSGALG00010028692 | RALGPS1 | 493 | 542 | 444 | 1.22 | 4.69E-02 |
| ENSGALG00010028711 |  | 86 | 102 | 70 | 1.465 | 2.79E-03 |
| ENSGALG00010028743 | ARRDC1 | 863 | 802 | 923 | 0.869 | 4.46E-02 |
| ENSGALG00010028745 |  | 420 | 329 | 512 | 0.643 | 2.66E-02 |
| ENSGALG00010028760 | SSR2 | 1,224 | 1,083 | 1,365 | 0.793 | 1.44E-02 |
| ENSGALG00010028761 | CTSS | 1,354 | 1,063 | 1,645 | 0.646 | 3.00E-02 |
| ENSGALG00010028832 |  | 38 | 12 | 64 | 0.187 | 1.35E-08 |
| ENSGALG00010028854 | RPL7A | 26,621 | 24,100 | 29,142 | 0.827 | 1.59E-02 |
| ENSGALG00010028861 | SURF4 | 3,572 | 3,382 | 3,762 | 0.899 | 2.53E-03 |
| ENSGALG00010028872 | SLC2A6 | 60 | 42 | 78 | 0.534 | 1.25E-02 |
| ENSGALG00010028888 | WDR5B | 949 | 996 | 902 | 1.105 | 2.86E-02 |
| ENSGALG00010028900 | TNC | 2,300 | 1,307 | 3,293 | 0.397 | 2.05E-06 |
| ENSGALG00010028903 |  | 17 | 12 | 22 | 0.569 | 2.45E-03 |
| ENSGALG00010028909 |  | 224 | 199 | 248 | 0.805 | 4.63E-02 |
| ENSGALG00010028915 | ADGRD2 | 96 | 55 | 136 | 0.409 | 1.69E-02 |
| ENSGALG00010028925 |  | 41 | 47 | 35 | 1.337 | 1.92E-02 |
| ENSGALG00010028992 |  | 18 | 12 | 24 | 0.533 | 7.97E-03 |
| ENSGALG00010029057 |  | 82 | 95 | 68 | 1.378 | 3.05E-02 |
| ENSGALG00010029115 |  | 558 | 647 | 469 | 1.382 | 1.09E-03 |
| ENSGALG00010029142 |  | 69 | 78 | 60 | 1.308 | 2.80E-02 |
| ENSGALG00010029143 | SH2D3C | 279 | 308 | 251 | 1.228 | 1.94E-02 |
| ENSGALG00010029150 | NARF | 2,235 | 2,538 | 1,932 | 1.314 | 7.15E-05 |
| ENSGALG00010029165 | RAC3 | 1,800 | 1,533 | 2,067 | 0.742 | 2.06E-05 |
| ENSGALG00010029166 | ZZEF1 | 5,873 | 5,196 | 6,550 | 0.793 | 9.81E-03 |
| ENSGALG00010029167 |  | 1,084 | 1,197 | 971 | 1.233 | 2.19E-02 |
| ENSGALG00010029168 | RFNG | 1,561 | 1,676 | 1,447 | 1.159 | 3.75E-02 |
| ENSGALG00010029170 | DCXR | 101 | 86 | 116 | 0.738 | 1.79E-02 |
| ENSGALG00010029180 | CA4 | 173 | 224 | 121 | 1.849 | 1.40E-03 |
| ENSGALG00010029190 | DHRS7C | 4,079 | 3,413 | 4,746 | 0.719 | 7.26E-05 |
| ENSGALG00010029201 | PIGS | 221 | 197 | 244 | 0.806 | 1.43E-02 |
| ENSGALG00010029203 | ALDOC | 1,104 | 781 | 1,428 | 0.547 | 4.82E-04 |
| ENSGALG00010029206 | CACNG4 | 54 | 41 | 67 | 0.613 | 2.93E-02 |
| ENSGALG00010029209 | PRKCA | 2,682 | 3,060 | 2,304 | 1.328 | 5.85E-04 |
| ENSGALG00010029219 | TLCD1 | 349 | 233 | 466 | 0.5 | 3.24E-09 |
| ENSGALG00010029221 | AXIN2 | 29 | 35 | 22 | 1.569 | 5.48E-03 |
| ENSGALG00010029247 | CCDC92B | 209 | 287 | 130 | 2.215 | 3.16E-05 |
| ENSGALG00010029267 |  | 148 | 131 | 165 | 0.793 | 3.65E-02 |
| ENSGALG00010029269 | WSCD1 | 127 | 140 | 115 | 1.218 | 1.09E-02 |
| ENSGALG00010029270 | TIMP2 | 4,356 | 3,576 | 5,135 | 0.696 | 8.11E-05 |
| ENSGALG00010029274 | VMP1 | 954 | 845 | 1,064 | 0.794 | 3.94E-02 |
| ENSGALG00010029276 | TUBD1 | 176 | 144 | 208 | 0.693 | 3.28E-04 |
| ENSGALG00010029282 | LUC7L3 | 1,255 | 1,338 | 1,172 | 1.141 | 3.46E-02 |
| ENSGALG00010029283 | RPS6KB1 | 3,330 | 3,595 | 3,066 | 1.173 | 4.88E-03 |
| ENSGALG00010029285 | CUX1 | 1,320 | 1,154 | 1,487 | 0.776 | 7.66E-05 |
| ENSGALG00010029291 | RNFT1 | 618 | 556 | 680 | 0.818 | 1.16E-06 |
| ENSGALG00010029297 | SH2B2 | 2,098 | 1,806 | 2,390 | 0.756 | 4.79E-02 |
| ENSGALG00010029307 | SGSH | 74 | 63 | 86 | 0.735 | 4.99E-02 |
| ENSGALG00010029308 | GAA | 112 | 83 | 140 | 0.59 | 3.39E-03 |
| ENSGALG00010029315 | TBC1D16 | 1,551 | 1,385 | 1,717 | 0.807 | 1.76E-02 |
| ENSGALG00010029318 | C17orf58 | 368 | 323 | 414 | 0.778 | 3.57E-02 |
| ENSGALG00010029321 | TBX2 | 59 | 69 | 49 | 1.398 | 1.22E-02 |
| ENSGALG00010029332 | YWHAE | 8,284 | 8,657 | 7,911 | 1.094 | 2.13E-02 |
| ENSGALG00010029344 |  | 413 | 353 | 474 | 0.743 | 1.46E-02 |
| ENSGALG00010029348 | HGS | 990 | 914 | 1,067 | 0.855 | 8.61E-03 |
| ENSGALG00010029357 | ARHGDIA | 3,855 | 3,562 | 4,148 | 0.858 | 1.51E-02 |
| ENSGALG00010029366 | MGAT5B | 111 | 73 | 149 | 0.49 | 1.35E-02 |
| ENSGALG00010029374 | RCC1L | 315 | 344 | 287 | 1.201 | 6.02E-05 |
| ENSGALG00010029376 |  | 392 | 300 | 484 | 0.618 | 2.52E-03 |
| ENSGALG00010029384 | WDR81 | 545 | 488 | 603 | 0.809 | 4.81E-02 |
| ENSGALG00010029385 | CLIP2 | 346 | 376 | 315 | 1.192 | 3.42E-02 |
| ENSGALG00010029387 | SERPINF1 | 7,206 | 6,238 | 8,174 | 0.763 | 7.45E-03 |
| ENSGALG00010029388 | ST6GALNAC2 | 41 | 31 | 51 | 0.612 | 1.95E-02 |
| ENSGALG00010029410 | VTN | 23 | 16 | 31 | 0.521 | 2.14E-02 |
| ENSGALG00010029417 |  | 177 | 148 | 206 | 0.717 | 4.52E-02 |
| ENSGALG00010029420 | LAT2 | 40 | 25 | 55 | 0.453 | 3.14E-07 |
| ENSGALG00010029424 | MAP2K6 | 2,217 | 2,555 | 1,879 | 1.36 | 4.92E-02 |
| ENSGALG00010029427 | SPNS3 | 39 | 25 | 54 | 0.459 | 7.39E-04 |
| ENSGALG00010029440 | FOXK2 | 1,309 | 1,382 | 1,236 | 1.118 | 4.72E-02 |
| ENSGALG00010029451 |  | 314 | 386 | 242 | 1.597 | 2.45E-03 |
| ENSGALG00010029468 |  | 65 | 83 | 47 | 1.799 | 4.57E-04 |
| ENSGALG00010029487 | CDC42EP4 | 108 | 78 | 139 | 0.565 | 1.09E-03 |
| ENSGALG00010029491 |  | 526 | 416 | 636 | 0.654 | 3.64E-02 |
| ENSGALG00010029498 |  | 467 | 343 | 590 | 0.581 | 4.56E-03 |
| ENSGALG00010029506 | OXLD1 | 51 | 45 | 57 | 0.781 | 4.80E-02 |
| ENSGALG00010029515 | NPLOC4 | 2,540 | 2,295 | 2,784 | 0.825 | 1.15E-03 |
| ENSGALG00010029522 | AKAP1 | 6,075 | 5,703 | 6,447 | 0.884 | 1.74E-03 |
| ENSGALG00010029523 | FSCN2 | 10 | 5 | 15 | 0.309 | 5.13E-05 |
| ENSGALG00010029528 | TBL2 | 230 | 198 | 262 | 0.756 | 1.81E-02 |
| ENSGALG00010029539 | FKBP6 | 9 | 7 | 12 | 0.569 | 4.56E-02 |
| ENSGALG00010029552 | NOS2 | 200 | 220 | 180 | 1.217 | 1.07E-02 |
| ENSGALG00010029553 | MYH1C | 680,394 | 765,742 | 595,045 | 1.287 | 4.68E-03 |
| ENSGALG00010029562 | CCL4 | 26 | 16 | 35 | 0.462 | 2.98E-02 |
| ENSGALG00010029564 | NLK | 808 | 860 | 755 | 1.141 | 3.32E-02 |
| ENSGALG00010029574 | SGSM2 | 263 | 228 | 298 | 0.766 | 3.39E-02 |
| ENSGALG00010029579 | GALNT17 | 342 | 379 | 305 | 1.241 | 3.49E-02 |
| ENSGALG00010029582 | SCPEP1 | 994 | 844 | 1,143 | 0.738 | 8.67E-03 |
| ENSGALG00010029584 | TRIM25 | 757 | 662 | 852 | 0.776 | 4.31E-02 |
| ENSGALG00010029590 | UTP6 | 1,165 | 1,099 | 1,232 | 0.892 | 2.91E-02 |
| ENSGALG00010029591 | SUZ12 | 422 | 392 | 451 | 0.868 | 1.66E-02 |
| ENSGALG00010029595 | CLUH | 2,079 | 2,387 | 1,770 | 1.349 | 8.06E-05 |
| ENSGALG00010029605 | C17orf75 | 355 | 325 | 386 | 0.839 | 1.32E-02 |
| ENSGALG00010029611 | YWHAG | 8,003 | 7,252 | 8,754 | 0.828 | 3.95E-03 |
| ENSGALG00010029612 | HSPB1 | 4,856 | 3,648 | 6,063 | 0.602 | 4.88E-03 |
| ENSGALG00010029619 |  | 315 | 276 | 354 | 0.78 | 9.02E-06 |
| ENSGALG00010029624 | RASL10B | 289 | 148 | 430 | 0.343 | 2.32E-02 |
| ENSGALG00010029651 | CHAD | 2,310 | 2,092 | 2,527 | 0.828 | 4.04E-02 |
| ENSGALG00010029659 | VKORC1L1 | 1,952 | 2,374 | 1,530 | 1.553 | 1.43E-03 |
| ENSGALG00010029661 | KRABZFP | 121 | 73 | 169 | 0.434 | 1.26E-02 |
| ENSGALG00010029665 | ASL1 | 143 | 116 | 170 | 0.682 | 2.41E-02 |
| ENSGALG00010029668 | CRCP | 498 | 415 | 580 | 0.714 | 2.46E-03 |
| ENSGALG00010029677 | PHF12 | 2,031 | 2,219 | 1,844 | 1.203 | 1.45E-03 |
| ENSGALG00010029693 | HN1 | 2,816 | 2,448 | 3,184 | 0.769 | 2.98E-04 |
| ENSGALG00010029709 | SLC6A4 | 50 | 61 | 39 | 1.543 | 4.14E-02 |
| ENSGALG00010029716 | GOSR1 | 719 | 687 | 752 | 0.914 | 1.74E-02 |
| ENSGALG00010029717 | TRARG1 | 5 | 2 | 7 | 0.276 | 8.46E-04 |
| ENSGALG00010029726 | SEPTIN9 | 1,874 | 1,607 | 2,142 | 0.75 | 1.19E-03 |
| ENSGALG00010029739 | P2RX5 | 2,651 | 2,034 | 3,269 | 0.622 | 5.12E-05 |
| ENSGALG00010029743 | MYOCD | 2,064 | 2,272 | 1,856 | 1.225 | 2.94E-02 |
| ENSGALG00010029749 | ASPA | 148 | 174 | 122 | 1.432 | 3.33E-02 |
| ENSGALG00010029754 | NLE1 | 217 | 250 | 183 | 1.367 | 3.08E-03 |
| ENSGALG00010029757 | ULK2 | 1,195 | 1,020 | 1,370 | 0.745 | 2.75E-03 |
| ENSGALG00010029758 | DNAH9 | 25 | 17 | 34 | 0.489 | 2.42E-02 |
| ENSGALG00010029761 | MRPL38 | 422 | 393 | 452 | 0.869 | 1.23E-02 |
| ENSGALG00010029762 | SLC47A2 | 17 | 10 | 25 | 0.395 | 1.06E-05 |
| ENSGALG00010029767 | GLOD4 | 838 | 669 | 1,007 | 0.664 | 6.52E-08 |
| ENSGALG00010029768 | KCNJ2 | 1,411 | 1,557 | 1,264 | 1.233 | 4.57E-02 |
| ENSGALG00010029771 | SOX9 | 59 | 47 | 72 | 0.646 | 5.93E-05 |
| ENSGALG00010029774 | RFLNB | 588 | 647 | 530 | 1.22 | 1.15E-03 |
| ENSGALG00010029776 | WBP2 | 669 | 562 | 775 | 0.725 | 2.19E-04 |
| ENSGALG00010029783 | RAD51C | 200 | 232 | 167 | 1.387 | 2.01E-02 |
| ENSGALG00010029801 |  | 3 | 2 | 4 | 0.422 | 3.75E-02 |
| ENSGALG00010029810 |  | 16 | 22 | 9 | 2.539 | 1.36E-03 |
| ENSGALG00010029816 |  | 4 | 7 | 1 | 6.622 | 1.48E-05 |
| ENSGALG00010029836 |  | 4 | 6 | 2 | 3.383 | 5.07E-03 |
| ENSGALG00010029864 | SLC16A3 | 1,079 | 1,348 | 809 | 1.665 | 3.18E-04 |
| ENSGALG00010029936 | SERPINF2 | 361 | 208 | 513 | 0.405 | 2.36E-03 |
| ENSGALG00010029941 | COX10 | 948 | 1,039 | 856 | 1.214 | 3.65E-02 |
| ENSGALG00010029948 | MYADML2 | 1,586 | 1,389 | 1,784 | 0.779 | 1.61E-02 |
| ENSGALG00010029958 |  | 26 | 12 | 39 | 0.3 | 2.84E-02 |
| ENSGALG00010030006 |  | 3 | 5 | 2 | 2.66 | 8.36E-03 |
| ENSGALG00010030014 | SLC47A1 | 39 | 32 | 45 | 0.694 | 2.03E-02 |
| ENSGALG00010030076 |  | 11 | 14 | 8 | 1.749 | 4.22E-02 |
| ENSGALG00010030109 | FBF1 | 1,123 | 1,232 | 1,014 | 1.217 | 3.87E-02 |

**Additional Table 2**. The gene ontology (GO) terms of genes predominantly overexpressed in the pHu- line (N = 53), the pHu+ line (N = 130), or both lines (N = 1) of chickens.

| Cluster number | Information content (IC) | GO ID | GO Term | P-value | Significant genes * |
| --- | --- | --- | --- | --- | --- |
| **Enriched GO terms related to pHu- line up-regulated genes** | | | | | |
| 3 | 7.06 | GO:0010906 | Regulation of glucose metabolic process | 1.06E-03 | PDK3; HMGB1; MLYCD; SIK1; DYRK2; PGP; USP7; PPARA; IRS1; FOXK2 |
| 3 | 8.22 | GO:0045923 | Positive regulation of fatty acid metabolic process | 5.62E-03 | ELOVL5; MLYCD; NR1H3; PPARA; IRS1 |
| 4 | 8.54 | GO:0006086 | Acetyl-coa biosynthetic process from pyruvate | 2.03E-03 | PDK3; DLD; PDHB; DLAT |
| 4 | 8.54 | GO:0006182 | Cgmp biosynthetic process | 2.03E-03 | GUCY1A2; GUCY1A1; GUCY1B1; |
| 4 | 5.97 | GO:0009152 | Purine ribonucleotide biosynthetic process | 7.53E-03 | ATP6; ELOVL5; PDK3; GUCY1A2; MLYCD; GUCY1A1; GUCY1B1; MMUT; DLD; PPARGC1A; GART; ELOVL7; ADSS1; NME3; PANK4; PDHB; ADK; PPARA; DLAT; |
| 4 | 8.91 | GO:0035336 | Long-chain fatty-acyl-coa metabolic process | 2.03E-03 | ELOVL5; DGAT2; ELOVL7; FAR1 |
| 4 | 8.17 | GO:1900542 | Regulation of purine nucleotide metabolic process | 7.02E-03 | PDK3; ME1; PPARGC1A; DNM1L; PPARA |
| 5 | 7.75 | GO:0006120 | Mitochondrial electron transport, NADH to ubiquinone | 9.68E-05 | ND1; ND2; ND3; ND4; ND5; DNAJC15; DLD; NDUFS1 |
| 5 | 3.41 | GO:0006796 | Phosphate-containing compound metabolic process | 6.60E-03 | ATP6; FGFR2; PTP4A1; PRPS2; KLHL31; ELOVL5; PDK3; CAV1; ME1; DGAT2; PRAG1; RRM1; RNGTT; ENPP3; GUCY1A2; FLT1; CRYL1; CDK8; TEK; HMGB1; CKMT2; CDH5; MLYCD; GUCY1A1; GUCY1B1; MMUT; PPARGC1B; ADGRF5; PPIP5K2; NHLRC1; NUDT12; HNMT; PTK2; DLD; PPARGC1A; HUNK; MAK; CDC14B; FECH; DNM1L; PRPF4B; KITLG; SIK1; DYRK2; GART; PDP1; PDGFB; CLK4; STK38L; PTPRR; BPGM; PLPP6; KDR; OPA1; HMGCS1; GHR; ELOVL7; OCRL; DGKK; PAK3; PWP1; NADK2; MTMR7; SNRK; ADSS1; GPCPD1; PRKCH; PGP; PLCB1; NME3; SOCS5; RALB; PANK4; PRKCZ; MOCS1; BRAT1; MAPK8; NEK2; PDHB; PKLR; FAR1; DHRS7B; STRADA; DNAJC10; ADK; NR1H3; TIE1; PPARA; TLK2; STOX1; PTK6; PAK6; TPI1; EPHB1; KCTD20; TOLLIP; STRADB; DLAT; BMPR2; CCND1; FGF4; ACSL3; MAPKAPK5; NDUFS1; NUDT3; CRKL; CCND3; SRPK1; WNK2; PFKFB2; MKNK2; FLAD1; SLC25A25; PKN3; PRKCA; AXIN2; RPS6KB1; YWHAE; MAP2K6; NLK |
| 5 | 5.65 | GO:0015980 | Energy derivation by oxidation of organic compounds | 3.12E-03 | ND1; ND2; ATP6; ND3; ND4L; ND4; ND5; CYTB; DNAJC15; KL; HMGB1; ADGRF5; NHLRC1; DLD; PPARGC1A; DYRK2; COQ10B; UGP2; PPIF; SLC25A12; IRS1; ETFA; IREB2; PHKB; IDH3A; NDUFS1; SLC25A25; COX10 |
| 6 | 7.98 | GO:0000097 | Sulfur amino acid biosynthetic process | 5.97E-03 | MTRR; MTAP; CBS |
| 6 | 8.48 | GO:0006744 | Ubiquinone biosynthetic process | 7.97E-03 | PDSS2; COQ10B; COQ5; PPTC7 |
| 6 | 4.8 | GO:0032787 | Monocarboxylic acid metabolic process | 7.38E-03 | ELOVL5; PDK3; CAV1; PEX2; PHYH; HPGDS; CRYL1; MLYCD; ALDH5A1; ETFDH; DLD; GPR50; MBLAC2; AGXT2; BPGM; PGK2; ELOVL7; ABAT; ERLIN2; GATM; HACD2; PDHB; PKLR; DAGLA; NR1H3; PPARA; TPI1; IRS1; ETFA; LDHA; INSIG1; DLAT; ACSL3; PFKFB2; CRAT; FOXK2; ASPA |
| 6 | 6.83 | GO:0043648 | Dicarboxylic acid metabolic process | 5.78E-03 | MTRR; ME1; PHYH; ALDH5A1; AADAT; GLUD2; GLS2; D2HGDH; ASPA |
| 7 | 5.77 | GO:0000209 | Protein polyubiquitination | 9.63E-03 | UBE3D; RNF144A; RNF6; NHLRC1; KLHL20; UBR5; RNF152; JADE2; TRIM2; UBE3A; WDR24; ARIH2; CDC27; UBE3C; UBE2E3; ZFP91; RNF123 |
| 7 | 4.79 | GO:0016567 | Protein ubiquitination | 9.21E-04 | MARCHF11; CAV1; HERC2; KLHL15; UBE3D; PEX2; RNF144A; RNF6; NHLRC1; RNF139; KLHL20; ENC1; HECW2; UBR5; RNF152; MTA1; JADE2; WWP1; AMER1; TRIM2; KLHL9; UBE3A; RBBP6; WDR24; SOCS5; UBR2; CAND2; KBTBD8; MINAR1; PDZRN3; ARIH2; FBXL15; RNF141; MSL2; RFWD3; ZNRF3; CDC27; UBE3C; UBE2E3; ZFP91; KLHL22; RNF123; FBXW5 |
| 9 | 5.38 | GO:0043161 | Proteasome-mediated ubiquitin-dependent protein catabolic process | 2.12E-03 | FEM1C; HERC2; KLHL15; RNF144A; BBS7; KLHDC10; NHLRC1; KLHL20; MTA1; WWP1; TRIM2; UBE3A; ERLIN2; RNF4; USP7; SOCS5; UBR2; ARIH2; FBXL15; DNAJC10; TLK2; SIAH1; PCBP2; KLHL22; YOD1; FBXW5; FEM1A; AXIN2 |
| 10 | 6.83 | GO:0009063 | Amino acid catabolic process | 6.40E-05 | MTRR; AASS; HIBADH; BCKDHB; ALDH5A1; DLD; GLUD2; CBS; AGXT2; GLS2; ABAT; ETFA; NOS2 |
| 10 | 7.05 | GO:1901606 | Alpha-amino acid catabolic process | 9.30E-03 | MTRR; AASS; HIBADH; GLUD2; CBS; AGXT2; GLS2; NOS2 |
| 11 | 8.48 | GO:0046339 | Diacylglycerol metabolic process | 7.97E-03 | DGAT2; DGKK; PLCE1; DAGLA |
| 13 | 7.75 | GO:1904262 | Negative regulation of TORC1 signaling | 9.32E-03 | DEPTOR; RNF152; SESN1; UBE3A; USP7; NLK |
| 14 | 8.54 | GO:0032228 | Regulation of synaptic transmission, gabaergic | 7.97E-03 | PLCL2; CA2; USP46; ADORA2A |
| 16 | 3.48 | GO:0033554 | Cellular response to stress | 6.04E-04 | TBX3; RNFT2; KLHL31; PDK3; CAV1; SMC6; HERC2; DDX1; KLHL15; RRM1; PEX2; DNAJC15; DNAJB1; MYO6; VASH1; FBXO5; MCM10; EPC1; EGLN3; SUV39H2; DCLRE1C; MLH3; BRCA2; HMGB1; PPARGC1B; NHLRC1; RNF139; ZNF365; PPARGC1A; ESCO2; AQP3; FECH; USP15; CBS; DYRK2; UBR5; RNF152; RAD54B; OXR1; MTA1; RFC1; OPA1; SFPQ; SESN1; FIRRM; FOXO3; RMI1; TMEM33; XRCC3; SLX4; MECOM; LETM1; ERLIN2; RBBP6; MSH6; ACTL6A; WDR24; EPAS1; USP7; PLCB1; EME2; NME3; SOCS5; MGME1; GCH1; RALB; BRAT1; MAPK8; PPIF; SUSD6; ERCC6; DNAJC10; USP33; GPR155; NR1H3; PPARA; TLK2; STOX1; CHRNA4; RFWD3; BOK; USP1; GNB1L; EPHB1; INSIG1; STRADB; BMPR2; CCND1; MAPKAPK5; YOD1; NOTCH1; AXIN2; TBX2; YWHAE; MAP2K6; NLK; VKORC1L1; RAD51C |
| 17 | 6.93 | GO:0007200 | Phospholipase C-activating G protein-coupled receptor signaling pathway | 9.12E-03 | P2RY2; GPR83; CHRM3; GNAQ; AGTR1; LPAR4; PLCB1; PLCE1; ORAI1 |
| 17 | 8.6 | GO:0007263 | Nitric oxide mediated signal transduction | 4.29E-04 | GUCY1A2; GUCY1A1; GUCY1B1; CBS; NOS2 |
| 17 | 6.25 | GO:0030509 | BMP signaling pathway | 4.74E-03 | CDH5; GREM2; USP15; RGMB; KDR; SMAD5; FBN1; RGMA; FBXL15; ZNF423; NFIA; BMPR2; HJV; NOTCH1 |
| 17 | 8.67 | GO:0038166 | Angiotensin-activated signaling pathway | 5.97E-03 | CAV1; CA2; AGTR1; PRKCA |
| 18 | 8.54 | GO:0045662 | Negative regulation of myoblast differentiation | 1.93E-03 | TBX3; DLL1; SOX8; NMRK2; NOTCH1 |
| 18 | 7.91 | GO:1901532 | Regulation of hematopoietic progenitor cell differentiation | 8.45E-04 | HMGB1; HSPA9; KITLG; KDR; YTHDF2; SOS2; NOTCH1 |
| 18 | 8.36 | GO:2000737 | Negative regulation of stem cell differentiation | 3.43E-03 | TBX3; HSPA9; YTHDF2; JAG1; NOTCH1 |
| 19 | 6.91 | GO:0045446 | Endothelial cell differentiation | 5.71E-03 | CDH5; DLL1; KDR; JAG1; PLCB1; SOX18; TIE1; HOXB5; BMPR2; NOTCH1 |
| 19 | 5.47 | GO:0061448 | Connective tissue development | 2.06E-03 | HOXA11; DGAT2; RXFP1; PPARGC1A; CBS; PDGFB; CHADL; SOX5; SMAD5; SOX8; MKKS; DHRS7B; SOX6; SATB2; SULF2; NFIA; BMPR2; FGF4; CD34; SLC25A25; NOTCH1; AXIN2; RFLNB |
| 20 | 8.54 | GO:0001946 | Lymphangiogenesis | 9.80E-04 | VASH1; PPP3CB; SOX18; TIE1; BMPR2 |
| 20 | 6.51 | GO:0002040 | Sprouting angiogenesis | 3.60E-03 | TEK; HMGB1; DLL1; SEMA6A; MMRN2; KDR; ESM1; AGTR1; VSTM4; PKLR; DLL4; NOTCH1; NRARP |
| 21 | 9.01 | GO:0003174 | Mitral valve development | 2.03E-03 | GJA5; BMPR2; NOTCH1; AXIN2 |
| 21 | 8.13 | GO:0003176 | Aortic valve development | 8.64E-03 | DLL4; TIE1; BMPR2; NOTCH1; AXIN2 |
| 21 | 8.67 | GO:0003177 | Pulmonary valve development | 7.97E-03 | GJA5; JAG1; BMPR2; NOTCH1 |
| 21 | 7.09 | GO:0003179 | Heart valve morphogenesis | 6.76E-03 | GJA5; JAG1; DLL4; TIE1; BMPR2; NOTCH1; AXIN2 |
| 21 | 6.38 | GO:0035051 | Cardiocyte differentiation | 5.64E-03 | TBX3; TBX18; DLL1; SIK1; FHL2; JAG1; MEF2A; SOX18; SOX6; NOTCH1; TBX2; MYOCD |
| 23 | 8.82 | GO:0021535 | Cell migration in hindbrain | 3.03E-03 | RBFOX2; RERE; DAB1; PLXNA2 |
| 30 | 8.48 | GO:0007076 | Mitotic chromosome condensation | 5.97E-03 | SMC4; AKAP8L; NCAPD2; NUSAP1 |
| 30 | 8.36 | GO:0010971 | Positive regulation of G2/M transition of mitotic cell cycle | 4.43E-03 | RRM1; FBXO5; STOX1; CCND1; RAD51C |
| 30 | 7.42 | GO:0140962 | Multicellular organismal-level chemical homeostasis | 2.66E-03 | ADGRF5; AQP3; FECH; KDR; SCNN1G; EPAS1; IREB2; HJV |
| 32 | 7.7 | GO:0000002 | Mitochondrial genome maintenance | 3.05E-03 | RRM1; PPARGC1A; OPA1; MGME1; MEF2A; STOX1 |
| 34 | 6.5 | GO:0001935 | Endothelial cell proliferation | 5.24E-03 | CAV2; CAV1; VASH1; FLT1; TEK; HMGB1; MMRN2; PDGFB; KDR; AGTR1; VSTM4; DLL4; TIE1; BMPR2; CD34; NRARP; PRKCA |
| 34 | 7.87 | GO:0001937 | Negative regulation of endothelial cell proliferation | 6.66E-03 | CAV2; CAV1; VASH1; FLT1; MMRN2; DLL4 |
| 34 | 6.51 | GO:0072089 | Stem cell proliferation | 8.84E-03 | TBX3; KITLG; KDR; YTHDF2; ARIH2; SOX18; ZNRF3; FGF4; CD34; NOTCH1; AXIN2 |
| 35 | 8.6 | GO:0010644 | Cell communication by electrical coupling | 2.03E-03 | CAV1; GJA5; GJC3; GJC2 |
| 37 | 5.61 | GO:0031667 | Response to nutrient levels | 6.75E-03 | BCKDHB; PEX2; DNAJC15; PPARGC1A; CBS; RNF152; SESN1; FOXO3; RMI1; CREBBP; MKKS; WDR24; RALB; MAPK8; USP33; GPR155; PPARA; BMPR2; CRHR2; SLC25A25; RXRA; RPS6KB1; FOXK2 |
| 39 | 7.48 | GO:0001895 | Retina homeostasis | 9.00E-03 | NXNL2; MAK; USH2A; MKKS; VSTM4; CROCC; ERCC6 |
| 39 | 8.01 | GO:0120163 | Negative regulation of cold-induced thermogenesis | 4.60E-03 | PLCL2; LAMA4; NR1H3; ZNF423; NOTCH1; MAP2K6 |
| 40 | 6.78 | GO:0061337 | Cardiac conduction | 5.58E-03 | CAV1; TBX18; GJA5; EHD3; MEF2A; GJC3; SCN3B; KCNJ2 |
| 40 | 8.26 | GO:0097150 | Neuronal stem cell population maintenance | 4.43E-03 | DLL1; HOOK3; FOXO3; JAG1; NOTCH1 |
| **Enriched GO terms related to pHu+ line up-regulated genes** | | | | | |
| 1 | 5.05 | GO:0001819 | Positive regulation of cytokine production | 3.18E-03 | COBL; WWTR1; PLXNB2; PAK1; SDCBP; CALR; SOX11; FAM150B; FGF2; JCAD; TGFB3; PLXNA4; CAMK1D; PRKCQ; ITGB1; EAPP; PGF; ID4; ROBO1; TERT; IST1; SEMA4D; SYK; IL11RA; SLC35F6; CD38; FZD3; TNN; TAPT1; IL7R; TNFSF13B; NAP1L1; FGF1; NRG1; KDM1A; TP63; MMP9; HMOX1; HTR2B; LGMN; PDCL3; SAXO1; RPS6; RTN4; CSF1R; DDRGK1; FERMT2; TRAF5; VASH2; ARPC2; EGR3; ANKRD1; CYR61; F3; THBS1; BCAR3; PTPRN; OCSTAMP; GREM1; SRC; CD276; FGF6; PID1; CCND2; CD81; TF; HRAS; NCKAP1L; SCG2; STAT3; MAPK14; PIM1; AQP1; TWF2; DDR1; PPARD; CRLF1; TNC; CDC42EP4; SUZ12; SEPTIN9; SOX9; SERPINF2 |
| 1 | 8.05 | GO:0032655 | Regulation of interleukin-12 production | 5.20E-03 | MET; CALR; FGF2; JCAD; MAPRE2; NUS1; FGF1; MMP9; HMOX1; LGMN; RTN4; ADAM9; RIN2; THBS1; MAP2K3; SRC; HSPB1; SOX9 |
| 1 | 8.26 | GO:0032731 | Positive regulation of interleukin-1 beta production | 1.25E-03 | CD109; FGF2; WFDC1; SMAD3; THBS1; ANXA2; UBASH3B; SERPINE2; SERPINF2 |
| 2 | 9.01 | GO:0010954 | Positive regulation of protein processing | 7.85E-05 | CTSZ; THBS1; SERPINE2; SERPINF2 |
| 2 | 8.91 | GO:0010955 | Negative regulation of protein processing | 8.69E-03 | FGF2; CTSZ; ENO1; THBS1; ANXA2; SERPINE2; SERPINF2 |
| 2 | 5.2 | GO:0031401 | Positive regulation of protein modification process | 6.81E-03 | WWTR1; CD109; MET; SMPD1; KCTD21; CALR; CCDC3; PRKCQ; BANK1; MYOZ2; ITGB1; SHISA2; CCDC125; ROBO1; LTBP1; TERT; DUSP1; TNFAIP6; CLU; SLC35F6; ACAA2; SLIT3; TNN; DUSP22; CREB3; RGS2; RCAN1; STRAP; FKTN; STAT1; KDM1A; RPS6KA1; APCDD1L; TP63; PMEPA1; MMP9; HMOX1; HTR2B; LGMN; UBQLN1; TNIP1; HOMER2; EGR1; SQSTM1; CTNNBIP1; ENO1; APOD; PARP1; CILP; DUSP5; SFRP5; DDRGK1; EIF4E2; PRNP; FERMT1; WFIKKN1; TMBIM1; CAV3; BFAR; CIT; DHRS3; THBS1; IVNS1ABP; MAP2K3; GREM1; SRC; CNOT9; ITGA6; MAPK8IP1; USP47; PIK3IP1; SPRED1; DHX58; PID1; TSPAN15; EYA2; DUSP3; AMFR; TRABD2B; TF; APOA1; ADIPOR1; HYOU1; CASP8; UBASH3B; SCG2; SERPINE2; THY1; MAPK14; IL17RD; RNF126; ATP2B4; BAK1; HSPA5; MCL1; ARRDC1; HGS; NPLOC4; YWHAG; HSPB1; SOX9 |
| 2 | 8.42 | GO:0031639 | Plasminogen activation | 1.54E-04 | SMPD1; NEU3; TM9SF2; GBA2; ST3GAL1; B3GALT2; B4GALT5; ASAH1; GM2A; CYR61; ELOVL1; NEU2; CERS6; CFTII; |
| 3 | 8.26 | GO:0001919 | Regulation of receptor recycling | 2.67E-03 | SMPD1; MAPK11; IRAK4; MAP3K7; TNFRSF11A; EGR1; IRAK2; ANKRD1; SRC; MAPK13; CCL4; SOX9 |
| 3 | 8.91 | GO:0002091 | Negative regulation of receptor internalization | 1.67E-03 | BAG2; FGF2; ENO1; SRC; ANXA2; GSN |
| 3 | 8.67 | GO:0032967 | Positive regulation of collagen biosynthetic process | 5.06E-04 | SDCBP; LRRTM2; LRPAP1; ANKRD13D; ANXA2 |
| 3 | 5.48 | GO:0042327 | Positive regulation of phosphorylation | 2.68E-03 | SARAF; MCUB; FGF2; METTL21C; ATP1B1; PANX1; CYBA; CACNA1E; CACNA2D1; F2RL1; TRPC5; CACNA2D4; TMEM38B; ANXA6; TMCO1; PLN; HTR2B; SRL; CALM1; ERO1A; PRNP; CAV3; SLC30A1; GSTO2; MCU; MICU1; PACSIN3; ANXA2; UBASH3B; THY1; CACNA1S; ATP2B4; BAK1; CACNG4 |
| 3 | 8.54 | GO:0045429 | Positive regulation of nitric oxide biosynthetic process | 8.04E-03 | HPX; CSF2RA; IRAK4; MAP3K7; CCDC3; CCR6; ROBO1; SYK; IL11RA; SLIT3; TNFRSF11A; IL7R; EDAR; STAT1; STAT4; EGR1; CSF1R; TXK; CCR8; EIF4E2; TRAF5; WBP1L; IRAK2; PARP9; SRC; CNOT9; APOA1; ADIPOR1; STK39; STAT3; SH2B2; CCL4 |
| 3 | 7.91 | GO:2000379 | Positive regulation of reactive oxygen species metabolic process | 1.92E-03 | HPX; VIM; IRF8; STAT1; RAB20; TXK; CALM1; VAMP3; PARP9; ZYX; PIM1; GSN; CDC42EP4; CCL4 |
| 6 | 8.31 | GO:0006691 | Leukotriene metabolic process | 1.67E-03 | RGCC; VIM; TGFB3; F2RL1; SOX9; SERPINF2 |
| 8 | 6.83 | GO:0002181 | Cytoplasmic translation | 9.71E-03 | PDIA3; SCEL; HPX; IRAK4; NEU3; PAK1; DFNA5; ATP6V1C2; RPS3; SDCBP; MAP3K7; SPP1; ITGA8; CALR; RPS7; SOX11; FAM150B; LY96; ALPK1; TRIM55; CRH; FGF2; JCAD; GPR158; MAPRE2; BANK1; ITGB1; ROBO1; CTSC; ARC; DCDC2; TERT; LRRTM2; SEMA4D; GADD45G; MAP4K1; SYK; CYBA; WISP1; C7orf73; CTSV; CD180; F2RL1; DNAJC27; GCNT2; LY86; APP; DUSP22; TNFRSF11A; SYT1; RAP1B; IL7R; NMU; EDAR; PRR5; FGF1; NRG1; GKAP1; TP63; MMP9; LARGE1; HTR2B; LGMN; BVES; NUP62; CYP46A1; SH3RF2; RTN4; SLC9A1; CSF1R; TXK; SQSTM1; PARP1; TNIP2; DYNC1LI1; CALM1; GPR68; GPR65; LIPA; FERMT2; FERMT1; VAMP3; TRAF5; REL; LMCD1; CORO7; MAD1L1; ADAM8; ARL6IP5; ANKRD1; PARP9; CYR61; BCL10; F3; THBS1; MMD2; BCAR3; MCU; MAP2K3; GREM1; SRC; CNOT9; MAPK8IP1; USP47; DOK4; AFAP1L2; SPRED1; CD63; DHX58; GADD45A; WLS; PLEKHF1; LIMS2; SERINC3; CD81; CTSD; ANXA2; TF; APOA1; CASP10; CASP8; CAT; TMEM9; STK39; ANO1; HRAS; NCKAP1L; SERPINE2; STAT3; RAB7L1; PIM1; WNT2B; DDR1; PPARD; BAK1; SHC1; MCL1; CCL4; TRIM25; RASL10B; SOX9; WBP2; SERPINF2 |
| 8 | 7.13 | GO:0006487 | Protein N-linked glycosylation | 5.63E-03 | CRPPA; FKTN; LARGE1; LARGE2; POMGNT1 |
| 8 | 7.5 | GO:0006506 | GPI anchor biosynthetic process | 8.20E-03 | SYK; MGST3; LTA4H; PLA2G4A; ALOX5 |
| 8 | 8.26 | GO:0035269 | Protein O-linked mannosylation | 8.04E-03 | PIGN; PIGH; PIGT; PIGK; PGAP3; PIGX; PIGS |
| 9 | 8.6 | GO:0007039 | Protein catabolic process in the vacuole | 1.26E-03 | PTX3; CLU; SMAD3; DDAH1; ASL1 |
| 9 | 6.46 | GO:0036503 | ERAD pathway | 7.63E-03 | ATP1B1; CTNNA3; RGS2; TMEM38B; PLN; SLC9A1; CALM1; CAV3; JUP |
| 10 | 8.54 | GO:0046479 | Glycosphingolipid catabolic process | 8.69E-03 | PGM3; ST3GAL1; NUS1; TUSC3; MGAT4B; RPN2; DERL3; GORASP1; RPN1; MGAT5B |
| 11 | 6.53 | GO:0006672 | Ceramide metabolic process | 2.38E-03 | SSR3; SNX10; IMMP2L; RAB23; RAB31; USO1; CALR; GDAP1; SRP72; ARL11; SCARB2; STX7; IPO5; SEC23A; BCAP29; SYK; DERL1; STX10; HSP90B1; CLU; MICALL1; DNAJC27; MLPH; LAMP2; ARL4C; AP3B1; RANGAP1; TM9SF4; HSPA4; LARGE1; SEC62; SQSTM1; ERLEC1; GGA2; APOD; PRNP; FERMT1; AP2M1; JUP; IPO13; UNC93B1; SNX11; M6PR; STX2; SRP14; WLS; COPB1; KPNB1; CD81; COPB2; HRAS; DERL3; STAT3; YWHAH; CLTCL1; MAPK14; RAB7L1; ARF4; COPG1; COPA; STOM; OAZ1; HSPA5; HGS; NPLOC4; C17orf75; YWHAG; HSPB1 |
| 11 | 8.6 | GO:0006706 | Steroid catabolic process | 2.95E-04 | NEU3; GBA2; GM2A; NEU2 |
| 11 | 6.78 | GO:0008203 | Cholesterol metabolic process | 1.81E-04 | SOX11; TGFB3; BANK1; SAMSN1; DUSP1; TNFRSF11B; TNFAIP6; DUSP22; IL7R; PDCD1; UBQLN1; FGL1A; C1QC; SQSTM1; APOD; PARP1; GPR68; EIF4E2; PRNP; MAD1L1; THBS1; CD276; SDC4; DHX58; DUSP3; ASCL2; APOA1; NCKAP1L; UBASH3B; THY1; PIM1; RASSF5; PARP3; NPLOC4; SOX9 |
| 11 | 7.28 | GO:0046470 | Phosphatidylcholine metabolic process | 4.50E-03 | SMPD1; CYP39A1; GBA2; APP; FGF1; CYP46A1; LIPA; SCARB1; CYP11A1; CYP27A1; NFE2L1; CETP; LCAT; LPCAT3; APOA1; CAT |
| 12 | 5.25 | GO:0002253 | Activation of immune response | 6.80E-03 | MAP3K7; LY96; ALPK1; IPO5; SYK; CYBA; C7orf73; CD38; DUSP22; AP3B1; C3AR1L; ITK; TLR1A; UBQLN1; RTN4; SQSTM1; PARP1; TNIP2; LIPA; PRNP; IRAK2; UNC93B1; SRC; CMKLR1; DHX58; CD79B; DUSP3; CD81; TF; HRAS; NCKAP1L; THY1; RAB7L1; FCER1G; MAPKAPK3; SH2B2; LAT2; NPLOC4; TRIM25 |
| 12 | 5.32 | GO:0002764 | Immune response-regulating signaling pathway | 5.57E-03 | CALR; CAMK1D; PLA2G7; CREB3; LGMN; CSF1R; MCU; CMKLR1; STK39; NCKAP1L |
| 12 | 8.17 | GO:0050856 | Regulation of T cell receptor signaling pathway | 2.34E-03 | SPP1; CYP39A1; CYP46A1; SCARB1; CYP27A1; YWHAH |
| 13 | 4.17 | GO:0009968 | Negative regulation of signal transduction | 6.30E-03 | ; ITGA8; ITGB1; TNN; DUSP22; FGL1A; APOD; ADAM9; FERMT2; RIN2; FERMT1; LDB1; THBS1; JUP; GREM1; SRC; VCL; CD63; ZYX; ANXA2; THY1; VTN |
| 13 | 8.74 | GO:0070885 | Negative regulation of calcineurin-NFAT signaling cascade | 2.66E-03 | ATP1B1; PRNP; KCNAB2; KCNIP2; CD63; KLHL24; STK39 |
| 13 | 6.8 | GO:2001243 | Negative regulation of intrinsic apoptotic signaling pathway | 4.52E-03 | CAMK1D; TNFAIP6; CD99L2; ADAM8; MCU; NCKAP1L; RAC3 |
| 14 | 8.17 | GO:0035330 | Regulation of hippo signaling | 8.58E-03 | PDIA3; KLF5; HPX; MAPK11; CSF2RA; IRAK4; SNX10; DFNA5; MAP3K7; VIM; CCDC3; CCR6; IRF8; ROBO1; DUSP1; SYK; IL11RA; NPR2; SLIT3; CACYBP; ATIC; TNFRSF11A; IL7R; DCSTAMP; EDAR; STAT1; STAT4; RAB20; GFPT2; ASAH1; EGR1; CSF1R; TXK; CCR8; CALM1; PARP16; EIF4E2; VAMP3; TRAF5; WBP1L; IRAK2; ANKRD1; PARP9; RPLP0; OCSTAMP; SRC; CNOT9; PID1; ZYX; TCIRG1; LSP1P1; APOA1; ADIPOR1; STK39; NRP2; STAT3; MAPK14; MAPK13; PIM1; GSN; HSPA5; SH2B2; CDC42EP4; CCL4; TRIM25; SOX9 |
| 14 | 6.65 | GO:0043123 | Positive regulation of canonical NF-kappab signal transduction | 6.59E-03 | RETREG1; SMPD1; SNX10; USO1; RAB2A; FHDC1; COG5; NDRG1; STX10; CLU; MICALL1; SYT1; AP3B1; A4GALT; BAIAP2L2; TRPC5; TMEM38B; SNX3; TMCO1; PLN; TOM1; LARGE1; RTN4; SQSTM1; DES; CAV3; CORO7; CSRP3; EMC7; TMEM43; PTPRN; BAIAP2L1; LNPK; PACSIN3; STX2; LPCAT3; PLEKHF1; MTSS2; ESYT1; TMEM9; SERPINE2; GORASP1; RAB7L1; AQP1; GSN; SURF4; TLCD1 |
| 14 | 5.44 | GO:0043410 | Positive regulation of MAPK cascade | 6.06E-03 | CD109; SNX10; TNFRSF11B; WISP1; TNFAIP6; TNFRSF11A; DCSTAMP; CSF1R; GPR68; OCSTAMP; SRC; TCIRG1; CD81; ANXA2; UBASH3B; MAPK14 |
| 14 | 7.57 | GO:1901184 | Regulation of ERBB signaling pathway | 5.44E-03 | COG5; COPB1; COPB2; COPG1; COPA; CUX1 |
| 15 | 5.41 | GO:0019221 | Cytokine-mediated signaling pathway | 5.95E-03 | RETREG1; MGMT; PDK4; CAMK1D; PRKCQ; ITGB1; ARHGAP10; RPS3A; TERT; MRE11; ST3GAL1; HSP90B1; CLU; SLC35F6; ACAA2; CD38; FZD3; CREB3; TMBIM4; IL7R; KDM1A; RPS6KA1; MMP9; PPT1; HMOX1; HTR2B; LGMN; RPS6; NUP62; SH3RF2; CSF1R; ENO1; TNIP2; PRNP; TMBIM1; EGR3; ADAM8; ANKRD1; BFAR; CYR61; THBS1; IVNS1ABP; TNFRSF18; GREM1; SRC; ITGA6; MAPK8IP1; USP47; ITGA5; EYA2; LIMS2; CCND2; HYOU1; CAT; HRAS; NCKAP1L; SCG2; PIM1; AQP1; BAK1; TOR4A; SHC1; HSPA5; MCL1; CRLF1; HSPB1; SOX9 |
| 15 | 7.25 | GO:0035924 | Cellular response to vascular endothelial growth factor stimulus | 2.03E-03 | RGCC; FGF2; THBS1; JUP; SPRED1; GADD45A; ATP2B4 |
| 15 | 7.25 | GO:0070555 | Response to interleukin-1 | 2.97E-03 | HPX; MAP3K7; STX7; PRKCQ; CCR6; CTSC; DUSP22; IL7R; STAT4; C1QC; C1QA; PLA2G4A; UNC93B1; B2M; TCIRG1; CD81; ASCL2; HRAS; NCKAP1L; STAT3; PARP3 |
| 15 | 4.81 | GO:0071345 | Cellular response to cytokine stimulus | 7.02E-03 | CFL2; LAMP2; PLN; LARGE1; CSRP3; GAA |
| 15 | 7.32 | GO:0071346 | Cellular response to type II interferon | 8.63E-05 | MAPK11; IRF8; SYK; THBS1; UNC93B1; CMKLR1; MAPK14 |
| 16 | 7.06 | GO:0030968 | Endoplasmic reticulum unfolded protein response | 1.76E-04 | USP14; DERL1; HSP90B1; UBQLN1; ERLEC1; UBXN4; AMFR; DERL3; UBXN6; HSPA5; RNFT1; NPLOC4; TRIM25 |
| 16 | 7.62 | GO:0038066 | P38mapk cascade | 5.44E-03 | PFN2; MET; LMOD2; PAK1; SYK; C7orf73; CLU; RAP1B; BAIAP2L2; NRG1; FERMT2; FERMT1; ARPC2; SRC; BAIAP2L1; TRABD2B; LMOD1; NCKAP1L; RHOC; BAK1 |
| 16 | 7.12 | GO:0071456 | Cellular response to hypoxia | 2.16E-03 | CLU; HSPA2; DNAJA4; DNAJB6 |
| 16 | 7.73 | GO:1903573 | Negative regulation of response to endoplasmic reticulum stress | 5.20E-03 | PDIA3; LMO7; PLXNB2; RAP2B; NRCAM; FREM2; PCDH17; IBSP; BLB2; RGCC; SPP1; ITGA8; CALR; AP1AR; DSG1; ALCAM; PLXNA4; PRKCQ; ITGB1; ROBO1; CTNNAL1; DUSP1; CELSR1; SEMA4D; PCDH15; CTNNA3; SYK; TNFAIP6; GCNT2; TNN; DUSP22; GRHL2; TLN1; IL7R; AP3B1; TGFBI; TM9SF4; PARVB; CD164; ADAM19; TGM2; FGL1A; BVES; ZDHHC2; RPSAP58; TNIP1; SUSD5; APOD; ADAM9; MXRA8; FERMT2; PRNP; RIN2; FERMT1; VAMP3; ARPC2; EGR3; MAD1L1; PPP1CB; ADAM8; CYR61; LDB1; THBS1; JUP; GREM1; SRC; CD276; SDC4; CSTA; ITGA6; ITGA5; VCL; CD63; B2M; ZYX; LIMS2; DUSP3; CD81; ASCL2; ANXA2; APOA1; CELSR2; DNAJB6; NCAM1; NCKAP1L; UBASH3B; SERPINE2; THY1; MAPK14; CFTII; BSG; PPARD; SHC1; COMP; TNC; VMP1; VTN; SOX9 |
| 17 | 7.1 | GO:0007229 | Integrin-mediated signaling pathway | 7.67E-04 | SUCNR1; MET; PAK1; SDCBP; CALR; FGF2; JCAD; CAMK1D; MAPRE2; ITGB1; CCR6; PLA2G7; SEMA4D; F2RL1; GCNT2; APP; CD99L2; CREB3; NUS1; FGF1; MMP9; HMOX1; LGMN; BVES; SUN2; SH3RF2; RTN4; CSF1R; ADAM9; DDRGK1; FERMT2; RIN2; FERMT1; ADAM8; CYR61; THBS1; PTRF; MCU; MAP2K3; SRC; ITGA6; CMKLR1; ASCL2; CASP8; STK39; HRAS; NCKAP1L; SCG2; THY1; STAT3; AQP1; RHOC; NSMF; HSPA5; VTN; HSPB1; SOX9 |
| 17 | 6.67 | GO:0017015 | Regulation of transforming growth factor beta receptor signaling pathway | 5.91E-03 | USP14; CLU; CREB3; DDRGK1; BFAR; LPCAT3; HSPA5 |
| 17 | 6.28 | GO:0019722 | Calcium-mediated signaling | 5.76E-03 | METTL21C; ATP1B1; ARC; CYBA; CACNA2D1; KCNJ3; F2RL1; TMEM38B; PLN; CALM1; PRNP; KCNAB2; SLC30A1; KCNIP2; GSTO2; CD63; KCNJ5; TCIRG1; KLHL24; STK39; UBASH3B; THY1; BAK1; STOM |
| 17 | 7.81 | GO:0031663 | Lipopolysaccharide-mediated signaling pathway | 8.20E-03 | CRH; SYK; CYBA; THBS1; PID1; GADD45A; MAPK14; DCXR |
| 18 | 7.52 | GO:0010718 | Positive regulation of epithelial to mesenchymal transition | 5.44E-03 | NEU3; MVB12A; MMP9; LGMN; RTN4; RHBDF1; CNOT9; AFAP1L2; RNF126 |
| 18 | 6.73 | GO:0030316 | Osteoclast differentiation | 3.13E-04 | PLA2G7; SCARB1; PLA2G4A; CETP; LCAT; LPCAT3; APOA1; LPCAT2; CHKA |
| 18 | 6.6 | GO:0045667 | Regulation of osteoblast differentiation | 4.87E-03 | PFN2; RAP2B; HPX; PAK1; SDCBP; FGF2; BANK1; MUSK; MRE11; SEMA4D; SYK; APP; PRR5; FGF1; MMP9; CSF1R; HSPA2; DDRGK1; PRNP; PARP9; CYR61; THBS1; BCAR3; GREM1; SRC; CNOT9; BTBD10; ITGA6; FGF6; ITGA5; CCND2; HRAS; NCKAP1L; ATP2B4; CRLF1; VTN; SOX9 |
| 19 | 3.21 | GO:0009888 | Tissue development | 4.81E-03 | PPP1R9A; ATP1B1; MYOZ2; CCR6; RCAN2; SYK; APP; NCALD; TMBIM4; RCAN1; TMEM38B; C3AR1L; PLN; HTR2B; HOMER2; SLC9A1; CCR8; CALM1; PRNP; LMCD1; MCU; CMKLR1; ATP2B4 |
| 20 | 6.67 | GO:0045766 | Positive regulation of angiogenesis | 5.91E-03 | PFN2; MAPK11; RGCC; CALR; SOX11; FGF2; TGFB3; BANK1; ITGB1; ID4; ROBO1; CTSC; SAMSN1; MTMR2; TNFRSF11B; WISP1; TNFAIP6; DUSP22; RNF128; RGS2; STAT1; TP63; PLN; LGMN; FGL1A; EVL; HOMER2; LRPAP1; RTN4; TNMD; C1QC; APOD; RAI1; GPR68; PRNP; FERMT1; REL; CAV3; MAD1L1; PGAM5; THBS1; JUP; GREM1; PTHLH; CD276; SDC4; CMKLR1; SPRED1; DHX58; B2M; GADD45A; DNAJA4; DUSP3; CHID1; ASCL2; DNAJB11; ANXA2; APOA1; ADIPOR1; ATP1A1; STK39; NCKAP1L; UBASH3B; SERPINE2; THY1; YWHAH; GORASP1; IL17RD; RASSF5; WNT2B; ATP2B4; PARP3; PTGDS; HGS; SERPINF1; NPLOC4; SOX9; SERPINF2 |
| 21 | 5.97 | GO:0060348 | Bone development | 9.18E-03 | RGCC; TERT; NDRG1; ACAA2; HMOX1; UBQLN1; ENO1; ADAM8; DDAH1; HYOU1; AQP1; PPARD; TBL2 |
| 22 | 7.44 | GO:0002690 | Positive regulation of leukocyte chemotaxis | 2.74E-03 | SMPD1; NRCAM; COLQ; USO1; RUBCNL; SEC31A; NPC2; STX7; SEC23A; SCLT1; SPIRE2; GBA2; NDRG1; STX10; CLU; SLC35F6; ACAA2; MICALL1; DOCK2; SYT1; AP3B1; A4GALT; DCSTAMP; BAIAP2L2; LAPTM5; EEA1; HSPA4; TRPC5; RAB20; SNX3; TMCO1; PPT1; TOM1; LARGE1; PLTP; RTN4; PRNP; DES; VAMP3; AP2M1; CAV3; KCNIP2; CSRP3; GLTP; THBS1; EMC7; TMEM43; BAIAP2L1; PACSIN3; STX2; LPCAT3; EYA2; MTSS2; ANXA2; APOA1; ESYT1; VAMP2; NCKAP1L; BAK1; GSN; RAC3; TLCD1; HGS; GOSR1 |
| 22 | 7.67 | GO:0071677 | Positive regulation of mononuclear cell migration | 9.39E-04 | CCR6; APP; CD99L2; APOD; ADAM8; ASCL2; STK39 |
| 22 | 9.01 | GO:0090025 | Regulation of monocyte chemotaxis | 8.69E-03 | DUSP22; LIPA; PRNP; DUSP3; CD81; THY1; RAB7L1 |
| 22 | 8.13 | GO:1902622 | Regulation of neutrophil migration | 2.34E-03 | MCUB; FGF2; METTL21C; ATP1B1; CYBA; F2RL1; APP; LAMP2; AP3B1; SLC9A2; TM9SF4; ATP6V0D2; TMEM38B; RAB20; ANXA6; TMCO1; PPT1; PLN; HMOX1; HTR2B; TGM2; SLC9A9; SLC9A1; CALM1; ERO1A; CAV3; SLC30A1; CSRP3; GSTO2; MCU; MICU1; SLC39A13; ATP6V1A; MINPP1; B2M; TCIRG1; ANXA2; TMEM9; ATP1A1; UBASH3B; FTH1; THY1; CACNA1S; ATP2B4; BAK1 |
| 22 | 8.17 | GO:2000404 | Regulation of T cell migration | 2.34E-03 | DUSP1; PLA2G7; CREB3; LGMN |
| 23 | 6.33 | GO:0010634 | Positive regulation of epithelial cell migration | 7.27E-03 | SUCNR1; BLB2; SOX11; TPD52; PRKCQ; BANK1; CCR6; IRF8; CTSC; SAMSN1; SYK; NDRG1; ST3GAL1; CLU; CD180; CD38; DOCK2; APP; DUSP22; IL7R; AP3B1; DCSTAMP; TNFSF13B; STAT4; ITK; FGL1A; RPS6; EGR1; DNASE1; C1QA; ADAM9; LIPA; SMAD3; PRNP; EGR3; MAD1L1; ADAM8; PLA2G4A; THBS1; CD276; SDC4; MAPK8IP1; B2M; CD79B; TCIRG1; DUSP3; CD81; ASCL2; STK39; NCKAP1L; THY1; STAT3; PRDX1; RAB7L1; RASSF5; PARP3; BAK1; GSN; PTGDS; LAT2 |
| 23 | 8.17 | GO:0043537 | Negative regulation of blood vessel endothelial cell migration | 1.73E-03 | RGCC; FGF2; THBS1; JUP; SPRED1; GADD45A; ATP2B4 |
| 23 | 8.82 | GO:0051546 | Keratinocyte migration | 2.66E-03 | CALR; PLA2G7; APP; CD99L2; CREB3; LGMN; ADAM8; ASCL2; STK39; |
| 23 | 8.36 | GO:2001026 | Regulation of endothelial cell chemotaxis | 1.26E-03 | JCAD; ITGB1; ROBO1; NUS1; EGR3; MAP2K3; CD63; NRP2; MAPK14; ATP2B4; HSPB1 |
| 24 | 5.26 | GO:0006816 | Calcium ion transport | 7.69E-03 | EIF3A; EIF3E; EIF3L; NMNAT2; RPL18A; RPS14; RPL31; RPSAP58; RPS6; RPL9; PARP16; RPL6; RPL19 |
| 24 | 8.22 | GO:0010880 | Regulation of release of sequestered calcium ion into cytosol by sarcoplasmic reticulum | 5.78E-03 | MAPRE2; MMP9; ADAM9; FERMT1; PPARD |
| 24 | 6.35 | GO:0034765 | Regulation of monoatomic ion transmembrane transport | 9.94E-03 | DFNA5; FAM150B; FGF2; JCAD; BANK1; GADD45G; MAP4K1; SYK; F2RL1; DNAJC27; GCNT2; APP; DUSP22; TNFRSF11A; RAP1B; EDAR; FGF1; NRG1; HTR2B; SH3RF2; CSF1R; FERMT2; ADAM8; ARL6IP5; BCAR3; MAP2K3; SRC; MAPK8IP1; DOK4; GADD45A; CD81; STK39; HRAS; SHC1; CCL4; SERPINF2 |
| 24 | 6.07 | GO:0071805 | Potassium ion transmembrane transport | 2.79E-03 | TPP1; MFSD8; LAMP2; SLC17A9; TCIRG1; CD81 |
| 24 | 7.23 | GO:0086009 | Membrane repolarization | 1.47E-03 | ATP1B1; CTNNA3; CACNA2D1; RGS2; TMEM38B; PLN; LARGE1; TNNC2; SLC9A1; CALM1; KBTBD13; CAV3; CSRP3; JUP; MAP2K3; KCNJ5; ATP1A1; KCND3; GSN; GAA |
| 24 | 7.73 | GO:1901379 | Regulation of potassium ion transmembrane transport | 3.11E-03 | INPPL1; MAPK11; SNX10; RAB23; SEMA4D; NPR2; WISP1; TNN; TMEM38B; LARGE1; GPR68; LIPA; IFITM5; DHRS3; TTC9; GREM1; PTHLH; SRC; ANXA2; TF; MAPK14; SOX9 |
| 25 | 8.09 | GO:0006891 | Intra-Golgi vesicle-mediated transport | 2.67E-03 | AP1AR; BVES; VAMP3; ANXA2; RAB7L1; TBC1D16 |
| 25 | 5.08 | GO:0006897 | Endocytosis | 4.33E-03 | SUCNR1; GRPR; HPX; MAP3K7; LY96; ALPK1; IPO5; CTSC; SYK; CYBA; WISP1; C7orf73; APP; TNFRSF11A; AP3B1; TLR1A; UBQLN1; RTN4; TXK; SQSTM1; PARP1; TNIP2; IRAK2; ADAM8; PARP9; UNC93B1; SRC; DHX58; CD81; TF; LRSAM1; MAPKAPK3; NPLOC4; TRIM25 |
| 25 | 6.94 | GO:0006906 | Vesicle fusion | 9.31E-03 | SUCNR1; MAP3K7; TRIM55; CRH; ITGB1; CCR6; CTSC; WFDC1; SYK; CYBA; WISP1; TNFAIP6; C7orf73; CLU; F2RL1; APP; TNFRSF11A; AP3B1; HMOX1; LARGE1; DNASE1; C1QA; CTNNBIP1; APOD; LIPA; SMAD3; REL; ADAM8; F3; CSRP3; THBS1; MAP2K3; CD276; CMKLR1; AFAP1L2; POMGNT1; LPCAT3; ALOX5; TCIRG1; CHID1; CD81; APOA1; STK39; STAT3; MAPK14; PPARD; TNC; CCL4 |
| 25 | 4.59 | GO:0140352 | Export from cell | 8.14E-03 | IRAK4; MAP3K7; ALPK1; F2RL1; EDAR; HTR2B; NUP62; PARP1; TNIP2; TRAF5; REL; TF; CASP10; CASP8; TRIM25 |
| 26 | 7.21 | GO:0032370 | Positive regulation of lipid transport | 2.44E-03 | HPX; DFNA5; MAP3K7; LY96; ALPK1; IPO5; SYK; CYBA; C7orf73; CD180; LY86; AP3B1; TLR1A; UBQLN1; RTN4; TXK; SQSTM1; PARP1; TNIP2; IRAK2; ADAM8; PARP9; UNC93B1; SRC; DHX58; TF; MAPKAPK3; NPLOC4; TRIM25 |
| 26 | 9.01 | GO:0043691 | Reverse cholesterol transport | 8.69E-03 | METTL21C; TMEM38B; PLN; CALM1; GSTO2 |
| 27 | 8.13 | GO:0006622 | Protein targeting to lysosome | 8.58E-03 | SCARB1; CETP; LCAT; APOA1 |
| 27 | 4.78 | GO:0006886 | Intracellular protein transport | 3.80E-04 | PFN2; MET; LMOD2; PAK1; RGCC; TGFB3; CFL2; WDR1; BAIAP2L2; NUP62; EVL; DYNC1H1; GPR65; FERMT2; ARPC2; KATNBL1; BAIAP2L1; APOA1; LMOD1; NCKAP1L; RHOC; GSN; SERPINF2 |
| 27 | 5.87 | GO:0072659 | Protein localization to plasma membrane | 7.94E-03 | SUCNR1; PTX3; HPX; IRAK4; RGCC; MAP3K7; STX7; TGFB3; CCR6; IRF8; CTSC; PANX1; SYK; WDR1; ST3GAL1; CLU; CD180; DOCK2; APP; DUSP22; IL7R; STAT4; RPS6; DNASE1; C1QC; C1QA; LIPA; UNC93B1; DHX58; B2M; TCIRG1; CD81; ASCL2; APOA1; NCKAP1L; STAT3; PRDX1; PARP3; PTGDS |
| 28 | 6.47 | GO:0030041 | Actin filament polymerization | 2.41E-03 | MAPK11; RGCC; MAP3K7; LY96; PRKCQ; IRF8; PANX1; SYK; CYBA; C7orf73; CLU; F2RL1; APP; RUNX1; STAT1; ITK; HTR2B; EGR1; CSF1R; EIF2AK3; TXK; FERMT1; ADAM8; F3; THBS1; UNC93B1; CD276; AFAP1L2; DHX58; B2M; CD81; CASP8; HRAS; STAT3; MAPK14; MAPK13; WNT2B; HSPB1; SERPINF2 |
| 28 | 7.46 | GO:0045010 | Actin nucleation | 5.72E-04 | ITGB1; WISP1; F2RL1; FERMT2; FERMT1; THBS1; HRAS; SERPINF2 |
| 28 | 8.13 | GO:0045214 | Sarcomere organization | 4.05E-03 | PFN2; PAK1; RGCC; TGFB3; EVL; GPR65; FERMT2; APOA1; RHOC; SERPINF2 |
| 28 | 6.45 | GO:0051495 | Positive regulation of cytoskeleton organization | 6.40E-03 | SUCNR1; PFN2; RAB31; CYB5R4; SDCBP; RGCC; SPP1; SOX11; CRH; GPR158; TGFB3; ATP1B1; PRSS12; UNC13B; SYK; F2RL1; TNFRSF11A; SYT1; ARFGAP3; RAP1B; SCAMP1; TMEM38B; PPT1; TGM2; SLC9A1; SYNGR3; EXOC5; ADAM9; CALM1; GPR68; RAB26; VAMP3; RHBDF1; ANKRD1; STEAP3; KCNIP2; PLA2G4A; PTRF; PTPRN; MCU; SDC4; M6PR; SIDT2; WLS; TCIRG1; ABCB11; ATP1A1; G6PC2; ANO1; NCKAP1L; SERPINE2; COPG1; KCND3; AQP1; PTGDS; HGS; RASL10B; SLC47A2; SLC47A1 |
| 28 | 7.65 | GO:0051496 | Positive regulation of stress fiber assembly | 9.39E-04 | SCARB2; CLU; LAMP2; AP3B1; M6PR; HGS |
| 29 | 4.74 | GO:0043066 | Negative regulation of apoptotic process | 6.91E-03 | PFN2; RAP2B; SMPD1; HPX; PAK1; FGF2; BANK1; MUSK; MRE11; SEMA4D; SYK; DERL1; APP; PRR5; FGF1; KDM1A; MMP9; UBQLN1; TNIP1; EGR1; CSF1R; HSPA2; CALM1; DDRGK1; PRNP; WBP1L; PPP2R5A; PARP9; CYR61; CHFR; BCAR3; GREM1; SRC; CNOT9; FGF6; ITGA5; CCND2; TRABD2B; HRAS; RASSF5; ATP2B4; HSPA5; CRLF1; VTN; SOX9 |
| 29 | 7.5 | GO:0070231 | T cell apoptotic process | 7.42E-03 | LMOD2; MYOZ2; ITGB1; CFL2; WDR1; CAV3; CSRP3 |
| 29 | 8.74 | GO:2000811 | Negative regulation of anoikis | 2.66E-03 | MYOZ2; RCAN1; HOMER2; PRNP; ATP2B4 |
| 30 | 4.4 | GO:0008284 | Positive regulation of cell population proliferation | 3.90E-04 | MAPK11; MAP3K7; DUSP1; GADD45G; CAV3; MAP2K3; GADD45A; STK39; MAPK14 |
| 30 | 6.56 | GO:0031334 | Positive regulation of protein-containing complex assembly | 1.70E-03 | USO1; RUBCNL; STX7; STX10; SYT1; EEA1; RAB20; TOM1; VAMP3; ANXA2; VAMP2; GOSR1 |
| 30 | 8.91 | GO:0034375 | High-density lipoprotein particle remodeling | 9.86E-04 | PTX3; CALR; CAMK1D; CYBA; DOCK2; LMAN2; APOA1; NCKAP1L |
| 30 | 5.37 | GO:0043068 | Positive regulation of programmed cell death | 5.33E-03 | WWTR1; SDCBP; RGCC; TGFB3; GCNT2; SMAD3; FERMT2; ADAM8; SOX9 |
| 30 | 7.87 | GO:0048260 | Positive regulation of receptor-mediated endocytosis | 3.16E-03 | PTX3; SMPD1; SNX3; GSN; TRIM25 |
| 30 | 7.94 | GO:0050766 | Positive regulation of phagocytosis | 1.92E-03 | SYK; CLU; PPT1; GREM1; CD63; B2M; ANXA2; VTN |
| 30 | 8.67 | GO:0060142 | Regulation of syncytium formation by plasma membrane fusion | 4.01E-03 | PLTP; SCARB1; CETP; LCAT; APOA1 |
| 30 | 8.91 | GO:0090083 | Regulation of inclusion body assembly | 8.69E-03 | PANX1; RCAN1; UCHL1; BVES; EGR1; CSF1R |
| 31 | 5.42 | GO:0002683 | Negative regulation of immune system process | 4.70E-03 | DERL1; CREB3; EIF2AK3; ERLEC1; PARP16; DDRGK1; ERO1A; BFAR; AMFR; DERL3; BAK1; HSPA5; TBL2 |
| 31 | 3.87 | GO:0023056 | Positive regulation of signaling | 7.83E-03 | COBL; SCEL; KLF5; WWTR1; PLXNB2; CD109; MET; MAPK11; SNX10; PAK1; FREM2; DFNA5; MATN3; SDCBP; RGCC; ITGA8; RPS7; SOX11; TPP1; FGF2; VIM; SVIL; TGFB3; METTL21C; PLXNA4; COL8A1; MYOZ2; ITGB1; CFL2; ID4; ROBO1; ZNF516; PGRMC2; CELSR1; WDR1; TMOD1; NPR2; WISP1; GCNT2; FZD3; UMODL1; TNN; GCNT4; CTSB; GRHL2; NTN4; MGP; RAP1B; AP3B1; RCAN1; RUNX1; SLC9A2; FGF1; FAM172A; NRG1; STAT1; TMEM38B; TST; TP63; MMP9; PLN; LARGE1; HTR2B; TGM2; ASAH1; BVES; CTSZ; EGR1; RTN4; TNMD; SLC9A1; ASB2; CTNNBIP1; EXOC5; ADAM9; DUSP5; CHRND; DDRGK1; LIPA; SMAD3; FERMT2; FERMT1; RPL3L; ALDH1A3; CAV3; ADAM8; NELL1; ANKRD1; CYR61; IFITM5; LDB1; CSRP3; ANXA7; MMP15; GREM1; PTHLH; SRC; CD276; SDC4; CSTA; ITGA6; SLC39A13; LNPK; FGF6; ITGA5; MYBPC3; VCL; SPRED1; ELOVL1; SIDT2; WLS; EYA2; TCIRG1; MEOX1; ETV4; DZIP1L; ASCL2; ANXA2; APOA1; ADIPOR1; HRAS; UPK1B; SERPINE2; NRP2; MAPK14; IL17RD; PIM1; IRF6; AQP1; WNT2B; PPARD; MUSTN1; CRLF1; TNC; ALDOC; GAA; VTN; SOX9 |
| 31 | 3.83 | GO:0032879 | Regulation of localization | 3.71E-04 | RGCC; MAP3K7; LY96; ALPK1; IPO5; SYK; CYBA; C7orf73; CD38; DUSP22; AP3B1; C3AR1L; ITK; TLR1A; UBQLN1; RTN4; C1QC; SQSTM1; C1QA; PARP1; TNIP2; LIPA; PRNP; IRAK2; UNC93B1; SRC; CMKLR1; DHX58; CD79B; DUSP3; CD81; TF; HRAS; NCKAP1L; THY1; RAB7L1; MAPKAPK3; SH2B2; NPLOC4; TRIM25 |
| 31 | 5.13 | GO:0040017 | Positive regulation of locomotion | 7.89E-03 | LY96; CD180; LY86; IRAK2; SCARB1; TF; MAPK14 |
| 31 | 8.54 | GO:0046596 | Regulation of viral entry into host cell | 8.04E-03 | PRKCQ; ST3GAL1; IL7R; PDCD1; RPS6; LIPA; ADAM8; BAK1 |
| 32 | 5.93 | GO:0007033 | Vacuole organization | 6.15E-03 | COBL; PFN2; LMOD2; FAM49A; TMOD1; GBA2; BAIAP2L2; ARPC2; CORO7; TTC17; ARPC4; BAIAP2L1; LMOD1; NCKAP1L; TWF2; GSN |
| 32 | 5.17 | GO:0010256 | Endomembrane system organization | 5.62E-04 | INPPL1; PTX3; SMPD1; LMBRD1; NEU3; SNX10; RAB31; SDCBP; CALR; SCARB2; CAMK1D; ITGB1; IRF8; STON2; ARC; LRRTM2; ANXA11; SYK; CYBA; CLU; MICALL1; DOCK2; APP; SYT1; LMAN2; TM9SF4; EEA1; RAB20; SNX3; NCF4; PPT1; HTR2B; TGM2; LRPAP1; CANX; LIPA; AP2M1; CAV3; SCARB1; THBS1; GREM1; SRC; PACSIN3; CD63; B2M; DNAJC6; ANKRD13D; CD81; ANXA2; APOA1; HRAS; NCKAP1L; CLTCL1; GSN; LRSAM1; MAPKAPK3; RAC3; VTN |
| 32 | 5.82 | GO:0030198 | Extracellular matrix organization | 3.38E-03 | SNX10; CRH; NPR2; STRAP; LIPA; SCARB1; LPCAT3; APOA1; STK39; AQP1; COPA; SOX9 |
| 32 | 4.84 | GO:0061024 | Membrane organization | 1.75E-04 | WDR45; RAB23; TPP1; FEZ2; MFSD8; NPR2; LAMP2; AP3B1; LAPTM5; RAB20; PPT1; TOM1; UBQLN1; LIPA; GABARAPL1; TCIRG1; CTSD; ANXA2; ATG4B; TMEM9; TMEM39A; LRSAM1; VMP1; GAA |
| 32 | 7 | GO:0150115 | Cell-substrate junction organization | 8.18E-03 | PDK4; SYK; TNFRSF11A; DCSTAMP; ADAM8; UBASH3B |
| 32 | 8.54 | GO:0150146 | Cell junction disassembly | 1.26E-03 | TMEM182; DCSTAMP; ADAM9; OCSTAMP; MAPK14 |
| 33 | 5.23 | GO:0002252 | Immune effector process | 1.05E-03 | PFN2; WWTR1; PTX3; SMPD1; NEU3; RAB23; PCDH17; SDCBP; RGCC; SPP1; CALR; SOX11; SARAF; CRH; FGF2; STX7; GPR158; TGFB3; METTL21C; CAMK1D; ATP1B1; ITGB1; IPO5; STON2; ARC; TERT; LRRTM2; SYK; CYBA; STX10; TNFAIP6; CLU; SLC35F6; CACNA2D1; ACAA2; KCNJ3; F2RL1; POFUT2; DNAJC27; DOCK2; TNFRSF11A; DENND5B; SYT1; LMAN2; BEST3; GOPC; RAP1B; NUS1; RANGAP1; TM9SF4; NRG1; KDM1A; TMEM38B; SNX3; FHL1; PPT1; PLN; HTR2B; TGM2; BVES; PLTP; ZDHHC2; NUP62; LRPAP1; RTN4; DYNC1H1; SQSTM1; ERLEC1; APOD; PARP1; ADAM9; DYNC1LI1; CALM1; GPR68; DDRGK1; ERO1A; FERMT2; CCT7; PRNP; FERMT1; VAMP3; EEPD1; AP2M1; TMBIM1; RHBDF1; CAV3; PPP2R5A; KCNAB2; MAD1L1; ARL6IP5; ANKRD1; PARP9; SLC30A1; SCARB1; KCNIP2; PLA2G4A; CSRP3; THBS1; MMD2; JUP; GSTO2; MCU; GREM1; SRC; SDC4; CETP; VCL; FXYD6; PACSIN3; CD63; SIDT2; PID1; B2M; WLS; KCNJ5; LPCAT3; DNAJC6; TCIRG1; ANKRD13D; ABCB11; KLHL24; CD81; DZIP1L; CTSD; ANXA2; APOA1; CELSR2; DNAJB6; SLC1A2; G6PC2; STK39; ANO1; HRAS; NCKAP1L; UBASH3B; SERPINE2; DERL3; THY1; YWHAH; CLTCL1; MAPK14; RAB7L1; CACNA1S; RASSF5; AQP1; ATP2B4; PPARD; BAK1; GSN; STOM; OAZ1; LRSAM1; SURF4; HGS; ARHGDIA; VTN; TRIM25; RASL10B |
| 33 | 6.09 | GO:0002460 | Adaptive immune response based on somatic recombination of immune receptors built from immunoglobulin superfamily domains | 3.58E-03 | PLN; SLC9A1; CSRP3; ATP1A1; ATP2B4; GAA |
| 33 | 9.01 | GO:0019886 | Antigen processing and presentation of exogenous peptide antigen via MHC class II | 8.69E-03 | CD109; SDCBP; ITGA8; SOX11; LTBP1; STRAP; PMEPA1; CILP; SMAD3; FERMT1; WFIKKN1; CAV3; THBS1; SPRED1; IL17RD; HSPA5 |
| 33 | 4.36 | GO:0045321 | Leukocyte activation | 4.00E-03 | SPP1; CRH; TNFRSF11A; DENND5B; PLTP; EEPD1; PLA2G4A; CETP; LPCAT3; ANXA2; APOA1 |
| 34 | 9.01 | GO:0033689 | Negative regulation of osteoblast proliferation | 8.69E-03 | BLB2; LGMN; UNC93B1; B2M |
| 35 | 6.07 | GO:0006457 | Protein folding | 6.78E-04 | ITGB1; SYK; TLN1; FERMT2; FERMT1; SRC; ITGA5; CD63; ZYX; LIMS2; APOA1; THY1; |
| 35 | 4 | GO:0007155 | Cell adhesion | 4.44E-03 | WWTR1; SOX11; FGF2; SEMA4D; WISP1; TNFAIP6; TNN; TP63; CTNNBIP1; SMAD3; FERMT2; NELL1; CYR61; GREM1; CD276; TCIRG1; TF; SOX9 |
| 35 | 6.23 | GO:0007160 | Cell-matrix adhesion | 1.96E-03 | PTX3; MATN3; RGCC; ITGA8; DPT; COL8A1; ITGB1; IMPG2; TNFRSF11B; APP; TGFBI; TMEM38B; MMP9; LARGE1; COL4A6; VWA1; ERO1A; SMAD3; FERMT1; ADAMTS14; CYR61; GREM1; P3H4; POMGNT1; ANXA2; DNAJB6; VTN; SOX9; SERPINF2 |
| 35 | 7.28 | GO:0061077 | Chaperone-mediated protein folding | 5.63E-03 | ; CLU; CREB3; KDM1A; MMP9; ENO1; IVNS1ABP; SRC; MAPK8IP1; USP47; HYOU1; HSPB1 |
| 36 | 5.52 | GO:0002833 | Positive regulation of response to biotic stimulus | 6.55E-03 | CLU; PDCL3; ERO1A; CCT7; SDF2L1; FKBP4; DNAJB6; FKBP5; HSPA5; HSPB1 |
| 36 | 5.38 | GO:0031349 | Positive regulation of defense response | 4.36E-03 | RAB31; STX7; GPR158; ATP1B1; ITGB1; SEC23A; LARGE1; ZDHHC2; SQSTM1; GGA2; EXOC5; RAB26; PRNP; VAMP3; AP2M1; TMBIM1; CAV3; PPP2R5A; ARL6IP5; CSRP3; JUP; PID1; TSPAN15; PLEKHF1; CD81; GORASP1; BSG; ATP2B4 |
| 36 | 5.15 | GO:0032103 | Positive regulation of response to external stimulus | 4.58E-04 | LMOD2; AP1AR; SPIRE2; ARPC2; ARPC1A; ARPC1B; ARPC4; FMNL1; LMOD1; GSN |
| 36 | 7.46 | GO:0061045 | Negative regulation of wound healing | 7.76E-03 | PDIA3; SMPD1; DFNA5; RPS3; RGCC; RPS7; CAMK1D; ITGB1; CTSC; GADD45G; SYK; TNFAIP8; CTSV; CLU; LAPTM5; TP63; PDCD1; MMP9; HMOX1; TGM2; RPS6; PRNP; RYBP; ALDH1A3; ADAM8; ARL6IP5; SHQ1; ANKRD1; CYR61; BCL10; AIFM2; THBS1; ITGA6; GADD45A; PLEKHF1; SERINC3; CTSD; BAK1; GSN; MCL1 |
| 36 | 7.67 | GO:0090303 | Positive regulation of wound healing | 7.42E-03 | PDIA3; PPIC; CALR; HSPA4L; ERP44; PFDN1; CLGN; HSP90B1; CLU; POFUT2; HSPA4; PDCL3; CANX; HSPA2; ERO1A; CCT7; DNAJA4; SDF2L1; DNAJB11; FKBP4; DNAJB6; FKBP5; HSPA5; FKBP6; HSPB1 |
| 37 | 8.36 | GO:0002931 | Response to ischemia | 5.01E-03 | FGF2; JCAD; ITGB1; TNN; FGF1; HMOX1; PDCL3; VASH2; F3; THBS1; JUP; GREM1; ITGA5; STAT3; AQP1; HSPB1 |
| 37 | 5.14 | GO:0006954 | Inflammatory response | 5.20E-03 | SUCNR1; MET; HPX; MAP3K7; CALR; LY96; ALPK1; FGF2; CAMK1D; IPO5; CTSC; PLA2G7; SYK; CYBA; WISP1; C7orf73; CD180; LY86; APP; CREB3; TNFRSF11A; AP3B1; TLR1A; LGMN; UBQLN1; RTN4; CSF1R; TXK; SQSTM1; PARP1; TNIP2; IRAK2; ADAM8; PARP9; THBS1; MCU; UNC93B1; SRC; CMKLR1; DHX58; CD81; TF; STK39; NCKAP1L; SCG2; LRSAM1; MAPKAPK3; NPLOC4; TRIM25; HSPB1; SERPINF2 |
| 37 | 8.6 | GO:0035994 | Response to muscle stretch | 1.54E-04 | TGFB3; MAPRE2; ITGB1; C1QB; C1QC; C1QA |
| 38 | 5.21 | GO:0030003 | Intracellular monoatomic cation homeostasis | 3.30E-03 | MAPRE2; DUSP22; TLN1; APOD; FERMT2; TNS1; LDB1; GREM1; SRC; ITGA5; VCL; THY1 |
| 38 | 8.26 | GO:0046716 | Muscle cell cellular homeostasis | 6.62E-03 | PANX1; C7orf73; APP; EGR1; CASP8; STAT3; HSPB1 |
| 39 | 8.13 | GO:0045124 | Regulation of bone resorption | 8.58E-03 | SOX11; CORO7; CIT; MAP2K3; SRC; MAPK14 |
| 39 | 4.45 | GO:0051241 | Negative regulation of multicellular organismal process | 3.77E-03 | MET; FGF2; FGF1; LGMN; THBS1; HSPB1 |
| 40 | 8.31 | GO:0002026 | Regulation of the force of heart contraction | 1.87E-03 | ATP1B1; KCNG2; KCNJ3; KCNS2; SLC9A2; SLC12A5; TMEM38B; LARGE1; SLC9A9; PRNP; KCNK2; KCNAB2; KCNK17; KCNIP2; LRRC38; CD63; KCNJ5; KCNK15; KLHL24; ATP1A1; STK39; KCND3; KCNN1 |
| 40 | 6.27 | GO:0006941 | Striated muscle contraction | 5.40E-03 | PDK4; ITGB1; SRC; ITGA5; MCL1 |
| 40 | 7.05 | GO:0022600 | Digestive system process | 4.71E-03 | TNN; SMAD3; NELL1; GREM1 |
| 40 | 7.3 | GO:0055117 | Regulation of cardiac muscle contraction | 9.18E-03 | ATP1B1; WDR1; CACNA2D1; CAV3; KCNIP2; KCNJ5; ATP1A1; KCND3 |
| **Enriched GO termsrelated genes up-regulated in the pHu+ or pHu- line** | | | | | |
| 14 | 3.99 | GO:0009967 | Positive regulation of signal transduction | 4.07E-03 | [pHu-] NETO1; FGFR2; CAV2; CAV1; CA8; DDX1; CALCR; FRMD1; GUCY1A2; FLT1; AKAP12; TEK; KL; HMGB1; CDH5; GUCY1A1; INTU; DLL1; RTKN2; DNM1L; KITLG; USP15; UBR5; CDC73; PDGFB; AMER1; ARHGAP8; DEPDC1B; KDR; SFPQ; WASF1; GHR; ESM1; TMEM33; UBE3A; XRCC3; CREBBP; FRMD7; PRKCH; LPAR4; JAG1; SHOC2; WDR24; PLCB1; RALB; JAG2; PRKCZ; MAPK8; PDE8A; FBXL15; DLL4; GPR155; NR1H3; STOX1; PTK6; BOK; IRS1; USP1; SULF2; ZNF423; SIAH1; VWF; BMPR2; FGF4; CRKL; KLHL22; ADORA2A; NOTCH1; RXRA; NRARP; RFNG; PRKCA; RPS6KB1; MAP2K6  [pHu+] PDIA3; SCEL; HPX; IRAK4; NEU3; PAK1; DFNA5; ATP6V1C2; RPS3; SDCBP; MAP3K7; ITGA8; CALR; RPS7; SOX11; FAM150B; LY96; ALPK1; TRIM55; FGF2; JCAD; MAPRE2; BANK1; ITGB1; ROBO1; CTSC; DCDC2; TERT; SEMA4D; GADD45G; MAP4K1; SYK; CYBA; WISP1; C7orf73; CTSV; CD180; F2RL1; DNAJC27; GCNT2; LY86; APP; DUSP22; TNFRSF11A; RAP1B; IL7R; EDAR; PRR5; FGF1; NRG1; GKAP1; TP63; MMP9; HTR2B; NUP62; SH3RF2; RTN4; SLC9A1; CSF1R; TXK; PARP1; TNIP2; DYNC1LI1; CALM1; GPR65; LIPA; FERMT2; FERMT1; TRAF5; REL; LMCD1; CORO7; MAD1L1; ADAM8; ARL6IP5; ANKRD1; PARP9; CYR61; BCL10; F3; THBS1; MMD2; BCAR3; MAP2K3; GREM1; SRC; CNOT9; MAPK8IP1; USP47; DOK4; AFAP1L2; SPRED1; CD63; DHX58; GADD45A; WLS; PLEKHF1; LIMS2; SERINC3; CD81; CTSD; TF; APOA1; CASP10; CASP8; CAT; TMEM9; STK39; HRAS; NCKAP1L; STAT3; RAB7L1; PIM1; WNT2B; DDR1; PPARD; BAK1; SHC1; MCL1; CCL4; TRIM25; SOX9; WBP2; SERPINF2 |
